# Development of a Cross‐Conjugated Vinylogous [4+2] Anionic Annulation and Application to the Total Synthesis of Natural Antibiotic (±)‐ABX

**DOI:** 10.1002/anie.201914657

**Published:** 2020-02-25

**Authors:** Jing‐Kai Huang, Kak‐Shan Shia

**Affiliations:** ^1^ Institute of Biotechnology and Pharmaceutical Research National Health Research Institutes 35 Keyan Road Zhunan Miaoli County 35053 Taiwan, R.O.C.

**Keywords:** acceptor, anionic annulation, benzylogous, donor, vinylogous

## Abstract

The cross‐conjugated vinylogous [4+2] anionic annulation has been newly developed, the cascade process of which has a high preference for regiochemical control and chemoselectivity, giving rise to exclusively Michael‐type adducts in moderate to high yields (up to 94 %, 35 examples). By making use of this approach as a key operation, the first total synthesis of natural antibiotic ABX, in racemic form, has been successfully achieved in a concise 7‐step sequence with an overall yield of about 20 %.

## Graphical abstract

**Novel vinylogous donors** have been developed to carry out a unique [4+2] cascade process in a completely regio‐ and chemoselective manner, by which the first total synthesis of natural antibiotic ABX, in racemic form, was accomplished in a rather concise fashion. This versatile methodology may have great potential in synthesizing polyaromatic compounds and heterocycles.

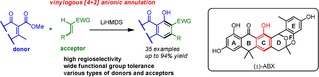

The [4+2] anionic annulation has been widely applied to the synthesis of a variety of structurally complex molecules especially for type II polyketide natural products.^1^ As highlighted in red in the bioactive molecules displayed in Figure 1,^2^, ^3^, ^4^, ^5^, ^6^ the tandem annulation process starts with 1,4‐conjugate addition of a nucleophilic donor to an enone acceptor followed by Dieckmann–Claisen condensation to mainly generate a new six‐membered ring in a one‐pot operation, which indeed, is difficult for other synthetic methods to accomplish in such an efficient manner.

**Figure 1:**
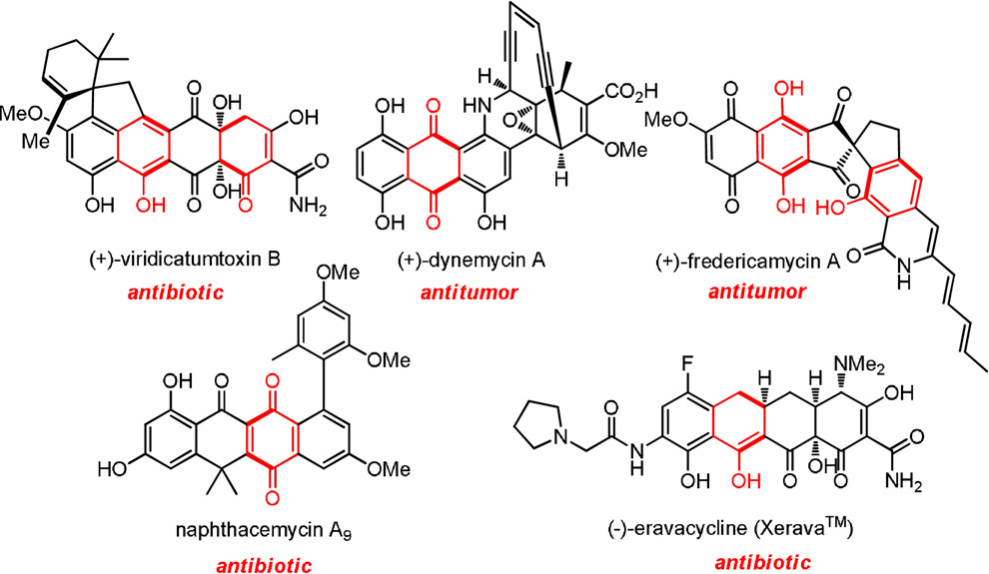
Bioactive molecules synthesized through [4+2] anionic annulation.

In addition to regular Michael‐type [4+2] anionic annulation, Mannich‐ and Aldol‐type annulation are also well documented and can be viewed as the coupling of a 1,4‐dipolar and 1,2‐dipolar synthon as shown in Figure 2 a.^7^, ^8^ Some typical benzylogous donors, comprehending both phthalide and toluate donors, are selected from many historical cases and summarized in Figure 2 b.^1^, ^9^, ^10^ More specifically, while following the Michael‐type cascade process, Hauser–Kraus donors are featured by the formation of a *para*‐benzoquinone ring and others, including Staunton–Weinreb, Sammes, Swenton, and Tamura donors, are noted for the rapid construction of a cyclohexanone ring. The present work disclosed in Figure 2 c is aimed to develop hitherto unknown cross‐conjugated vinylogous donors, which are conceptually considered equivalent and/or complementary to the corresponding benzylogous counterparts, to facilitate Michael‐, Mannich‐, and Aldol‐type annulation processes, furnishing structurally diverse molecules, which are hard to be provided by any other synthetic elaborations. Practically, when making use of this newly developed methodology as a key operation, we have completed the first total synthesis of a complex natural product (±)‐ABX in a rather concise manner, details of which are presented as follows.

**Figure 2:**
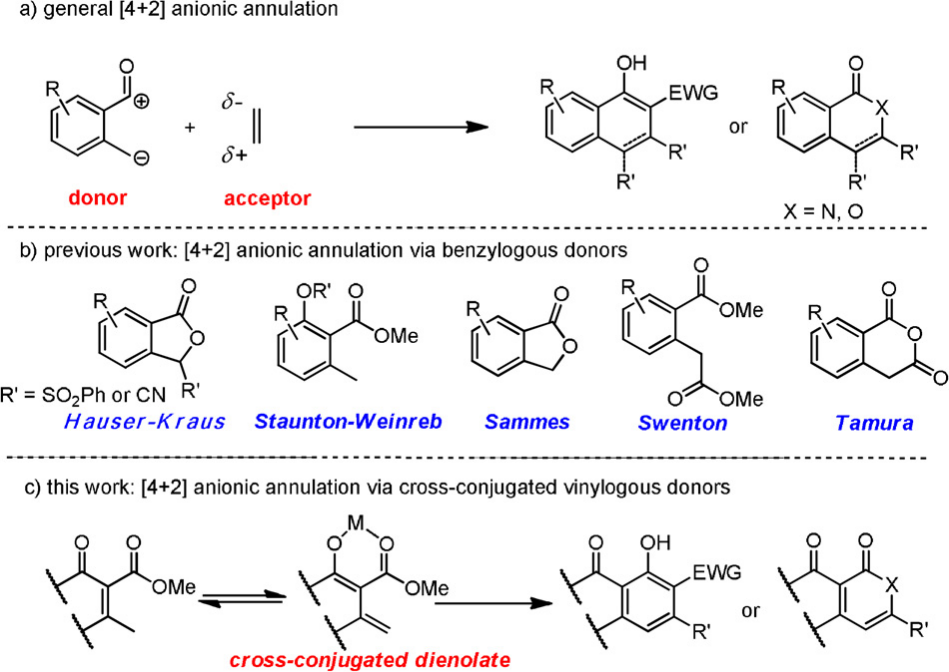
a) General [4+2] anionic annulation. b) Benzylogous donors. c) Cross‐conjugated vinylogous donors.

ABX, also known as BE‐24566B and L755805, was first isolated in 1995 from a culture of actinomycete (MA7150) by Lam and co‐workers and was claimed to be a natural antibiotic against Gram‐positive strains including drug‐resistant bacteria VRE (vancomycin‐resistant enterococci) and MRSA (methicillin‐resistant *Staphylococcus aureus*).^11^

According to retrosynthetic analysis illustrated in Scheme 1, (±)‐ABX could be simply disconnected into two major fragments **1** and **2** through pathway A or fragments **3** and **4** through pathway B. Fragments **1** and **3** are anticipated to serve as novel vinylogous donors to execute similar Michael‐type annulation to traditional benzylogous donors because they all possess an acidic *γ* proton which should be deprotonated by an appropriate base to form the corresponding nucleophilic dienolates as illustrated in Figure 2 c. Approach A was preferentially taken owing to our previously substantial experience in preparation of a variety of cross‐conjugated dienophiles for the Diels–Alder reaction.^12^ The current work began with synthesizing enone ester **5**, bearing a high structural similarity to **1**, as a model compound for the proof‐of‐concept (Scheme 2). Commercially available tetralone **6** was first treated with dimethyl carbonate in the presence of sodium hydride to form the corresponding β‐keto ester which, without purification, was oxidized with DDQ to afford enone ester **7** in 89 % yield over two steps.

**Scheme 1:**
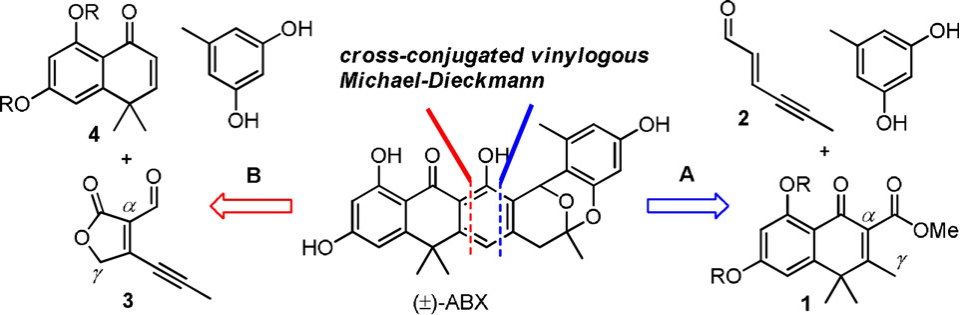
Retrosynthetic analysis of (±)‐ABX.

**Scheme 2:**
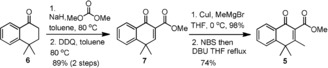
Preparation of cross‐conjugated vinylogous donor **5**.

Compound **7** was then subjected to 1,4‐conjugate addition with MeMgBr under catalysis with CuI to afford β‐methyl keto ester (98 %), which sequentially underwent bromination and 1,2‐elimination to accomplish the desired target **5** in 74 % yield over two repeated cycles. Compound **5**, a potential vinylogous donor, and methyl acrylate, a typical Michael acceptor, were then used to test the proposed Michael‐type [4+2] anionic annulation.

Various combinations of reaction parameters, such as solvents, bases, and temperature elevation, were explored to screen the optimal reaction conditions. Results are compiled in Table 1 and discussed thereby. As indicated in entries 1–5, originally, we thought the γ proton should be quite acidic, and thus weak bases, such as Li_2_CO_3_, Na_2_CO_3_, K_2_CO_3_, DBU, and Cs_2_CO_3_, were attempted. As a result, the expected product **8** (entry 2) was merely obtained in 18 % yield while Na_2_CO_3_ was used at elevated temperature (150 °C) in DMF, suggesting that stronger bases might be required to drive the reaction forward. As such, LiHMDS, NaHMDS, KHMDS, and NaH (entries 6–11) were tried. To our delight, reactions could be significantly improved, giving rise to the desired product **8** in 24–75 % yields at much lower temperature (66–110 °C). Reaction conditions shown in entries 6 (63 %) and 10 (75 %) seem to be promising and deserve to be further modified. In one approach, when the amount of base (LiHMDS) and Michael acceptor in entry 10 was reduced to 1.1 equiv, the reaction system in entry 11 (83 % yield) was tentatively considered optimum and adopted as a standard protocol for the newly developed Michael‐type annulation process. Medium‐strong bases, including ^*t*^BuOLi, ^*t*^BuONa, and ^*t*^BuOK, were also tested based on the standard conditions, results of which again suggested that the base with the lithium counter cation could afford product **8** in the highest yield (entry 12; 79 %, ^*t*^BuOLi). These results along with a wealth of well‐documented literature strongly support that lithium bases appear superior to others presumably due to their multifunction Li^+^ cation, allowing it to be effectively chelated with both dienolate ion and α‐ester carbonyl after enolization (Figure 2 c) and activate oxygen‐containing electrophiles through its strong oxophilicity, thereby not only initiating 1,4‐conjugate addition but also facilitating the subsequent ring closure.1b, 13 To examine the generality of this newly developed method, many structurally diverse vinylogous donors and Michael acceptors were randomly paired off and desired products were commonly achieved in moderate to high yields (36–94 %) as listed in Table 2. To make Michael‐type adducts more readable, those parts of products highlighted in blue are derived from vinylogous donors, which contain the 1‐tetralone, verbenone, coumarin, chromone, 2‐quinolone, γ‐lactam, or γ‐lactone moiety, and those in green are from Michael acceptors, containing a α,β‐unsaturated ketone, lactone, aldehyde, ester, phosnate, sulfone, or nitrile unit. We observed that some vinylogous donors are much more reactive than the model donor **5**, and the annulation process can be affected at lower temperature (0–50 °C) and shorter reaction time to obtain the corresponding non‐aromatized intermediates instead. In those cases, further dehydrogenation with a stronger oxidant DDQ rather than mild dry air (O_2_) was required to reach the final aromatized products as exemplified with adducts **15**–**17**, **23**, and **34**. Some Michael acceptors highlighted in green in adducts **11**, **18**, **26, 27**, **32**, **38**, and **42** are quinone monoketals, which have been known to possess redox activity,^14^ and could play dual functions to provide both desired and redox products. Taking acceptor **43** as an example (Scheme 3 a), when it was coupled with donor **5** under standard conditions, the regular product **11** was formed in 51 % yield accompanied with a redox product **44** in 44 % yield. After careful examination, similar redox property was also found in α,β‐unsaturated tetralone acceptors that we used to generate adducts **24**, **25**, and **33**. As evidenced by a typical example (Scheme 3 b), product **33** and a reduced byproduct **47** were isolated in 48 % and 42 % yield, respectively, under standard conditions. However, when charged with 2.1 equiv of acceptor **46**, the reaction reached completion and the desirable product **33** was obtained in 71 % yield.

**Scheme 3:**
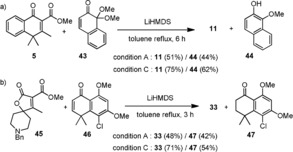
Some Michael acceptors also play a role as an oxidant in [4+2] annulation.

**Table 1:** Screening of vinylogous [4+2] annulation conditions. 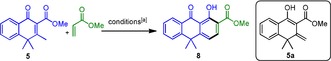

Entry	Michael acceptor	Base (equiv)	Solvent	*T* [°C]/*t* [h]	Isolated yield
1.	(1.5 equiv)	Li_2_CO_3_ (1.5 equiv)	DMF	150 °C/15 h	trace^[b]^
2.	(1.5 equiv)	Na_2_CO_3_ (1.5 equiv)	DMF	150 °C/15 h	18 %^[b]^
3.	(1.5 equiv)	K_2_CO_3_ (1.5 equiv)	DMF	150 °C/2 h	trace
4.	(1.5 equiv)	Cs_2_CO_3_ (1.5 equiv)	DMF	150 °C/2 h	0 %
5.	(1.5 equiv)	DBU (1.5 equiv)	toluene	110 °C/6 h	0 %^[c]^
6.	(1.5 equiv)	LiHMDS (1.5 equiv)	THF	66 °C/15 h	63 %^[b]^
7.	(1.5 equiv)	NaHMDS (1.5 equiv)	THF	66 °C/2 h	44 %
8.	(1.5 equiv)	KHMDS (1.5 equiv)	THF	0–66 °C/2 h	25 %
9.	(1.5 equiv)	NaH (2.0 equiv)	THF	66 °C/15 h	24 %
10.	(1.5 equiv)	LiHMDS (1.5 equiv)	toluene	110 °C/15 h	75 %
11.	(1.1 equiv)	LiHMDS (1.1 equiv)	toluene	110 °C/15 h	83 %
12.	(1.1 equiv)	^*t*^BuOLi (1.1 equuiv.)	toluene	110 °C/6 h	79 %
13.	(1.1 equiv)	^*t*^BuONa (1.1 equiv)	toluene	110 °C/6 h	61 %
14.	(1.1 equiv)	^*t*^BuOK (1.1 equiv)	toluene	110 °C/6 h	trace

[a] All reactions were performed in solvent (0.2 m) as indicated above under dry air. [b] Donor **5** and its dienol tautomer **5 a** were not completely consumed and recovered. [c] Only donor **5** was recovered.

**Table 2:** Scope of Michael‐type [4+2] anionic annulation.

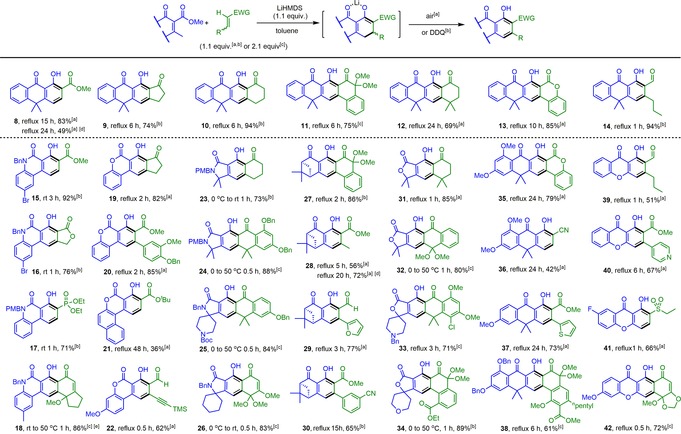

[a] Condition A: All reactions were performed in toluene (0.2 m) under dry air. [b] Condition B: Instead of air, DDQ was used as an oxidant. [c] Condition C: 2.1 equiv of the Michael acceptor was used. [d] A linear acetylenic Michael acceptor was used. [e] THF was used instead to increase the poor solubility of the donor of compound **18**.

To provide the mechanistic insight into the cascade annulation, a typical reaction under condition A was then carried out but quenched at 0 °C before heating. Consequently, donor **5** and its dienol tautomer **5 a** (1:9), as determined by ^1^H NMR of the crude mixture,^15^ was completely recovered, suggesting that deprotonation did occur but the Michael–Dieckmann sequence was not activated. Additionally, no 1,4‐conjugate addition intermediates had been identified for many other vinylogous donors that we used to generate adducts **15**–**17**, **23**–**26, 32**, and **34** (Table 2), wherein all reactions were conducted and completed at lower temperature (0–50 °C). Taken together, these results imply that Michael addition is very likely to be a rate‐limiting step in the above domino reactions; once it is initiated, the ensuing Dieckmann condensation might take place spontaneously. Structurally, though vinylogous donors potentially suffer self‐condensation through their cross‐conjugated dienolates after deprotonation,1c, 16 however, no self‐condensation products have been detected in all cases (35 examples) examined under reaction condition A, B, and C in Table 2. According to retrosynthetic analysis shown in pathway A (Scheme 1), fragments **1** and **2** are apparently two feasible chemical elements to implement the Michael‐type [4+2] anionic annulation which had been experimentally realized in many cases as shown in Table 2. Encouraged by these results, we decided to carry out the total synthesis of (±)‐ABX based on the original design (Scheme 4). Starting with *bis*‐benzyl protected tetralone **48**, enone ester **50** was readily prepared in 57 % over 4 steps through a similar synthetic sequence for enone ester **5** as depicted in Scheme 2. Vinylogous donor **50** was then treated with aldehyde **2** under standard conditions to afford tricyclic aldehyde **53** in 53 % yield. Aldehyde **2** was readily prepared in 78 % yield through a Wittig reaction in the presence of ylide **51** and 2‐butynal, formed in situ by oxidation of 2‐butynol with MnO_2_. A series of cascade events, involving metal‐catalyzed cycloisomerization, proton demetallation, Friedel–Crafts acylation, and ring closure, was then successfully triggered between compound **53** and orcinol under catalysis with PtCl_2_ to give the corresponding intermediates **55** and **56** (1:1 as determined by ^1^H NMR), which contained requisite D, E, and F rings by putatively invoking the intermediacy of the metal‐benzopyrilium **54** species as an initiator.^17^ Without purification, the mixture was further deprotected through hydrogenolysis under catalysis with Pd/C to accomplish the desired target, in racemic form, in 65 % yield over 2 steps. The ^1^H NMR, ^13^C NMR, and mass spectral data obtained for the synthetic (±)‐ABX, whose structure is further unambiguously confirmed by an X‐ray crystallographic analysis,^18^ are in good agreement with those reported for the naturally occurring product.11c, 11d, 19

**Scheme 4:**
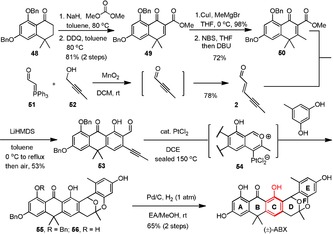
Application to the total synthesis of (±)‐ABX.

Vinylogous [4+2] anionic annulation can also be applied to other 1,2‐dipolar synthons such as the cyano or imine group to rapidly incorporate a pyridine‐2‐ol or pyridine‐2‐one unit in various alkaloid systems, historically, many of which might potentially possess a wide range of biological activities.^20^ As listed in Table 3, all cases examined turned out to be fruitless (0 % yield) when vinylogous donors were individually treated with 1,2‐dipolar acceptors under standard reaction conditions. However, as an extra ZnI_2_ (0.2 equiv) was added, the desired [4+2] annulation was activated, providing products **57**–**62** in moderate to good yields (51–86 %). As for the aldol‐type annulation, though the corresponding lactones (for eample, **63**) were allowed to form under standard conditions, they are quite labile and prone to undergo lactone‐ring cleavage to give thermodynamically more stable conjugated products, particularly when aryl aldehydes were used as acceptors as seen in acid products **64** (77 %) and **65** (84 %). Similar ring‐opening results had also been observed and reported by J. M. Charlton, et al.^21^ Collectively, it could be declared that the methodology described above is eligible to proceed in a regio‐ and chemocontrol manner, and should have great synthetic utility in terms of its operational simplicity and easy access to a variety of cross‐conjugated vinylogous donors.

**Table 3:** Scope of Mannich‐ and Aldol‐type [4+2] anionic annulation.

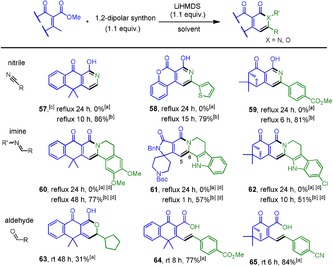

[a] The same as condition A in Table 2. [b] Lewis acid ZnI_2_ (0.2 equiv) was added. [c] 1, 3, 5‐triazine was used as an acceptor. [d] THF was used as solvent to increase the poor solubility of all imine acceptors.

In conclusion, the first total synthesis of (±)‐ABX has been successfully achieved by making use of a vinylogous Michael‐type annulation approach that is rather efficient in light of regiochemical control, a short synthetic sequence involved (7 steps), and the overall yield (about 20 %). In association with well‐documented benzylogous donors, the newly developed protocol with particular emphasis on creating vinylogous donors may augment synthetic capacity greatly to expand the structural diversity of a molecular library for screening various natural and/or non‐natural antibiotics, especially possessing a pharmacophore featured with an aromatic polyketide‐type scaffold, in both pharmaceutical and academic frontiers.

## Conflict of interest

The authors declare no conflict of interest.

## Supporting information

As a service to our authors and readers, this journal provides supporting information supplied by the authors. Such materials are peer reviewed and may be re‐organized for online delivery, but are not copy‐edited or typeset. Technical support issues arising from supporting information (other than missing files) should be addressed to the authors.

Supplementary
