# Supplementary material for: Development of a Cross‐Conjugated Vinylogous [4+2] Anionic Annulation and Application to the Total Synthesis of Natural Antibiotic (±)‐ABX
Source: Angew Chem Int Ed Engl. 2020 Feb 25;59(16):6540–5. doi: 10.1002/anie.201914657 (PMC7187479; doi:10.1002/anie.201914657)
Supplement: Supplementary file 1 — Supplementary [file ANIE-59-6540-s001.pdf]

Angewandte Chemie
Eine Zeitschrift der Gesellschaft Deutscher Chemiker

Supporting Information

Development of a Cross-Conjugated Vinylogous [4+2] Anionic Annulation and Application to the Total Synthesis of Natural Antibiotic (±)-ABX

*Jing-Kai Huang and Kak-Shan Shia**

anie_201914657_sm_miscellaneous_information.pdf

Table of Contents

Materials and Methods ………………………………………………….………..….S2

Synthetic procedures and characterization of various vinylogous donors …………………..S3

Synthetic procedures and characterization of new [4+2] annulation compounds ………….S16

Synthetic procedures and characterization of Michael acceptors …………………….…….S39

Synthetic procedures and characterization of (±)-ABX ……………………………………S42

Table S2. & S3. ^{1}H and ^{13}C NMR data of synthetic (±)-ABX and natural ABX …………..S44

References ……………………………………………………………………………..S46

^{1}H and ^{13}C NMR spectra for all new compounds ……………………………………..S47

Materials and Methods

All reactions were performed under nitrogen unless otherwise stated. All reagents were employed as received without further purification. All solvents were dried and distilled by standard techniques. Tetrahydrofuran was distilled from potassium under N_2. Dichloromethane and toluene were distilled from calcium hydride under N_2. Analytical thin layer chromatography was performed on SiO_2 60 F-254 plates and flash column chromatography was carried out using SiO_2 60 (particle size 0.040-0.055 mm, 230–400 mesh). Visualization was performed under UV irradiation at 254 nm followed by staining with aqueous potassium permanganate and charring by heat gun. Infrared spectra (IR) were recorded on a FT-IR spectrometer and expressed in cm^{-1}. ^{1}H and ^{13}C-NMR spectra were recorded by VNMR-300, VNMR-400 or VNMRS-700. Chemical shifts are expressed in ppm using TMS in $CDCl_3$ (δ = 0.00), residual methanol-d_4 (δ = 3.31), DMSO-d_6 (δ = 2.50) or Acetone-d_6 (δ = 2.05) as internal standard in ^{1}H-NMR spectra. ^{13}C-NMR spectra were recorded in either $CDCl_3$, methanol-d_4, DMSO-d_6 or Acetone-d_6, using the central resonances of $CDCl_3$ (δ = 77.00), methanol-d_4 (δ = 49.15), DMSO-d_6 (δ = 39.51) or Acetone-d_6 (δ = 29.92) as the internal references. Multiplicities are recorded as s (singlet), d (doublet), t (triplet), q (quartet), quint (quintet), dd (doublet of doublets), dt (doublet of triplets), tt (triplet of triplets), ddd (doublet of doublet of doublets), m (multiplet), br (broad). Coupling constants (*J*) are expressed in Hz. HRMS was obtained on a triple quadrupole mass analysis using electrospray ionization (ESI) source, and spectral data were recorded as m/z values. Melting points were measured using an Electrothermal instrument.

Synthetic procedures and characterization of various vinylogous donors

General procedure for preparation of 2-quinolone series:[1]

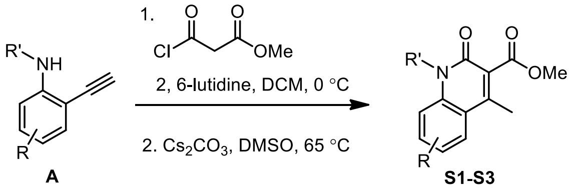

To a stirred solution of *o*-ethynylaniline **A** (10.0 mmol) and 2,6-lutidine (3.21 g, 30.0 mmol) in dry DCM (20 mL) at 0 °C was added methyl malonyl chloride (2.05 g, 15.0 mmol) dropwise under N_2. The mixture was then allowed to react at 0 °C for 30 min. under N_2. After the reaction was complete, sat. $NaHCO_{3(aq)}$ (20 mL) was added to quench the reaction. The organic layer was separated, and the aqueous layer was extracted with DCM (20 mL × 2). The organic portions were combined, washed with 5% $HCl_{(aq)}$ and sat. $NaHCO_{3(aq)}$, dried over $MgSO_4$, filtered and concentrated to give the amide intermediate. To a stirred solution of the amide intermediate in dry DMSO (10 mL) was added Cs_2CO_3 (3.58 g, 11.0 mmol) under N_2. The resulting mixture was then allowed to react at 65 °C for 15 h. After the reaction was complete, 5% $HCl_{(aq)}$ (10 mL) was added to quench the reaction at 0 °C, and the resulting mixture was extracted with EtOAc (20 mL × 2). The organic portions were combined, washed with sat. $NaHCO_{3(aq)}$ and brine, dried over $MgSO_4$, filtered and concentrated to give the crude residue, which was purified by chromatography on silical gel to afford the 2-quinolone donors **S1-S3**, individually.

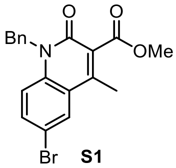

S1

Methyl 1-benzyl-6-bromo-4-methyl-2-oxo-1,2-dihydroquinoline-3-carboxylate (S1). Following the general procedure, 2-quinolone **S1** (2.70 g, 70% yield over 2 steps) was prepared from *N*-benzyl-4-bromo-2-ethynylaniline (2.86 g, 10.0 mmol) as a white solid: mp = 143–146 °C; IR (CH_2Cl_2 cast, cm^{-1}) ν_{max} 3062, 3031, 3002, 2951, 1736, 1645, 1590, 1560; ^1H NMR ($CDCl_3$, 400 MHz): δ 2.48 (s, 3H), 3.99 (s, 3H), 5.51 (br s, 2H), 7.17 (d, J = 9.2 Hz, 1H), 7.19-7.32 (m, 5H), 7.54 (dd, J = 9.2, 2.0 Hz, 1H), 7.88 (d, J = 2.0 Hz, 1H); ^{13}C NMR ($CDCl_3$, 100 MHz): δ 16.4, 46.0, 52.7, 115.6, 117.1, 122.1, 126.5, 127.2, 127.4, 128.3, 128.8, 134.1, 135.5, 137.6, 143.2, 158.8, 166.6; HRMS (ESI) m/z: $[M + Na]^+$ calcd. for $C_{19}H_{16}NO_3Br$ 408.0206, found 408.0216.

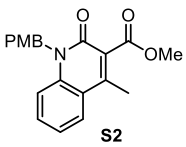

Methyl 1-(4-methoxybenzyl)-4-methyl-2-oxo-1,2-dihydroquinoline-3-carboxylate (S2). Following the general procedure, 2-quinolone **S2** (2.50 g, 74% yield over 2 steps) was prepared from 2-ethynyl-*N*-(4-methoxybenzyl)aniline (2.38 g, 10.0 mmol) as a cloroless oil; IR (CH_2Cl_2 cast, cm^{-1}) ν_{max} 2999, 2952, 2837, 1737, 1643, 1613, 1598; ^1H NMR ($CDCl_3$, 400 MHz): δ 2.50 (s, 3H), 3.76 (s, 3H), 3.99 (s, 3H), 5.48 (br s, 2H), 7.18 (d, J = 8.8 Hz, 2H), 7.18 (d, J = 8.8 Hz, 2H), 7.24 (t, J = 8.0 Hz, 1H), 7.35 (d, J = 8.4 Hz, 1H), 7.48 (td, J = 8.0, 1.2 Hz, 1H), 7.78 (dd, J = 8.4, 1.2 Hz, 1H); ^{13}C NMR ($CDCl_3$, 75 MHz): δ 16.4, 45.4, 52.6, 55.1, 114.1, 115.3, 120.4, 122.4, 125.8, 126.3, 128.0, 131.3, 138.7, 144.2, 158.7, 159.1, 167.1; HRMS (ESI) m/z: $[M + Na]^+$ calcd. for $C_{20}H_{19}NO_4Na$ 360.1206, found 360.1203.

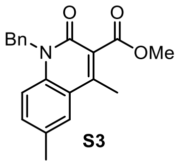

Methyl 1-benzyl-4,6-dimethyl-2-oxo-1,2-dihydroquinoline-3-carboxylate (S3). Following the general procedure, 2-quinolone **S3** (1.96 g, 61% yield over 2 steps) was prepared from *N*-benzyl-2-ethynyl-4-methylaniline (2.22 g, 10.0 mmol) as a white solid: mp = 188–190 °C; IR (CH_2Cl_2 cast, cm^{-1}) ν_{max} 3061, 3031, 2951, 2864, 1735, 1643, 1601, 1571; ^1H NMR ($CDCl_3$, 400 MHz): δ 2.40 (s, 3H), 2.49 (s, 3H), 3.99 (s, 3H), 5.53 (s, 2H), 7.18-7.30 (m, 7H), 7.55 (d, J = 1.2 Hz, 1H); ^{13}C NMR ($CDCl_3$, 100 MHz): δ 16.3, 20.6, 45.8, 52.5, 115.2, 120.3, 125.6, 126.1, 126.6, 127.1, 128.6, 131.9, 132.5, 136.1, 136.6, 144.0, 159.0, 167.2; HRMS (ESI) m/z: $[M +Na]^+$ calcd. for $C_{20}H_{19}NO_3Na$ 344.1257, found 344.1262.

General procedure for preparation of the coumarin series:[2]

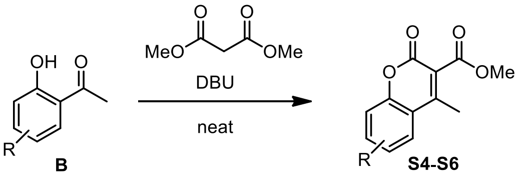

To a stirred solution of *o*-hydroxyacetophenone **B** (5.0 mmol) in DBU (3 mL) at room temperature was added dimethyl malonate (925 mg, 7.0 mmol) dropwise under N_2. The mixture was then allowed to react at 100 °C for 4 h under N_2. After the reaction was complete, 5% $HCl_{(aq)}$ (5 mL) was added to quench the reaction at 0 °C, and the resulting mixture was extracted with EtOAc (10 mL × 2). The organic portions were combined, washed with sat. $NaHCO_{3(aq)}$ and brine, dried over $MgSO_4$, filtered and concentrated to give the crude residue,

which was purified by chromatography on silical gel to afford the coumarin donors **S4-S6**, individually.

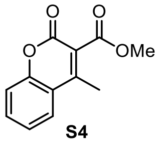

S4

Methyl 4-methyl-2-oxo-2*H*-chromene-3-carboxylate (S4). Following the general procedure, coumarin **S4** (906 mg, 83% yield) was prepared from 2-hydroxyacetophenone (681 mg, 5.0 mmol) as a white solid: mp = 80–82 °C; IR (CH_2Cl_2 cast, cm^{-1}) ν_{max} 3003, 2954, 1735, 1719, 1618, 1605, 1570; ^1H NMR ($CDCl_3$, 300 MHz): δ 2.49 (s, 3H), 3.97 (s, 3H), 7.35 (td, J = 8.1, 1.2 Hz, 1H), 7.36 (dd, J = 8.1, 1.2 Hz, 1H), 7.60 (td, J = 8.1, 1.2 Hz, 1H), 7.70 (dd, J = 8.1, 1.2 Hz, 1H); ^{13}C NMR ($CDCl_3$, 75 MHz): δ 16.0, 52.7, 116.9, 118.8, 120.7, 124.6, 125.3, 132.7, 150.6, 152.6, 157.6, 165.1; HRMS (ESI) m/z: $[M + H]^+$ calcd. for $C_{12}H_{11}O_4$ 219.0652, found 219.0650.

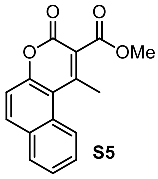

S5

Methyl 1-methyl-3-oxo-3*H*-benzo[*f*]chromene-2-carboxylate (S5). Following the general procedure, coumarin **S5** (1.02 g, 76% yield) was prepared from 1-acetyl-2-naphthol (931 mg, 5.0 mmol) as a white solid: mp = 156–158 °C; IR (CH_2Cl_2 cast, cm^{-1}) ν_{max} 3077, 3028, 2959, 2847, 1735, 1710, 1605, 1554; ^1H NMR ($CDCl_3$, 400 MHz): δ 2.88 (s, 3H), 4.00 (s, 3H), 7.45 (d, J = 8.8 Hz, 1H), 7.58 (td, J = 8.0, 1.2 Hz, 1H), 7.67 (td, J = 9.2, 1.2 Hz, 1H), 7.94 (dd, J = 8.0, 1.2 Hz, 1H), 8.02 (d, J = 9.2 Hz, 1H), 8.49 (d, J = 8.8 Hz, 1H); ^{13}C NMR ($CDCl_3$, 75 MHz): δ 22.9, 52.8, 113.6, 117.0, 121.5, 124.9, 125.6, 127.9, 129.7, 131.2, 134.6, 151.8, 153.7, 157.2, 165.7; HRMS (ESI) m/z: $[M + H]^+$ calcd. for $C_{16}H_{13}O_4$ 269.0808, found 269.0813

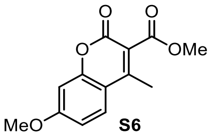

S6

Methyl 7-methoxy-4-methyl-2-oxo-2*H*-chromene-3-carboxylate (S6). Following the general procedure, coumarin **S6** (820 mg, 66% yield) was prepared from 2-hydroxy-4-methoxyacetophenone (831 mg, 5.0 mmol) as a white solid: mp = 131–132 °C; IR (CH_2Cl_2 cast, cm^{-1}) ν_{max} 3080, 2953, 2845, 1718, 1609, 1560; ^1H NMR ($CDCl_3$, 400 MHz): δ 2.46 (s, 3H), 3.89 (s, 3H), 3.95 (s, 3H), 6.82 (d, J = 2.4 Hz, 1H), 6.90 (dd, J = 9.2, 2.4 Hz,

1H), 7.59 (d, J = 9.2 Hz, 1H); ^{13}C NMR ($CDCl_3$, 100 MHz): δ 16.1, 52.6, 55.7, 100.6, 112.4, 122.9, 117.5, 126.5, 151.3, 154.7, 158.1, 163.5, 165.5; HRMS (ESI) m/z: $[M + Na]^+$ calcd. for $C_{13}H_{12}O_5Na$ 271.0577, found 271.0581.

General procedure for preparation of the γ-lactam series:[3]

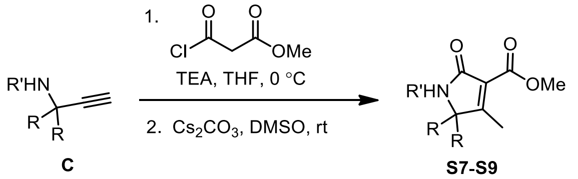

To a stirred solution of 1-ethynylamine **C** (5.0 mmol) and TEA (1.01 g, 10.0 mmol) in dry THF (20 mL) at 0 °C was added methyl malonyl chloride (819 mg, 6.0 mmol) dropwise under N_2. The mixture was then allowed to react at 0 °C for 30 min under N_2. After the reaction was complete, sat. $NaHCO_{3(aq)}$ (20 mL) was added to quench the reaction. The layers were separated, and the aqueous layer was extracted with EtOAc (10 mL × 2). The organic portions were combined, washed with 5% $HCl_{(aq)}$, sat. $NaHCO_{3(aq)}$ and brine, dried over $MgSO_4$, filtered and concentrated to give the mixture of product and amide intermediate. To a stirred solution of the mixture in dry DMSO (5 mL) was added Cs_2CO_3 (1.95 g, 6.0 mmol) under N_2. The resulting mixture was then allowed to react at room temperature for 1 h. After the reaction was complete, 5% $HCl_{(aq)}$ (5.0 mL) was added to quench the reaction at 0 °C, and the resulting mixture was extracted with EtOAc (10 mL × 2). The organic portions were combined, washed with sat. $NaHCO_{3(aq)}$ and brine, dried over $MgSO_4$, filtered and concentrated to give the crude residue, which was purified by chromatography on silical gel to afford γ-lactam donors **S7-S9**, individually.

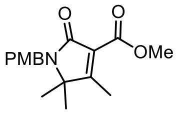

S7

Methyl 1-(4-methoxybenzyl)-4,5,5-trimethyl-2-oxo-2,5-dihydro-1H-pyrrole-3-carboxylate (S7). Following the general procedure, γ-lactam **S7** (1.35 g, 89% yield over 2 steps) was prepared from *N*-(4-methoxybenzyl)-2-methylbut-3-yn-2-amine (1.02 g, 5.0 mmol) as a colorless oil; IR (CH_2Cl_2 cast, cm^{-1}) ν_{max} 2977, 2951, 2838, 1741, 1711, 1686, 1613, 1513; ^{1}H NMR ($CDCl_3$, 300 MHz): δ 1.17 (s, 6H), 2.25 (s, 3H), 3.78 (s, 3H), 3.89 (s, 3H), 4.55 (s, 2H), 6.81 (d, J = 8.8 Hz, 2H), 7.26 (d, J = 8.8 Hz, 2H); ^{13}C NMR ($CDCl_3$, 75 MHz): δ 12.1, 23.3, 42.0, 51.7, 55.1, 64.9, 113.6, 122.5, 129.0, 130.5, 158.6, 163.6, 165.9, 172.8; HRMS (ESI) m/z: $[M + H]^+$ calcd. for $C_{17}H_{22}NO_4$ 304.1543, found 304.1555.

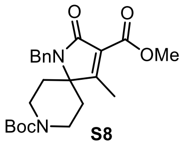

S8

8-*tert*-Butyl 3-methyl 1-benzyl-4-methyl-2-oxo-1,8-diazaspiro[4.5]dec-3-ene-3,8-dicarboxylate (S8). Following the general procedure, γ-lactam **S8** (1.97 g, 95% yield over 2 steps) was prepared from *tert*-butyl 4-(benzylamino)-4-ethynylpiperidine-1-carboxylate (1.57 g, 5.0 mmol) as a white solid: mp = 102–103 °C; IR (CH_2Cl_2 cast, cm^{-1}) ν_{max} 2973, 2930, 1744, 1690, ; ^1H NMR ($CDCl_3$, 400 MHz): δ 1.46 (s, 9H), 1.77 (br s, 4H), 2.39 (s, 3H), 3.53 (br s, 4H), 3.91 (s, 3H), 4.75 (br s, 2H), 7.22-7.32 (m, 5H); ^{13}C NMR ($CDCl_3$, 100 MHz): δ 14.1, 27.9, 30.3, 39.4, 43.2, 51.5, 64.4, 79.5, 123.3, 126.5, 126.7, 128.1, 137.5, 154.1, 163.0, 165.9, 170.9; HRMS (ESI) m/z: $[M +Na]^+$ calcd. for $C_{23}H_{30}N_2O_5Na$ 437.2047, found 437.2049.

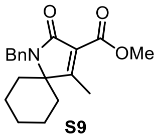

S9

Methyl 1-benzyl-4-methyl-2-oxo-1-azaspiro[4.5]dec-3-ene-3-carboxylate (S9). Following the general procedure, γ-lactam **S9** (1.44 g, 92% yield over 2 steps) was prepared from *N*-benzyl-1-ethynylcyclohexanamine (1.07 g, 5.0 mmol) as a white solid: mp = 117–119 °C; IR (CH_2Cl_2 cast, cm^{-1}) ν_{max} 3061, 3030, 2946, 2872, 2854, 1742, 1712, 1690; ^1H NMR ($CDCl_3$, 400 MHz): δ 1.43-1.67 (m, 10H), 2.43 (s, 3H), 3.89 (s, 3H), 4.75 (s, 2H), 7.19-7.30 (m, 5H); ^{13}C NMR ($CDCl_3$, 100 MHz): δ 15.5, 22.1, 23.9, 32.0, 43.6, 51.9, 66.8, 123.3, 126.8, 126.8, 128.3, 138.4, 163.9, 166.5, 173.3; HRMS (ESI) m/z: $[M + H]^+$ calcd. for $C_{19}H_{24}NO_3$ 314.1751, found 314.1742.

General procedure for preparation of verbenone derivative:

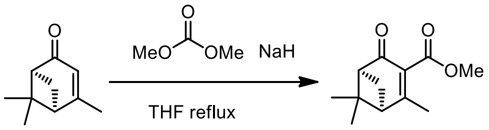

(1*R*,5*S*)-Methyl 2,6,6-trimethyl-4-oxobicyclo[3.1.1]hept-2-ene-3-carboxylate (S10).[4] To a stirred solution of (-)-verbenone (15.02 g, 100.0 mmol) and dimethyl carbonate (13.51 g, 150.0 mmol) in dry THF (500 mL) at room temperature was added 60% NaH (6.00 g, 150.0 mmol) portionwise under N_2. The mixture was then heated up to reflux for 15 h under N_2. After the reaction was complete, 5% $HCl_{(aq)}$ (100 mL) was added to quench the reaction at 0 °C, and the resulting mixture was extracted with EtOAc (400 mL × 2). The organic portions

were combined, washed with sat. $NaHCO_{3(aq)}$ and brine, dried over $MgSO_4$, filtered and concentrated to give the crude residue, which was purified by chromatography on silical gel to afford donor **S10** (17.07 g, 82% yield) as a yellow oil; IR (CH_2Cl_2 cast, cm^{-1}) ν_{max} 2953, 2873, 1735, 1684, 1624; ^{1}H NMR ($CDCl_3$, 300 MHz): δ 1.05 (s, 3H), 1.50 (s, 3H), 2.14 (d, J = 12.8 Hz, 1H), 2.18 (s, 3H), 2.54 (t, J = 7.2 Hz, 1H), 2.75 (t, J = 7.2 Hz, 1H), 2.81 (dt, J = 12.8, 7.2 Hz, 1H), 3.84 (s, 3H); ^{13}C NMR ($CDCl_3$, 75 MHz): δ 21.6, 22.1, 26.4, 39.0, 50.7, 51.8, 53.4, 57.2, 126.1, 165.7, 171.9, 198.4; HRMS (ESI) m/z: [M + Na]$^+$ calcd. for $C_{12}H_{16}O_3Na$ 231.0992, found 231.0985.

General procedure for the γ-lactone series:[5]

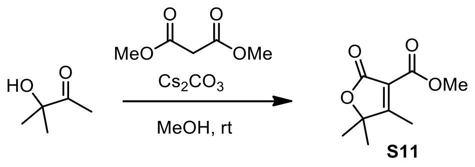

Methyl 4,5,5-trimethyl-2-oxo-2,5-dihydrofuran-3-carboxylate (S11).[5b] To a stirred solution of 3-Hydroxy-3-methyl-2-butanone (1.02 g, 10.0 mmol) and dimethyl malonate (3.96 g, 30.0 mmol) in dry MeOH (50 mL) at room temperature was added Cs_2CO_3 (8.15 g, 25.0 mmol) portionwise under N_2. The mixture was then allowed to react at room temperature for 5 h under N_2. After the reaction was complete, 5% $HCl_{(aq)}$ (10 mL) was added to quench the reaction at 0 °C, and most of solvent was evaporated under reduced pressure. The resulting aqueous solution was extracted with EtOAc (20 mL x 2). The organic portions were combined, washed with sat. $NaHCO_{3(aq)}$ and brine, dried over $MgSO_4$, filtered and concentrated to give the crude residue, which was purified by chromatography on silical gel to afford γ-lactone donor **S11** (976 mg, 53% yield) as a white solid: mp = 64–65 °C; IR (CH_2Cl_2 cast, cm^{-1}) ν_{max} 2992, 2958, 2850, 1763, 1712, 1635; ^{1}H NMR ($CDCl_3$, 300 MHz): δ 1.49 (s, 6H), 2.36 (s, 3H), 3.89 (s, 3H); ^{13}C NMR ($CDCl_3$, 75 MHz): δ 13.1, 24.3, 52.1, 85.4, 117.9, 162.1, 167.2, 181.2; HRMS (ESI) m/z: [M + Na]$^+$ calcd. for $C_9H_{12}O_4Na$ 207.0628, found 207.0615.

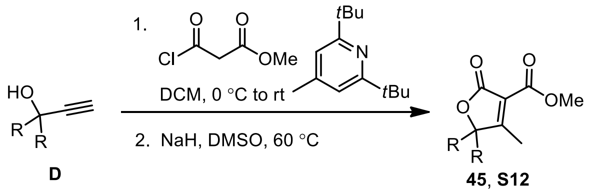

To a stirred solution of alcohol **D** (10.0 mmol) and 2,6-di-*tert*-butyl-4-methylpyridine (2.26 g, 11.0 mmol) in dry DCM (40 mL) at 0 °C was added methyl malonyl chloride (1.50 g, 11.0 mmol) dropwise under N_2. The mixture was then allowed to react at room temperature for 1 h under N_2. After the reaction was complete, sat. $NaHCO_{3(aq)}$ (20 mL) was added to quench the reaction. The layers were separated, and the aqueous layer was extracted with DCM (20 mL).

The organic portions were combined, washed with 5% $HCl_{(aq)}$ and sat. $NaHCO_{3(aq)}$, dried over $MgSO_4$, filtered and concentrated to give the mixture of desired product and ester intermediate. Without purification, to a stirred solution of the mixture in dry DMSO (20 mL) was added 60% NaH (600 mg, 15.0 mmol) portionwise under N_2. The resulting mixture was then allowed to react at 60 °C for 10 h. After the reaction was complete, 5% $HCl_{(aq)}$ (10 mL) was added to quench the reaction at 0 °C; the aqueous layer was separated and extracted with EtOAc (20 mL × 2). The organic portions were combined, washed with sat. $NaHCO_{3(aq)}$ and brine, dried over $MgSO_4$, filtered and concentrated to give the crude residue, which was purified by chromatography on silical gel to afford γ-lactone donors **45** and **S12**, individually.

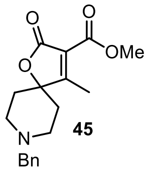

45

Methyl 8-benzyl-4-methyl-2-oxo-1-oxa-8-azaspiro[4.5]dec-3-ene-3-carboxylate (45). Following the general procedure, γ-lactone **45** (1.89 g, 60% yield over 2 steps) was prepared from 1-benzyl-4-ethynylpiperidin-4-ol (2.15 g, 10.0 mmol) as a colorless oil; IR (CH_2Cl_2 cast, cm^{-1}) ν_{max} 3061, 3028, 2951, 2925, 2815, 2771, 1772, 1719, 1653; ^1H NMR ($CDCl_3$, 400 MHz): δ 1.47 (dd, J = 12.8, 2.4 Hz, 2H), 2.09 (td, J = 12.8, 4.8 Hz, 2H), 2.36 (s, 3H), 2.47 (td, J = 11.6, 2.4 Hz, 2H), 2.88 (ddd, J = 11.6, 4.8, 2.4 Hz, 2H), 3.57 (s, 2H), 3.88 (s, 3H), 7.26-7.33 (m, 5H); ^{13}C NMR ($CDCl_3$, 100 MHz): δ 13.3, 32.8, 48.7, 52.1, 62.8, 85.3, 118.5, 127.1, 128.2, 129.0, 137.7, 162.0, 167.0, 180.4; HRMS (ESI) m/z: $[M + Na]^+$ calcd. for $C_{18}H_{21}NO_4Na$ 338.1363, found 338.1366.

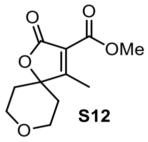

S12

Methyl 4-methyl-2-oxo-1,8-dioxaspiro[4.5]dec-3-ene-3-carboxylate (S12). Following the general procedure, γ-lactone **S12** (1.47 g, 65% yield over 2 steps) was prepared from 4-ethynyltetrahydro-2*H*-pyran-4-ol (1.26 g, 10.0 mmol) as a white solid: mp = 143–145 °C; IR (CH_2Cl_2 cast, cm^{-1}) ν_{max} 2987, 2958, 2923, 2873, 1771, 1717, 1680, 1654; ^1H NMR ($CDCl_3$, 400 MHz): δ 1.41 (d, J = 12.4 Hz, H), 2.12 (td, J = 12.4, 4.8 Hz, 2H), 2.37 (s, 3H), 3.86 (td, J = 12.0, 2.0 Hz, 2H), 3.90 (s, 3H), 4.00 (dd, J = 12.0, 4.8 Hz, 2H); ^{13}C NMR ($CDCl_3$, 100 MHz): δ 13.2, 32.5, 52.0, 63.2, 84.3, 118.7, 161.8, 166.6, 179.0; HRMS (ESI) m/z: $[M + Na]^+$ calcd. for $C_{11}H_{14}O_5Na$ 249.0733, found 249.0739.

General procedure for preparation of the 1-tetralone series:

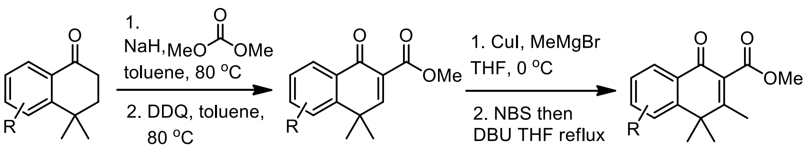

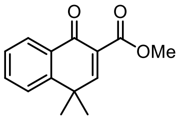

7

Methyl 4,4-dimethyl-1-oxo-1,4-dihydronaphthalene-2-carboxylate (7). To a stirred solution of 4,4-dimethyl-3,4-dihydronaphthalen-1(2*H*)-one (8.71 g, 50.0 mmol) and dimethyl carbonate (6.76 g, 75.0 mmol) in dry toluene (150 mL) at room temperature was added 60% NaH (2.40 g, 60.0 mmol) portionwise under N_2. The mixture was then allowed to react at 80 °C for 4 h under N_2. After the reaction was complete, 5% $HCl_{(aq)}$ (100 mL) was added to quench the reaction at 0 °C, and the resulting mixture was extracted with EtOAc (50 mL × 2). The organic portions were combined, washed with sat. $NaHCO_{3(aq)}$ and brine, dried over $MgSO_4$, filtered and concentrated to give the 1,3-ketone ester intermediate. To a stirred solution of the 1,3-ketone ester intermediate in dry toluene (150 mL) was added DDQ (11.35 g, 50.0 mmol) in one portion. The resulting mixture was then stirred at 80 °C under N_2 for 2 h. After reaction was complete, the reaction mixture was filtrated with celite, and quenched with saturated $NaHCO_{3(aq)}$ (50 mL) and extracted with EtOAc (50 mL × 2). The combined organic extract was washed with saturated $NaHCO_{3(aq)}$ and brine, dried over $MgSO_4$, filtered and concentrated to give the crude residue, which was purified by chromatography on silical gel to afford enone **7** (10.25 g, 89% yield over 2 steps) as a colorless oil; IR (CH_2Cl_2 cast, cm^{-1}) ν_{max} 3065, 3031, 2970, 2930, 2869, 1745, 1663, 1603; ^1H NMR ($CDCl_3$, 400 MHz): δ 1.55 (s, 6H), 3.91 (s, 3H), 7.42 (td, *J* = 7.6, 1.2 Hz, 1H), 7.53 (dd, *J* = 7.6, 0.8 Hz, 1H), 7.61 (td, *J* = 8.0, 0.8 Hz, 1H), 7.63 (s, 1H), 8.22 (dd, *J* = 8.0, 1.2 Hz, 1H); ^{13}C NMR ($CDCl_3$, 100 MHz): δ 29.0, 37.3, 52.1, 125.8, 126.9, 127.1, 129.1, 130.3, 132.8, 148.0, 161.9, 165.3, 180.5; HRMS (ESI) m/z: $[M + H]^+$ calcd. for $C_{14}H_{15}O_3$ 231.1016, found 231.1030.

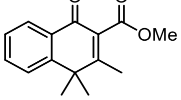

5

Methyl 3,4,4-trimethyl-1-oxo-1,4-dihydronaphthalene-2-carboxylate (5). To a stirred solution of CuI (8.57 g, 45.0 mmol) in dry THF (200 mL) at 0 °C was added MeMgBr solution (3.0 M in Et_2O, 14.0 mmol) dropwise under N_2. The mixture was then allowed to react at 0 °C for 30 min under N_2. To the stirred mixture at 0 °C was then added the enone **7** solution (9.21 g in 20 mL THF, 40.0 mmol) dropwise under N_2. The resulting solution was

allowed to react at 0 °C for 30 min under N_2. After the reaction was complete, sat. $NH_4Cl_{(aq)}$ (50 mL) was added to quench the reaction at 0 °C, and the resulting mixture was diluted with EtOAc (200 mL) and filtrated with celite. The organic layer was separated, and the aqueous layer was extracted with EtOAc (50 mL). The organic portions were combined, washed with water and brine, dried over $MgSO_4$, filtered and concentrated to give the *β*-methyl-1,3-ketone ester intermediate. To a stirred solution of the *β*-methyl-1,3-ketone ester intermediate in THF (200 mL) was added NBS (7.83 g, 50.0 mmol) in one portion at room temperature. After the resulting mixture was stirred at room temperature for 1 h, DBU (15.22 g, 100.0 mmol) was added, and the resulting mixture was then heated to reflux under N_2 for 24 h. The reaction mixture was quenched with saturated $NaHCO_{3(aq)}$ (50 mL) and extracted with EtOAc (200 mL × 2). The combined organic extract was washed with saturated $NaHCO_{3(aq)}$ and brine, dried over $MgSO_4$, filtered and concentrated to give the crude residue, which was purified by chromatography on silical gel to afford enone **5** (4.45 g, 46%) and recovered *β*-methyl-1,3-ketone ester (4.79 g, 49%). The recovered intermediate was used to reproduce enone **5** by reapting the bromination/*β*-elimation sequence. Over two repeated cycles, the desired target enone donor **5** (7.43 g, 76% yield over 2 steps) was affored as a white solid: mp = 97–99 °C; IR (CH_2Cl_2 cast, cm^{-1}) ν_{max} 3066, 3032, 2981, 2952, 2874, 1738, 1652, 1603; ^{1}H NMR ($CDCl_3$, 400 MHz): δ 1.55 (s, 6H), 2.13 (s, 3H), 3.91 (s, 3H), 7.37-7.41 (m, 1H), 7.58-7.63 (m, 2H), 8.18 (dd, J = 8.8, 0.4 Hz, 1H); ^{13}C NMR ($CDCl_3$, 100 MHz): δ 17.4, 28.3, 40.3, 52.3, 126.3, 126.6, 126.7, 129.2, 132.6, 132.9, 150.1, 161.7, 167.8, 180.7; HRMS (ESI) m/z: $[M + H]^+$ calcd. for $C_{15}H_{17}O_3$ 245.1172, found 245.1169.

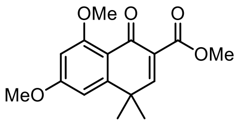

S13

Methyl 6,8-dimethoxy-4,4-dimethyl-1-oxo-1,4-dihydronaphthalene-2-carboxylate (S13). According to the synthetic procedures similar to compound **7**, enone **S13** (5.34 g, 92% yield over 2 steps) was prepared from 6,8-dimethoxy-4,4-dimethyl-3,4-dihydronaphthalen-1(2*H*)-one (4.69 g, 20.0 mmol) as a white solid: mp = 171–172 °C; IR (CH_2Cl_2 cast, cm^{-1}) ν_{max} 2968, 2843, 1739, 1667, 1643, 1598; ^{1}H NMR ($CDCl_3$, 400 MHz): δ 1.49 (s, 6H), 3.87 (s, 3H), 3.89 (s, 3H), 3.92 (s, 3H), 6.42 (d, J = 2.4 Hz, 1H), 6.54 (d, J = 2.4 Hz, 1H), 7.31 (s, 1H); ^{13}C NMR ($CDCl_3$, 100 MHz): δ 29.9, 37.5, 52.1, 55.2, 55.9, 96.9, 102.7, 114.4, 131.4, 153.0, 156.4, 162.4, 163.6, 166.1, 179.5; HRMS (ESI) m/z: $[M + Na]^+$ calcd. for $C_{16}H_{18}O_5Na$ 313.1046, found 313.1061.

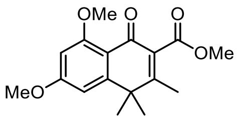

S14

Methyl 6,8-dimethoxy-3,4,4-trimethyl-1-oxo-1,4-dihydronaphthalene-2-carboxylate (S15). According to the synthetic procedures similar to compound **5**, enone donor **S14** (2.04 g, 67% yield over 2 steps) was prepared from enone **S13** (2.90 g, 10.0 mmol) as a white solid: mp = 144–146 °C; IR (CH_2Cl_2 cast, cm^{-1}) ν_{max} 2978, 2949, 2842, 1733, 1655, 1638, 1598; ^{1}H NMR ($CDCl_3$, 400 MHz): δ 1.50 (s, 6H), 2.06 (s, 3H), 3.87 (s, 3H), 3.90 (s, 3H), 3.91 (s, 3H), 6.42 (d, *J* = 2.4 Hz, 1H), 6.64 (d, *J* = 2.4 Hz, 1H); ^{13}C NMR ($CDCl_3$, 100 MHz): δ 16.8, 29.0, 40.7, 52.0, 55.3, 56.0, 96.6, 103.4, 113.2, 134.1, 155.2, 156.4, 162.5, 163.6, 168.2, 179.5; HRMS (ESI) m/z: $[M + H]^+$ calcd. for $C_{17}H_{21}O_5$ 305.1384, found 305.1389.

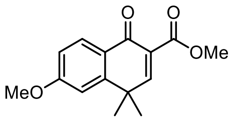

S15

Methyl 6-methoxy-4,4-dimethyl-1-oxo-1,4-dihydronaphthalene-2-carboxylate (S15). According to the synthetic procedures similar to compound **7**, enone **S15** (4.53 g, 87% yield over 2 steps) was prepared from 6-methoxy-4,4-dimethyl-3,4-dihydronaphthalen-1(2*H*)-one (4.08 g, 20.0 mmol) as a white solid: mp = 103–105 °C; IR (CH_2Cl_2 cast, cm^{-1}) ν_{max} 2970, 2842, 1740, 1656, 1600, 1569; ^{1}H NMR ($CDCl_3$, 400 MHz): δ 1.52 (s, 6H), 3.90 (s, 3H), 3.91 (s, 3H), 6.94 (d, *J* = 2.8 Hz, 1H), 6.94 (dd, *J* = 8.0, 2.8 Hz, 1H), 7.54 (s, 1H), 8.20 (d, *J* = 8.0 Hz, 1H); ^{13}C NMR ($CDCl_3$, 100 MHz): δ 29.3, 37.4, 52.2, 55.3, 110.6, 113.1, 124.1, 129.3, 129.8, 150.4, 160.9, 163.2, 165.6, 179.7; HRMS (ESI) m/z: $[M + Na]^+$ calcd. for $C_{15}H_{16}O_4Na$ 283.0941, found 283.0945.

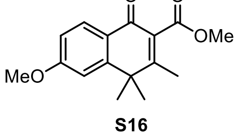

S16

Methyl 6-methoxy-3,4,4-trimethyl-1-oxo-1,4-dihydronaphthalene-2-carboxylate (S16). According to the synthetic procedures similar to compound **5**, enone donor **S16** (2.03 g, 74% yield over 2 steps) was prepared from enone **S15** (2.60 g, 10.0 mmol) as a white solid: mp = 118–119 °C; IR (CH_2Cl_2 cast, cm^{-1}) ν_{max} 2981, 2949, 2842, 1735, 1649, 1601; ^{1}H NMR ($CDCl_3$, 400 MHz): δ 1.53 (s, 6H), 2.12 (s, 3H), 3.90 (s, 3H), 3.91 (s, 3H), 6.94 (dd, *J* = 8.8, 2.4 Hz, 1H), 7.01 (d, *J* = 2.4 Hz, 1H), 8.16 (d, *J* = 8.8 Hz, 1H); ^{13}C NMR ($CDCl_3$, 100 MHz):

δ 17.3, 28.4, 40.3, 52.1, 55.3, 111.2, 112.8, 122.9, 129.0, 132.5, 152.5, 160.6, 163.3, 167.9, 179.8; HRMS (ESI) m/z: $[M + Na]^+$ calcd. for $C_{16}H_{18}O_4Na$ 297.1097, found 297.1105.

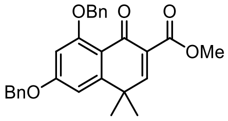

49

Methyl 6,8-bis(benzyloxy)-4,4-dimethyl-1-oxo-1,4-dihydronaphthalene-2-carboxylate (46). According to the synthetic procedures similar to compound **7**, enone **49** (7.17 g, 81% yield over 2 steps) was prepared from 6,8-bis(benzyloxy)-4,4-dimethyl-3,4-dihydronaphthalen-1(2*H*)-one (**48**) (7.73 g, 20.0 mmol) as a white solid: mp = 130–132 °C; IR (CH_2Cl_2 cast, cm^{-1}) ν_{max} 3063, 3032, 2966, 2950, 2930, 2876, 1736, 1669, 1642, 1598; ^1H NMR ($CDCl_3$, 400 MHz): δ 1.47 (s, 6H), 3.87 (s, 3H), 5.07 (s, 2H), 5.20 (s, 2H), 6.53 (d, J = 2.4 Hz, 1H), 6.62 (d, J = 2.4 Hz, 1H), 7.31 (s, 1H), 7.30-7.41 (m, 8H), 7.57 (d, J = 6.8 Hz, 2H); ^{13}C NMR ($CDCl_3$, 100 MHz): δ 30.1, 37.6, 52.2, 70.1, 70.6, 99.5, 104.4, 115.1, 126.7, 127.5, 128.3, 128.6, 131.5, 135.8, 136.5, 153.0, 156.5, 161.4, 162.5, 166.1, 179.3; HRMS (ESI) m/z: $[M + H]^+$ calcd. for $C_{28}H_{27}O_5$ 443.1853, found 443.1850.

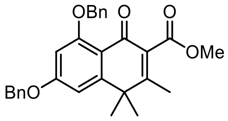

50

Methyl 6,8-bis(benzyloxy)-3,4,4-trimethyl-1-oxo-1,4-dihydronaphthalene-2-carboxylate (50). According to the synthetic procedures similar to compound **5**, enone donor **50** (3.29 g, 72% yield over 2 steps) was prepared from enone **49** (4.43 g, 10.0 mmol) as a white solid: mp = 141–143 °C; IR (CH_2Cl_2 cast, cm^{-1}) ν_{max} 3063, 3032, 2981, 2950, 1733, 1655, 1638, 1599; ^1H NMR ($CDCl_3$, 400 MHz): δ 1.48 (s, 6H), 2.05 (s, 3H), 3.89 (s, 3H), 5.06 (s, 2H), 5.20 (s, 2H), 6.51 (d, J = 2.4 Hz, 1H), 6.71 (d, J = 2.4 Hz, 1H), 7.28-7.41 (m, 8H), 7.55 (d, J = 8.0 Hz, 2H); ^{13}C NMR ($CDCl_3$, 100 MHz): δ 16.9, 29.0, 40.6, 52.1, 70.1, 70.6, 99.3, 105.0, 113.8, 126.7, 127.5, 127.6, 128.3, 128.5, 128.7, 134.2, 135.9, 136.6, 155.1, 156.3, 161.2, 162.5, 168.4, 179.2; HRMS (ESI) m/z: $[M + H]^+$ calcd. for $C_{29}H_{29}O_5$ 457.2010, found 457.2006.

General procedure for preparation of the chromone series:[6]

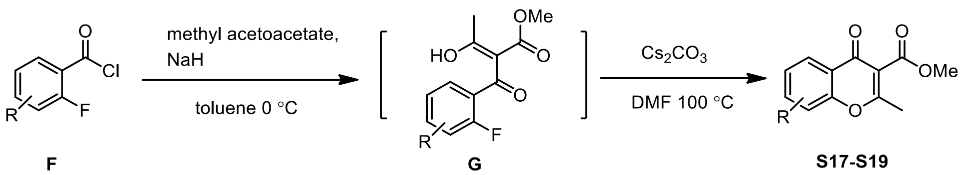

To a stirred solution of methyl acetoacetate (1.28 g, 11.0 mmol) in dry toluene (50 mL) at 0 °C was added 60% NaH (440 mg, 11.0 mmol) portionwise under N_2. The mixture was then allowed to react at 0 °C for 30 min under N_2. To this stirred mixture at 0 °C was then added the acid chloride **F** solution (in 25 mL toluene, 10.0 mmol) dropwise under N_2, and allowed to react at 0 °C for 30 min under N_2. After the reaction was complete, water (25 mL) was added to quench the reaction at 0 °C, and the resulting mixture was extracted with EtOAc (25 mL × 2). The organic portions were combined, washed with sat. $NaHCO_{3(aq)}$ and brine, dried over $MgSO_4$, filtered and concentrated to give the intermediate **G**. To a stirred solution of the intermediate **G** in dry DMF (10 mL) was added Cs_2CO_3 (4.89 g, 15.0 mmol) in one portion at room temperature. The mixture was then allowed to react at 100 °C for 1 h under N_2. After the reaction was complete, water (25 mL) was added to quench the reaction, and the resulting mixture was extracted with EtOAc (25 mL × 2). The organic portions were combined, washed with water (10 mL × 3) and brine, dried over $MgSO_4$, filtered and concentrated to give the crude residue, which was purified by chromatography on silical gel to afford chromone donors **S17-S19**, individually.

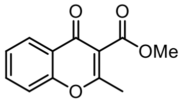

S17

Methyl 2-methyl-4-oxo-4*H*-chromene-3-carboxylate (S17).[6] Following the general procedure, chromone **S17** (1.72 g, 79% yield over 2 steps) was prepared from 2-fluorobenzoyl chloride (1.59 g, 10.0 mmol) as a white solid: mp = 120–121 °C; IR (CH_2Cl_2 cast, cm^{-1}) ν_{max} 3104, 3051, 3017, 2956, 2925, 1734, 1646, 1618, 1577; ^{1}H NMR ($CDCl_3$, 300 MHz): δ 2.53 (s, 3H), 3.94 (s, 3H), 7.41 (td, J = 8.4, 1.2 Hz, 1H), 7.43 (d, J = 8.4 Hz, 1H), 7.67 (td, J = 7.8, 1.8 Hz, 1H), 8.21 (dd, J = 7.8, 1.8 Hz, 1H); ^{13}C NMR ($CDCl_3$, 75 MHz): δ 19.4, 52.4, 117.5, 123.0, 125.3, 125.8, 133.8, 155.2, 165.4, 167.1, 174.1; HRMS (ESI) m/z: $[M + Na]^+$ calcd. for $C_{12}H_{10}O_4Na$ 241.0471, found 241.0472.

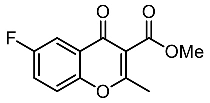

S18

Methyl 6-fluoro-2-methyl-4-oxo-4*H*-chromene-3-carboxylate (S18). Following the general procedure, chromone **S18** (1.58 g, 67% yield over 2 steps) was prepared from 2,5-difluorobenzoyl chloride (1.77 g, 10.0 mmol) as a white solid: mp = 130–133 °C; IR (CH_2Cl_2 cast, cm^{-1}) ν_{max} 3062, 2964, 1733, 1639, 1586; ^{1}H NMR ($CDCl_3$, 400 MHz): δ 2.54 (s, 3H), 3.95 (s, 3H), 7.37-7.46 (m, 2H), 7.84 (dd, J = 8.4, 3.2 Hz, 1H); ^{13}C NMR ($CDCl_3$, 100 MHz): δ 19.5, 52.6, 110.8 (d, J_{C-F} = 23.7 Hz), 117.0, 119.9 (d, J_{C-F} = 8.4 Hz), 122.1 (d, J_{C-F} =

25.1 Hz), 124.4 (d, $J_{C\text{-}F}$ = 7.6 Hz), 151.6, 159.6 (d, $J_{C\text{-}F}$ = 246.2 Hz), 165.2, 167.5, 173.4; HRMS (ESI) m/z: $[M + Na]^+$ calcd. for $C_{12}H_{10}O_4FNa$ 259.0377, found 259.0386.

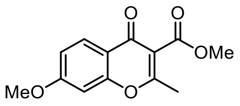

S19

Methyl 7-methoxy-2-methyl-4-oxo-4*H*-chromene-3-carboxylate (S19). Following the general procedure, chromone **S19** (1.37 g, 55% yield over 2 steps) was prepared from 2-fluoro-4-methoxybenzoyl chloride (1.88 g, 10.0 mmol) as a white solid: mp = 109–111 °C; IR (CH_2Cl_2 cast, cm^{-1}) ν_{max} 3070, 3009, 2982, 2951, 2838, 1718, 1645, 1624, 1600, 1568; ^{1}H NMR ($CDCl_3$, 400 MHz): δ 2.49 (s, 3H), 3.90 (s, 3H), 3.93 (s, 3H), 6.81 (d, J = 2.4 Hz, 1H), 6.96 (dd, J = 9.2, 2.4 Hz, 1H), 8.10 (d, J = 9.2 Hz, 1H); ^{13}C NMR ($CDCl_3$, 100 MHz): δ 18.9, 52.0, 55.4, 99.6, 114.2, 116.4, 117.0, 126.7, 156.7, 163.8, 165.2, 166.1, 173.1; HRMS (ESI) m/z: $[M + Na]^+$ calcd. for $C_{13}H_{23}O_5Na$ 271.0577, found 271.0580.

Synthetic procedures and characterization of new [4+2] annulation compounds

Table S1. Screening of vinylogous [4+2] annulation conditions.

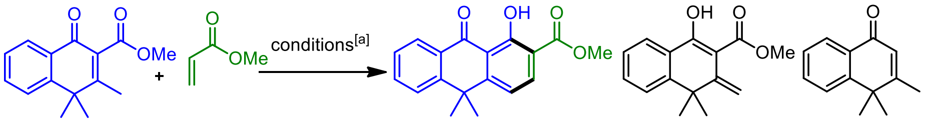

Entry	Michael acceptor	base (equiv.)	solvent	T (°C) / t (h)	isolated yield of 8
1.	(1.5 equiv.)	Li_2CO_3 (1.5 equiv.)	DMF	150 °C / 15 h	trace[b]
2.	(1.5 equiv.)	Na_2CO_3 (1.5 equiv.)	DMF	150 °C / 15 h	18%[b]
3.	(1.5 equiv.)	K_2CO_3 (1.5 equiv.)	DMF	150 °C / 2 h	trace
4.	(1.5 equiv.)	Cs_2CO_3 (1.5 equiv.)	DMF	150 °C / 2 h	0%
5.	(1.5 equiv.)	DBU (1.5 equiv.)	toluene	110 °C / 6 h	0%[c]
6.	(1.5 equiv.)	LiHMDS (1.5 equiv.)	THF	66 °C / 15 h	63%[b]
7.	(1.5 equiv.)	NaHMDS (1.5 equiv.)	THF	66 °C / 2 h	44%
8.	(1.5 equiv.)	KHMDS (1.5 equiv.)	THF	0 °C to 66 °C / 2 h	25%
9.	(1.5 equiv.)	NaH (2.0 equiv.)	THF	66 °C / 15 h	24%
10.	(1.5 equiv.)	LiHMDS (1.5 equiv)	toluene	110 °C / 15 h	75%
11.	(1.1 equiv.)	LiHMDS (1.1 equiv)	toluene	110 °C / 15 h	83%
12.	(1.1 equiv.)	^{t}BuOLi (1.1 equuiv.)	toluene	110 °C / 6 h	79%
13.	(1.1 equiv.)	^{t}BuONa (1.1 equiv.)	toluene	110 °C / 6 h	61%
14.	(1.1 equiv.)	^{t}BuOK (1.1 equiv.)	toluene	110 °C / 6 h	trace
15.	(1.1 equiv.)	LiHMDS (1.1 equiv)	toluene	0 °C / 6 h	0%[b]
16.	none	LiHMDS (1.1 equiv)	toluene	110 °C / 24 h	0%[b] [d]

[a] All reactions were performed in solvent (0.2 M) as indicated above under dry air. [b] Donor **5** and its dienol tautomer **5a** was not completely consumed and recovered. [c] Only donor **5** was recovered. [d] Decarboxylated compound **5b** was isolated in 37% yield.

Characterization of dienol tautomer **5a** and decarboxylated compound **5b**:

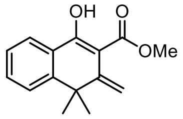

5a

Methyl 1-hydroxy-4,4-dimethyl-3-methylene-3,4-dihydronaphthalene-2-carboxylate (5a). ^{1}H and ^{13}C NMR data of the crude mixture of donor **5** and its dienol tautomer **5a** in different ratio along progress of time are provided (see spectra S49, S50 and S51). ^{1}H and ^{13}C NMR data of **5a**: ^{1}H NMR ($CDCl_3$, 400 MHz): δ 1.46 (s, 6H), 3.89 (s, 3H), 5.28 (s, 1H), 5.34 (s, 1H), 7.28-7.31 (m, 1H), 7.39-7.41 (m, 2H), 7.89 (dd, J = 7.6, 0.8 Hz, 1H), 13.17 (s, 1H); ^{13}C NMR ($CDCl_3$, 100 MHz): δ 27.3, 40.3, 52.0, 99.6, 111.1, 123.1, 125.4, 126.3, 128.2, 131.6,

145.0, 147.8, 165.4, 173.2.

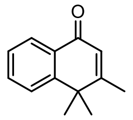

5b

3,4,4-Trimethylnaphthalen-1(4*H*)-one (5b). Compound **5b** was islated as a colorless oil: IR (CH_2Cl_2 cast, cm^{-1}) ν_{max} 3064, 3033, 2977, 2933, 2872, 1660, 1601, 1574; ^{1}H NMR ($CDCl_3$, 400 MHz): δ 1.51 (s, 6H), 2.15 (d, J = 1.2 Hz, 3H), 6.33 (q, J = 1.2 Hz, 1H), 7.36-7.40 (m, 1H), 7.56-7.61 (m, 2H), 8.17 (dd, J = 7.6, 0.8 Hz, 1H); ^{13}C NMR ($CDCl_3$, 100 MHz): δ 20.3, 28.5, 40.4, 126.3, 126.3, 126.5, 126.5, 130.4, 132.4, 151.1, 165.5, 184.5; HRMS (ESI) m/z: $[M + H]^+$ calcd. for $C_{13}H_{15}O$ 187.1117, found 187.1119.

Scope of vinylogous donors and Michael acceptors:

General procedure for condition A:

To a stirred solution of vinylogous donor (0.50 mmol) and Michael acceptor (0.55 mmol, 1.1 equiv.) in dry toluene (2.0 mL) at 0 °C was added LiHMDS (1 M in toluene, 0.55 mL, 1.1 equiv.) dropwise under N_2. The mixture was then allowed to react at the indicated temperature and time as listed in Tables 2 and 3 under dry air. After the reaction was complete, sat. $NH_4Cl_{(aq)}$ (5.0 mL) was added to quench the reaction. The organic layer was separated, and the aqueous layer was extracted with EtOAc (20 mL). The organic portions were combined, washed with water and brine, dried over $MgSO_4$, filtered and concentrated to give the crude residue, which was purified by chromatography on silical gel to afford the annulated product.

General procedure for condition B:

To a stirred solution of vinylogous donor (0.50 mmol) and Michael acceptor (0.55 mmol, 1.1 equiv.) in dry toluene (2.0 mL) at 0 °C was added LiHMDS (1 M in toluene, 0.55 mL) dropwise under N_2. The mixture was then allowed to react at the indicated temperature and time as listed in Table 2 under dry air. After the reaction was complete, sat. $NH_4Cl_{(aq)}$ (5 mL) was added to quench the reaction. The organic layer was separated, and the aqueous layer was extracted with EtOAc (20 mL). The organic portions were combined, washed with water and brine, dried over $MgSO_4$, filtered to give the solution of crude residue, which was mixture of aromatized product and non-aromatized intermediate. To the solution of mixture was added DDQ (114 mg, 0.50 mmol), and then allowed to react at room temperature under N_2 for 1 h. After reaction was complete, sat. $NaHCO_{3(aq)}$ (20 mL) was added to quench the reaction. The separated organic layer was washed with sat. $NaHCO_{3(aq)}$ (10 mL × 2) and brine, dried over $MgSO_4$, filtered and concentrated to give the crude residue, which was purified by

chromatography on silical gel to afford the annulated product.

General procedure for condition C:

To a stirred solution of vinylogous donor (0.50 mmol) and Michael acceptor (1.05 mmol, 2.1 equiv.) in dry toluene (2.0 mL) at 0 °C was added LiHMDS (1 M in toluene, 0.55 mL) dropwise under N_2. The mixture was then allowed to react at the indicated temperature and time as listed in Table 2 under dry air. After the reaction was complete, sat. $NH_4Cl_{(aq)}$ (5 mL) was added to quench the reaction. The organic layer was separated, and the aqueous layer was extracted with EtOAc (20 mL). The organic portions were combined, washed with water and brine, dried over $MgSO_4$, filtered and concentrated to give the crude residue, which was purified by chromatography on silical gel to afford the annulated product.

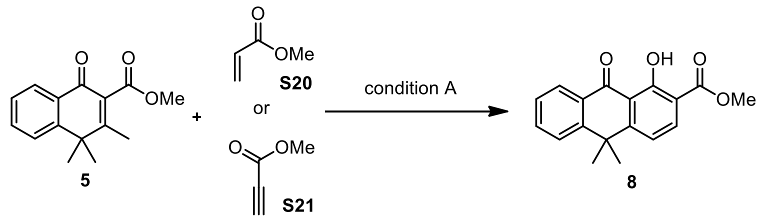

Methyl 1-hydroxy-10,10-dimethyl-9-oxo-9,10-dihydroanthracene-2-carboxylate (8). Following the general procedure of condition A, product **8** (123 mg, 83% yield) was prepared from donor **5** (122 mg, 0.50 mmol) and methyl acrylate (**S20**) (47 mg, 0.55 mmol) as a yellow solid: mp = 149–151 °C; IR (CH_2Cl_2 cast, cm^{-1}) ν_{max} 3070, 2976, 2950, 2873, 1732, 1703, 1627, 1600; ^1H NMR ($CDCl_3$, 400 MHz): δ 1.73 (s, 6H), 3.96 (s, 3H), 7.17 (d, J = 8.4 Hz, 1H), 7.46-7.50 (m, 1H), 7.67-7.73 (m, 2H), 8.17 (d, J = 8.4 Hz, 1H), 8.37 (d, J = 8.8 Hz, 1H); ^{13}C NMR ($CDCl_3$, 100 MHz): δ 32.9, 38.6, 52.1, 115.2, 116.3, 116.5, 126.6, 127.0, 127.3, 128.8, 134.5, 138.4, 150.4, 157.1, 163.9, 165.8, 189.1; HRMS (ESI) m/z: $[M + H]^+$ calcd. for $C_{18}H_{17}O_4$ 297.1121, found 297.1122. Using methyl propiolate (**S21**) (46 mg, 0.55 mmol) as a Michael acceptor instead, product **8** (73 mg, 49% yield) could also be afforded but in a much lower yield.

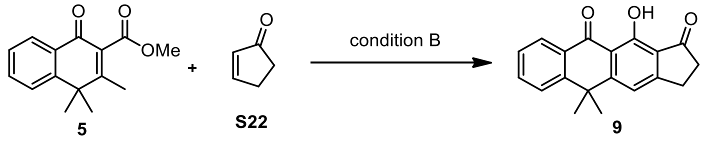

11-Hydroxy-5,5-dimethyl-2,3-dihydro-1*H*-cyclopenta[*b*]anthracene-1,10(5*H*)-dione (9). Following the general procedure of condition B, product **9** (108 mg, 74% yield) was prepared from donor **5** (122 mg, 0.50 mmol) and cyclopent-2-enone (**S22**) (45 mg, 0.55 mmol) as a yellow solid: mp = 209–213 °C; IR (CH_2Cl_2 cast, cm^{-1}) ν_{max} 3065, 2974, 2928, 1713, 1626, 1610, 1597; ^1H NMR ($CDCl_3$, 400 MHz): δ 1.75 (s, 6H), 2.74 (t, J = 6.4 Hz, 2H), 3.18 (t, J =

6.4 Hz, 2H), 7.19 (s, 1H), 7.46-7.50 (m, 1H), 7.69-7.71 (m, 2H), 8.36 (d, *J* = 8.0 Hz, 1H), 14.82 (s, 1H); ^{13}C NMR ($CDCl_3$, 100 MHz): *δ* 26.5, 33.1, 36.5, 39.0, 113.3, 114.9, 122.6, 126.6, 127.0, 127.1, 128.6, 134.5, 150.3, 159.3, 162.5, 163.8, 189.0, 203.2; HRMS (ESI) m/z: $[M + Na]^+$ calcd. for $C_{19}H_{16}O_3Na$ 315.0992, found 315.0994.

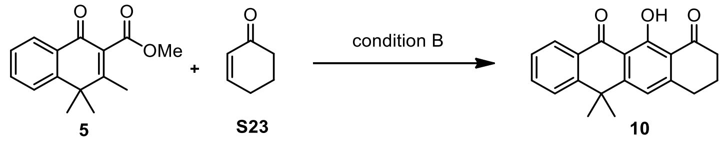

12-Hydroxy-6,6-dimethyl-3,4-dihydrotetracene-1,11(2*H*,6*H*)-dione (10). Following the general procedure of condition B, product **10** (144 mg, 94% yield) was prepared from donor **5** (122 mg, 0.50 mmol) and cyclohex-2-enone (**S23**) (53 mg, 0.55 mmol) as a yellow solid: mp = 191–193 °C; IR (CH_2Cl_2 cast, cm^{-1}) ν_{max} 3065, 3035, 2928, 2872, 2854, 1677, 1618, 1596; ^{1}H NMR (DMSO-d_6, 400 MHz): *δ* 1.69 (s, 6H), 2.02 (quint, *J* = 6.8 Hz, 2H), 2.60 (t, *J* = 6.8 Hz, 2H), 3.02 (t, *J* = 6.8 Hz, 2H), 7.32 (s, 1H), 7.51 (t, *J* = 7.6 Hz, 1H), 7.56 (dd, *J* = 8.0, 1.2 Hz, 1H), 7.90 (d, *J* = 7.6 Hz, 1H), 8.17 (dd, *J* = 8.0, 1.2 Hz, 1H), 14.56 (s, 1H); ^{13}C NMR (DMSO-d_6, 100 MHz): *δ* 22.0, 30.6, 32.3, 38.5, 113.9, 117.6, 117.6, 126.2, 127.1, 127.2, 128.9, 134.3, 149.9, 153.6, 157.5, 164.3, 186.3, 197.8; HRMS (ESI) m/z: $[M + H]^+$ calcd. for $C_{20}H_{19}O_3$ 307.1329, found 307.1336.

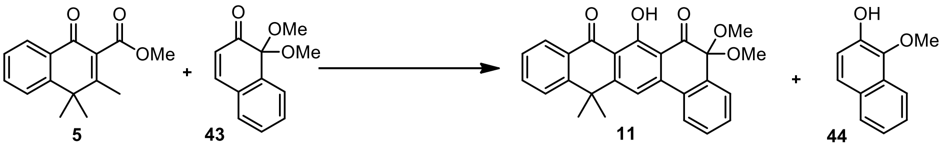

condition A : **11** (51%) / **44** (44%) / recovered **5+5a** (39%)
condition C : **11** (75%) / **44** (62%)

7-Hydroxy-5,5-dimethoxy-13,13-dimethylbenzo[*a*]tetracene-6,8(5*H*,13*H*)-dione (11). Following the general procedure of condition C, product **11** (155 mg, 75% yield) was prepared from donor **5** (122 mg, 0.50 mmol) and acceptor **43** (214 mg, 1.05 mmol) as a yellow solid: mp = 196–198 °C; IR (CH_2Cl_2 cast, cm^{-1}) ν_{max} 3067, 2977, 2939, 2836, 2726, 1721, 1624, 1597; ^{1}H NMR ($CDCl_3$, 400 MHz): *δ* 1.82 (s, 6H), 3.42 (s, 6H), 7.44-7.54 (m, 3H), 7.58 (s, 1H), 7.69-7.74 (m, 2H), 7.86 (dd, *J* = 7.6, 1.2 Hz, 1H), 7.90 (d, *J* = 7.6 Hz, 1H), 8.37 (d, *J* = 7.6 Hz, 1H), 14.63 (s, 1H); ^{13}C NMR ($CDCl_3$, 100 MHz): *δ* 33.0, 38.9, 51.7, 97.5, 112.1, 114.8, 116.3, 125.7, 126.6, 127.1, 127.1, 128.3, 128.7, 129.5, 129.9, 132.1, 134.5, 1349.9, 143.7, 150.3, 157.4, 163.3, 188.1, 193.9; HRMS (ESI) m/z: $[M + Na]^+$ calcd. for $C_{26}H_{22}O_5Na$ 437.1359, found 437.1367. Byproduct **44**[7] (54 mg, 62%) was also isolated as a white solid.

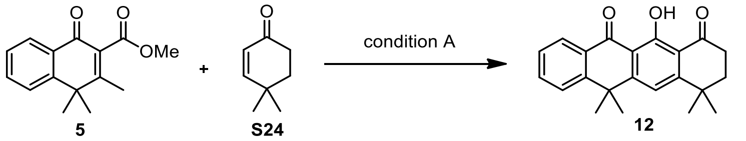

12-Hydroxy-4,4,6,6-tetramethyl-3,4-dihydrotetracene-1,11(2*H*,6*H*)-dione (12). Following the general procedure of condition A, product **12** (115 mg, 69% yield) was prepared from donor **5** (122 mg, 0.50 mmol) and 4,4-dimethylcyclohex-2-enone (**S24**) (68 mg, 0.55 mmol) as a yellow solid: mp = 211–213 °C; IR (CH_2Cl_2 cast, cm^{-1}) ν_{max} 3066, 2965, 2929, 2867, 2712, 1686, 1668, 1622, 1596; ^1H NMR (DMSO-d_6, 400 MHz): δ 1.40 (s, 6H), 1.71 (s, 6H), 1.93 (t, J = 6.8 Hz, 2H), 2.67 (t, J = 6.8 Hz, 2H), 7.37 (s, 1H), 7.49 (t, J = 8.0 Hz, 1H), 7.75 (t, J = 7.6 Hz, 1H), 7.90 (d, J = 8.0 Hz, 1H), 8.16 (d, J = 7.6 Hz, 1H), 14.56 (s, 1H); ^{13}C NMR (DMSO-d_6, 100 MHz): δ 29.1, 32.4, 35.0, 35.6), 35.7, 38.7, 114.0, 114.8, 116.3, 126.2, 127.0, 127.2, 129.0, 134.2, 149.9, 158.1, 160.3, 163.9, 185.6, 198.7; HRMS (ESI) m/z: $[M + Na]^+$ calcd. for $C_{22}H_{22}O_3Na$ 357.1461, found 357.1463.

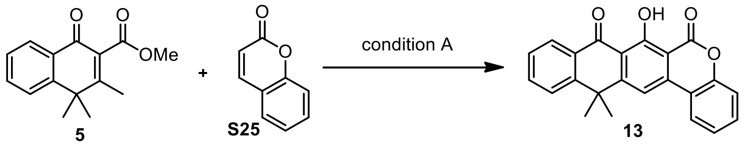

7-Hydroxy-13,13-dimethyl-6*H*-anthra[2,3-*c*]chromene-6,8(13*H*)-dione (13). Following the general procedure of condition A, product **13** (151 mg, 85% yield) was prepared from donor **5** (122 mg, 0.50 mmol) and coumarin **S25** (80 mg, 0.55 mmol) as a yellow solid: mp = 240–243 °C; IR (CH_2Cl_2 cast, cm^{-1}) ν_{max} 3068, 2978, 2926, 2854, 2718, 2673, 1743, 1683, 1609; ^1H NMR ($CDCl_3$, 400 MHz): δ 1.85 (s, 6H), 7.35 (d, J = 8.0 Hz, 1H), 7.38 (d, J = 8.0 Hz, 1H), 7.48-7.58 (m, 2H), 7.72-7.77 (m, 2H), 7.83 (s, 1H), 8.14 (d, J = 8.0 Hz, 1H), 8.39 (d, J = 8.0 Hz, 1H), 15.55 (brs, 1H); ^{13}C NMR ($CDCl_3$, 100 MHz): δ 33.2, 39.0, 107.0, 109.4, 114.1, 117.1, 117.8, 123.7, 124.4, 126.6, 127.3, 127.4, 128.7, 132.3, 134.8, 142.1, 149.9, 152.6, 157.3, 158.4, 167.8, 188.1; HRMS (ESI) m/z: $[M + Na]^+$ calcd. for $C_{23}H_{16}O_4Na$ 379.0941, found 379.0943.

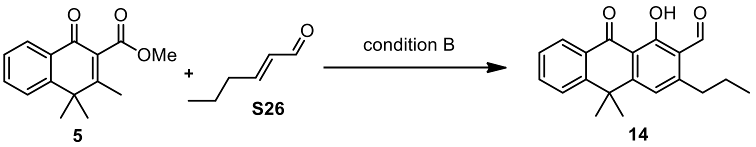

1-Hydroxy-10,10-dimethyl-9-oxo-3-propyl-9,10-dihydroanthracene-2-carbaldehyde (14). Following the general procedure of condition B, product **14** (145 mg, 94% yield) was prepared from donor **5** (122 mg, 0.50 mmol) and (*E*)-hex-2-enal (**S26**) (54 mg, 0.55 mmol) as a yellow solid: mp = 109–112 °C; IR (CH_2Cl_2 cast, cm^{-1}) ν_{max} 3068, 3036, 2963, 2931, 2931,

2871, 1686, 1625, 1598; ^{1}H NMR ($CDCl_3$, 400 MHz): δ 1.02 (t, J = 7.6 Hz, 3H), 1.63 (sext, J = 7.6 Hz, 2H), 1.73 (s, 6H), 3.06 (t, J = 7.6 Hz, 2H), 6.96 (s, 1H), 7.46-7.50 (m, 1H), 7.67-7.71 (m, 2H) 8.36 (d, J = 8.4 Hz, 1H), 10.73 (s, 1H), 14.46 (s, 1H); ^{13}C NMR ($CDCl_3$, 100 MHz): δ 14.1, 24.2, 32.7, 36.9, 38.5, 113.0, 119.7, 119.8, 126.7, 127.0, 128.6, 134.4, 150.4, 154.0, 157.1, 168.2, 188.4, 190.6; HRMS (ESI) m/z: $[M + H]^+$ calcd. for $C_{20}H_{21}O_3$ 309.1485, found 309.1489.

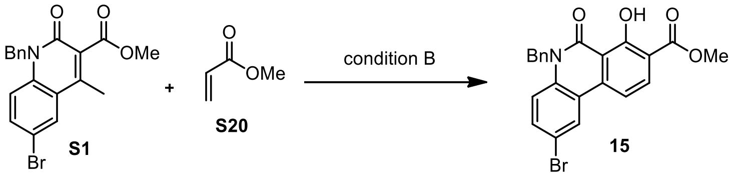

Methyl 5-benzyl-2-bromo-7-hydroxy-6-oxo-5,6-dihydrophenanthridine-8-carboxylate (15). Following the general procedure of condition A, product **15** (202 mg, 92% yield) was prepared from donor **S1** (193 mg, 0.50 mmol) and methyl acrylate (**S20**) (47 mg, 0.55 mmol) as a light yellow solid: mp = 233–236 °C; IR (KBr, cm^{-1}) ν_{max} 3081, 3031, 2946, 1731, 1700, 1635, 1607, 1581; ^{1}H NMR ($CDCl_3$, 400 MHz): δ 3.99 (s, 3H), 5.61 (brs, 2H), 7.22 (d, J = 8.8 Hz, 1H), 7.20-7.35 (m, 5H), 7.55 (dd, J = 8.8, 2.0 Hz, 1H), 7.67 (d, J = 8.4 Hz, 1H), 8.28 (d, J = 8.4 Hz, 1H), 8.38 (d, J = 2.0 Hz, 1H), 14.59 (s, 1H); ^{13}C NMR ($CDCl_3$, 100 MHz): δ 46.0, 52.3, 110.8, 111.0, 116.6, 117.1, 118.0, 120.9, 126.3, 127.2, 127.7, 129.1, 133.7, 135.0, 135.8, 137.1, 137.3, 163.4, 165.4, 165.7; HRMS (ESI) m/z: $[M + Na]^+$ calcd. for $C_{22}H_{16}Br\ NO_4$ 460.0155, found 460.0164.

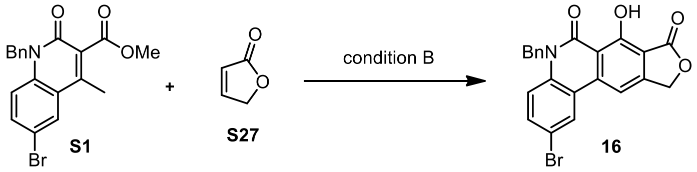

5-Benzyl-2-bromo-7-hydroxyfuro[3,4-*j*]phenanthridine-6,8(5*H*,10*H*)-dione (16). Following the general procedure of condition B, product **16** (166 mg, 76% yield) was prepared from donor **S1** (193 mg, 0.50 mmol) and 2-furanone (**S27**) (46 mg, 0.55 mmol) as a white solid: mp > 300 °C; IR (KBr, cm^{-1}) ν_{max} 3129, 3087, 2961, 2925, 2853, 1764, 1647, 1612, 1585; ^{1}H NMR (DMSO-d_6, 400 MHz): δ 5.48 (s, 2H), 5.64 (s, 2H), 7.25-7.35 (m, 5H), 7.71 (d, J = 9.2 Hz, 1H), 7.79 (dd, J = 9.2, 2.0 Hz, 1H), 8.28 (s, 1H), 8.75 (d, J = 2.0 Hz, 1H), 14.58 (br s, 1H); ^{13}C NMR (DMSO-d_6, 175 MHz): 45.1, 69.0, 106.3, 109.6, 109.8, 116.8, 119.1, 120.9, 126.4, 127.4, 127.6, 128.8, 134.1, 135.5, 135.9, 138.9, 154.3, 161.4, 165.3, 167.4; HRMS (ESI) m/z: $[M + Na]^+$ calcd. for $C_{22}H_{14}BrNO_4Na$ 457.9998, found 458.0006.

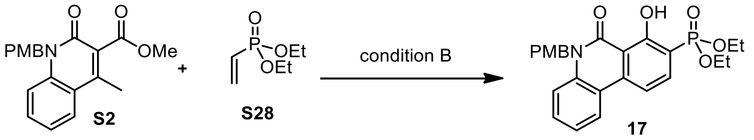

Diethyl (7-hydroxy-5-(4-methoxybenzyl)-6-oxo-5,6-dihydrophenanthridin-8-yl)phosphonate (17). Following the general procedure of condition B, product **17** (166 mg, 71% yield) was prepared from donor **S2** (169 mg, 0.50 mmol) and diethyl vinylphosphonate (**S28**) (90 mg, 0.55 mmol) as a white solid: mp = 92–94 °C; IR (CH_2Cl_2 cast, cm^{-1}) ν_{max} 3457, 2982, 2934, 2905, 2837, 1633, 1601, 1514; ^{1}H NMR ($CDCl_3$, 400 MHz): δ 1.39 (t, J = 6.8 Hz, 6H), 3.76 (s, 3H), 4.19-4.31 (m, 4H), 5.55 (br s, 2H), 6.85 (d, J = 8.8 Hz, 2H), 7.19 (d, J = 8.8 Hz, 2H), 7.34 (td, J = 8.0, 1.2 Hz, 1H), 7.39 (d, J = 8.4 Hz, 1H), 7.49 (td, J = 8.0, 1.2 Hz, 1H), 7.77 (dd, J = 8.4, 2.8 Hz, 1H), 8.22 (dd, J = 14.0, 8.0 Hz, 1H), 8.28 (dd, J = 8.0, 1.2 Hz, 1H), 14.39 (s, 1H); ^{13}C NMR ($CDCl_3$, 100 MHz): δ 16.3 (d, $J_{C\text{-}P}$ = 6.9 Hz), 45.1, 55.0, 62.3 (d, $J_{C\text{-}P}$ = 5.4 Hz), 109.9 (d, $J_{C\text{-}P}$ = 11.4 Hz), 111.0 (d, $J_{C\text{-}P}$ = 14.5 Hz), 113.0 (d, JC-P = 189.1 Hz), 114.2, 116.1, 119.0, 123.6, 124.3, 127.3, 127.7, 130.8, 136.7, 138.7, 139.4, 158.8, 164.8 (d, $J_{C\text{-}P}$ = 4.6 Hz), 165.3; HRMS (ESI) m/z: $[M + Na]^+$ calcd. for $C_{25}H_{26}NO_6PNa$ 490.1390, found 490.1400.

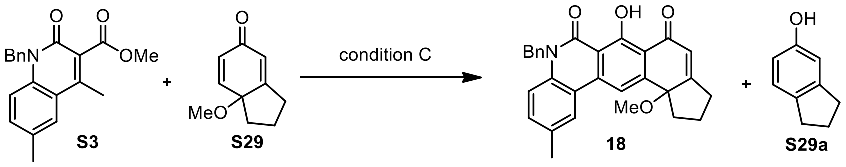

5-Benzyl-7-hydroxy-12a-methoxy-2-methyl-10,11,12,12a-tetrahydro-5*H*-indeno[5,4-*j*]phenanthridine-6,8-dione (18). Following the general procedure of condition C, product **18** (194 mg, 86% yield) was prepared from donor **S3** (161 mg, 0.50 mmol) and acceptor **S29** (172 mg, 1.05 mmol) as a brown solid: mp = 236–239 °C; IR (CH_2Cl_2 cast, cm^{-1}) ν_{max} 3031, 2928, 2820, 1677, 1654, 1624, 1597; ^{1}H NMR ($CDCl_3$, 400 MHz): δ 1.85-2.02 (m, 2H), 2.23-2.36 (m, 1H), 2.47 (s, 3H), 2.56-2.69 (m, 2H), 2.79-2.91 (m, 1H), 2.93 (s, 3H), 5.62 (br s, 2H), 6.44 (dd, J = 2.4, 1.2 Hz, 1H), 7.23-7.33 (m, 7H), 7.89 (s, 1H), 8.11 (s, 1H), 15.09 (br s, 1H); ^{13}C NMR ($CDCl_3$, 100 MHz): δ 20.8, 20.9, 28.6, 38.2, 45.8, 51.3, 79.7, 110.2, 110.5, 116.2, 117.3, 118.6, 124.6, 126.4, 127.4, 127.6, 128.8, 132.4, 133.2, 135.3, 135.7, 138.5, 148.1, 161.1, 164.3, 164.6, 184.6; HRMS (ESI) m/z: $[M + Na]^+$ calcd. for $C_{29}H_{25}NO_4Na$ 474.1676, found 474.1678. Byproduct **S29a** (54 mg, 80% yield), commercially available, was also isolated as a white solid.

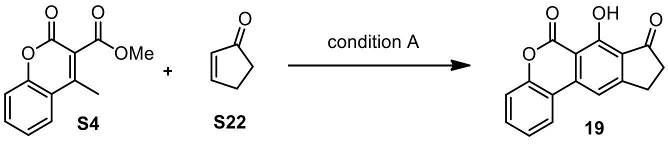

7-Hydroxy-9,10-dihydroindeno[5,6-*c*]chromene-6,8-dione (19). Following the general procedure of condition A, product **19** (109 mg, 82% yield) was prepared from donor **S4** (109 mg, 0.50 mmol) and cyclopent-2-enone (**S22**) (45 mg, 0.55 mmol) as a white solid: mp = 255–259 °C; IR (CH_2Cl_2 cast, cm^{-1}) ν_{max} 3063, 2925, 1708, 1666, 1627, 1606; ^{1}H NMR ($CDCl_3$, 400 MHz): δ 2.79 (t, *J* = 6.4 Hz, 2H), 3.26 (t, *J* = 6.4 Hz, 2H), 7.40 (d, *J* = 7.2 Hz, 1H), 7.41 (t, *J* = 7.2 Hz, 1H), 7.59 (t, *J* = 7.2 Hz, 1H), 7.63 (s, 1H), 8.08 (d, *J* = 7.2 Hz, 1H), 12.49 (s, 1H); ^{13}C NMR ($CDCl_3$, 100 MHz): δ 26.6, 36.5, 104.9, 109.7, 117.4, 117.8, 123.3, 124.0, 125.5, 132.1, 140.7, 151.0, 161.3, 164.7, 165.1, 202.5; HRMS (ESI) m/z: $[M + Na]^+$ calcd. for $C_{16}H_{10}O_4Na$ 289.0471, found 289.0475.

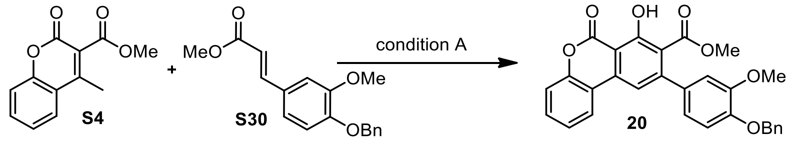

Methyl 9-(4-(benzyloxy)-3-methoxyphenyl)-7-hydroxy-6-oxo-6*H*-benzo[*c*]chromene-8-carboxylate (20). Following the general procedure of condition A, product **20** (205 mg, 85% yield) was prepared from donor **S4** (109 mg, 0.50 mmol) and acceptor **S30** (164 mg, 0.55 mmol) as a white solid: mp = 208–211 °C; IR (CH_2Cl_2 cast, cm^{-1}) ν_{max} 3062, 3032, 2950, 1731, 1682, 1621, 1611; ^{1}H NMR ($CDCl_3$, 400 MHz): δ 3.73 (s, 3H), 3.93 (s, 3H), 5.22 (s, 2H), 6.97 (d, *J* = 8.4 Hz, 1H), 6.98 (dd, *J* = 8.4, 1.2 Hz, 1H), 7.04 (d, *J* = 1.2 Hz, 1H), 7.31-7.57 (m, 8H), 7.58 (s, 1H), 8.06 (d, *J* = 8.0 Hz, 1H), 11.83 (s, 1H); ^{13}C NMR ($CDCl_3$, 100 MHz): δ 52.5, 56.1, 70.9, 104.6, 111.6, 113.2, 113.8, 117.6, 117.8, 120.6, 121.3, 123.5, 125.3, 127.3, 128.0, 128.6, 131.4, 132.1, 135.8, 136.7, 148.7, 149.6, 150.9, 159.7, 165.0, 167.0; HRMS (ESI) m/z: $[M + H]^+$ calcd. for $C_{29}H_{23}O_7$ 483.1438, found 483.1434.

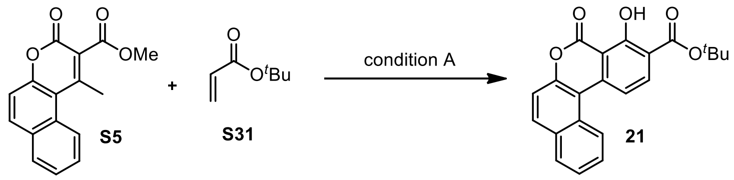

***tert*-Butyl 4-hydroxy-5-oxo-5*H*-dibenzo[*c*,*f*]chromene-3-carboxylate (21).** Following the general procedure of condition A, product **21** (65 mg, 36% yield) was prepared from donor **S5** (134 mg, 0.50 mmol) and *tert*-butyl acrylate (**S31**) (70 mg, 0.55 mmol) as a light yellow solid: mp = 173–175 °C; IR (CH_2Cl_2 cast, cm^{-1}) ν_{max} 3059, 2975, 2930, 1750, 1724, 1697, 1678, 1615; ^{1}H NMR ($CDCl_3$, 400 MHz): δ 1.66 (s, 9H), 7.47 (d, *J* = 8.4 Hz, 1H), 7.58 (t, *J* = 7.2 Hz, 1H), 7.67 (t, *J* = 7.2 Hz, 1H), 7.97 (t, *J* = 8.4 Hz, 2H), 8.08 (d, *J* = 8.4 Hz, 1H), 8.28 (d, *J* = 8.4 Hz, 1H), 8.71 (d, *J* = 8.4 Hz, 1H), 12.60 (s, 1H); ^{13}C NMR ($CDCl_3$, 100 MHz): δ 28.2, 82.7, 108.5, 112.3, 116.3, 116.6, 117.0, 124.9, 125.8, 128.1, 129.4, 129.4, 131.7, 133.0, 137.6,

140.3, 150.6, 162.5, 163.4, 165.5; HRMS (ESI) m/z: $[M + Na]^+$ calcd. for $C_{22}H_{18}O_5Na$ 385.1046, found 385.1051.

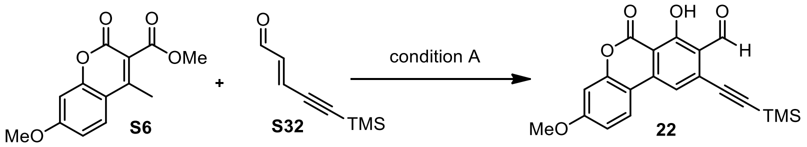

7-Hydroxy-3-methoxy-6-oxo-9-((trimethylsilyl)ethynyl)-6*H*-benzo[*c*]chromene-8-carbaldehyde (22). Following the general procedure of condition C, product **22** (114 mg, 62% yield) was prepared from donor **S6** (124 mg, 0.50 mmol) and acceptor **S32** (80 mg, 0.55 mmol) as a yellow solid: mp = 203–205 °C; IR (CH_2Cl_2 cast, cm^{-1}) ν_{max} 3095, 2960, 2851, 2156, 1704, 1693, 1606, 1584; ^1H NMR ($CDCl_3$, 400 MHz): δ 0.32 (s, 9H), 3.89 (s, 3H), 6.81 (d, J = 2.8 Hz, 1H), 6.94 (dd, J = 8.8, 2.8 Hz, 1H), 7.58 (s, 1H), 7.91 (d, J = 8.8 Hz, 1H), 10.52 (s, 1H), 12.79 (br s, 1H); ^{13}C NMR ($CDCl_3$, 100 MHz): δ -0.13, 55.9, 100.1, 101.4, 105.8, 107.1, 109.6, 113.6, 117.0, 120.2, 125.3, 133.2, 140.9, 153.3, 162.1, 163.4, 165.0, 190.6; HRMS (ESI) m/z: $[M + Na]^+$ calcd. for $C_{20}H_{18}O_5SiNa$ 389.0816, found 389.0809.

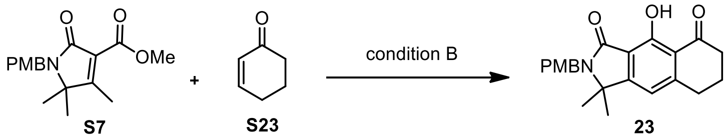

9-Hydroxy-2-(4-methoxybenzyl)-3,3-dimethyl-2,3,6,7-tetrahydro-1*H*-benzo[*f*]isoindole-1,8(5*H*)-dione (23). Following the general procedure of condition B, product **23** (133 mg, 73% yield) was prepared from donor **S7** (152 mg, 0.50 mmol) and cyclohex-2-enone (**S23**) (53 mg, 0.55 mmol) as a white solid: mp = 250–254 °C; IR (CH_2Cl_2 cast, cm^{-1}) ν_{max} 3033, 2996, 2968, 2862, 1677, 1616; ^1H NMR ($CDCl_3$, 400 MHz): δ 1.31 (s, 6H), 2.13 (quint, J = 6.0 Hz, 2H), 2.72 (t, J = 6.0 Hz, 2H), 3.00 (t, J = 6.0 Hz, 2H), 3.78 (s, 3H), 4.64 (s, 2H), 6.68 (s, 1H), 6.90 (d, J = 8.8 Hz, 2H), 7.32 (d, J = 8.8 Hz, 2H), 13.35 (s, 1H); ^{13}C NMR ($CDCl_3$, 100 MHz): δ 22.4, 25.9, 30.5, 38.7, 41.5, 55.0, 62.3, 111.1, 113.5, 115.5, 116.4, 129.0, 130.8, 150.2, 158.5, 160.8, 160.9, 165.4, 204.8; HRMS (ESI) m/z: $[M + H]^+$ calcd. for $C_{22}H_{24}NO_4$ 366.1700, found 366.1708.

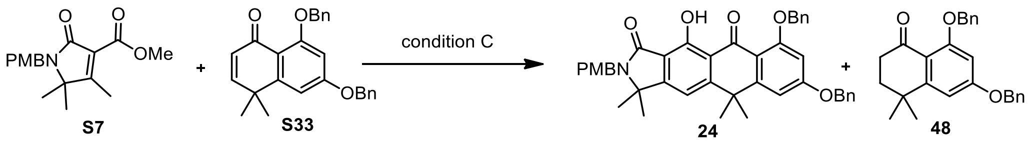

7,9-Bis(benzyloxy)-11-hydroxy-2-(4-methoxybenzyl)-3,3,5,5-tetramethyl-2,3-dihydro-1H-naphtho[2,3-*f*]isoindole-1,10(5*H*)-dione (24). Following the general procedure of condition

C, product **24** (288 mg, 88% yield) was prepared from donor **S7** (152 mg, 0.50 mmol) and acceptor **S33** (404 mg, 1.05 mmol) as a yellow oil: IR (CH_2Cl_2 cast, cm^{-1}) ν_{max} 3062, 3033, 2975, 2932, 2836, 1687, 1633, 1599; ^{1}H NMR ($CDCl_3$, 400 MHz): δ 1.36 (s, 6H), 1.68 (s, 6H), 3.78 (s, 3H), 4.67 (s, 2H), 5.12 (s, 2H), 5.27 (s, 2H), 6.60 (d, *J* = 2.4 Hz. 1H), 6.81 (d, *J* = 11.6 Hz, 2H), 6.83 (d, *J* = 2.4 Hz, 1H), 6.97 (s, 1H), 7.32 (d, *J* = 11.6 Hz, 2H), 7.37-7.60 (m, 10H), 14.94 (s, 1H); ^{13}C NMR ($CDCl_3$, 100 MHz): δ 26.3, 34.0, 39.7, 41.7, 55.1, 62.4, 70.2, 70.9, 99.4, 105.3, 107.7, 113.2, 113.6, 115.1, 115.3, 126.5, 127.5, 127.7, 128.4, 128.5, 128.7, 129.1, 131.1, 135.6, 136.3, 155.0, 155.2, 158.6, 159.3, 161.4, 162.3, 163.7, 165.8, 187.8; HRMS (ESI) m/z: $[M + H]^+$ calcd. for $C_{42}H_{40}NO_6$ 654.2850, found 654.2857. Byproduct **48** (160 mg, 83%) was also isolated as a white solid: mp = 114–115 °C; IR (CH_2Cl_2 cast, cm^{-1}) ν_{max} 3032, 2961, 2926, 2864, 1670, 1595, 1568, 1324, 1157; ^{1}H NMR ($CDCl_3$, 400 MHz): δ 1.33 (s, 6H), 1.93 (t, *J* = 6.8 Hz, 2H), 2.68 (t, *J* = 6.8 Hz, 2H), 5.08 (s, 2H), 5.14 (s, 2H), 6.48 (d, *J* = 2.2 Hz, 1H), 6.59 (d, *J* = 2.2 Hz, 1H), 7.29-7.43 (m, 8H), 7.58 (d, *J* = 7.2 Hz, 2H); ^{13}C NMR ($CDCl_3$, 100 MHz): δ 29.7, 34.8, 36.4, 36.6, 70.0, 70.5, 98.7, 104.1, 115.9, 126.6, 127.5, 127.6, 128.2, 128.4, 128.6, 136.0, 136.6, 156.7, 161.0, 163.0, 196.0; HRMS (ESI) m/z: $[M + H]^+$ calcd. for $C_{26}H_{27}O_3$ 387.1955, found: 387.1956.

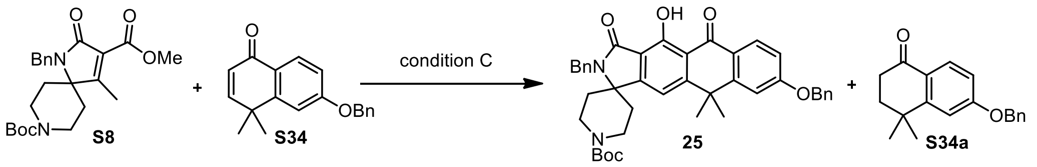

***tert*-Butyl 2-benzyl-8-(benzyloxy)-4-hydroxy-10,10-dimethyl-3,5-dioxo-2,3,5,10-tetrahydrospiro[naphtho[2,3-*f*]isoindole-1,4'-piperidine]-1'-carboxylate (25).** Following the general procedure of condition C, product **25** (277 mg, 84% yield) was prepared from donor **S8** (212 mg, 0.50 mmol) and acceptor **S34** (292 mg, 1.05 mmol) as a yellow solid: mp = 206–208 °C; IR (CH_2Cl_2 cast, cm^{-1}) ν_{max} 3062, 2975, 2929, 2880, 1693, 1635, 1596; ^{1}H NMR ($CDCl_3$, 400 MHz): δ 1.52 (s, 9H), 1.73 (s, 6H), 2.11 (t, *J* = 14.4 Hz, 2H), 3.39 (t, *J* = 14.4 Hz, 2H), 4.11 (br s, 2H), 4.77 (br s, 2H), 7.10 (dd, *J* = 8.8, 2.4 Hz, 1H), 7.19 (d, *J* = 2.4 Hz, 1H), 7.21-7.49 (m, 10H), 7.39 (s, 1H), 8.36 (d, *J* = 8.8 Hz, 1H), 14.56 (s, 1H); ^{13}C NMR ($CDCl_3$, 100 MHz): δ 28.2, 33.0, 33.5, 39.0, 39.9, 42.1, 62.7, 70.2, 80.1, 111.2, 112.5, 114.0, 114.3, 115.2, 122.2, 126.9, 127.1, 127.4, 128.1, 128.3, 128.5, 129.7, 135.7, 138.4, 152.7, 154.6, 155.9, 157.7, 161.4, 163.7, 165.6, 187.6; HRMS (ESI) m/z: $[M + Na]^+$ calcd. for $C_{41}H_{42}N2O_6Na$ 681.2935, found 681.2941. Byproduct **S34a** (107 mg, 76%) was also isolated as a white solid: mp = 104–105 °C; IR (CH_2Cl_2 cast, cm^{-1}) ν_{max} 3065, 3034, 2961, 2928, 2862, 1676, 1597, 1565; ^{1}H NMR ($CDCl_3$, 400 MHz): δ 1.36 (s, 6H), 1.99 (t, *J* = 6.8 Hz, 2H), 2.68 (t, *J* = 6.8 Hz, 2H), 5.12 (s, 2H), 6.89 (dd, *J* = 8.4, 2.4 Hz, 1H), 6.95 (d, *J* = 2.4 Hz, 1H), 7.33-7.46 (m, 5H), 8.02 (d, *J* = 8.4 Hz, 1H); ^{13}C NMR ($CDCl_3$, 100 MHz): δ 29.6, 34.1, 34.8, 37.1, 70.1, 111.8, 112.4,

125.1, 127.6, 128.2, 128.7, 130.0, 136.2, 154.8, 163.1, 197.2; HRMS (ESI) m/z: $[M + H]^+$ calcd. for $C_{19}H_{21}O_2$ 281.1536, found: 281.1544.

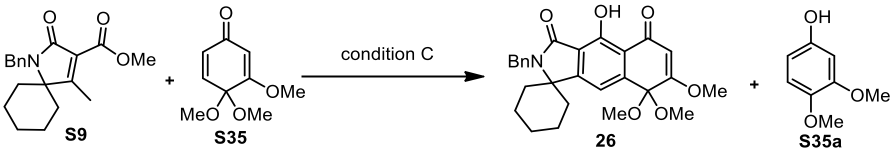

2-Benzyl-4-hydroxy-7,8,8-trimethoxyspiro[benzo[*f*]isoindole-1,1'-cyclohexane]-3,5(2*H*,8*H*)-dione (26). Following the general procedure of condition C, product **26** (192 mg, 83% yield) was prepared from donor **S9** (157 mg, 0.50 mmol) and acceptor **S35** (193 mg, 1.05 mmol) as a white solid: mp = 211–214 °C; IR (CH_2Cl_2 cast, cm^{-1}) ν_{max} 3060, 3028, 2939, 2872, 2852, 1694, 1648, 1614; ^1H NMR ($CDCl_3$, 400 MHz): δ 1.26-1.93 (m, 10 H), 3.13 (s, 6H), 3.97 (s, 3H), 4.76 (s, 2H), 5.92 (s, 1H), 7.19-7.37 (m, 5H), 7.58 (s, 1H), 13.28 (s, 1H); ^{13}C NMR ($CDCl_3$, 100 MHz): δ 22.1, 24.3, 34.0, 42.3, 52.1, 56.6, 65.3, 96.8, 105.7, 112.3, 115.1, 118.7, 126.8, 127.2, 128.3, 138.7, 142.1, 159.2, 165.5, 170.4, 189.7; HRMS (ESI) m/z: $[M + H]^+$ calcd. for $C_{27}H_{30}NO_6$ 464.2068, found 464.2072. Byproduct **S35a** (55 mg, 71%), commercially available, was also isolated as a white solid.

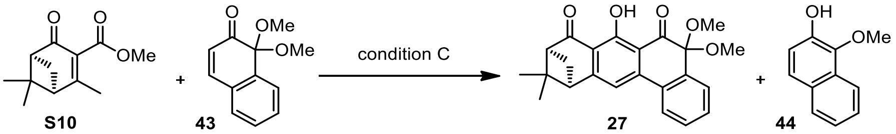

(9*S*,11*R*)-7-Hydroxy-5,5-dimethoxy-10,10-dimethyl-10,11-dihydro-9,11-methanotetraphene-6,8(5*H*,9*H*)-dione (27). Following the general procedure of condition C, product **27** (163 mg, 86% yield) was prepared from donor **S10** (104 mg, 0.50 mmol) and acceptor **43** (214 mg, 1.05 mmol) as a yellow solid: mp = 145–149 °C; IR (CH_2Cl_2 cast, cm^{-1}) ν_{max} 3062, 2955, 2872, 2840, 1721, 1698, 1645, 1620; ^1H NMR ($CDCl_3$, 400 MHz): δ 0.92 (s, 3H), 1.61 (s, 3H), 2.19 (d, J = 10.0 Hz, 1H), 2.88 (t, J = 5.6 Hz, 1H), 3.04 (dt, J = 10.0, 5.6 Hz, 1H), 3.14 (t, J = 5.6 Hz, 1H), 3.34 (s, 3H), 3.43 (s, 3H), 7.20 (s, 1H), 7.42-7.50 (m, 2H), 7.81-7.86 (m, 2H), 12.31 (s, 1H); ^{13}C NMR ($CDCl_3$, 100 MHz): δ 22.5, 26.4, 38.8, 49.3, 51.6, 51.8, 52.1, 57.4, 96.9, 112.2, 114.8, 116.6, 125.7, 128.1, 129.5, 129.8, 131.6, 134.7, 143.2, 156.8, 161.3, 194.9, 204.9; HRMS (ESI) m/z: $[M + Na]^+$ calcd. for $C_{23}H_{22}O_5Na$ 401.1359, found 401.1359. Byproduct **44**[7] (54 mg, 72%) was also isolated as a white solid.

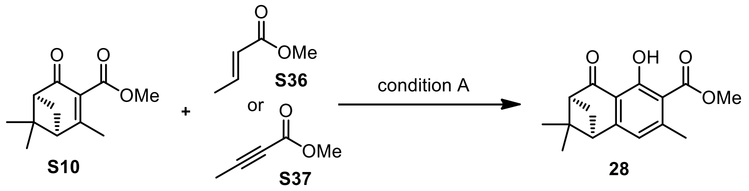

(1*R*,3*S*)-Methyl 5-hydroxy-2,2,7-trimethyl-4-oxo-1,2,3,4-tetrahydro-1,3-methanonaphthalene-6-carboxylate (28). Following the general procedure of condition A, product **28** (77 mg, 56% yield) was prepared from donor **S10** (104 mg, 0.50 mmol) and methyl crotonate (**S36**) (55 mg, 0.55 mmol) as a colorless oil: IR (CH_2Cl_2 cast, cm^{-1}) ν_{max} 3287, 2955, 2872, 1735, 1654, 1648, 1629; ^{1}H NMR ($CDCl_3$, 400 MHz): δ 0.84 (s, 3H), 1.57 (s, 3H), 2.11 (d, *J* = 9.6 Hz, 1H), 2.35 (s, 3H), 2.81 (t, *J* = 5.6 Hz, 1H), 2.96 (t, *J* = 5.6 Hz, 1H), 3.01 (dt, *J* = 9.6, 5.6 Hz, 1H), 3.95 (s, 3H), 6.55 (s, 1H), 11.52 (s, 1H); ^{13}C NMR ($CDCl_3$, 100 MHz): δ 20.6, 22.6, 26.6, 39.8, 48.3, 52.2, 52.8, 57.1, 112.4, 118.8, 120.9, 144.8, 151.4, 158.6, 167.3, 206.9; HRMS (ESI) m/z: $[M + H]^+$ calcd. for $C_{16}H_{19}O_4$ 275.1278, found 275.1279. Using methyl 2-butynoate (**S37**) (54 mg, 0.55 mmol) as a Michael acceptor instead, the annulated product **28** (99 mg, 72% yield) was afforded in a higher yield.

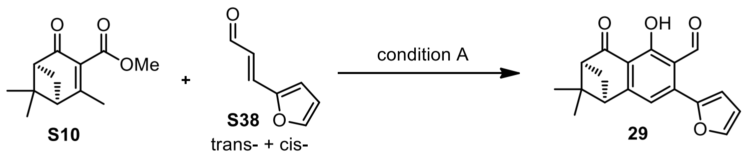

(1*R*,3*S*)-7-(Furan-2-yl)-5-hydroxy-2,2-dimethyl-4-oxo-1,2,3,4-tetrahydro-1,3-methanonaphthalene-6-carbaldehyde (29). Following the general procedure of condition A, annulative product **29** (114 mg, 77% yield) was prepared from donor **S10** (104 mg, 0.50 mmol) and acceptor **S38** (67 mg, 0.55 mmol) as a light orange oil: IR (KBr, cm^{-1}) ν_{max} 3121, 2956, 2926, 2870, 2855, 1697, 1648, 1616; ^{1}H NMR ($CDCl_3$, 400 MHz): δ 0.88 (s, 3H), 1.59 (s, 3H), 2.16 (d, *J* = 10.0 Hz, 1H), 2.89 (t, *J* = 5.6 Hz, 1H), 3.04 (dt, *J* = 10.0, 5.6 Hz, 1H), 3.10 (t, *J* = 5.6 Hz, 1H), 6.58 (dd, *J* = 3.6, 2.0 Hz, 1H), 6.82 (d, *J* = 3.6 Hz, 1H), 7.06 (s, 1H), 7.62 (d, *J* = 2.0 Hz, 1H), 10.31 (s, 1H), 12.13 (brs, 1H); ^{13}C NMR ($CDCl_3$, 100 MHz): δ 22.6, 26.5, 38.9, 49.1, 52.2, 57.5, 112.3, 114.2, 114.3, 116.5, 119.5, 138.6, 144.8, 150.1, 156.0, 162.4, 191.5, 204.7; HRMS (ESI) m/z: $[M + Na]^+$ calcd. for $C_{18}H_{16}O_4Na$ 319.0941, found 319.0937.

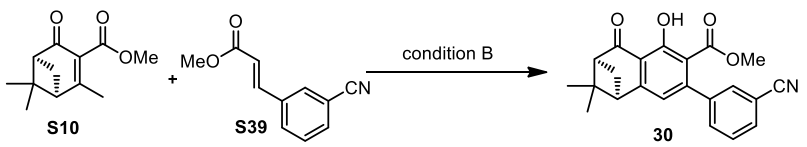

(1*R*,3*S*)-Methyl 7-(3-cyanophenyl)-5-hydroxy-2,2-dimethyl-4-oxo-1,2,3,4-tetrahydro-1,3-methanonaphthalene-6-carboxylate (30). Following the general procedure of condition B, product **30** (117 mg, 65% yield) was prepared from donor **S10** (104 mg, 0.50 mmol) and acceptor **S39** (103 mg, 0.55 mmol) as a white solid: mp = 159–161 °C; IR (CH_2Cl_2 cast, cm^{-1}) ν_{max} 3066, 2954,

2872, 2231, 1733, 1656, 1624; ^{1}H NMR ($CDCl_3$, 400 MHz): δ 0.91 (s, 3H), 1.61 (s, 3H), 2.20 (dt, J = 9.2, 3.6 Hz, 1H), 2.90 (t, J = 5.6 Hz, 1H), 3.07 (d, J = 5.6 Hz, 1H), 3.08 (dt, J = 9.2, 5.6 Hz, 1H), 3.70 (s, 3H), 6.68 (s, 1H), 7.53 (td, J = 7.6, 0.8 Hz, 1H), 7.62 (dt, J = 7.6, 1.6 Hz, 1H), 7.69 (dt, J = 7.6, 1.6 Hz, 1H), 7.70 (d, J = 0.8 Hz, 1H), 11.61 (s, 1H); ^{13}C NMR ($CDCl_3$, 100 MHz): δ 22.7, 26.5, 39.9, 48.4, 52.3, 52.9, 57.3, 112.8, 113.8, 117.9, 118.2, 120.9, 129.2, 131.4, 131.7, 132.3, 140.9, 144.6, 151.9, 158.5, 166.5, 206.9; HRMS (ESI) m/z: $[M + Na]^+$ calcd. for $C_{22}H_{19}NO_4Na$ 384.1206, found 384.1201.

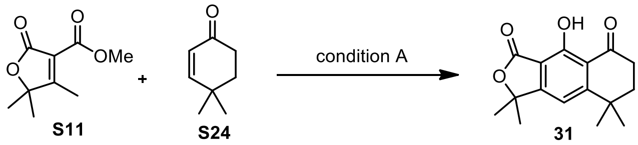

9-Hydroxy-3,3,5,5-tetramethyl-6,7-dihydronaphtho[2,3-*c*]furan-1,8(3*H*,5*H*)-dione (136). Following the general procedure of condition A, product **31** (116 mg, 85% yield) was prepared from donor **S11** (92 mg, 0.50 mmol) and 4,4-dimethylcyclohex-2-enone (**S24**) (68 mg, 0.55 mmol) as a white solid: mp = 227–230 °C; IR (CH_2Cl_2 cast, cm^{-1}) ν_{max} 2984, 2946, 2874, 1760, 1643, 1630, 1613; ^{1}H NMR ($CDCl_3$, 400 MHz): δ 1.42 (s, 6H), 1.64 (s, 6H), 2.03 (t, J = 6.8 Hz, 2H), 2.81 (t, J = 6.8 Hz, 2H), 6.82 (s, 1H), 13.94 (s, 1H); ^{13}C NMR ($CDCl_3$, 100 MHz): δ 26.8, 29.4, 34.5, 35.0, 35.8, 84.0, 107.7, 110.3, 115.5, 161.7, 162.3, 164.0, 166.4, 204.9; HRMS (ESI) m/z: $[M + H]^+$ calcd. for $C_{16}H_{19}O_4$ 275.1278, found 275.1283.

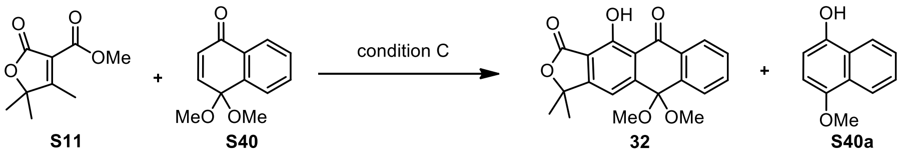

11-Hydroxy-5,5-dimethoxy-3,3-dimethylanthra[2,3-*c*]furan-1,10(3*H*,5*H*)-dione (32). Following the general procedure of condition C, product **32** (142 mg, 80% yield) was prepared from donor **S11** (92 mg, 0.50 mmol) and acceptor **S40** (214 mg, 1.05 mmol) as a yellow solid: mp = 297–299 °C; IR (CH_2Cl_2 cast, cm^{-1}) ν_{max} 3049, 2961, 2835, 1762, 1638, 1612, 1600, 1581; ^{1}H NMR ($CDCl_3$, 400 MHz): δ 1.73 (s, 6H), 2.97 (s, 6H), 7.30 (s, 1H), 7.67 (td, J = 8.0, 1.2 Hz, 1H), 7.85 (td, J = 7.6, 1.6 Hz, 1H), 7.90 (dd, J = 7.6, 1.6, 1H), 8.35 (dd, J = 8.0, 1.2 Hz, 1H), 13.90 (s, 1H); ^{13}C NMR ($CDCl_3$, 100 MHz): δ 26.9, 51.9, 84.4, 97.2, 109.6, 112.9, 117.0, 127.2, 127.2, 130.1, 132.0, 135.2, 139.0, 148.5, 162.0, 164.3, 166.1, 187.8; HRMS (ESI) m/z: $[M + Na]^+$ calcd. for $C_{20}H_{18}O_6Na$ 377.0996, found 377.1006. Byproduct **S40a** (60 mg, 69%), commercially available, was also isolated as a white solid.

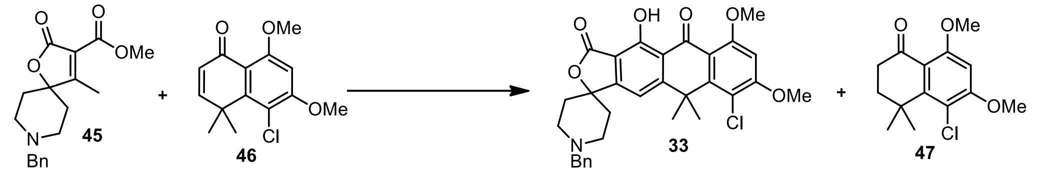

condition A : **33** (48%) / **47** (42%) / recovered **45** (37%)
condition C : **33** (71%) / **47** (54%)

1'-Benzyl-9-chloro-4-hydroxy-6,8-dimethoxy-10,10-dimethyl-3H-spiro[anthra[2,3-*c*]furan-1,4'-piperidine]-3,5(10*H*)-dione (33). Following the general procedure of condition C, annulative product **33** (195 mg, 71% yield) was prepared from donor **45** (158 mg, 0.50 mmol) and acceptor **46** (280 mg, 1.05 mmol) as a yellow solid: mp = 248–251 °C; IR (CH_2Cl_2 cast, cm^{-1}) ν_{max} 3060, 2924, 2851, 2813, 2769, 1762, 1640, 6112, 1580; ^{1}H NMR ($CDCl_3$, 400 MHz): δ 1.77 (d, J = 12.0 Hz, 2H), 2.01 (s, 6H), 2.23 (t, J = 12.0 Hz, 2H), 2.58 (t, J = 10.8 Hz, 2H), 2.94 (d, J = 10.8 Hz, 2H), 3.64 (s, 2H), 4.05 (s, 3H), 4.05 (s, 3H), 6.64 (s, 1H), 6.99 (s, 1H), 7.27-7.39 (m, 5H), 15.02 (br s, 1H); ^{13}C NMR ($CDCl_3$, 100 MHz): δ 29.3, 29.7, 36.0, 40.8, 49.4, 56.6, 56.7, 63.1, 83.2, 95.6, 108.8, 109.6, 113.3, 113.7, 114.1, 127.3, 128.3, 129.3, 149.6, 160.8, 161.0, 161.3, 162.5, 162.8, 166.7, 187.8; HRMS (ESI) m/z: $[M + Na]^+$ calcd. for $C_{31}H_{30}NO_6ClNa$ 570.1654, found 570.1677. Byproduct **47** (73 mg, 54%) was also isolated as a white solid: mp = 94–95 °C; IR (CH_2Cl_2 cast, cm^{-1}) ν_{max} 2936, 2861, 1682, 1578, 1466, 1377, 1330, 1314, 1234, 1214, 1047; ^{1}H NMR ($CDCl_3$, 400 MHz): δ 1.62 (s, 6H), 1.97 (dd, J = 6.8, 4.8 Hz, 2H), 2.60 (dd, J = 6.8, 4.8 Hz, 2H), 3.91 (s, 3H), 3.96 (s, 3H), 6.47 (s, 1H); ^{13}C NMR ($CDCl_3$, 100 MHz): δ 26.7, 36.8, 36.8, 40.4, 56.3, 56.4, 95.0, 114.1, 117.3, 150.8, 159.6, 160.5, 196.6; HRMS (ESI) m/z: $[M + Na]^+$ calcd. for $C_{14}H_{17}ClO_3Na$ 291.0758, found: 291.0760.

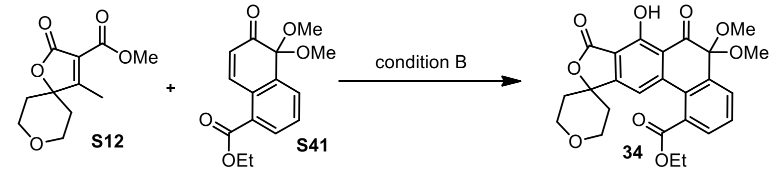

Ethyl 7-hydroxy-5,5-dimethoxy-6,8-dioxo-2',3',5',6,6',8-hexahydro-5*H*-spiro[phenanthro[2,3-*c*]furan-10,4'-pyran]-1-carboxylate (34). Following the general procedure of condition B, annulative product **34** (208 mg, 89% yield) was prepared from donor **S12** (113 mg, 0.50 mmol) and acceptor **S41** (152 mg, 0.55 mmol) as a yellow solid: mp = 210–213 °C; IR (CH_2Cl_2 cast, cm^{-1}) ν_{max} 2921, 2851, 1770, 1721, 1629, 1592; ^{1}H NMR ($CDCl_3$, 400 MHz): δ 1.29 (t, J = 7.2 Hz, 3H), 1.69 (d, J = 13.2 Hz, 2H), 2.17 (td, J = 13.2, 5.2 Hz, 2H), 3.37 (s, 6H), 3.94 (t, J = 11.6 Hz, 2H), 4.04 (dd, J = 11.6, 5.2 Hz, 2H), 4.31 (q, J = 7.2 Hz, 2H), 6.97 (s, 1H), 7.58 (t, J = 7.6 Hz, 1H), 7.69 (dd, J = 7.6, 1.2 Hz, 1H), 7.95 (dd, J = 7.6, 1.2 Hz, 1H), 11.76 (s, 1H); ^{13}C NMR ($CDCl_3$, 100 MHz): δ 14.1, 35.7, 52.5, 62.2, 63.9, 83.1, 96.0, 111.4,

112.7, 114.6, 129.7, 130.2, 130.3, 131.4, 133.1, 133.9, 142.5, 160.0, 161.7, 165.6, 169.7, 197.8; HRMS (ESI) m/z: $[M + Na]^+$ calcd. for $C_{25}H_{24}O_9Na$ 491.1313, found 491.1323.

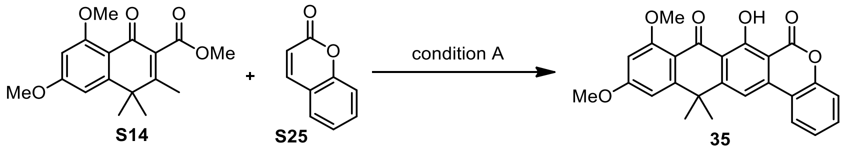

7-Hydroxy-9,11-dimethoxy-13,13-dimethyl-6*H*-anthra[2,3-*c*]chromene-6,8(13*H*)-dione (35). Following the general procedure of condition A, product **35** (164 mg, 79% yield) was prepared from donor **S14** (152 mg, 0.50 mmol) and coumarin **S25** (80 mg, 0.55 mmol) as a yellow solid: mp = 284–285 °C; IR (CH_2Cl_2 cast, cm^{-1}) ν_{max} 2974, 2932, 2848, 1739, 1603, 1588; ^1H NMR ($CDCl_3$, 400 MHz): δ 1.80 (s, 6H), 3.97 (s, 3H), 4.02 (s, 3H), 6.50 (d, J = 2.4 Hz, 1H), 6.79 (d, J = 2.4 Hz, 1H), 7.33 (d, J = 8.4 Hz, 1H), 7.34 (td, J = 7.2, 1.2 Hz, 1H), 7.52 (td, J = 7.2, 1.2 Hz, 1H), 7.73 (s, 1H), 8.10 (dd, J = 8.4, 1.2 Hz, 1H), 15.92 (br s, 1H); ^{13}C NMR ($CDCl_3$, 100 MHz): δ 33.8, 39.8, 55.6, 56.4, 96.9, 104.0, 107.1, 108.1, 112.6, 115.3, 117.3, 117.7, 123.5, 124.2, 131.8, 140.8, 152.5, 154.9, 156.8, 157.5, 163.9, 165.3, 167.6, 189.9; HRMS (ESI) m/z: $[M + Na]^+$ calcd. for $C_{25}H_{20}NO_6Na$ 439.1152, found 439.1161.

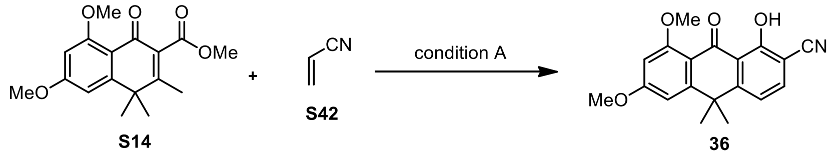

1-Hydroxy-6,8-dimethoxy-10,10-dimethyl-9-oxo-9,10-dihydroanthracene-2-carbonitrile (36). Following the general procedure of condition A, product **36** (68 mg, 42% yield) was prepared from donor **S14** (152 mg, 0.50 mmol) and acrylonitrile (**S42**) (29 mg, 0.55 mmol) as a yellow solid: mp = 237–239 °C; IR (CH_2Cl_2 cast, cm^{-1}) ν_{max} 2980, 2920, 2849, 2226, 1626, 1600, 1564; ^1H NMR ($CDCl_3$, 400 MHz): δ 1.69 (s, 6H), 3.95 (s, 3H), 4.02 (s, 3H), 6.50 (d, J = 2.2 Hz, 1H), 6.75 (d, J = 2.2 Hz, 1H), 7.10 (d, J = 8.4 Hz, 1H), 7.70 (d, J = 8.4 Hz, 1H), 14.79 (s, 1H); ^{13}C NMR ($CDCl_3$, 100 MHz): δ 33.6, 39.5, 55.6, 56.4, 96.9, 99.3, 104.2, 112.1, 115.9, 116.5, 137.6, 155.5, 155.6, 164.0, 165.4, 165.5, 187.1; HRMS (ESI) m/z: $[M + Na]^+$ calcd. for $C_{19}H_{17}NO_4Na$ 346.1050, found 346.1045.

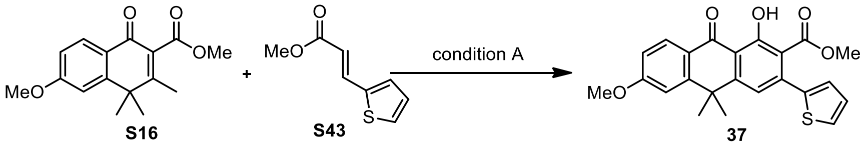

Methyl
1-hydroxy-6-methoxy-10,10-dimethyl-9-oxo-3-(thiophen-2-yl)-9,10-dihydroanthracene-2

-carboxylate (37). Following the general procedure of condition A, product **37** (149 mg, 73% yield) was prepared from donor **S16** (137 mg, 0.50 mmol) and acceptor **S43** (93 mg, 0.55 mmol) as a yellow solid: mp = 147–148 °C; IR (CH_2Cl_2 cast, cm^{-1}) ν_{max} 2976, 2947, 2842, 1732, 1626, 1593; ^{1}H NMR ($CDCl_3$, 400 MHz): δ 1.73 (s, 6H), 3.86 (s, 3H), 3.95 (s, 3H), 7.01 (dd, J = 8.8, 2.4 Hz, 1H), 7.10 (d, J = 2.4 Hz, 1H), 7.11 (dd, J = 5.2, 3.6 Hz, 1H), 7.23 (s, 1H), 7.27 (dd, J = 3.6, 1.2 Hz, 1H), 7.43 (dd, J = 5.2, 1.2 Hz, 1H), 8.33 (d, J = 8.8 Hz, 1H), 14.14 (s, 1H); ^{13}C NMR ($CDCl_3$, 100 MHz): δ 33.3, 38.6, 52.6, 55.6, 111.7, 113.2, 113.5, 118.1, 119.7, 122.4, 127.1, 127.3, 128.0, 129.9, 138.6, 140.8, 153.0, 153.2, 160.7, 164.7, 167.7, 187.2; HRMS (ESI) m/z: $[M + Na]^+$ calcd. for $C_{23}H_{20}O_5SNa$ 431.0924, found 431.0925.

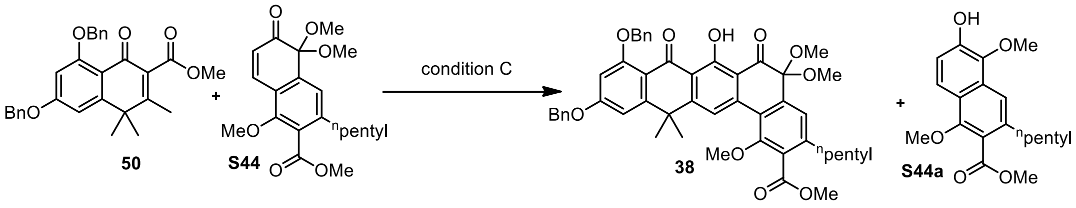

Methyl 9,11-bis(benzyloxy)-7-hydroxy-1,5,5-trimethoxy-13,13-dimethyl-6,8-dioxo-3-pentyl-5,6,8,13-tetrahydrobenzo[*a*]tetracene-2-carboxylate (38). Following the general procedure of condition C, product **38** (240 mg, 61% yield) was prepared from donor **50** (228 mg, 0.50 mmol) and acceptor **S44** (380 mg, 1.05 mmol) as a yellow solid: mp = 91–94 °C; IR (CH_2Cl_2 cast, cm^{-1}) ν_{max} 3064, 3032, 2934, 2862, 1731, 1623, 1595; ^{1}H NMR ($CDCl_3$, 400 MHz): δ 0.90 (t, J = 6.8 Hz, 3H), 1.32-1.36 (m, 4H), 1.40-1.65 (m, 2H), 1.71 (s, 6H), 2.59 (t, J = 8.0 Hz, 2H), 3.40 (s, 6H), 3.57 (s, 3H), 3.98 (s, 3H), 5.14 (s, 2H), 5.26 (s, 2H), 6.60 (d, J = 2.4 Hz, 1H), 6.83 (d, J = 2.4 Hz, 1H), 7.29-7.46 (m, 8H), 7.53 (s, 1H), 7.58 (d, J = 6.8 Hz, 2H), 8.18 (s, 1H), 15.07 (s, 1H); ^{13}C NMR ($CDCl_3$, 100 MHz): δ 13.9, 22.2, 30.4, 31.5, 33.2, 33.4, 39.5, 51.6, 52.3, 61.8, 70.2, 70.7, 97.5, 99.4, 105.4, 113.1, 115.6, 115.8, 116.8, 123.1, 124.9, 126.4, 127.5, 127.6, 128.3, 128.4, 128.6, 131.3, 135.6, 136.2, 139.7, 142.0, 155.1, 155.1, 155.4, 161.8, 162.3, 163.9, 168.1, 186.9, 193.9; HRMS (ESI) m/z: $[M + Na]^+$ calcd. for $C_{48}H_{48}O_{10}Na$ 807.3145, found 807.3152. Byproduct **S44a** (80 mg, 48%) was also isolated as a yellow oil: IR (CH_2Cl_2 cast, cm^{-1}) ν_{max} 3417 (br), 2952, 2933, 2859, 1729, 1626, 1605, 1501; ^{1}H NMR ($CDCl_3$, 300 MHz): δ 0.90 (t, J = 6.9 Hz, 3H), 1.34-1.39 (m, 4H), 1.67 (quint., J = 7.8 Hz, 2H), 2.75 (t, J = 7.8 Hz, 2H), 3.94 (s, 3H), 3.97 (s, 3H), 3.98 (s, 3H), 5.98 (br s, 1H), 7.20 (d, J = 9.0 Hz, 1H), 7.52 (s, 1H), 7.80 (d, J = 9.0 Hz, 1H); ^{13}C NMR ($CDCl_3$, 75 MHz): δ 13.9, 22.4, 30.6, 31.6, 33.9, 52.2, 61.5, 63.4, 115.9, 117.4, 119.9, 121.9, 122.5, 129.9, 138.4, 139.5, 146.7, 154.4, 169.0; HRMS (ESI) m/z: $[M + Na]^+$ calcd. for $C_{19}H_{24}O_5Na$ 355.1516, found: 355.1513.

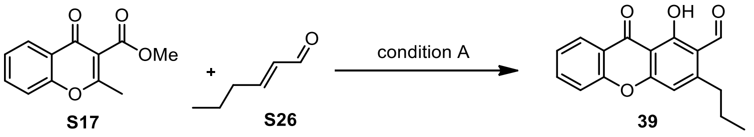

1-Hydroxy-9-oxo-3-propyl-9*H*-xanthene-2-carbaldehyde (39). Following the general procedure of condition A, product **39** (72 mg, 51% yield) was prepared from donor **S17** (109 mg, 0.50 mmol) and (*E*)-hex-2-enal (**S26**) (54 mg, 0.55 mmol) as a light yellow solid: mp = 162–163 °C; IR (CH_2Cl_2 cast, cm^{-1}) ν_{max} 3061, 2960, 2930, 2898, 2870, 1684, 1627, 1608, 1557; ^1H NMR ($CDCl_3$, 400 MHz): δ 1.03 (t, *J* = 7.2 Hz, 3H), 1.65 (sextet, *J* = 7.2 Hz, 2H), 3.08 (t, *J* = 7.2 Hz, 2H), 6.78 (s, 1H), 7.45 (td, *J* = 8.4, 1.2 Hz, 1H), 7.50 (dd, *J* = 8.4, 1.2 Hz, 1H), 7.80 (td, *J* = 8.0, 1.6 Hz, 1H), 8.28 (dd, *J* = 8.0, 1.6 Hz, 1H), 10.65 (s, 1H), 13.66 (s, 1H); ^{13}C NMR ($CDCl_3$, 100 MHz): δ 14.1, 23.9, 36.9, 107.0, 109.4, 116.4, 118.0, 120.6, 124.9, 126.0, 136.0, 155.9, 156.0, 158.8, 167.9, 181.7, 189.2; HRMS (ESI) m/z: $[M + Na]^+$ calcd. for $C_{17}H_{14}O_4Na$ 305.0784, found 305.0781.

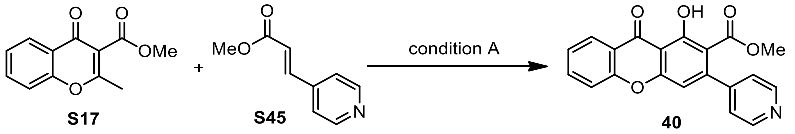

Methyl 1-hydroxy-9-oxo-3-(pyridin-4-yl)-9*H*-xanthene-2-carboxylate (40). Following the general procedure of condition A, product **40** (116 mg, 67% yield) was prepared from donor **S17** (109 mg, 0.50 mmol) and acceptor **S45** (90 mg, 0.55 mmol) as a light yellow solid: mp = 205–208 °C; IR (CH_2Cl_2 cast , cm^{-1}) ν_{max} 3080, 2922, 2851, 1721, 1638, 1611, 1599; ^1H NMR ($CDCl_3$, 400 MHz): δ 3.69 (s, 3H), 6.97 (s, 1H), 7.36 (d, *J* = 6.0 Hz, 2H), 7.48 (t, *J* = 8.0 Hz, 1H) 7.53 (d, *J* = 8.0 Hz, 1H), 7.83 (td, *J* = 8.0, 1.6 Hz, 1H), 8.32 (dd, *J* = 8.0, 1.6 Hz, 1H), 8.71 (br s, 2H), 13.36 (brs, 1H); ^{13}C NMR ($CDCl_3$, 100 MHz): δ 52.4, 108.0, 108.1, 115.4, 117.9, 120.4, 122.5, 124.8, 126.0, 136.2, 146.1, 147.0, 150.0, 156.0, 156.4, 160.3, 166.1, 181.8; HRMS (ESI) m/z: $[M + H]^+$ calcd. for $C_{20}H_{14}NO_5$ 348.0866, found 348.0880.

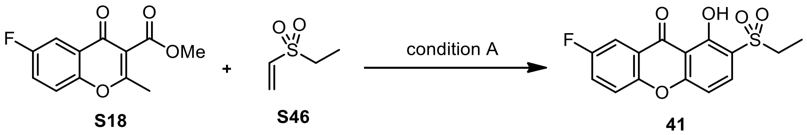

2-(Ethylsulfonyl)-7-fluoro-1-hydroxy-9*H*-xanthen-9-one (41). Following the general procedure of condition A, product **41** (106 mg, 66% yield) was prepared from donor **S18** (118 mg, 0.50 mmol) and ethyl vinyl sulfone (**S46**) (66 mg, 0.55 mmol) as a light yellow solid: mp = 213–215 °C; IR (CH_2Cl_2 cast, cm^{-1}) ν_{max} 3080, 2936, 1642, 1606, 1589, 1485; ^1H NMR ($CDCl_3$, 400 MHz): δ 1.31 (t, *J* = 7.2 Hz, 3H), 3.49 (q, *J* = 7.2 Hz, 2H), 7.10 (d, *J* = 8.8 Hz, 1H), 7.57-7.59 (m, 2H), 7.95 (dt, *J* = 7.6, 1.6 Hz, 1H), 8.27 (d, *J* = 8.8 Hz, 1H), 13.88 (s, 1H); ^{13}C NMR ($CDCl_3$, 100 MHz): δ 7.1, 48.7, 107.8, 108.3, 111.0 (d, $J_{C\text{-}F}$ = 23.6 Hz), 119.1, 120.3 (d, $J_{C\text{-}F}$ = 8.4 Hz), 121.2 (d, $J_{C\text{-}F}$ = 7.6 Hz), 124.9 (d, $J_{C\text{-}F}$ = 25.1 Hz), 137.5, 152.3, 159.3 (d,

$J_{C\text{-}F}$ = 247.0 Hz), 159.4, 161.1, 181.7 (d, $J_{C\text{-}F}$ = 2.3 Hz); HRMS (ESI) m/z: $[M + Na]^+$ calcd. for $C_{15}H_{11}O_5FSNa$ 345.0203, found 345.0209.

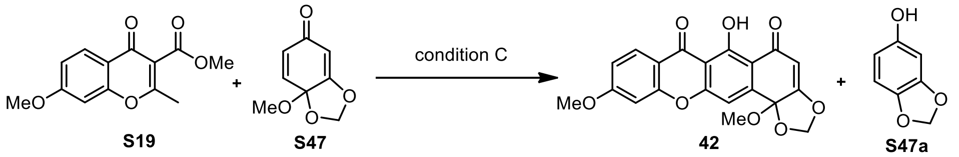

6-Hydroxy-10,13b-dimethoxy-5*H*-[1,3]dioxolo[4',5':3,4]benzo[1,2-*b*]xanthene-5,7(13b*H*)-dione (42). Following the general procedure of condition C, product **42** (138 mg, 72% yield) was prepared from donor **S19** (124 mg, 0.50 mmol) and acceptor **S47** (177 mg, 1.05 mmol) as a yellow solid: mp = 238–241 °C; IR (KBr, cm^{-1}) ν_{max} 3078, 2980, 2917, 2839, 1682, 1666, 1622, 1615; ^{1}H NMR ($CDCl_3$, 400 MHz): δ 3.24 (s, 3H), 3.95 (s, 3H), 5.75 (s, 1H), 5.76 (s, 1H), 5.76 (s, 1H), 6.88 (d, J = 2.4 Hz, 1H), 6.99 (dd, J = 9.2, 2.4 Hz, 1H), 7.13 (s, 1H), 8.22 (d, J = 9.2 Hz, 1H), 14.4 (s, 1H); ^{13}C NMR ($CDCl_3$/methanol-d_4 = 5/1, 100 MHz): δ 50.1, 55.6, 98.0, 99.0, 99.8, 105.3, 110.3, 114.1, 114.9, 127.5, 139.5, 157.1, 158.6, 163.4, 165.6, 167.3, 179.9, 187.8; HRMS (ESI) m/z: $[M + Na]^+$ calcd. for $C_{20}H_{14}O_8Na$ 405.0581, found 405.0600. Byproduct **S47a** (46 mg, 66%), commercially available, was also isolated as a white solid.

Scope of vinylogous donors and 1,2-dipolar acceptors:

General procedure for condition D:

To a stirred solution of vinylogous donor (0.50 mmol), 1,2-dipolar acceptor (0.55 mmol, 1.1 equiv.) and ZnI_2 (32 mg, 0.10 mmol, 0.2 equiv.) in dry toluene (2.0 mL) at 0 °C was added LiHMDS (1 M in toluene, 0.55 mL, 1.1 equiv.) dropwise under N_2. The mixture was then allowed to react at the indicated temperature and time as listed in Table 3 under dry air. After the reaction was complete, sat. $NH_4Cl_{(aq)}$ (5 mL) was added to quench the reaction. The organic layer was separated, and the aqueous layer was extracted with EtOAc (20 mL). The organic portions were combined, washed with water and brine, dried over $MgSO_4$, filtered and concentrated to give the crude residue, which was purified by chromatography on silical gel to afford the annulated product.

General procedure for condition E:

To a stirred solution of vinylogous donor (0.50 mmol), 1,2-dipolar acceptor (0.55 mmol, 1.1 equiv.) and ZnI_2 (32 mg, 0.10 mmol, 0.2 equiv.) in dry THF (2.0 mL) at 0 °C was added LiHMDS (1 M in THF, 0.55 mL, 1.1 equiv.) dropwise under N_2. The mixture was then allowed to react at the indicated temperature and time as listed in Table 3 under dry air. After the reaction was complete, sat. $NH_4Cl_{(aq)}$ (5 mL) was added to quench the reaction. The

organic layer was separated, and the aqueous layer was extracted with EtOAc (20 mL). The organic portions were combined, washed with water and brine, dried over $MgSO_4$, filtered and concentrated to give the crude residue, which was purified by chromatography on silical gel to afford the desired annulated product.

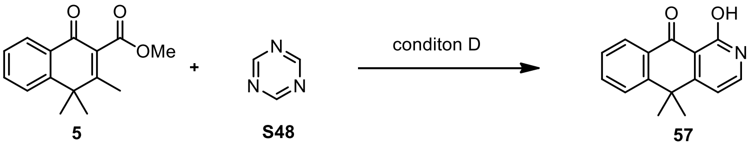

1-Hydroxy-5,5-dimethylbenzo[*g*]isoquinolin-10(5*H*)-one (57). Following the general procedure of condition D, product **57** (103 mg, 86% yield) was prepared from donor **5** (122 mg, 0.50 mmol) and 1,3,5-triazine (**S48**) (45 mg, 0.55 mmol) as a white solid: mp = 203–205 °C; IR (KBr, cm^{-1}) ν_{max} 3100, 3026, 3004, 2979, 2932, 2869, 2801, 1682, 1617, 1584; ^{1}H NMR (DMSO-d_6, 400 MHz): δ 1.63 (s, 6H), 6.75 (br s, 1H), 7.45 (t, *J* = 8.0 Hz, 1H), 7.67 (t, *J* = 8.0 Hz, 1H), 7.82 (d, *J* = 8.0 Hz, 1H), 8.05 (d, *J* = 8.0 Hz, 1H), 11.91 (br s, 1H), 13.57 (br s, 1H); ^{13}C NMR (DMSO-d_6, 175 MHz): δ 31.0, 38.1, 104.3, 117.6, 125.9, 126.9, 130.3, 133.0, 141.0, 148.2, 159.9, 166.8, 180.9; HRMS (ESI) m/z: $[M + Na]^+$ calcd. for $C_{15}H_{13}NO_2Na$ 262.0838, found 262.0840.

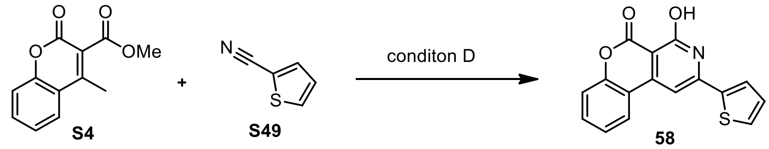

4-Hydroxy-2-(thiophen-2-yl)-5*H*-chromeno[3,4-*c*]pyridin-5-one (58). Following the general procedure of condition D, product **58** (117 mg, 79% yield) was prepared from donor **S4** (109 mg, 0.50 mmol) and 2-cyanothiophene (**S49**) (60 mg, 0.55 mmol) as a light yellow solid: mp = 281–284 °C; IR (KBr, cm^{-1}) ν_{max} 3078, 2924, 2849, 1753, 1632, 1612, 1590; ^{1}H NMR (DMSO-d_6, 400 MHz): δ 7.29 (dd, *J* = 4.8, 3.6 Hz, 1H), 7.43 (t, *J* = 8.6 Hz, 1H), 7.45 (t, *J* = 7.6 Hz, 1H), 7.71 (td, *J* = 8.6, 1.6 Hz, 1H), 7.91 (d, *J* = 4.8 Hz, 1H), 8.20 (d, *J* = 3.6 Hz, 1H), 8.49 (d, *J* = 7.6 Hz, 1H), 12.19 (br s, 1H); HRMS (ESI) m/z: $[M + Na]^+$ calcd. for $C_{16}H_9NO_3SNa$ 318.0195, found 318.0206.

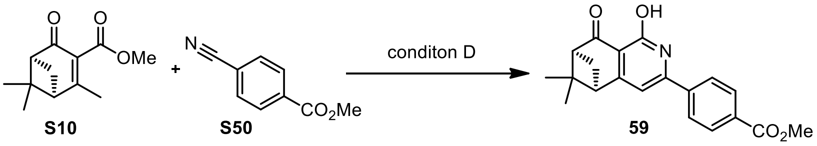

Methyl

4-((5*R*,7*S*)-1-hydroxy-6,6-dimethyl-8-oxo-5,6,7,8-tetrahydro-5,7-methanoisoquinolin-3-yl

)benzoate (59). Following the general procedure of condition D, product **59** (137 mg, 81% yield) was prepared from donor **S10** (104 mg, 0.50 mmol) and methyl 4-cyanobenzoate (**S50**) (89 mg, 0.55 mmol) as a yellow solid: mp = 193–195 °C; IR (CH_2Cl_2 cast, cm^{-1}) ν_{max} 3080, 2953, 2872, 1723, 1703, 1655, 1611; ^{1}H NMR ($CDCl_3$, 400 MHz): δ 0.91 (s, 3H), 1.64 (s, 3H), 2.23 (d, J = 9.6 Hz, 1H), 2.95 (t, J = 5.6 Hz, 1H), 3.10 (dt, J = 9.6, 5.6 Hz, 1H), 3.14 (t, J = 5.6 Hz, 1H), 3.95 (s, 3H), 7.25 (s, 1H), 8.14 (s, 4H), 11.27 (br s, 1H); ^{13}C NMR ($CDCl_3$, 100 MHz): δ 22.5, 26.5, 39.4, 48.4, 52.2, 52.7, 57.2, 108.9, 110.8, 127.5, 129.9, 131.5, 141.1, 159.1, 162.4, 163.7, 166.5, 204.5; HRMS (ESI) m/z: [M + Na]$^+$ calcd. for $C_{20}H_{19}NO_4Na$ 360.1206, found 360.1219.

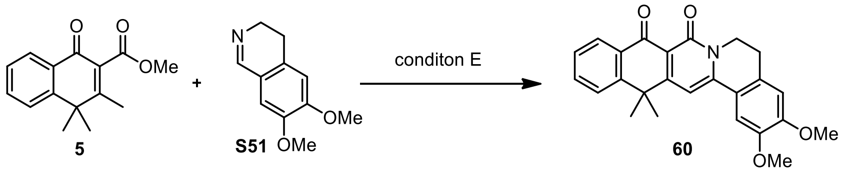

2,3-Dimethoxy-14,14-dimethyl-5*H*-benzo[*g*]isoquinolino[2,1-*b*]isoquinoline-8,9(6*H*,14*H*)-dione (60). Following the general procedure of condition E, product **60** (155 mg, 77% yield) was prepared from donor **5** (122 mg, 0.50 mmol) and imine **S51** (105 mg, 0.55 mmol) as a yellow solid: mp = 260–262 °C; IR (CH_2Cl_2 cast, cm^{-1}) ν_{max} 3061, 2975, 2933, 1681, 1609, 1593, 1574, 1509; ^{1}H NMR ($CDCl_3$, 400 MHz): δ 1.75 (s, 6H), 2.96 (t, J = 6.4 Hz, 2H), 3.98 (s, 3H), 4.02 (s, 3H), 4.35 (t, J = 6.4 Hz, 2H), 6.73 (s, 1H), 6.80 (s, 1H), 7.25 (s, 1H), 7.39-7.44 (m, 1H), 7.58-7.60 (m, 1H), 8.32 (d, J = 8.0 Hz, 1H); ^{13}C NMR ($CDCl_3$, 75 MHz): δ 27.2, 31.5, 38.1, 38.8, 55.9, 56.3, 99.1, 108.6, 110.5, 116.2, 120.4, 125.5, 126.6, 127.0, 130.4, 131.4, 132.2, 146.8, 147.1, 148.4, 152.0, 159.2, 164.4, 180.8; HRMS (ESI) m/z: [M + Na]$^+$ calcd. for $C_{25}H_{23}NO_4Na$ 424.1519, found 424.1529.

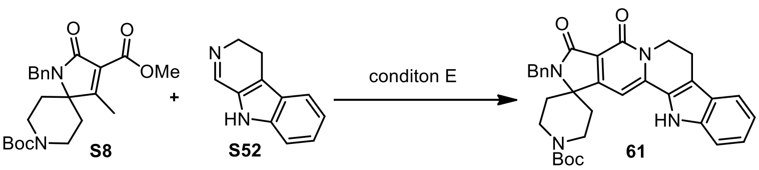

***tert*-Butyl**

2-benzyl-3,4-dioxo-2,3,4,6,7,12-hexahydrospiro[indolo[2,3-*a*]pyrrolo[3,4-*g*]quinolizine-1,4'-piperidine]-1'-carboxylate (61). Following the general procedure of condition E, product **61** (157 mg, 57% yield) was prepared from donor **S8** (207 mg, 0.50 mmol) and imine **S52** (94 mg, 0.55 mmol) as a yellow solid: mp = 243–246 °C; IR (CH_2Cl_2 cast, cm^{-1}) ν_{max} 3252 (br), 3062, 2974, 2927, 1694, 1640, 1571, 1549; ^{1}H NMR ($CDCl_3$, 400 MHz): δ 1.39 (s, 9H), 1.53 (d, J = 12.8 Hz, 2H), 2.00 (td, J = 12.8, 4.8 Hz, 2H), 3.14 (t, J = 7.2 Hz, 2H), 3.52 (t, J = 4.8 Hz, 2H), 4.01 (br s, 2H), 4.52 (t, J = 7.2 Hz, 2H), 4.65 (s, 2H), 7.14-7.33 (m, 8H), 7.21 (s, 1H), 7.60 (d, J = 8.0 Hz, 1H), 10.85 (s, 1H); ^{13}C NMR ($CDCl_3$, 100 MHz): δ 19.6, 28.2, 32.2, 32.8,

39.4, 40.3, 42.0, 62.9, 80.1, 97.1, 112.2, 113.3, 116.0, 119.7, 120.0, 124.9, 125.2, 127.0, 127.7, 128.4, 138.4, 139.3, 142.6, 154.7, 158.4, 164.3, 166.8; HRMS (ESI) m/z: $[M + Na]^+$ calcd. for $C_{33}H_{34}N_4O_4Na$ 573.2472, found 573.2484.

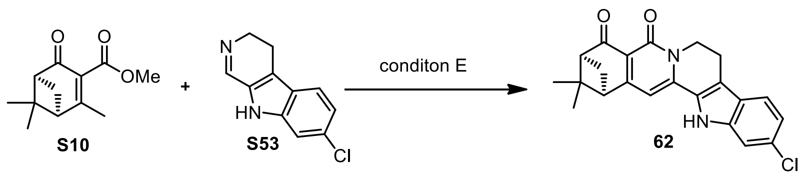

(1*R*,3*S*)-11-Chloro-2,2-dimethyl-2,3,7,8-tetrahydro-1,3-methanoindolo[2',3':3,4]pyrido[1,2-*b*]isoquinoline-4,5(1*H*,13*H*)-dione (62). Following the general procedure of condition E, product **62** (97 mg, 51% yield) was prepared from donor **S10** (104 mg, 0.50 mmol) and imine **S53** (113 mg, 0.55 mmol) as a yellow solid: mp > 300 °C; IR (KBr, cm^{-1}) ν_{max} 3226 (br), 3064, 2929, 2870, 1694, 1608, 1584, 1568, 1551; ^{1}H NMR (DMSO-d_6, 300 MHz): δ 0.87 (s, 3H), 1.50 (s, 3H), 1.98 (d, J = 8.4 Hz, 1H), 2.59 (t, J = 5.6 Hz, 1H), 2.90 (t, J = 5.6 Hz, 1H), 2.90 (dt, J = 8.4, 5.6 Hz, 1H), 3.09 (t, J = 7.5 Hz, 2H), 4.21 (dt, J = 14.1, 7.5 Hz, 1H), 4.38 (dt, J = 14.1, 7.5 Hz, 1H), 6.74 (s, 1H), 7.13 (dd, J = 8.7, 1.8 Hz, 1H), 7.50 (d, J = 1.8 Hz, 1H), 7.67 (d, J = 8.7 Hz, 1H), 12.09 (br s, 1H); ^{13}C NMR (DMSO-d_6, 75 MHz): δ 18.8, 22.1, 25.9, 37.1, 48.7, 50.1, 57.9, 99.4, 111.7, 112.6, 116.9, 120.6, 121.6, 123.7, 128.0, 129.6, 139.2, 141.8, 157.4, 165.6, 196.4; HRMS (ESI) m/z: $[M + Na]^+$ calcd. for $C_{22}H_{19}N_2O_2ClNa$ 401.1027, found 401.1031.

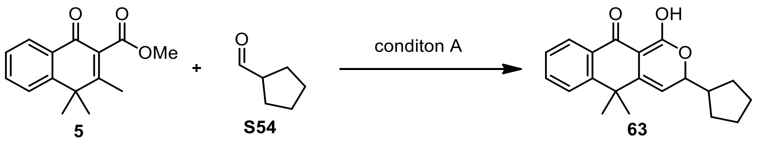

3-Cyclopentyl-1-hydroxy-5,5-dimethyl-3*H*-benzo[*g*]isochromen-10(5*H*)-one (63). Following the general procedure of condition A, product **63** (48 mg, 31% yield) was prepared from donor **5** (122 mg, 0.50 mmol) and cyclopentanecarboxaldehyde (**S54**) (54 mg, 0.55 mmol) as a yellow oil; IR (CH_2Cl_2 cast, cm^{-1}) ν_{max} 3443 (br), 3067, 2955, 2869, 1737, 1650, 1625; ^{1}H NMR ($CDCl_3$, 400 MHz): δ 1.41-1.92 (m, 8H), 1.45 (s, 3H), 1.49 (s, 3H), 2.21 (quint. d, J = 8.4, 6.4 Hz, 1H), 5.01 (dd, J = 6.4, 3.6 Hz, 1H), 5.48 (d, J = 3.6 Hz, 1H), 7.29-7.33 (m, 1H), 7.41-7.44 (m, 2H), 7.94 (d, J = 7.6 Hz, 1H), 12.96 (s, 1H); ^{13}C NMR ($CDCl_3$, 100 MHz): δ 25.4, 25.6, 27.9, 28.0, 30.7, 31.8, 37.9, 46.3, 83.9, 93.8, 111.9, 124.7, 124.7, 126.5, 126.7, 131.5, 139.5, 147.1, 161.4, 171.7; HRMS (ESI) m/z: $[M + Na]^+$ calcd. for $C_{20}H_{22}O_3Na$ 333.1461, found 333.1456.

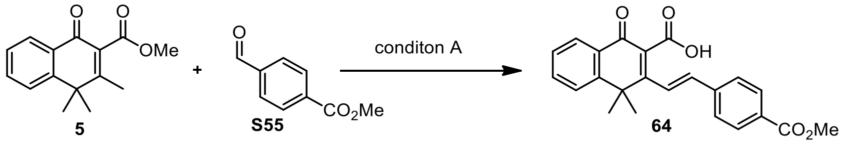

(*E*)-3-(4-(Methoxycarbonyl)styryl)-4,4-dimethyl-1-oxo-1,4-dihydronaphthalene-2-carboxylic acid (64). Following the general procedure of condition A, acid compound **64** (145 mg, 77% yield) was prepared from donor **5** (122 mg, 0.50 mmol) and methyl 4-formylbenzoate (**S55**) (90 mg, 0.55 mmol) as a white solid: mp > 300 °C; IR (KBr, cm^{-1}) ν_{max} 3130 (br), 2807, 1713, 1641, 1601, 1567; ^{1}H NMR (DMSO-d_6, 400 MHz): δ 1.56 (s, 6H), 3.89 (s, 3H), 7.28 (d, *J* = 16.4 Hz, 1H), 7.42 (t, *J* = 7.6 Hz, 1H), 7.42 (d, *J* = 16.4 Hz, 1H), 7.65 (td, *J* = 7.6, 1.2 Hz, 1H), 7.73 (d, *J* = 8.4 Hz, 2H), 7.81 (d, *J* = 7.6 Hz, 1H), 7.94 (d, *J* = 8.4 Hz, 2H); ^{13}C NMR (DMSO-d_6, 100 MHz): δ 29.3, 38.8, 52.3, 125.6, 126.6, 127.1, 127.5, 128.6, 128.9, 129.6, 132.9, 134.4, 134.5, 137.4, 141.5, 150.9, 152.0, 166.1, 170.4, 182.2; HRMS (ESI) m/z: $[M + H]^+$ calcd. for $C_{23}H_{21}O_5$ 377.1384, found 377.1382. Acid **64** was precipitaed out during the addition of sat. $NH_4Cl_{(aq)}$ to quench the reaction.

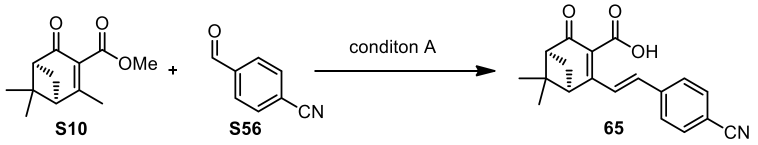

(1*R*,5*S*)-2-((*E*)-4-Cyanostyryl)-6,6-dimethyl-4-oxobicyclo[3.1.1]hept-2-ene-3-carboxylic acid (65). Following the general procedure of condition A, product **65** (129 mg, 84% yield) was prepared from donor **S10** (104 mg, 0.50 mmol) and 4-formylbenzonitrile (**S56**) (72 mg, 0.55 mmol) as a light yellow solid: mp > 300 °C; IR (KBr, cm^{-1}) ν_{max} 3118 (br), 3043, 2808, 2225, 1732, 1609; ^{1}H NMR (methanol-d_4, 400 MHz): δ 1.07 (s, 3H), 1.61 (s, 3H), 2.19 (d, *J* = 9.2 Hz, 1H), 2.71 (t, *J* = 5.6 Hz, 1H), 2.98 (dt, *J* = 9.2, 5.6 Hz, 1H), 3.33 (t, *J* = 5.6 Hz, 1H), 7.18 (d, *J* = 16.4 Hz, 1 H), 7.70 (d, *J* = 16.4 Hz, 1H), 7.72 (d, *J* = 8.8 Hz, 2H), 7.75 (d, *J* = 8.8 Hz, 2H); HRMS (ESI) m/z: $[M + H]^+$ calcd. for $C_{19}H_{18}NO_3$ 308.1281, found 308.1292. Acid **65** was precipitaed out during the addition of sat. $NH_4Cl_{(aq)}$ to quench the reaction. In addition, compound **65** was further transformed to its ester derivative **65-Et** as detailed as follows.

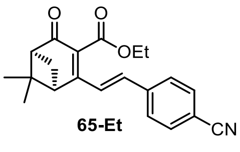

(1*R*,5*S*)-Ethyl 2-((*E*)-4-cyanostyryl)-6,6-dimethyl-4-oxobicyclo[3.1.1]hept-2-ene-3-carboxylate (65-Et). To a stirred solution of acid **65** (61 mg, 0.2 mmol) and K_2CO_3 (41 mg, 0.3 mmol) in dry DMF (1 mL) at room temperature was added EtI (47 mg, 0.3 mmol) dropwise under N_2. The

mixture was then allowed to react at room temperature for 1 h under N_2. After the reaction was complete, water (5 mL) was added to quench the reaction. The resulting mixture was extracted with EtOAc (10 mL × 2). The separated organic layer was washed with water (10 mL × 3) and brine, dried over $MgSO_4$, filtered and concentrated to give the crude residue, which was purified by chromatography on silical gel to afford ester **65-Et** (68 mg, 96% yield) as a yellow oil; IR (CH_2Cl_2 cast, cm^{-1}) ν_{max} 2977, 2226, 1729, 1674, 1616, 1559; ^1H NMR ($CDCl_3$, 300 MHz): δ 1.07 (s, 3H), 1.40 (t, J = 7.6 Hz, 3H), 1.60 (s, 3H), 2.19 (d, J = 9.6 Hz, 1H), 2.84 (t, J = 5.6 Hz, 1H), 2.94 (dt, J = 9.6, 5.4 Hz, 1H), 3.23 (t, J = 5.6 Hz, 1H), 4.41 (q, J = 7.6 Hz, 2H), 7.36 (d, J = 16.2 Hz, 1H), 7.48 (d, J = 16.2 Hz, 1H), 7.59 (d, J = 8.1 Hz, 2H), 7.66 (d, J = 8.1 Hz, 2H); ^{13}C NMR ($CDCl_3$, 75 MHz): δ 14.3, 21.8, 26.6, 38.3, 43.4, 52.2, 57.6, 61.4, 112.5, 118.4, 127.4, 127.5, 128.0, 132.6, 135.6, 140.0, 161.2, 165.2, 198.6; HRMS (ESI) m/z: $[M +Na]^+$ calcd. for $C_{21}H_{21}NO_3Na$ 358.1419, found 358.1424.

Synthetic procedures and characterization of Michael acceptors

Most of Michael acceptors and 1,2-dipolar acceptors were commercially available including **S20-S23**, **S24-S28**, **S30**, **S31**, **S36-S39**, **S42**, **S43**, **S45**, **S46** and **S54-S56**. The following known Michael acceptors and imines were prepared by reported procedures: **2**,[8] **43**,[9] **S29**,[10] **S32**,[8] **S33**,[11] **S34**,[11] **S35**,[12] **S40**,[13] **S47**,[12] **S52**[14] and **S53**.[14] The remaining Michael acceptors **S44**,[15] **S41** and **46** were prepared by following procedures:

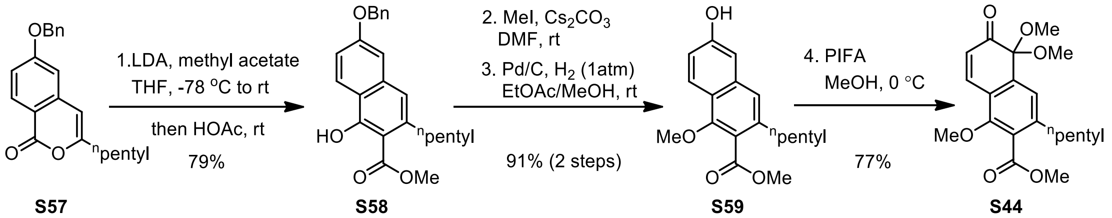

Methyl 6-(benzyloxy)-1-hydroxy-3-pentyl-2-naphthoate (S58). To a stirred solution of isocumarin **S57** (3.22 g, 10.0 mmol) and methyl acetate (1.11 g, 15.0 mmol) in dry THF (40 mL) at -78 °C was added fresh prepared LDA (2 M in THF, 0.55 mL, 1.1 equiv.) dropwise under N_2. The mixture was then allowed to react at 0 °C for 1 h under N_2. After the reaction was complete, sat. $NH_4Cl_{(aq)}$ (30 mL) was added to quench the reaction. The layers were separated, and the aqueous layer was extracted with EtOAc (30 mL × 2). The organic portions were combined, washed with water and brine, dried over $MgSO_4$, filtered and concentrated to give the undehydrated intermediate. The undehydrated intermediate was dissolved in HOAc (30 mL), and allowed to react at room temperature for 15 h under N_2 . After the reaction was complete, most of HOAc was evaporated and EtOAc (60 mL) was added to extract the reaction mixture. The organic portions were washed with sat. $NaHCO_{3(aq)}$, water and brine, dried over $MgSO_4$, filtered and concentrated to give the crude residue, which was purified by chromatography on silical gel to afford the naphthalene **S58** (2.99 g, 79%) as a white solid: mp = 89–91 °C; IR (CH_2Cl_2 cast, cm^{-1}) ν_{max} 3066, 3032, 2953, 2930, 2870, 1630, 1603, 1583; ^1H NMR ($CDCl_3$, 400 MHz): δ 0.90 (t, J = 6.8 Hz, 3H), 1.33-1.38 (m, 4H), 1.55-1.59 (m, 2H), 2.96 (t, J = 7.6 Hz, 2H), 3.98 (s, 3H), 5.17 (s, 2H), 6.98 (s, 1H), 7.02 (d, J = 2.4 Hz, 1H), 7.14 (dd, J = 9.2, 2.4 Hz, 1H), 7.32-7.49 (m, 5H), 8.27 (d, J = 9.2 Hz, 1H), 12.69 (s, 1H); ^{13}C NMR ($CDCl_3$, 100 MHz): δ 14.1, 22.6, 31.7, 32.1, 37.2, 52.0, 69.9, 104.1, 106.4, 117.4, 118.6, 119.4, 126.0, 127.5, 128.1, 128.6, 136.5, 138.0, 140.8, 159.9, 162.6, 172.9; HRMS (ESI) m/z: $[M + H]^+$ calcd. for $C_{24}H_{27}O_4$ 379.1904, found 379.1914.

Methyl 6-hydroxy-1-methoxy-3-pentyl-2-naphthoate (S59). To a stirred solution of naphthalene **S58** (2.99 g, 7.9 mmol) and Cs_2CO_3 (3.10 g, 9.5 mmol) in DMF (8 mL) at room temperature was added MeI (1.35 g, 9.5 mmol) dropwise under N_2. The reaction mixture was then allowed to react at room temperature for 2 h under N_2. After the reaction was complete, water (8 mL) was added to quench the reaction. The resulting reaction mixture was extracted with EtOAc (30 mL × 2). The separated organic layer was washed with water and brine, dried

over $MgSO_4$, filtered and concentrated to give the methylated intermediate. To a stirred solution of the methylated intermediate in EtOAc/MeOH (40 mL/8 mL) at room temperature was added 10% wet Pd/C (200 mg) portionwise under N_2. The mixture was then allowed to react at room temperature under 1 atm. H_2 for 15 h. After the reaction was complete, it was filtrated with celite and concentrated to give the crude residue, which was purified by chromatography on silical gel to afford 2-naphthol **S59** (2.17 g, 91% yield over 2 steps) as a white solid: mp = 82–83 °C; IR (CH_2Cl_2 cast, cm^{-1}) ν_{max} 3396 (br), 2954, 2932, 2858, 1728, 1706, 1631; ^1H NMR ($CDCl_3$, 400 MHz): δ 0.90 (t, J = 6.8 Hz, 3H), 1.26-1.36 (m, 4H), 1.73-1.79 (m, 2H), 2.69 (t, J = 8.0 Hz, 2H), 3.97 (s, 3H), 3.98 (s, 3H), 5.45 (brs, 1H), 7.06 (s, 1H), 7.07 (d, J = 9.6 Hz, 1H), 7.24 (s, 1H), 7.98 (d, J = 9.6 Hz, 1H); ^{13}C NMR ($CDCl_3$, 100 MHz): δ 14.0, 22.4, 30.6, 31.7, 33.7, 52.4, 63.4, 109.3, 117.8, 120.8, 122.1, 122.1, 124.4, 136.8, 138.2, 154.0, 155.1, 169.7; HRMS (ESI) m/z: $[M + H]^+$ calcd. for $C_{18}H_{23}O_4$ 303.1591, found 303.1592.

Methyl 1,5,5-trimethoxy-6-oxo-3-pentyl-5,6-dihydronaphthalene-2-carboxylate (S44). To a stirred solution of 2-naphthol **S59** (1.51 g, 5.0 mmol) in dry MeOH (25 mL) at 0 °C was added PIFA (4.73 g, 11.0 mmol) portionwise under N_2. The reaction mixture was then allowed to react at room temperature for 3 h under N_2. After the reaction was complete, sat. $NaHCO_{3(aq)}$ (25 mL) was added to quench the reaction, most of MeOH was evaporated and EtOAc (30 mL × 2) was added to extract the reaction mixture. The organic portions were washed with sat. $NaHCO_{3(aq)}$, water and brine, dried over $MgSO_4$, filtered and concentrated to give the crude residue, which was purified by chromatography on silical gel to afford the enone **S44** (1.40 g, 77% yield) as a yellow oil: IR (CH_2Cl_2 cast, cm^{-1}) ν_{max} 2952, 2871, 1734, 1682; ^1H NMR ($CDCl_3$, 400 MHz): δ 0.89 (t, J = 6.8 Hz, 3H), 1.31-1.34 (m, 4H), 1.58-1.63 (m, 2H), 2.60 (t, J = 8.0 Hz, 2H), 3.28 (s, 6H), 3.86 (s, 3H), 3.95 (s, 3H), 6.13 (d, J = 10.4 Hz, 1H), 7.37 (s, 1H), 7.61 (d, J = 10.4 Hz, 1H); ^{13}C NMR ($CDCl_3$, 100 MHz): δ 13.7, 22.1, 30.3, 31.3, 33.5, 51.6, 52.1, 63.9, 94.8, 122.6, 124.3, 124.6, 129.2, 136.7, 140.2, 143.8, 154.9, 167.4, 194.5; HRMS (ESI) m/z: $[M + Na]^+$ calcd. for $C_{20}H_{26}O_6Na$ 385.1627, found 385.1622.

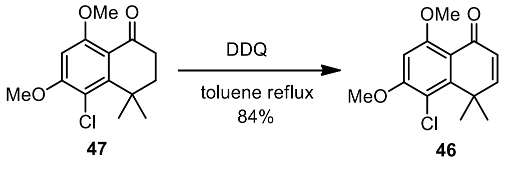

5-Chloro-6,8-dimethoxy-4,4-dimethylnaphthalen-1(4*H*)-one (46). To a stirred solution of tetralone **47** (2.68 g, 10.0 mmol) in dry toluene (50 mL) was added DDQ (2.38 g, 10.5 mmol) in one portion. The resulting mixture was then stirred at reflux temperature under N_2 for 6 h. After reaction was complete, the reaction mixture was filtrated with celite, and quenched with saturated $NaHCO_{3(aq)}$ (20 mL) and extracted with EtOAc (50 mL). The organic extract was washed with saturated $NaHCO_{3(aq)}$ and brine, dried over $MgSO_4$, filtered and concentrated to

give the crude residue, which was purified by chromatography on silical gel to afford enone **46** (2.24 g, 84% yield) as a white solid: mp = 106–107 °C; IR (CH_2Cl_2 cast, cm^{-1}) ν_{max} 3009, 2964, 2935, 1671, 1637, 1579; ^1H NMR ($CDCl_3$, 400 MHz) δ 1.71 (s, 6H), 3.96 (s, 3H), 3.99 (s, 3H), 6.15 (d, J = 10.4 Hz, 1H), 6.57 (s, 1H), 6.58 (d, J = 10.4 Hz, 1H); ^{13}C NMR ($CDCl_3$, 100 MHz) δ 24.9, 39.0, 56.2, 56.4, 95.5, 113.5, 115.7, 125.1, 148.9, 155.6, 159.1, 161.0, 184.2; HRMS (ESI) m/z: $[M + Na]^+$ calcd. for $C_{14}H_{15}ClO_3Na$ 289.0596, found 289.0602.

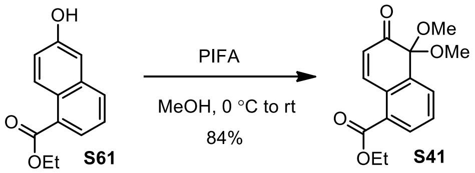

Ethyl 5,5-dimethoxy-6-oxo-5,6-dihydronaphthalene-1-carboxylate (S41). According to the synthetic procedures similar to compound **S44**, enone **S41** (1.63 g, 84% yield) was prepared from 2-naphthol **S61** (1.51 g, 7.0 mmol) as a yellow oil; IR (CH_2Cl_2 cast, cm^{-1}) ν_{max} 3090, 2982, 2942, 2834, 1717, 1684, 1588, 1570; ^1H NMR ($CDCl_3$, 400 MHz): δ 1.42 (t, J = 6.8 Hz, 3H), 3.27 (s, 6H), 4.41 (q, J = 6.8 Hz, 2H), 6.21 (d, J = 10.4 Hz, 1H), 7.50 (t, J = 7.6 Hz, 1H), 7.89 (dd, J = 7.6, 0.8 Hz, 1H), 7.94 (dd, J = 7.6, 0.8 Hz, 1H), 8.40 (d, J = 10.4 Hz, 1H); ^{13}C NMR ($CDCl_3$, 100 MHz): δ 14.1, 51.8, 61.6, 95.1, 126.2, 129.3, 130.1, 131.2, 131.3, 131.6, 139.5, 140.1, 166.4, 194.6; HRMS (ESI) m/z: $[M + Na]^+$ calcd. for $C_{15}H_{16}O_5Na$ 299.0890, found 299.0892.

Synthetic procedures and characterization of (±)-ABX

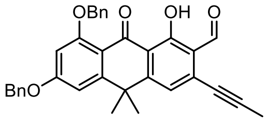

53

6,8-Bis(benzyloxy)-1-hydroxy-10,10-dimethyl-9-oxo-3-(prop-1-yn-1-yl)-9,10-dihydroanthracene-2-carbaldehyde (53). To a stirred solution of vinylogous donor **50** (1.37 g, 3.0 mmol) and aldehyde **2** (593 mg, 6.3 mmol) in dry toluene (11.4 mL) at 0 °C was added LiHMDS (1 M in toluene, 3.6 mL) dropwise under N_2. The mixture was then allowed to react at reflux temperature under dry air. After the reaction was complete, sat. $NH_4Cl_{(aq)}$ (10 mL) was added to quench the reaction. The layers were separated, and the aqueous layer was extracted with EtOAc (30 mL × 2). The organic portions were combined, washed with water and brine, dried over $MgSO_4$, filtered and concentrated to give the crude residue, which was purified by chromatography on silical gel to afford tricyclic aldehyde **53** (821 mg, 53% yield) as an orange solid: mp = 211–213 °C; IR (CH_2Cl_2 cast, cm^{-1}) ν_{max} 3064, 3032, 2978, 2929, 2864, 2632, 2225, 1691, 1621, 1592; ^1H NMR ($CDCl_3$, 400 MHz): δ 1.66 (s, 6H), 2.18 (s, 3H), 5.14 (s, 2H), 5.25 (s, 2H), 6.60 (d, J = 2.4 Hz, 1H), 6.81 (d, J = 2.4 Hz, 1H), 7.15 (s, 1H), 7.31-7.43 (m, 8H), 7.59 (d, J = 7.6 Hz, 2H), 10.62 (s, 1H), 15.28 (1H); ^{13}C NMR ($CDCl_3$, 100 MHz): δ 4.9, 33.3, 39.3, 70.3, 70.8, 77.9, 95.9, 99.3, 105.3, 112.9, 115.2, 121.5, 122.1, 126.5, 127.5, 127.8, 128.4, 128.5, 128.7, 131.4, 135.6, 136.2, 155.0, 155.2, 162.5, 164.1, 165.8, 187.1, 189.4; HRMS (ESI) m/z: $[M + H]^+$ calcd. for $C_{34}H_{29}O_5$ 517.2010, found 517.2032.

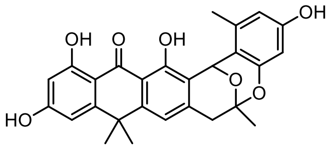

(±)-ABX

(±)-ABX.[16] To a stirred solution of compound **53** (258 mg, 0.5 mmol) and orcinol (124 mg, 1.0 mmol) in dry DCE (6 mL) at room temperature was added $PtCl_2$ (27 mg, 0.1 mmol). After degassed with argon for 30 mins, the reaction vial was sealed and then allowed to react at 150 °C under N_2 for 6 h. The reaction mixture was diluted with DCM (20 mL) and filtrated with celite. The filtrate was collected and concentrated to give intermediates **55** and **56** as 1:1 ratio as determined by crude ^1H-NMR. To a stirred solution of the mixture in EtOAc/MeOH (5 mL/1 mL) at room temperature was added 10% wet Pd/C (50 mg) portionwise under N_2. The mixture was then allowed to react at room temperature under 1 atm H_2 for 5 h. After the reaction was complete, it was filtrated with celite and concentrated to give the crude residue, which was purified by chromatography on silical gel to afford (±)-ABX (150 mg, 65% yield over 2 steps) as a yellow solid: mp = 243–246 °C; IR (KBr, cm^{-1}) ν_{max} 3370 (br), 2976, 2929,

1606; ^{1}H NMR (Acetone-d_6, 400 MHz): δ 1.60 (s, 3H), 1.64 (s, 3H), 1.68 (s, 3H), 2.44 (s, 3H), 3.13 (d, J = 18.0 Hz, 1H), 3.33 (d, J = 18.0 Hz, 1H), 6.15 (d, J = 2.4 Hz, 1H), 6.24 (s, 1H), 6.27 (d, J = 2.4 Hz, 1H), 6.32 (d, J = 2.2 Hz, 1H), 6.75 (d, J = 2.2 Hz, 1H), 7.15 (s, 1H), 12.78 (s, 1H), 13.47 (s, 1H); ^{13}C NMR (Acetone-d_6, 100 MHz): δ 19.6, 28.0, 34.1, 34.2, 39.6, 41.0, 66.1, 98.8, 101.9, 102.2, 107.5, 108.3, 111.1, 112.3, 115.0, 118.6, 124.7, 137.2, 143.2, 151.5, 153.6, 156.1, 158.1, 158.8, 166.5, 166.9, 192.0; HRMS (ESI) m/z: $[M + Na]^+$ calcd. for $C_{27}H_{24}O_7Na$ 483.1414, found 483.1436.

Table S2. ^{1}H and ^{13}C NMR data of synthetic (±)-ABX and natural ABX in acetone-d$_6$

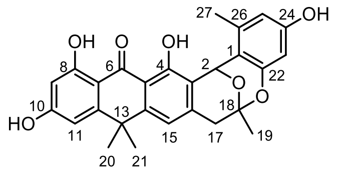

	Natural (400 MHz, acetone-d$_6$)[16c]		Synthetic (400 MHz, acetone-d$_6$)	
	δ_C	δ_H, mult. (J in Hz)	δ_C	δ_H, mult. (J in Hz)
1	113.8		113.9	
2[a]	65.0	6.24, s, 1H	65.0	6.24, s, 1H
3	123.6		123.7	
4	157.6		157.7	
5	111.3		111.3	
6	190.6		190.9	
7	107.1		107.2	
8	165.7		165.8	
9	101.2	6.31, d, (2.1), 1H	101.1	6.32, d (2.2), 1H
10	165.6		165.4	
11	106.5	6.74, d (2.1), 1H	106.4	6.75, d (2.2), 1H
12	154.8		155.1	
13	38.6		38.5	
14	150.3		150.5	
15	117.4	7.13, s, 1H	117.6	7.15, s, 1H
16	141.9		142.1	
17	40.1	3.13, d (18.4), 1H	40.0	3.13, d (18.0), 1H
		3.32, d (18.4), 1H		3.33, d (18.0), 1H
18	97.7		97.8	
19	27.1	1.64, s, 3H	27.0	1.64, s, 3H
20	33.2	1.67, s, 3H	33.0	1.68, s, 3H
21	33.3	1.60, s, 3H	33.2	1.60, s, 3H
22	152.4		152.6	
23	100.8	6.15, d, (2.5), 1H	100.8	6.15, d (2.4), 1H
24	157.0		157.1	
25	111.0	6.26, d (2.5), 1H	110.0	6.27, d (2.4), 1H
26	136.0		136.1	
27	18.7	2.44, s, 3H	18.5	2.44, s, 3H
4-OH		13.5, s, 1H		13.47, s, 1H
8-OH		12.8, s, 1H		12.78, s, 1H

[a] ^{13}C NMR spectral analyses are aligned and calibrated on the basis of C-2.

Table S3. ^{1}H and ^{13}C NMR data of synthetic (±)-ABX and natural ABX in methanol-d_4

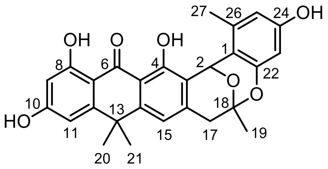

	Natural (400 MHz, methanol-d_4)[16d]		Synthetic (400 MHz, methanol-d_4)	
	δ_C	δ_H, mult. (J in Hz)	δ_C	δ_H, mult. (J in Hz)
1	113.6		113.7	
2[a]	65.2	6.18, s, 1H	65.2	6.17, s, 1H
3	123.7		123.6	
4	157.4		157.5	
5	100.7		100.7	
6	190.8		190.8	
7	106.1		106.1	
8	165.6		165.6	
9	106.9	6.19, d, (2.1), 1H	107.0	6.19, d (2.4), 1H
10	156.8		156.8	
11	111.2	6.58, d (2.1), 1H	111.2	6.58, d (2.4), 1H
12	154.8		154.8	
13	38.4		38.4	
14	150.6		150.5	
15	117.2	7.00, s, 1H	117.1	7.00, s, 1H
16	141.9		141.8	
17	39.8	3.03, d (18.4), 1H	39.8	3.03, d (18.4), 1H
		3.23, d (18.4), 1H		3.22, d (18.4), 1H
18	97.7		97.7	
19	29.4[b]	1.60, s, 3H	26.5	1.60, s, 3H
20	32.7	1.60, s, 3H	32.6	1.60, s, 3H
21	33.0	2.36, s, 3H	33.0	2.36, s, 3H
22	152.3		152.3	
23	100.5	6.17, d, (2.1), 1H	100.5	6.16, d (2.0), 1H
24	165.5		165.6	
25	109.8	6.05, d (2.1), 1H	109.8	6.06, d (2.0), 1H
26	136.1		136.1	
27	18.2	1.35,[b]s, 3H	18.2	1.53, s, 3H
4-OH				
8-OH				

[a] ^{13}C NMR spectral analyses are aligned and calibrated on the basis of C-2. [b]Original

signals for C-19 (^{13}C NMR) at δ 29.4 and the methyl protons on C-27 (^{1}H NMR) at δ 1.35 (s) are incorrectly assigned in methanol-d_4 by authors who isolated ABX natural product.[16d] After careful examination, we revise these signals as δ 26.5 and δ 1.53, respectively, as indicated above in the same *d*-solvent.

Reference

[1] Y.-J. Chen, C.-P. Chuang, *Synthesis* **2016**, *48*, 3603-3617.

[2] A. Carrër, J.-D. Brion, S. Messaoudi, M. Alami, *Adv. Synth. Catal.* **2013**, *355*, 2044-2054.

[3] a) A. Arcadi, F. Marinelli, L. Rossi, M. Verdecchia, *Synthesis* **2006**, *38*, 2019-2030; b) M. C. Nakhla, J. L. Wood, *J. Am. Chem. Soc.* **2017**, *139*, 18504-18507; c) M. C. Nakhla, K. N. Weeks, M. Navarro Villalobos, J. L. Wood, *Tetrahedron* **2018**, *74*, 5085-5088.

[4] M. Watanabe, T. Abe, N. Harada, *J. Org. Chem.* **1997**, *62*, 2992-2995.

[5] a) A. Arcadi, S. Cacchi, G. Fabrizi, F. Marinelli, *Synlett* **1993**, 65-68; b) S. Redon, G. Eucat, M. Ipuy, E. Jeanneau, I. Gautier-Luneau, A. Ibanez, C. Andraud, Y. Bretonnière, *Dyes Pigm.* **2018**, *156*, 116-132.

[6] G. M. Coppola, R. W. Dodsworth, *Synthesis* **1981**, *7*, 523-524.

[7] C. Bolchi, P. Catalano, L. Fumagalli, M. Gobbi, M. Pallavicini, A. Pedretti, L. Villa, G. Vistoli, E. Valoti, *Bioorg. Med. Chem.* **2004**, *12*, 4937-4951.

[8] a) E. Pavlakos, T. Georgiou, M. Tofi, T. Montagnon, G. Vassilikogiannakis, *Org. Lett.* **2009**, *11*, 4556-4559; b) J. D. Myrtle, A. M. Beekman, R. A. Barrow, *Org. Biomol. Chem.* **2016**, *14*, 8253-8260.

[9] S. K. Chittimalla, M. Koodalingam, V. Gadi, P. Anaspure, *Synlett* **2017**, *28*,475-480.

[10] H. N. Roy, M. S. Sarkar, D. Mal, *Synth. Commun.* **2005**, *35*, 2183-2188.

[11] J.-K. Huang, T.-L. Yang Lauderdale, C.-C. Lin, K.-S. Shia, *J. Org. Chem.* **2018**, *83*, 6508-6523.

[12] Y. Tamura, T. Yakura, J. Haruta, Y. Kita, *J. Org. Chem.* **1987**, *52*, 3927-3930.

[13] A. E. Fleck, J. A. Hobart, G. W. Morrow, *Synth. Commun.* **1992**, *22*, 179-187.

[14] N. Denizot, R. Guillot, C. Kouklovsky, G. Vincent, *Synthesis* **2018**, *50*, 4823-4828.

[15] F. M. Hauser, H. Liao, Y. Sun, *Org. Lett.* **2002**, *4*, 2241-2243.

[16] a) K. Kojiri, S. Nakajima, A. Fuse, H. Suzuki, H. Suda, *J. Antibiot.* **1995**, *48*, 1506-1508; b) Y. K. T. Lam, O. Hensens, G. Helms, D. Williams, M. Nallin, J. Smith, S. Gartner, L. H. Rodriguez, S. Stevensmiles, *Tetrahedron Lett.* **1995**, *36*, 2013-2016; c) H. Chen, N. Liu, Y. Huang, Y. Chen, *Tetrahedron: Asymmetry* **2014**, *25*, 113-116; d) X. Mei, X. Yan, H. Zhang, M. Yu, G. Shen, L. Zhou, Z. Deng, C. Lei, X. Qu, *ACS Chem. Biol.* **2018**, *13*, 200-206.

^{1}H and ^{13}C NMR spectra for all new compounds:

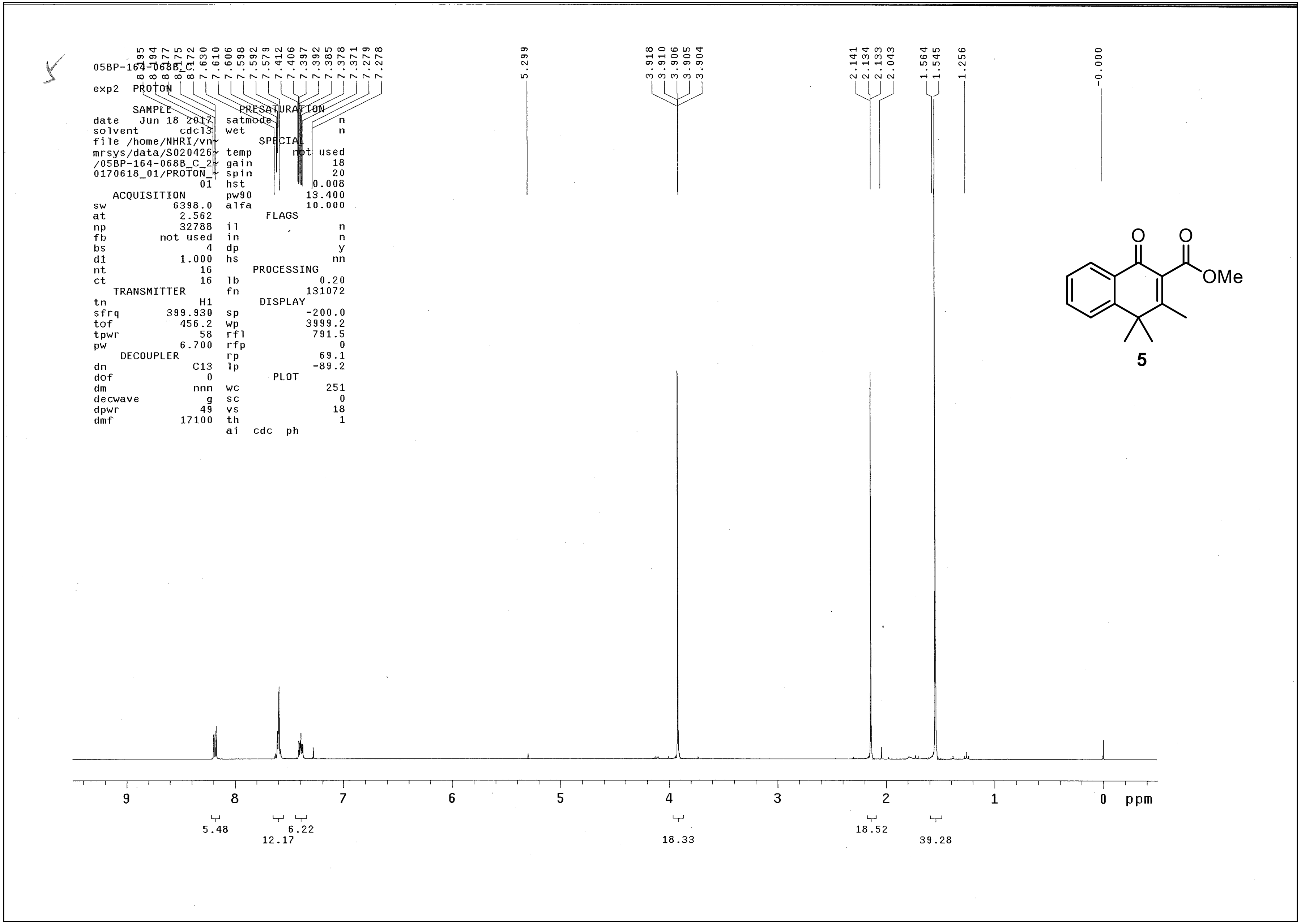

^{1}H NMR spectra for compound **5**

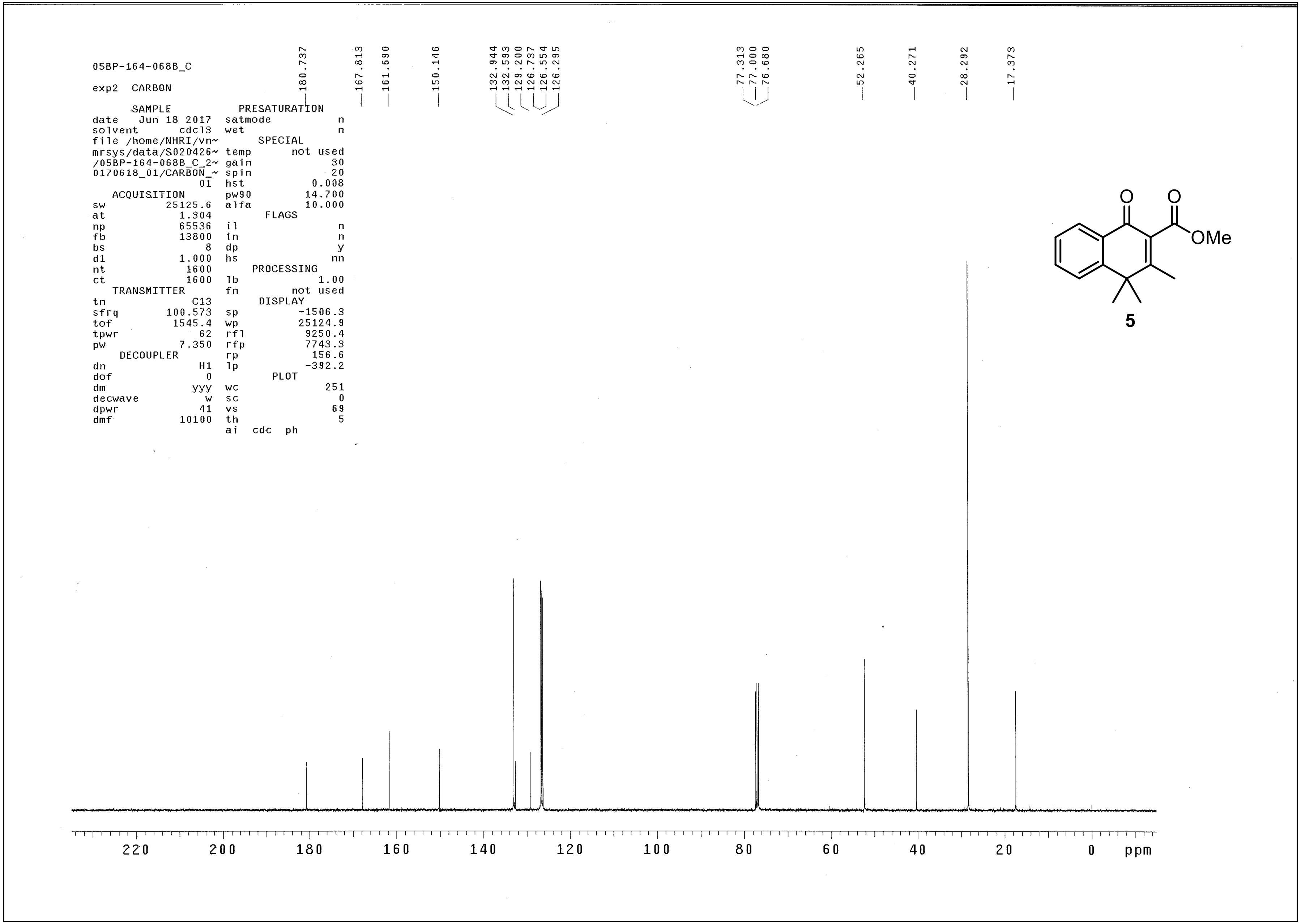

^{13}C NMR spectra for compound **5**

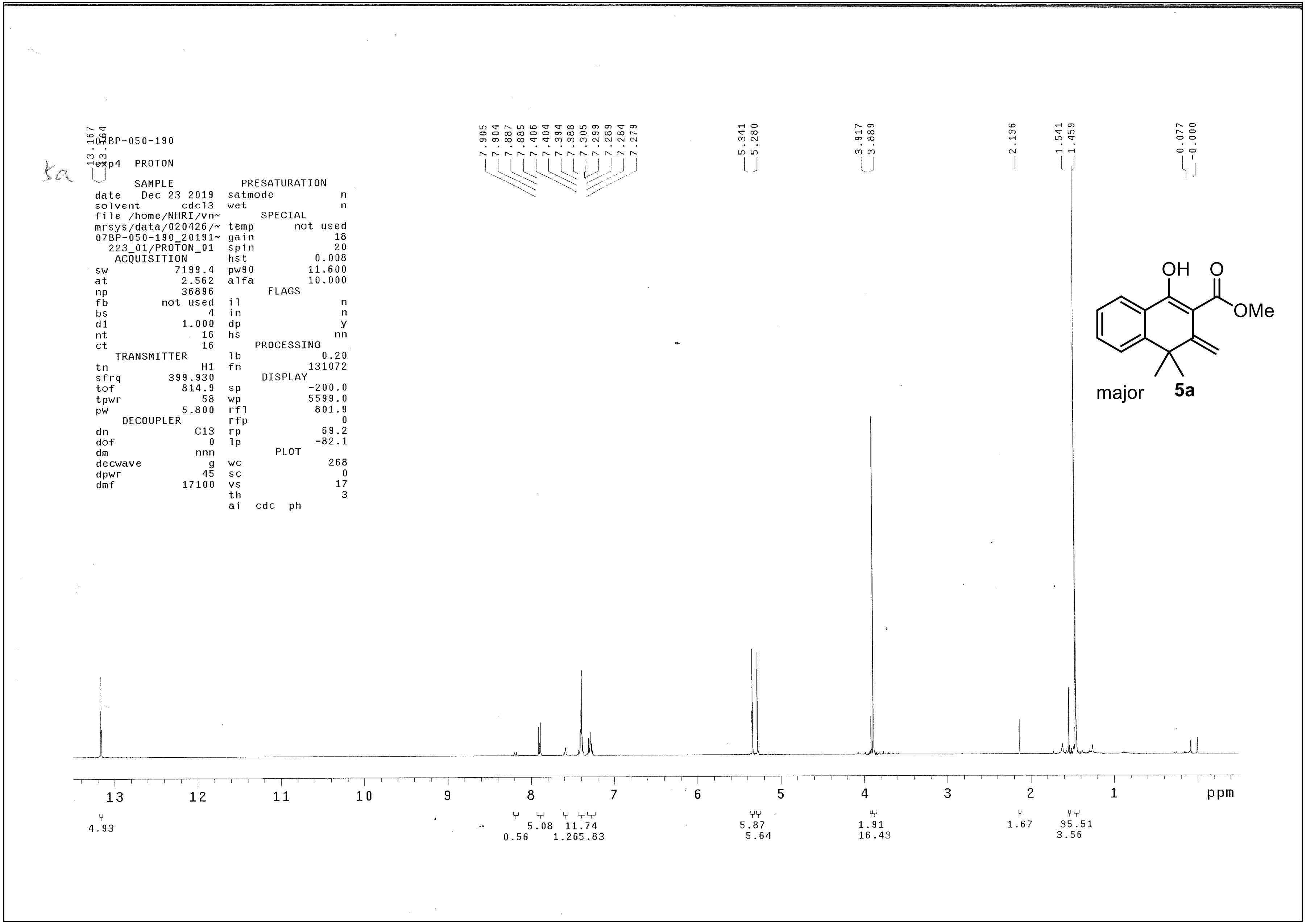

^{1}H NMR spectra for compound **5a**/**5** (t = 0 h)

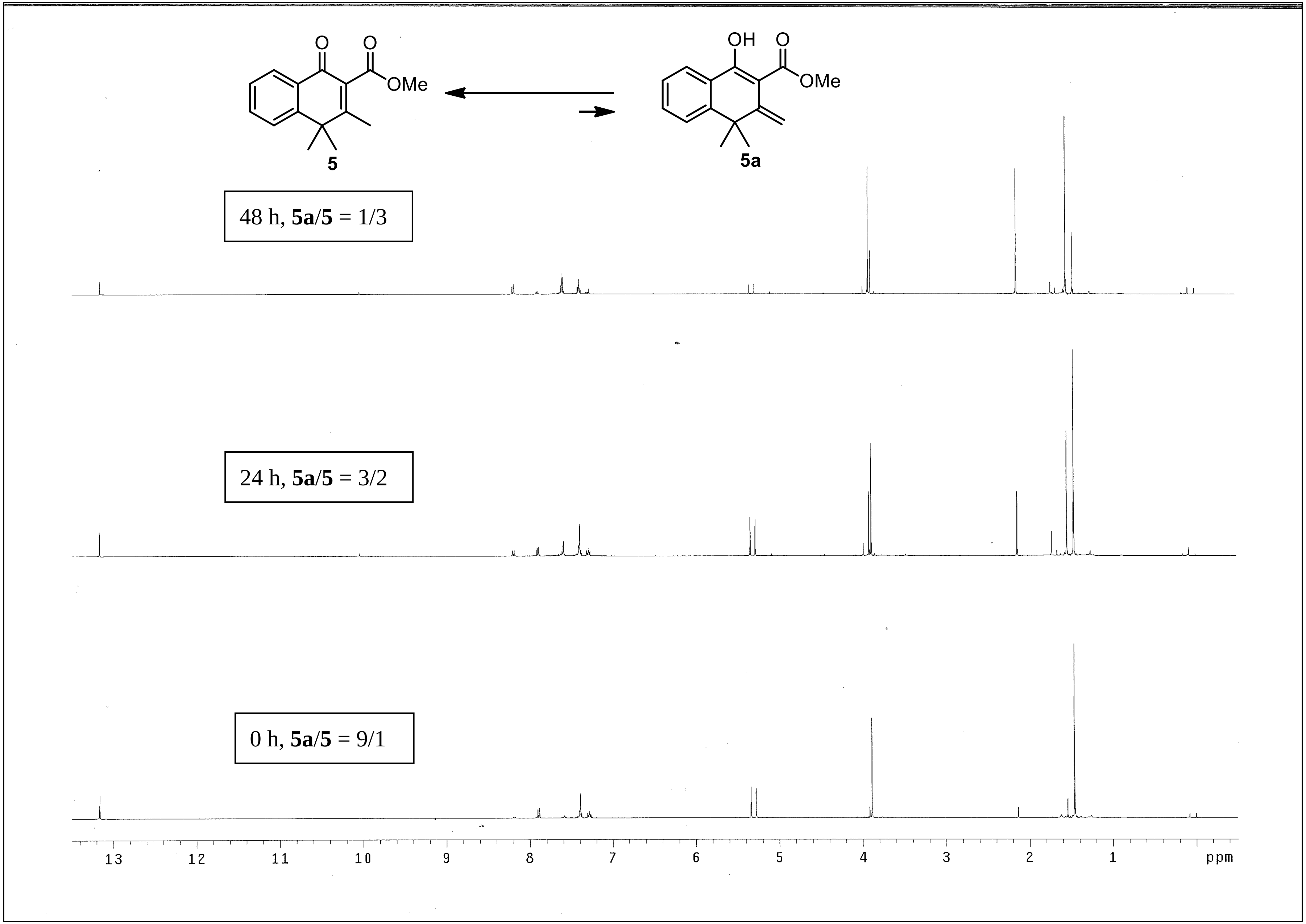

^{1}H NMR spectra for compound **5a/5** (**$CDCl_3$**) (t = 0, 24, 48 h)

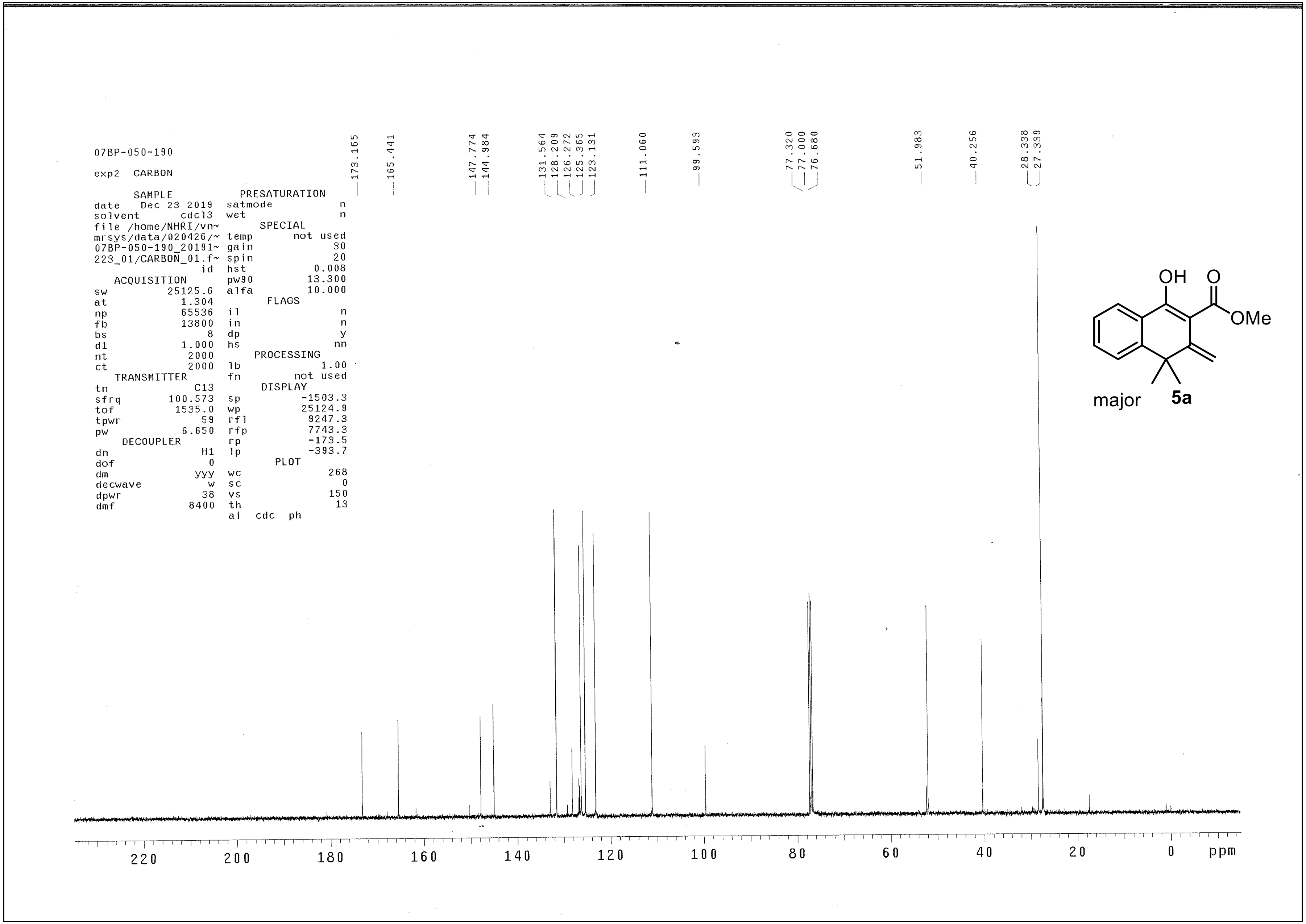

^{13}C NMR spectra for compound **5a**/**5** (t = 0 h)

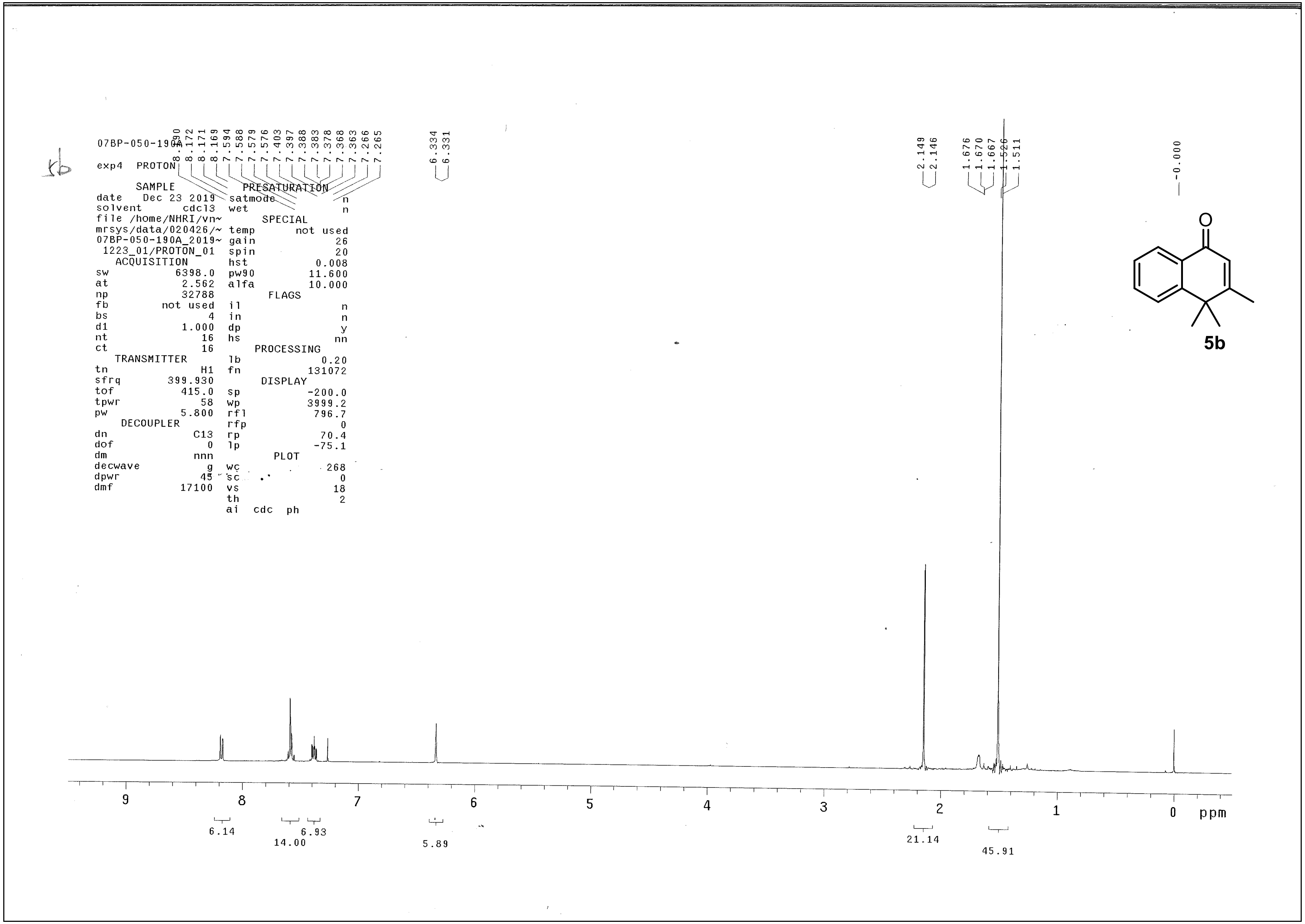

^{1}H NMR spectra for compound **5b**

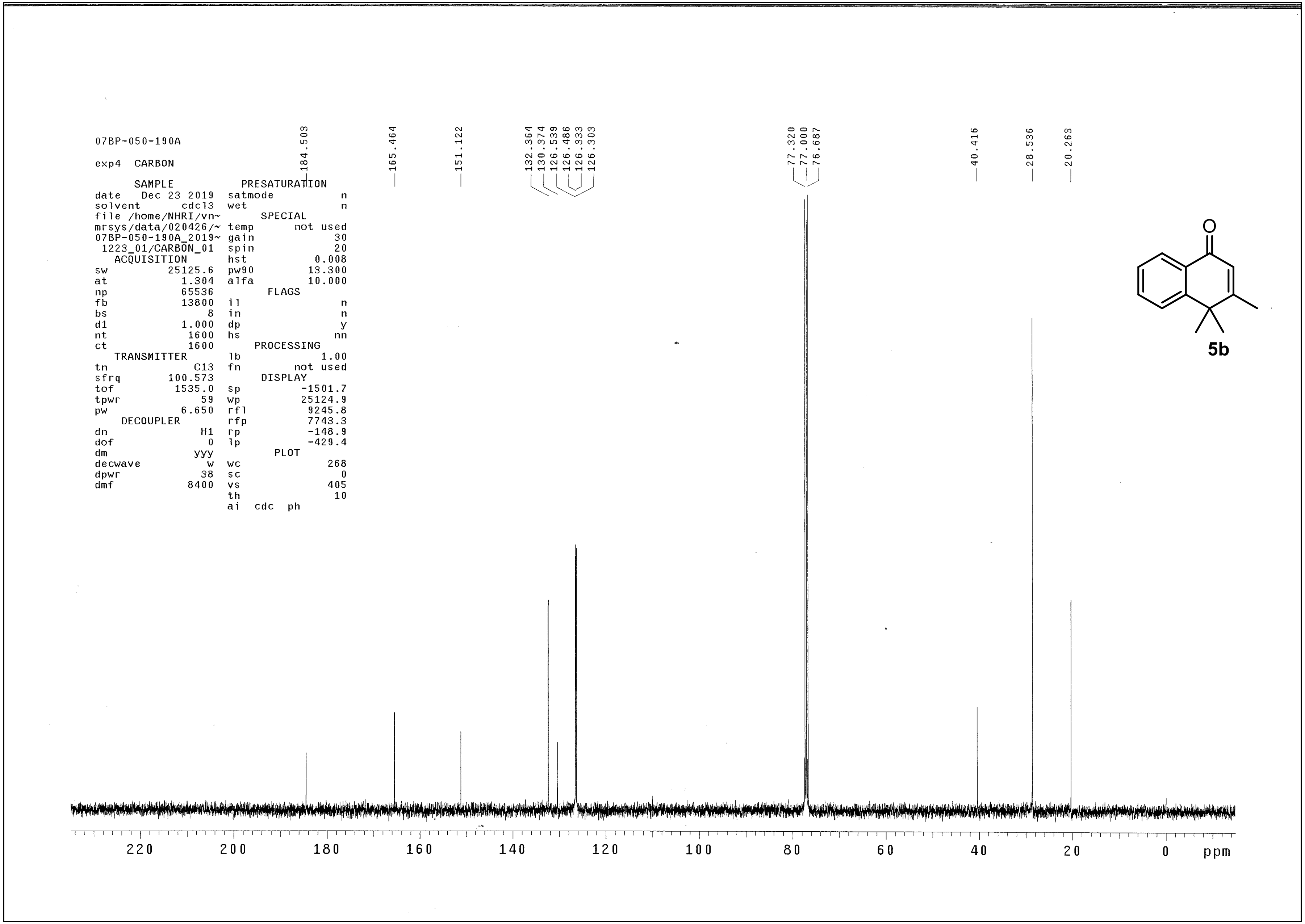

^{13}C NMR spectra for compound **5b**

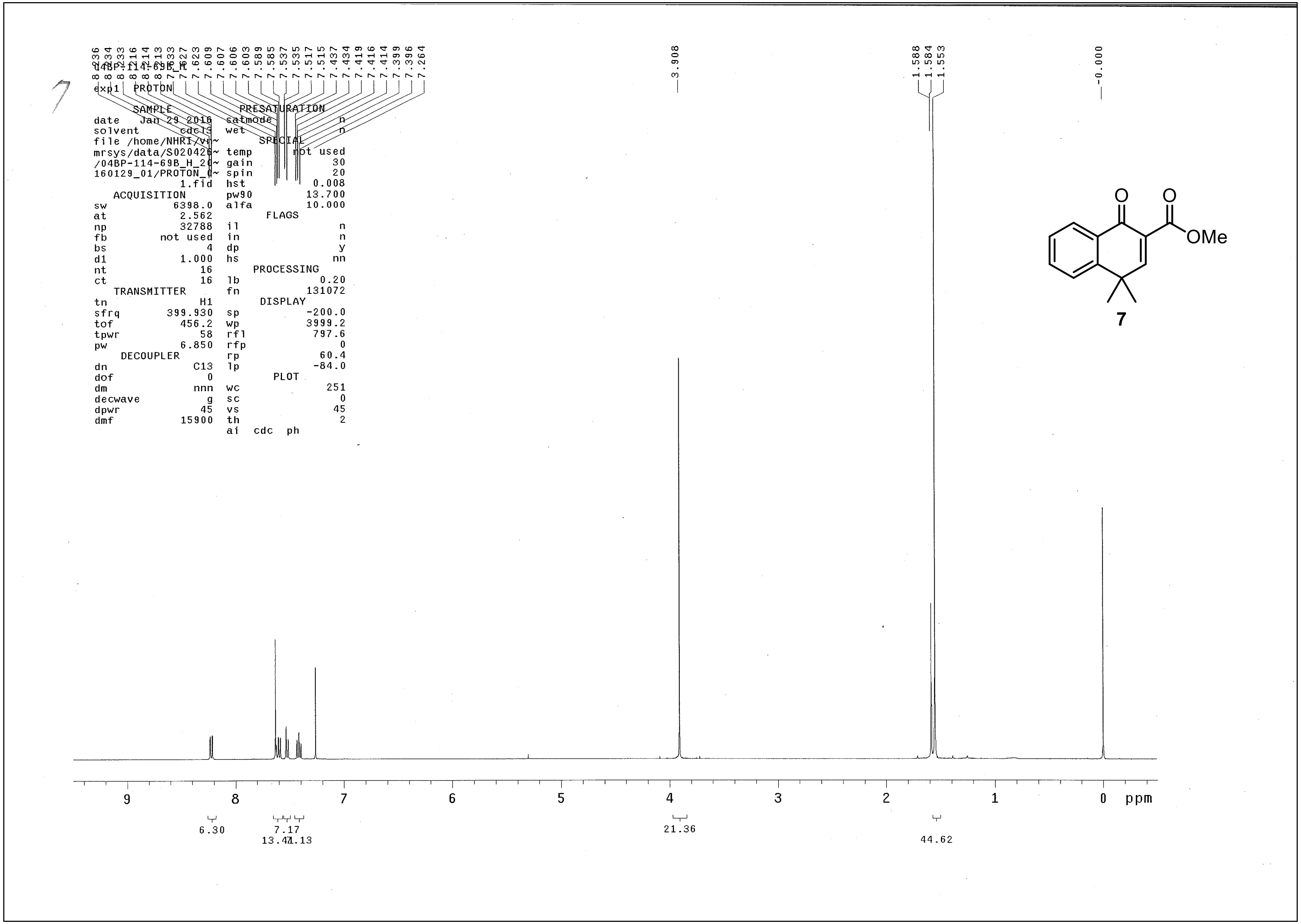

^{1}H NMR spectra for compound **7**

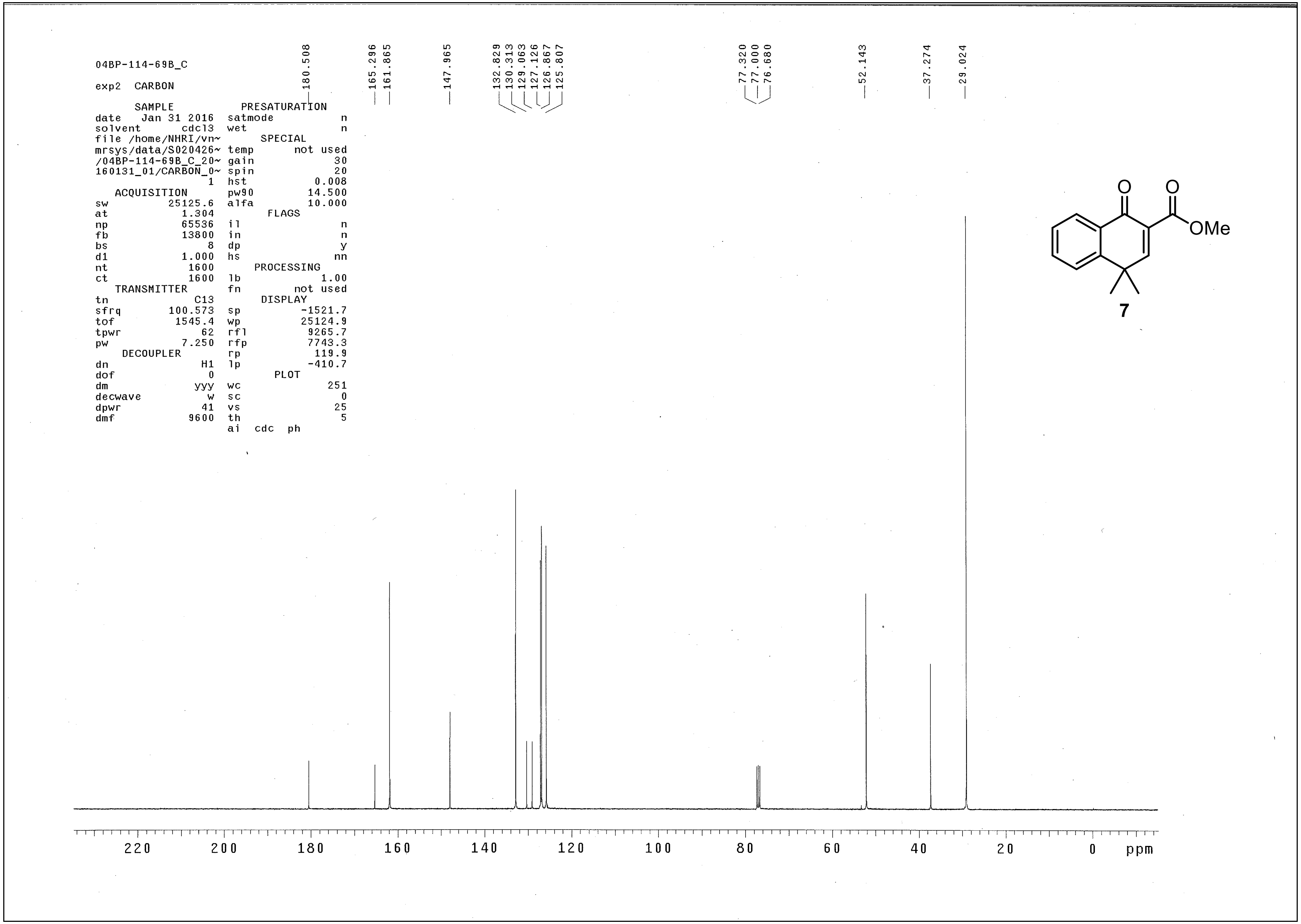

^{13}C NMR spectra for compound **7**

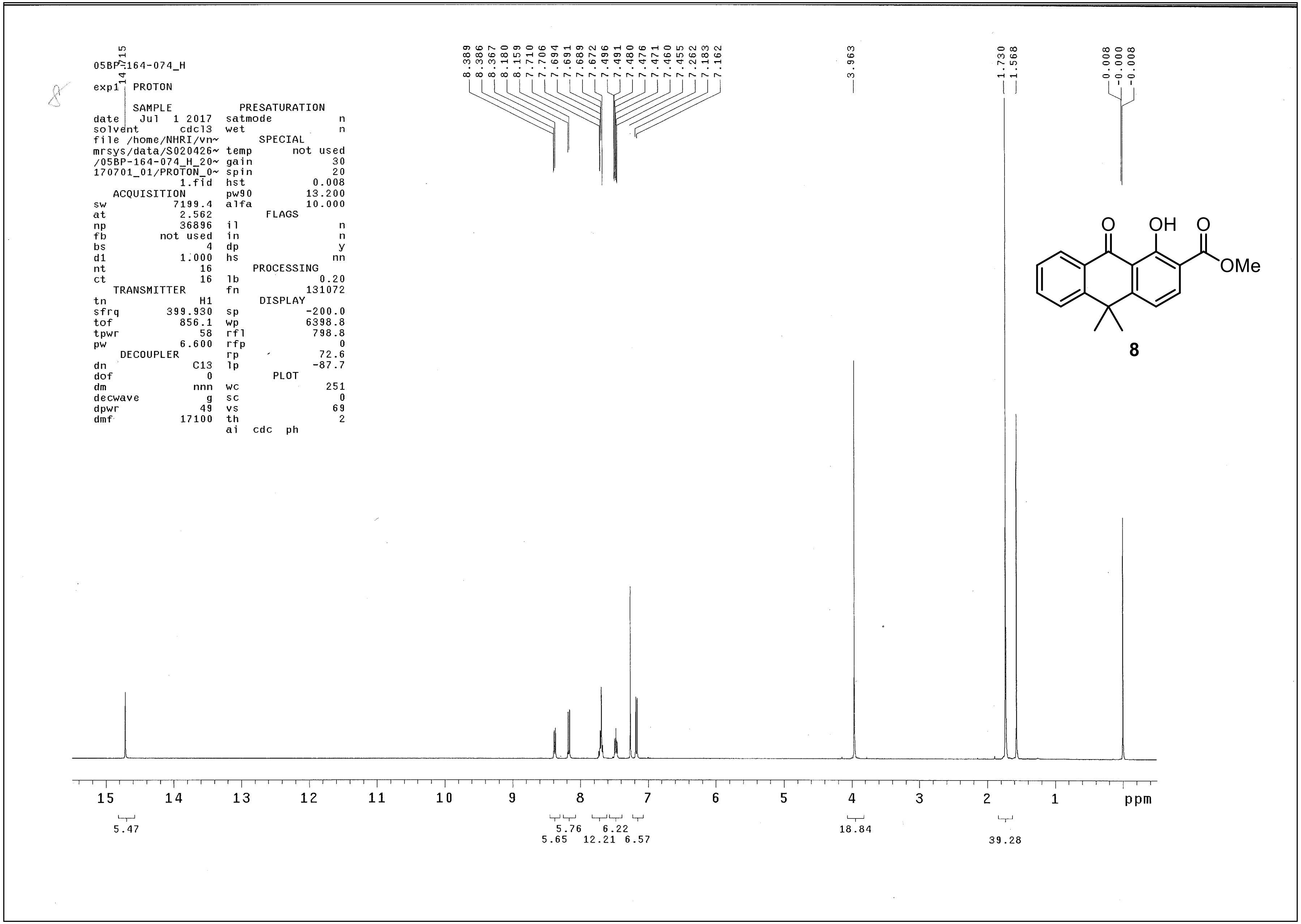

^{1}H NMR spectra for compound **8**

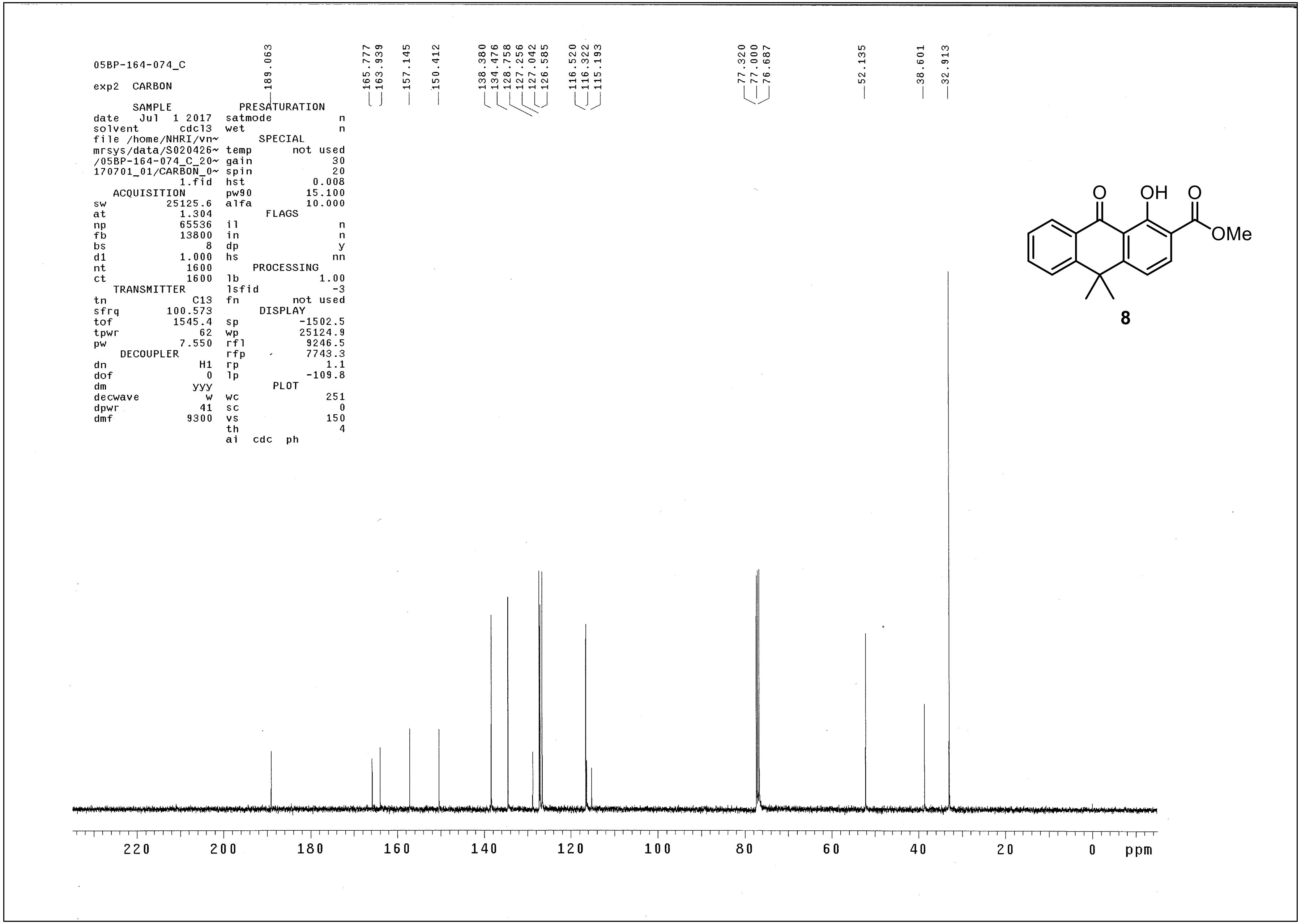

^{13}C NMR spectra for compound **8**

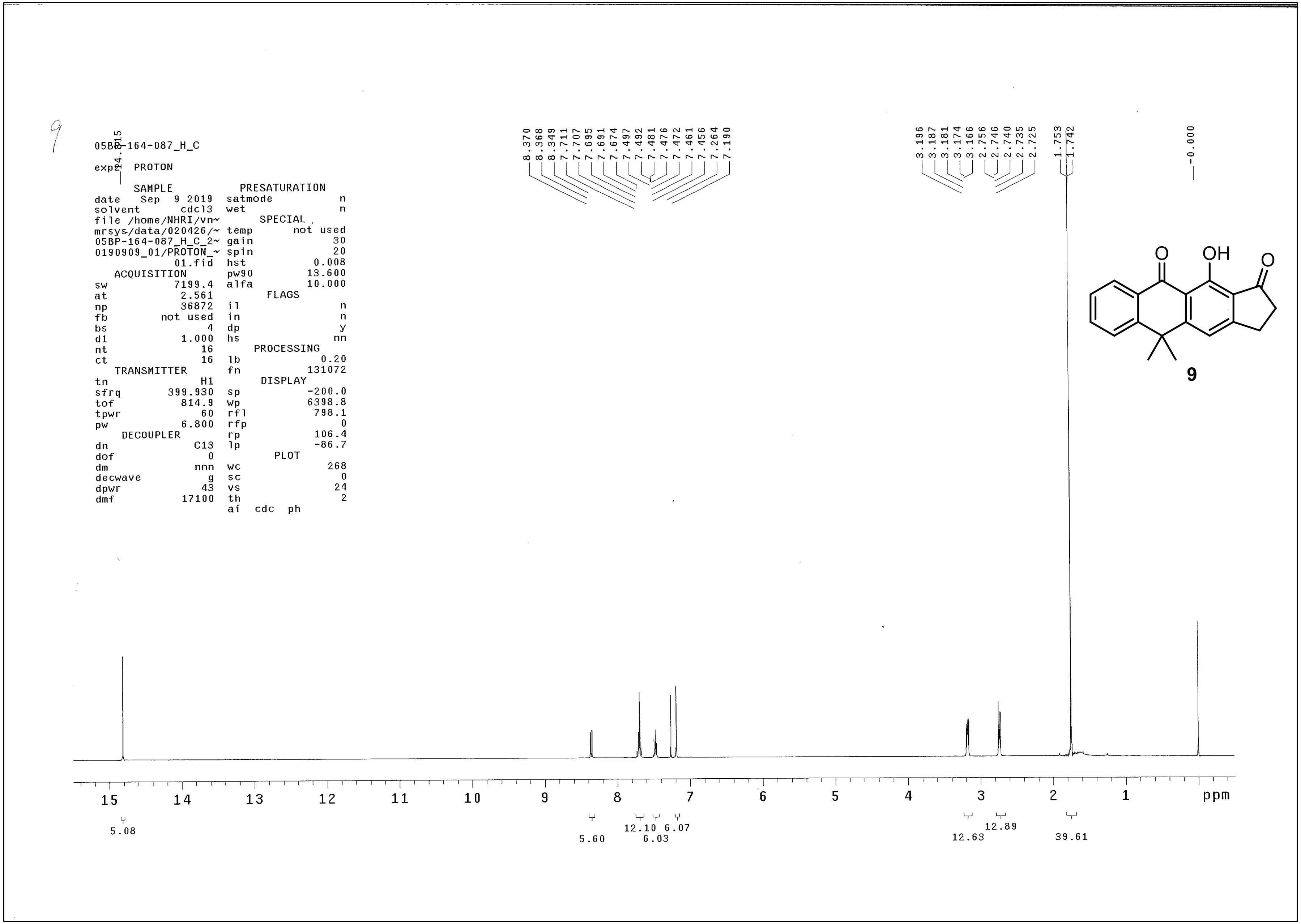

^{1}H NMR spectra for compound **9**

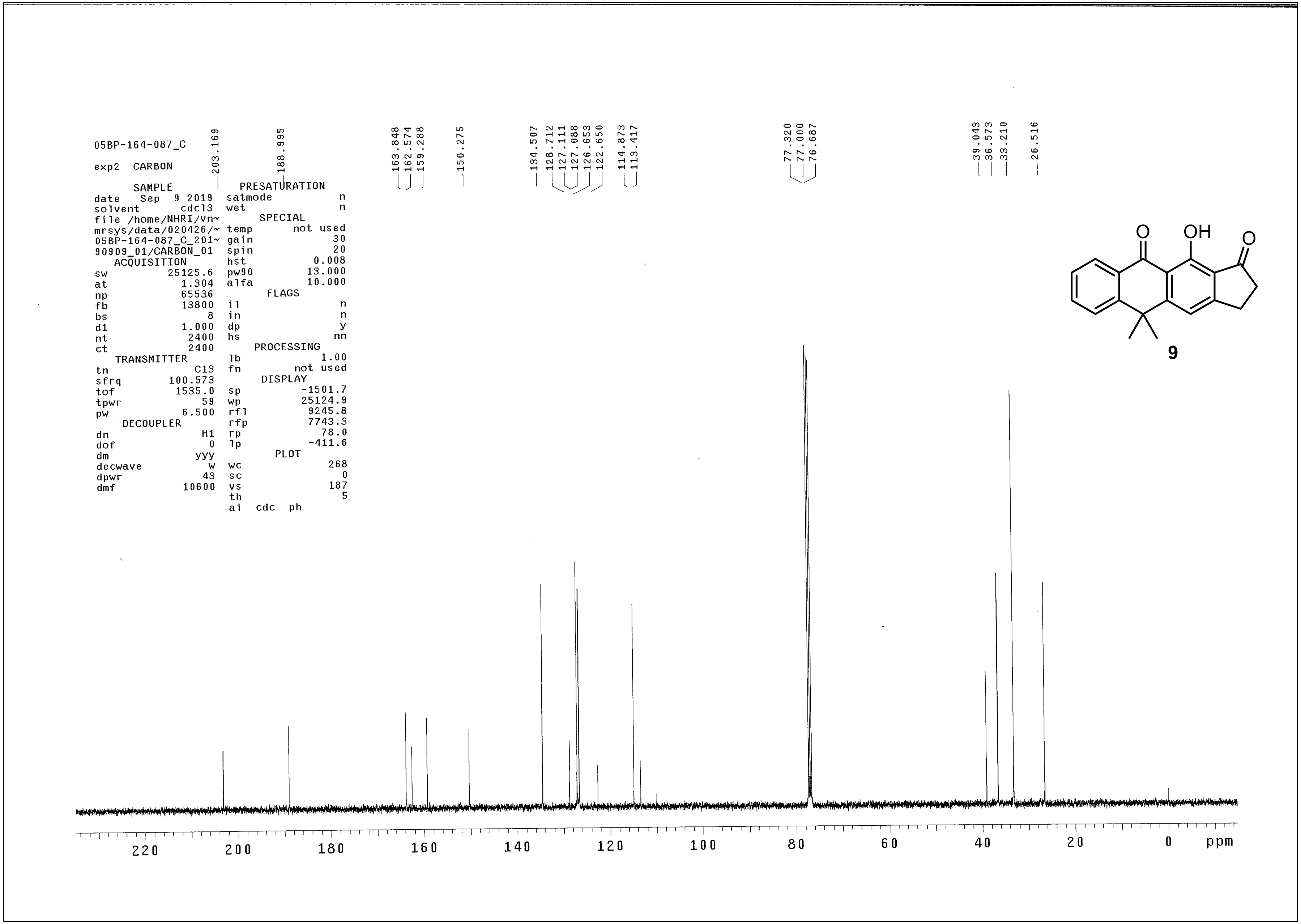

^{13}C NMR spectra for compound **9**

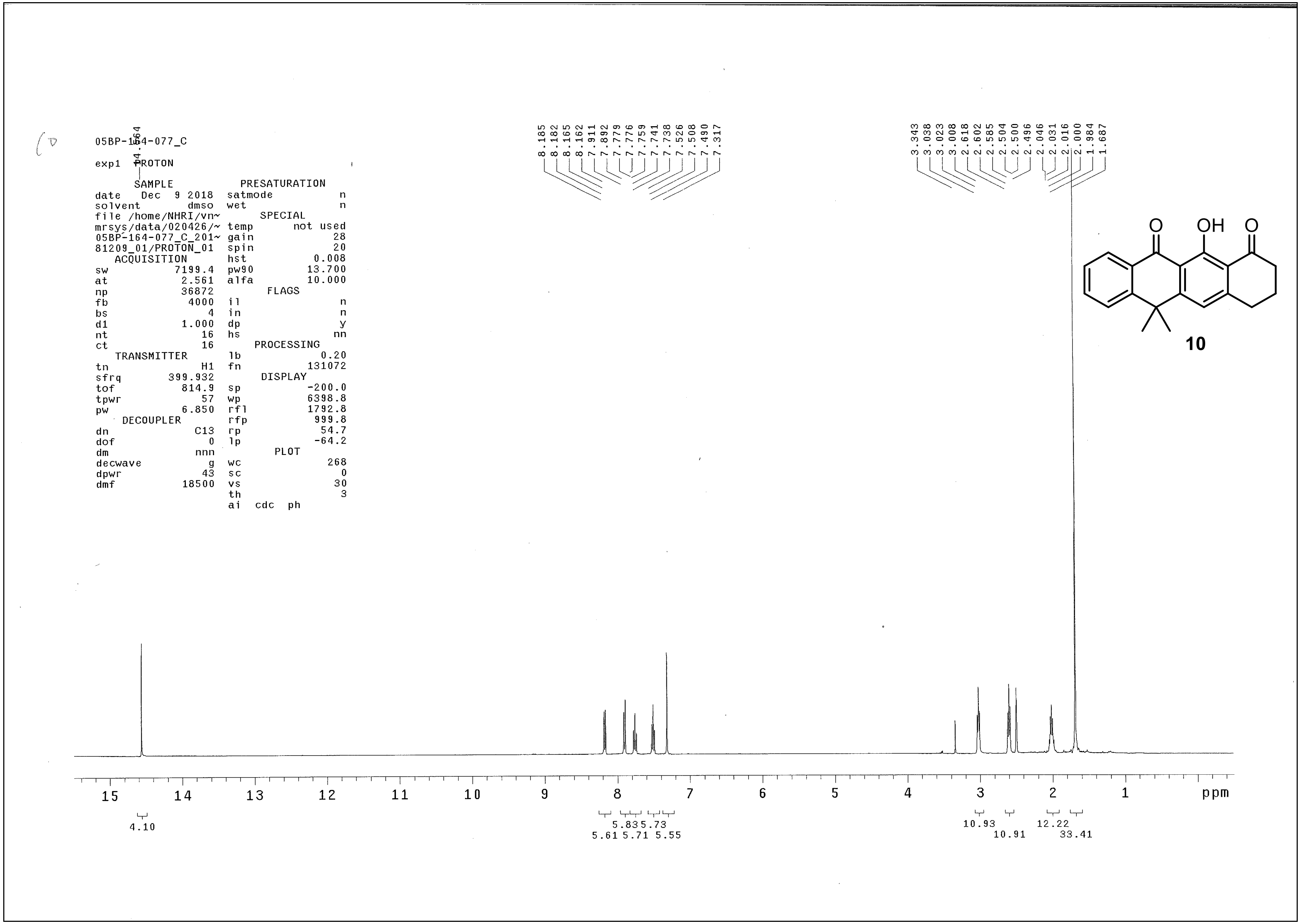

^{1}H NMR spectra for compound **10**

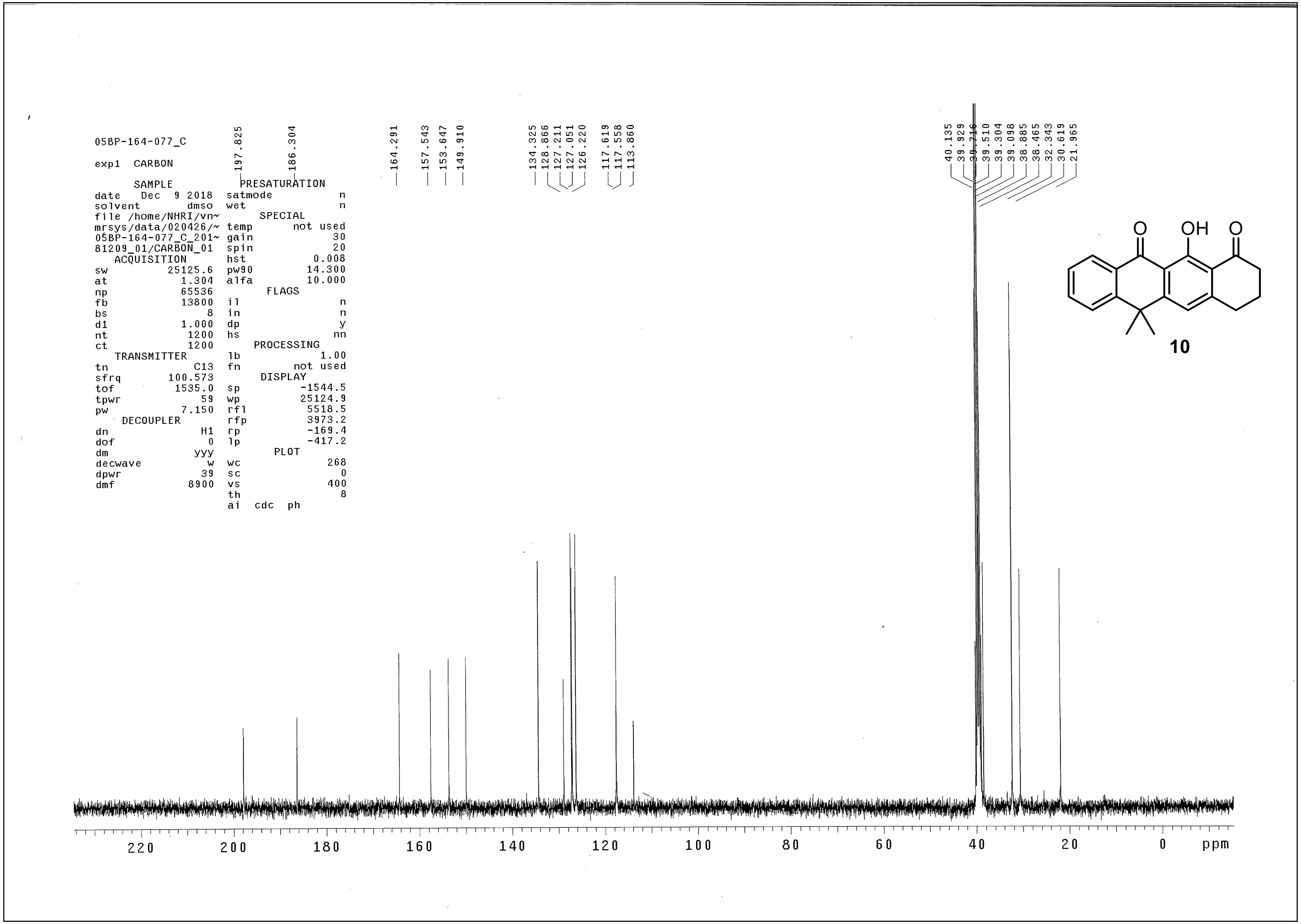

^{13}C NMR spectra for compound **10**

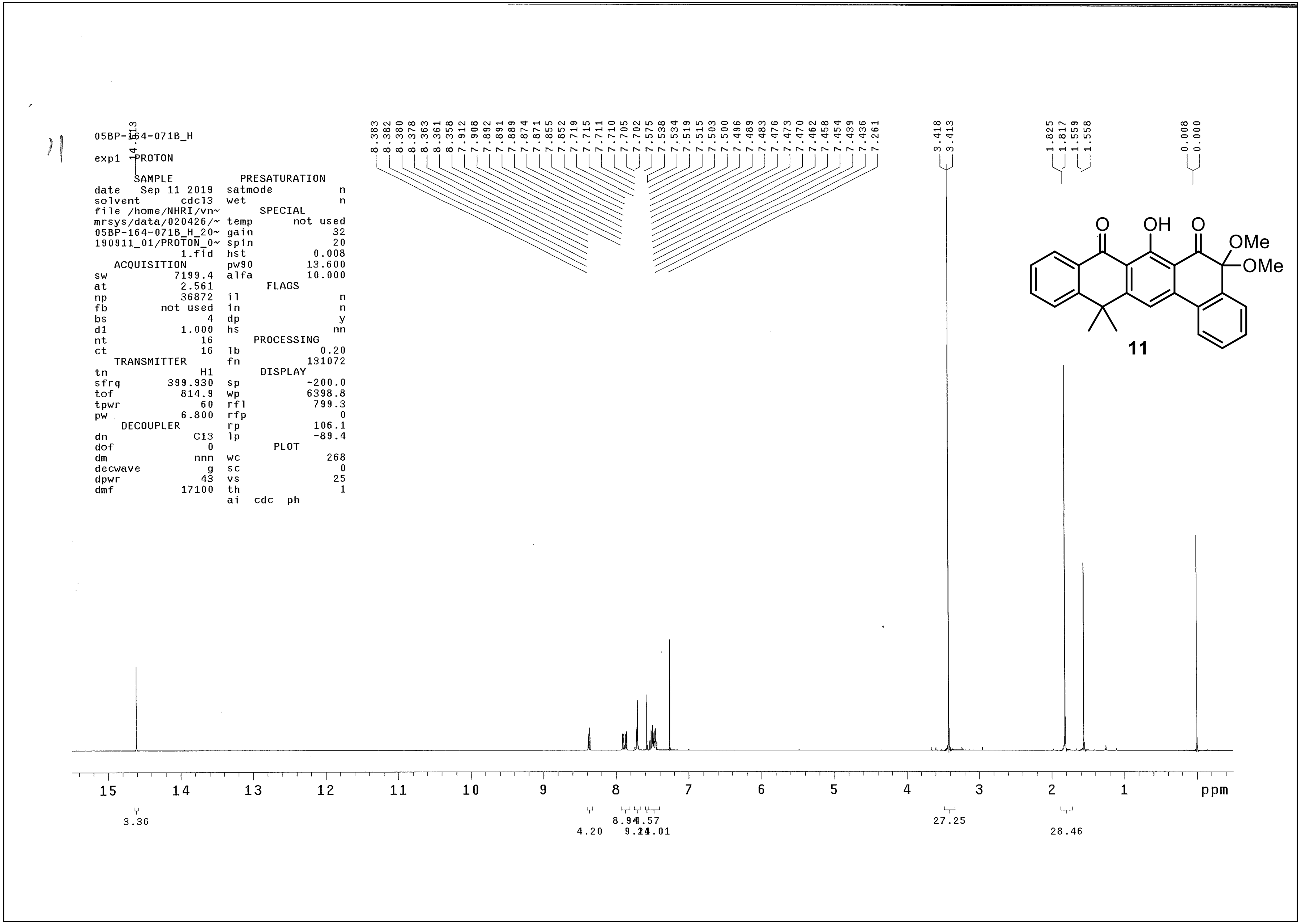

^{1}H NMR spectra for compound **11**

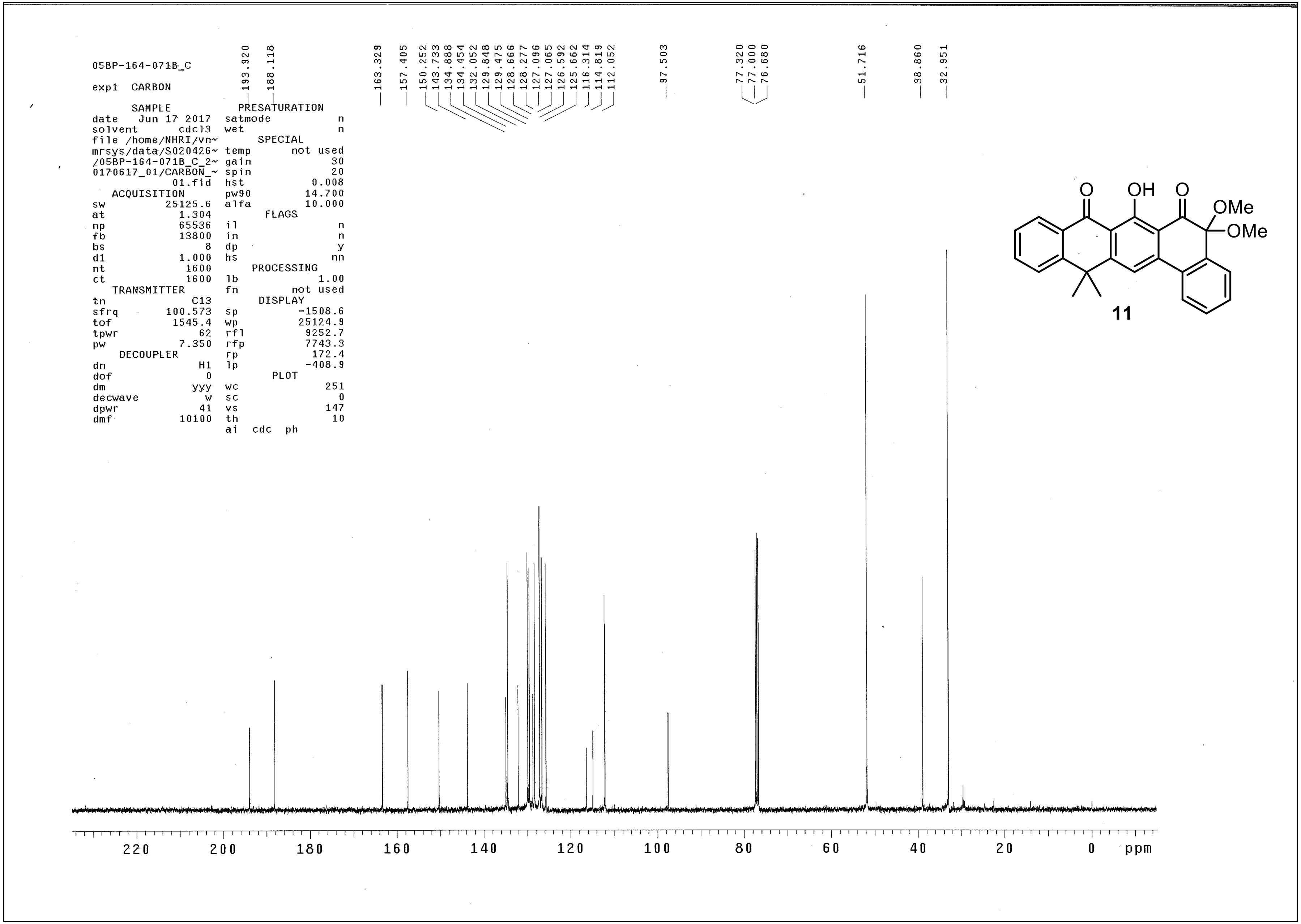

^{13}C NMR spectra for compound **11**

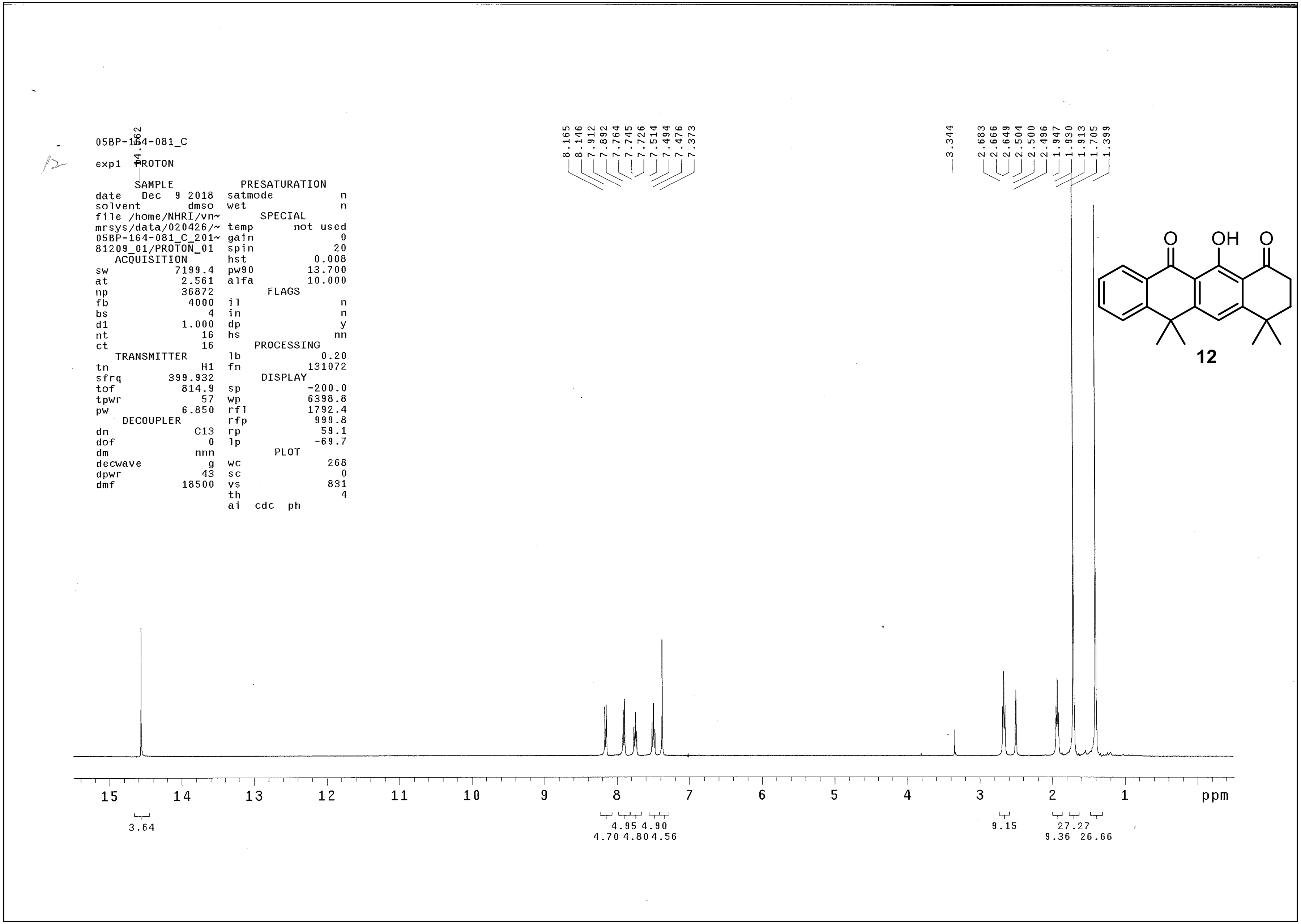

^{1}H NMR spectra for compound **12**

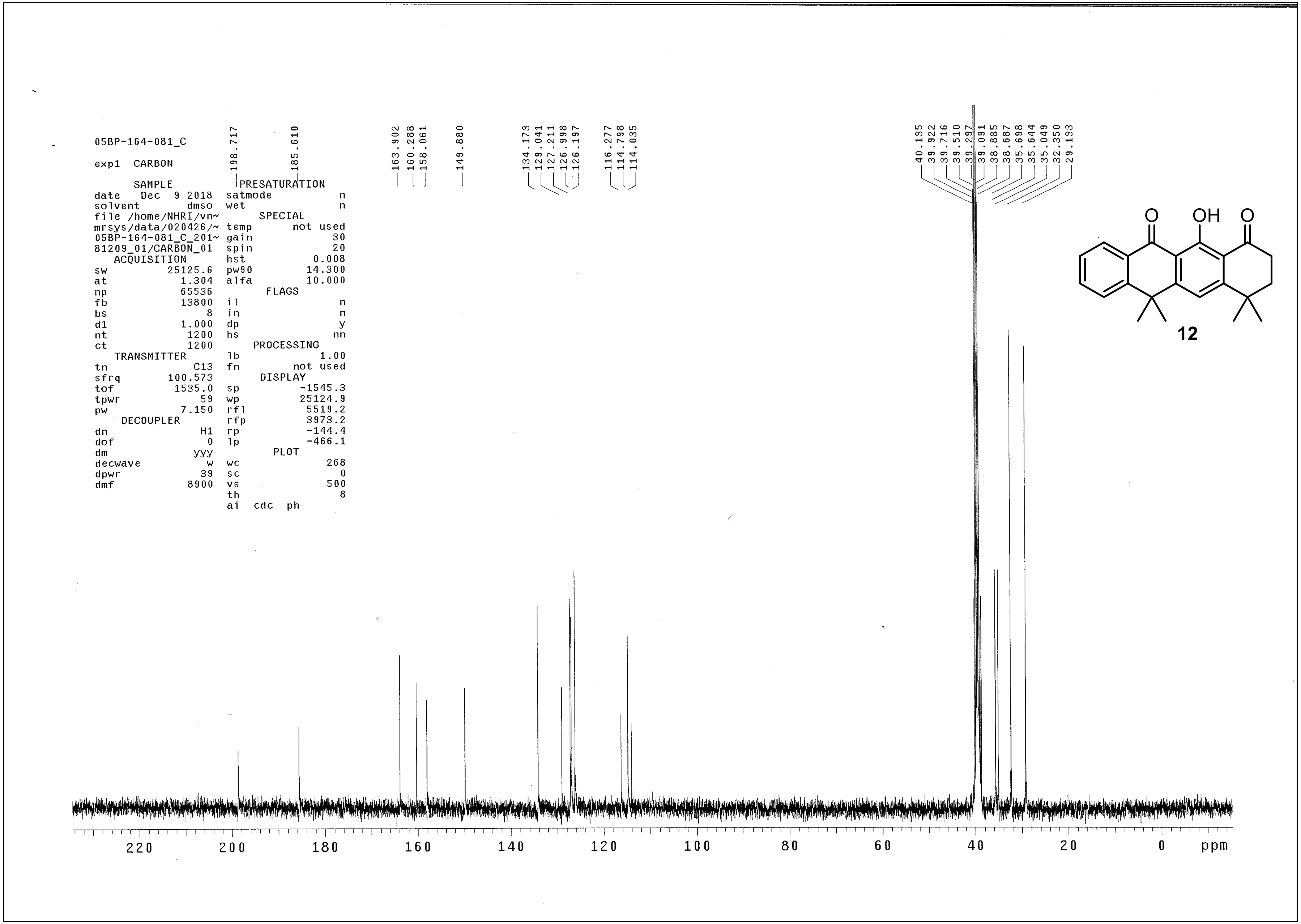

^{13}C NMR spectra for compound **12**

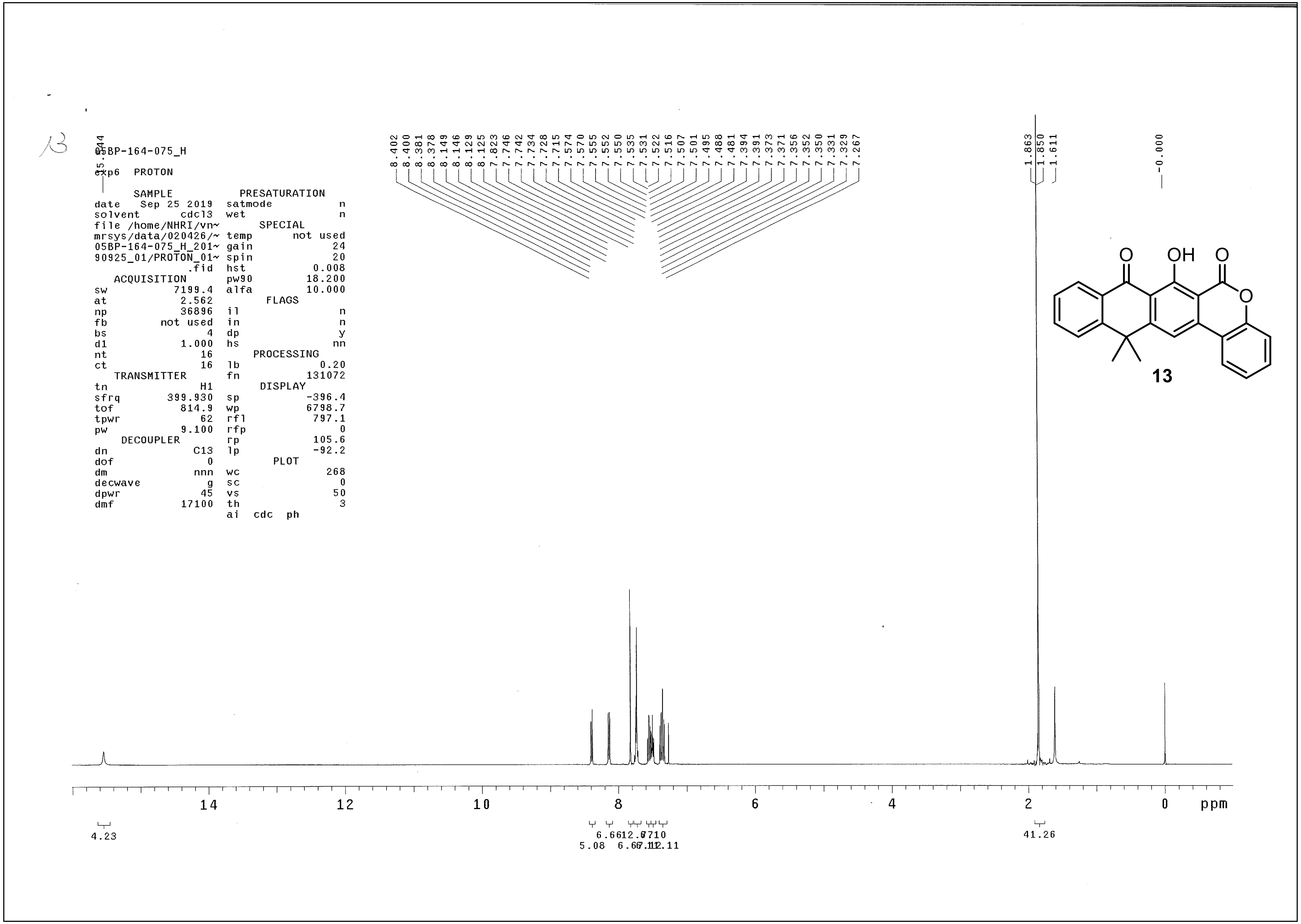

^{1}H NMR spectra for compound **13**

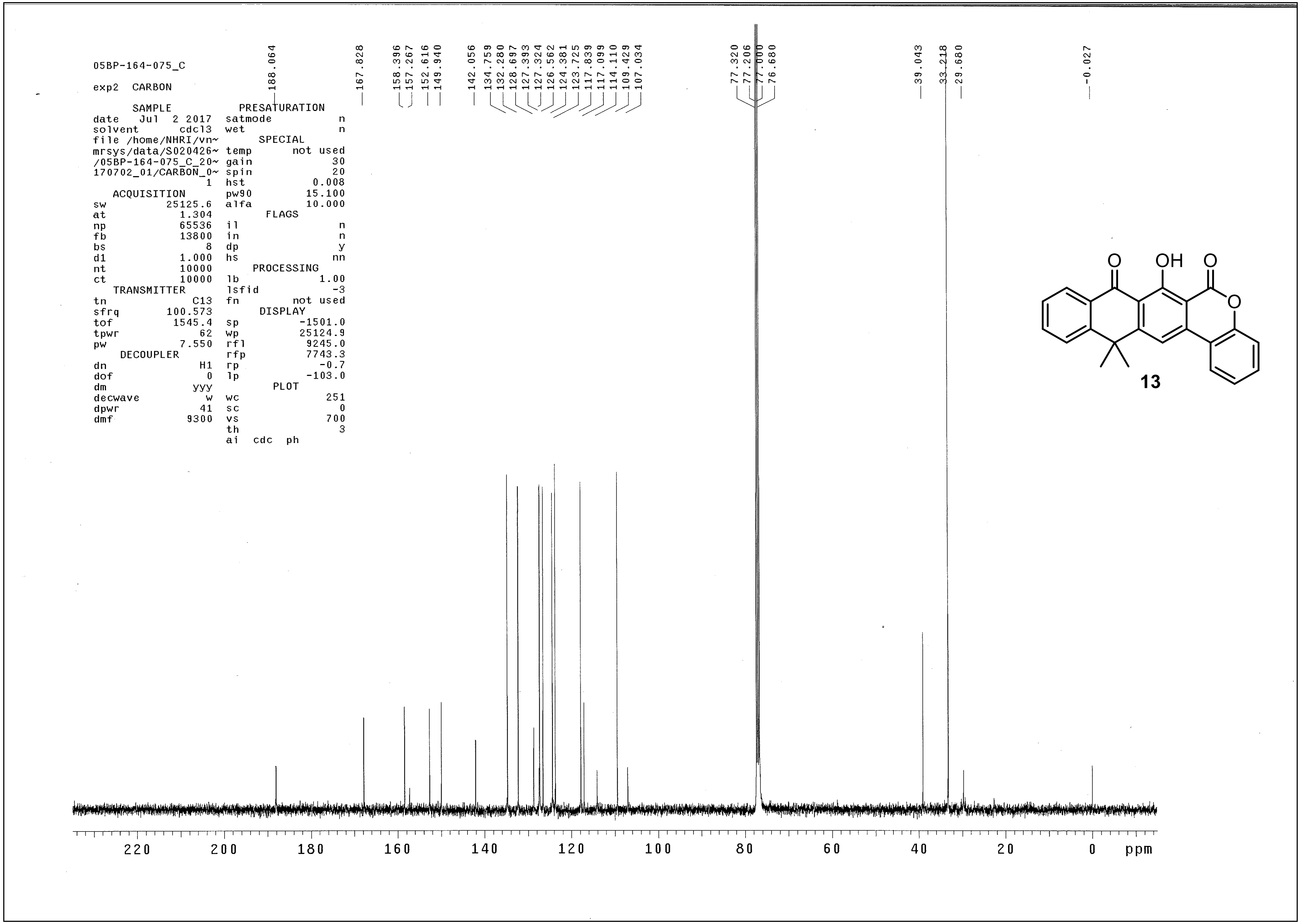

^{13}C NMR spectra for compound **13**

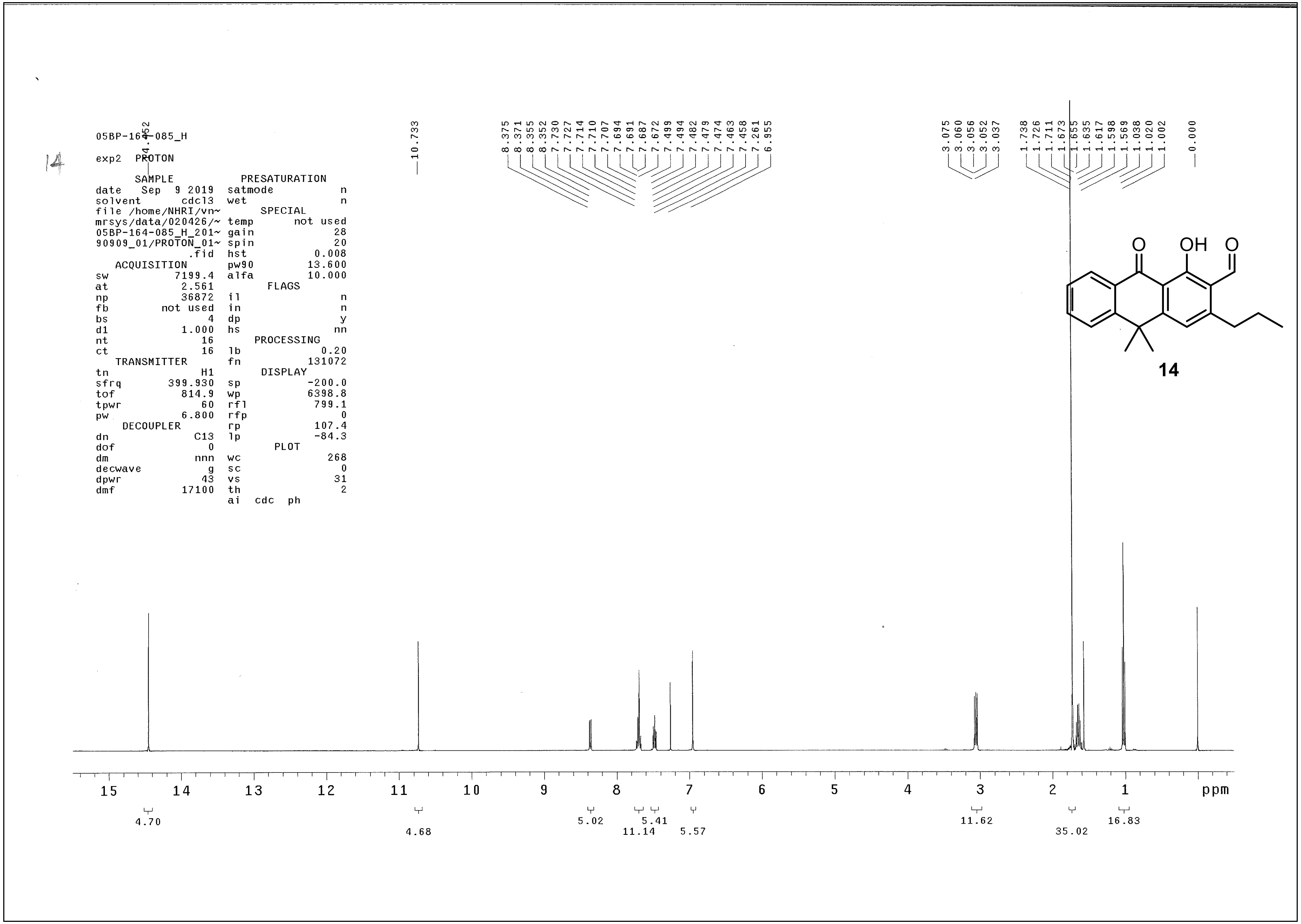

^{1}H NMR spectra for compound **14**

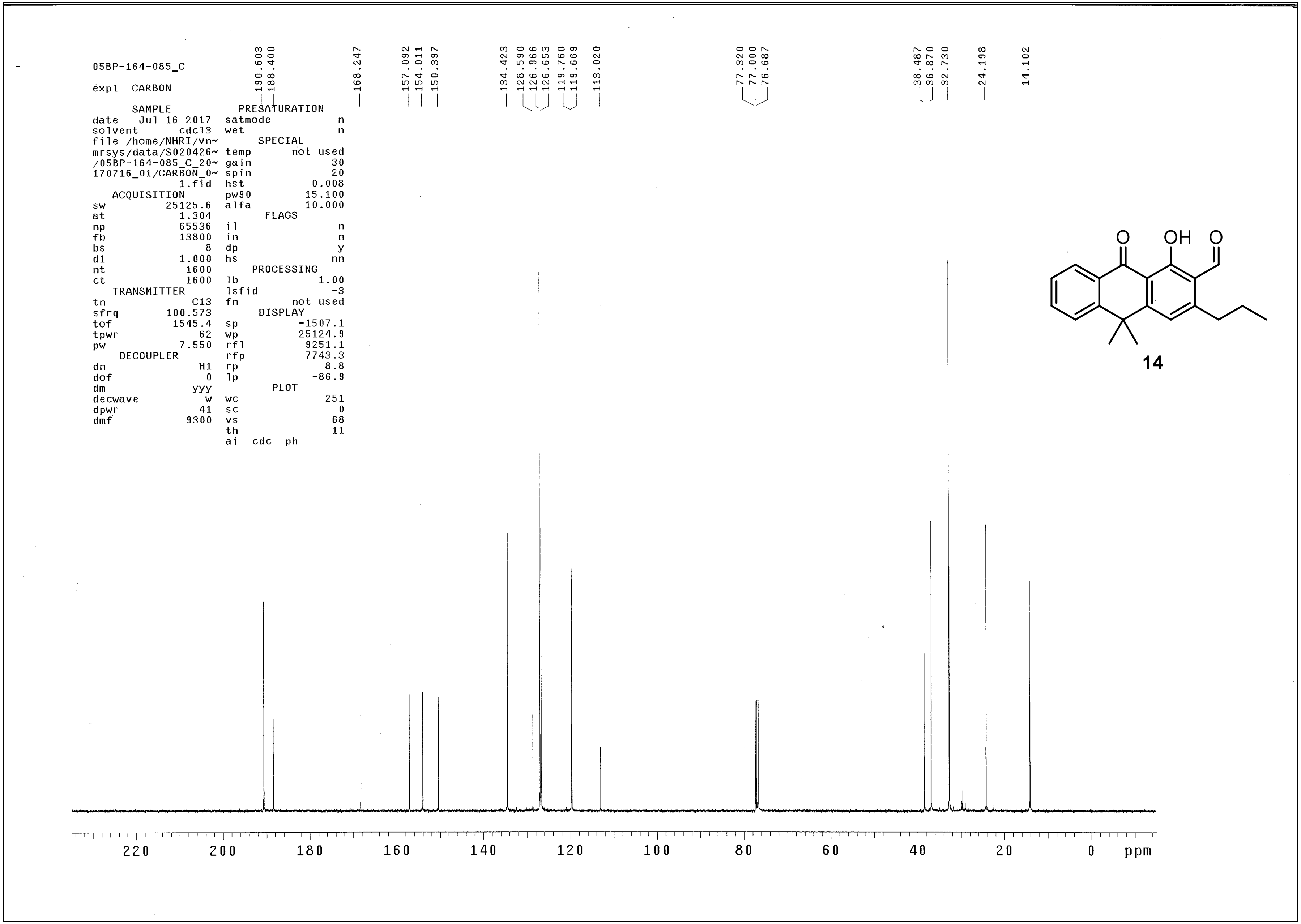

^{13}C NMR spectra for compound **14**

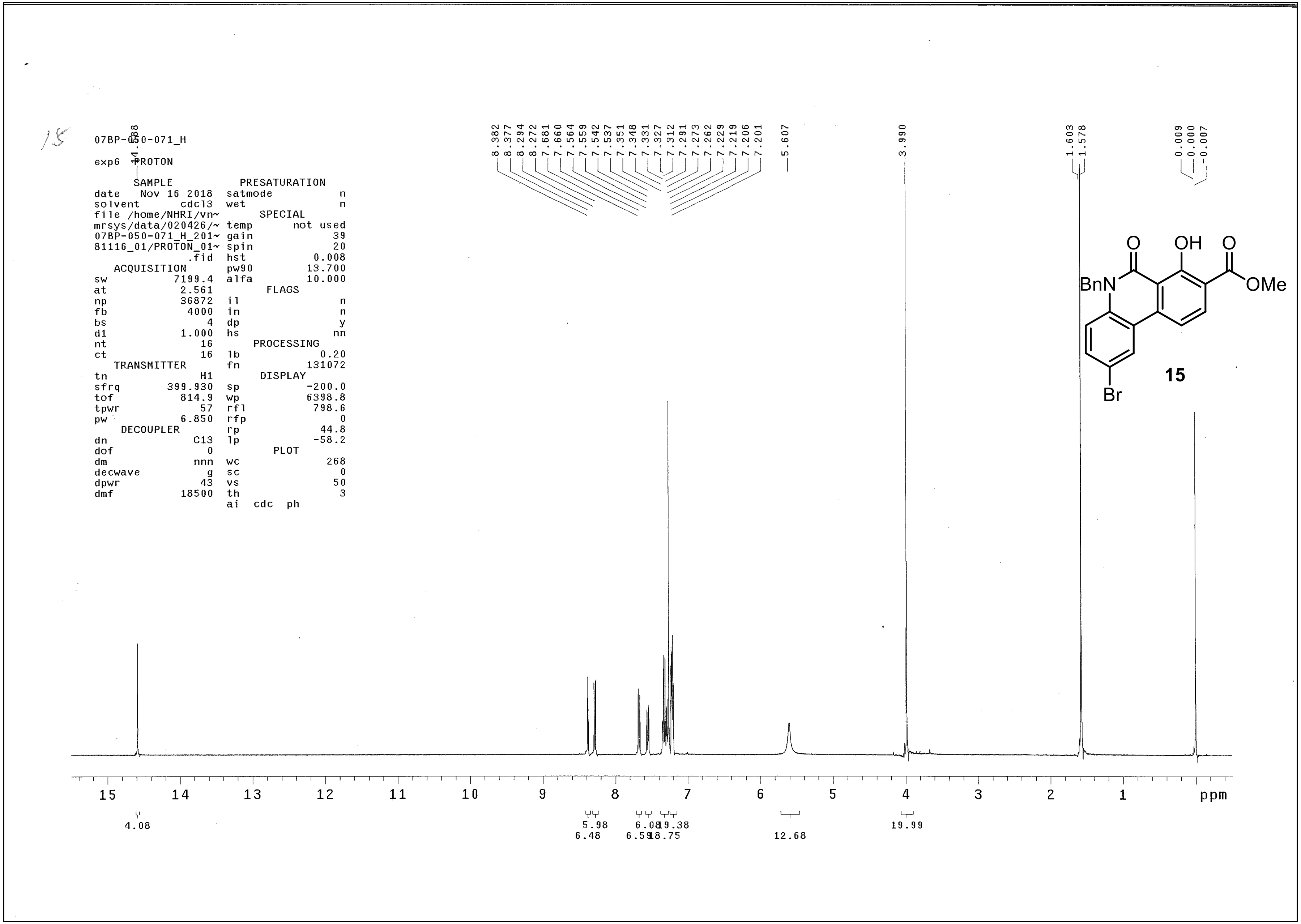

^{1}H NMR spectra for compound **15**

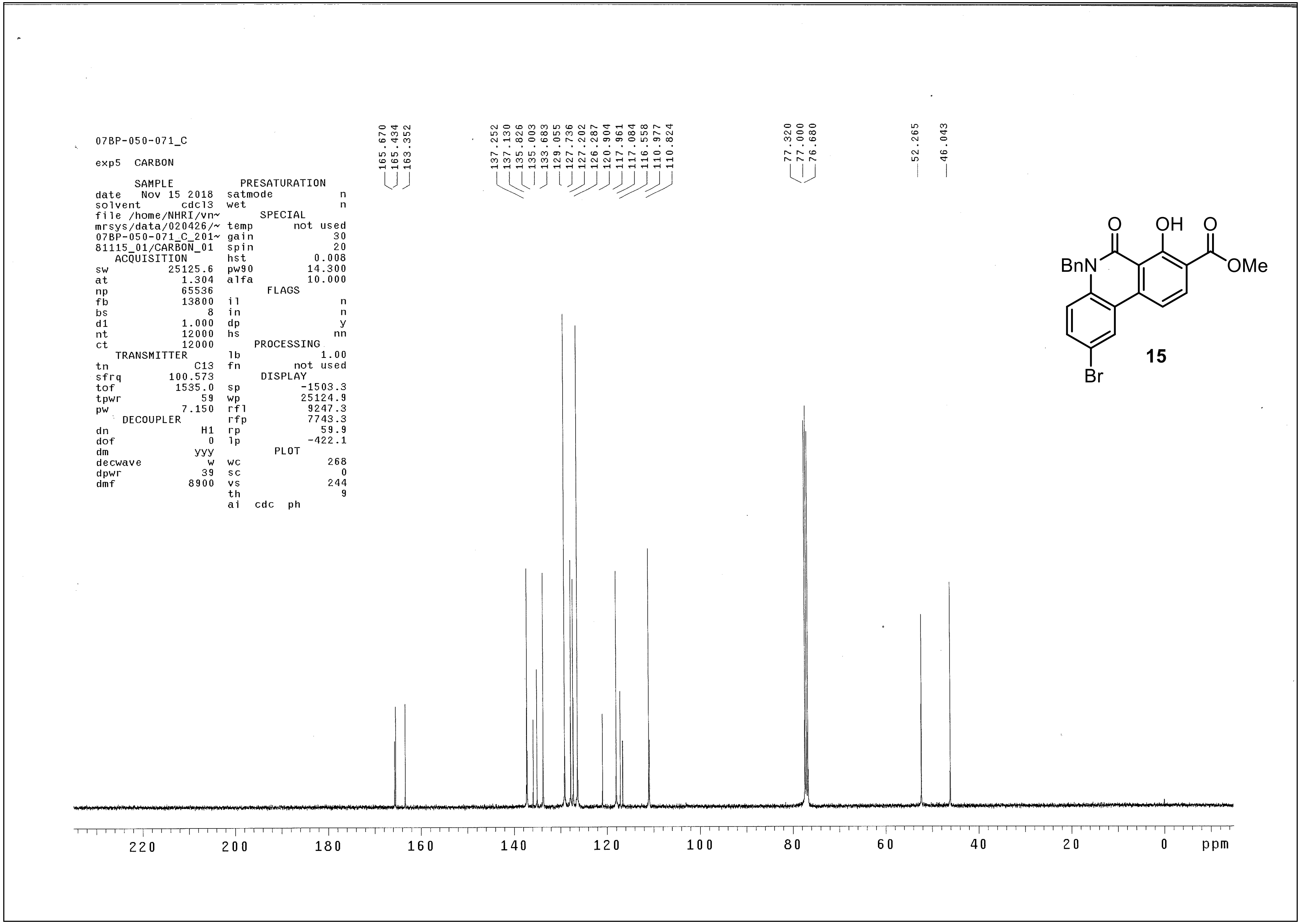

^{13}C NMR spectra for compound **15**

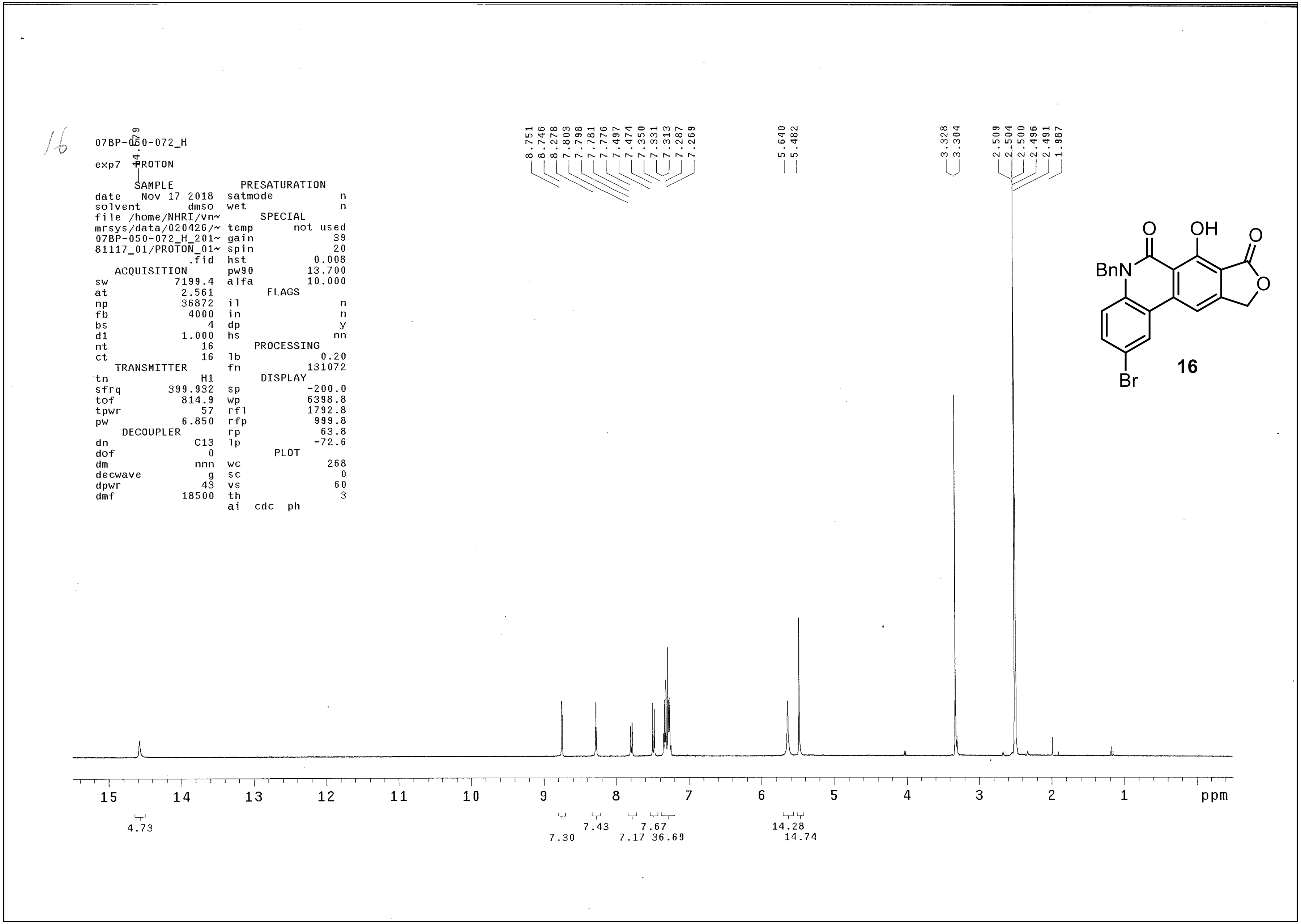

^{1}H NMR spectra for compound **16**

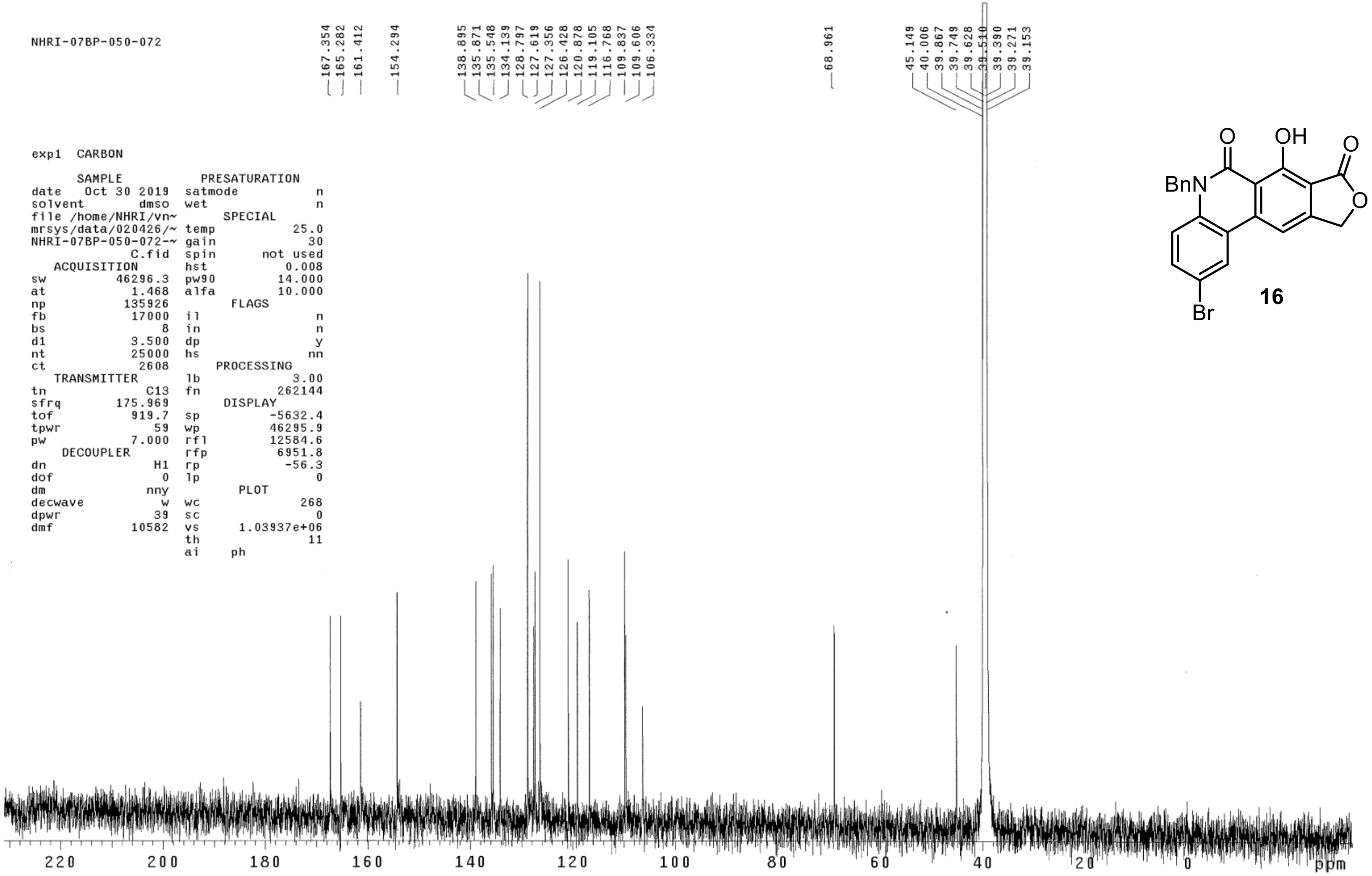

^{13}C NMR spectra for compound **16**

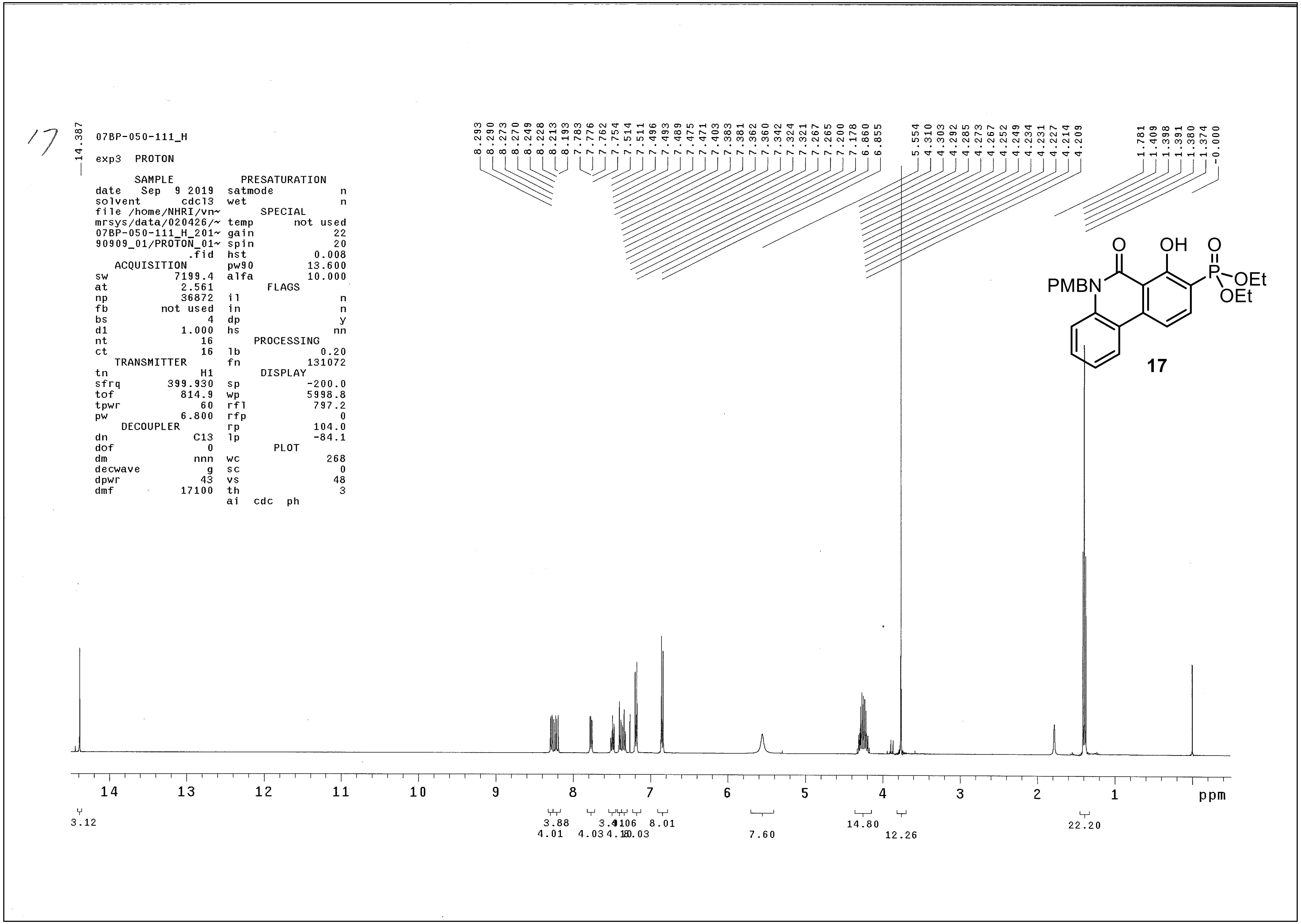

^{1}H NMR spectra for compound **17**

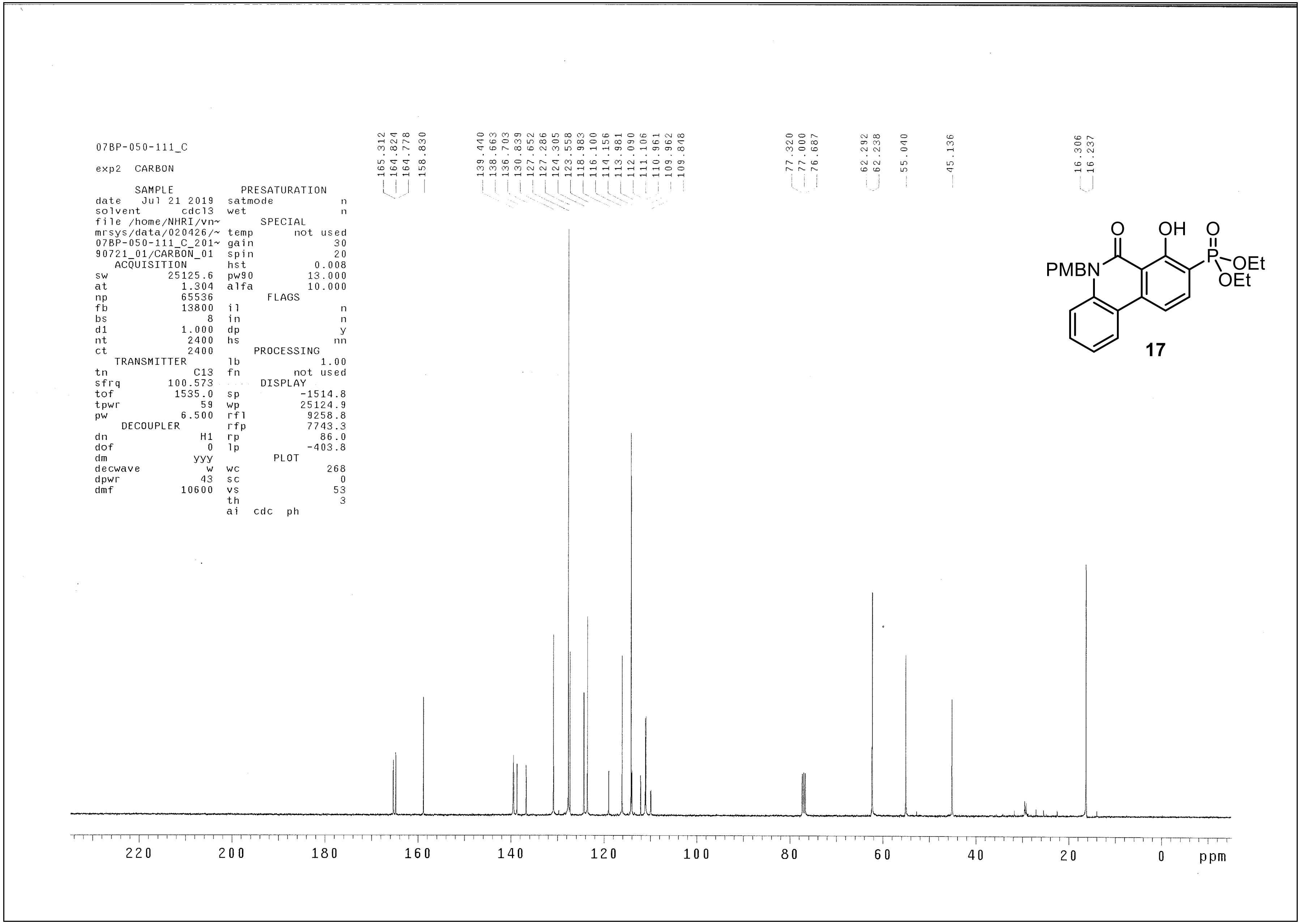

^{13}C NMR spectra for compound **17**

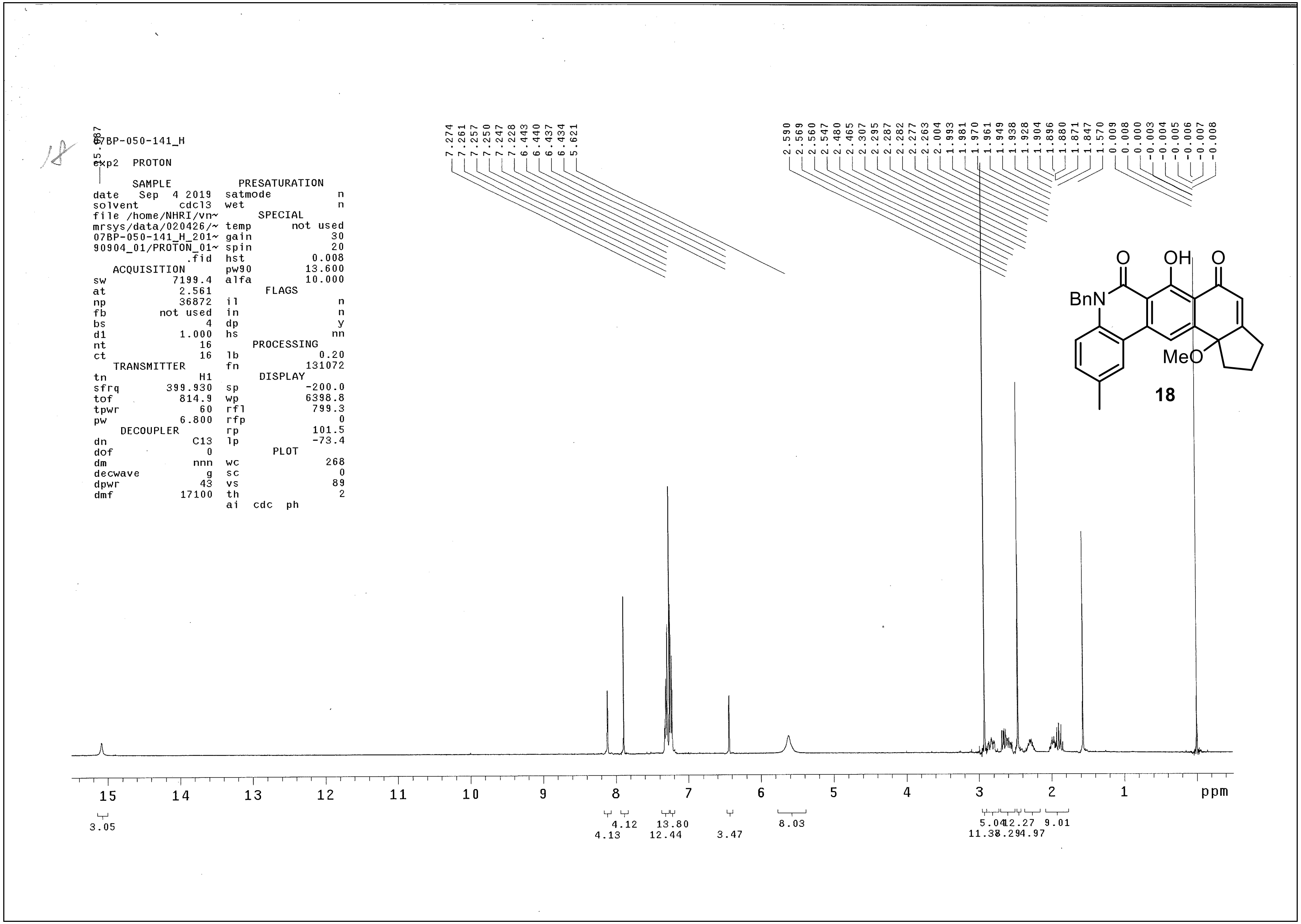

^{1}H NMR spectra for compound **18**

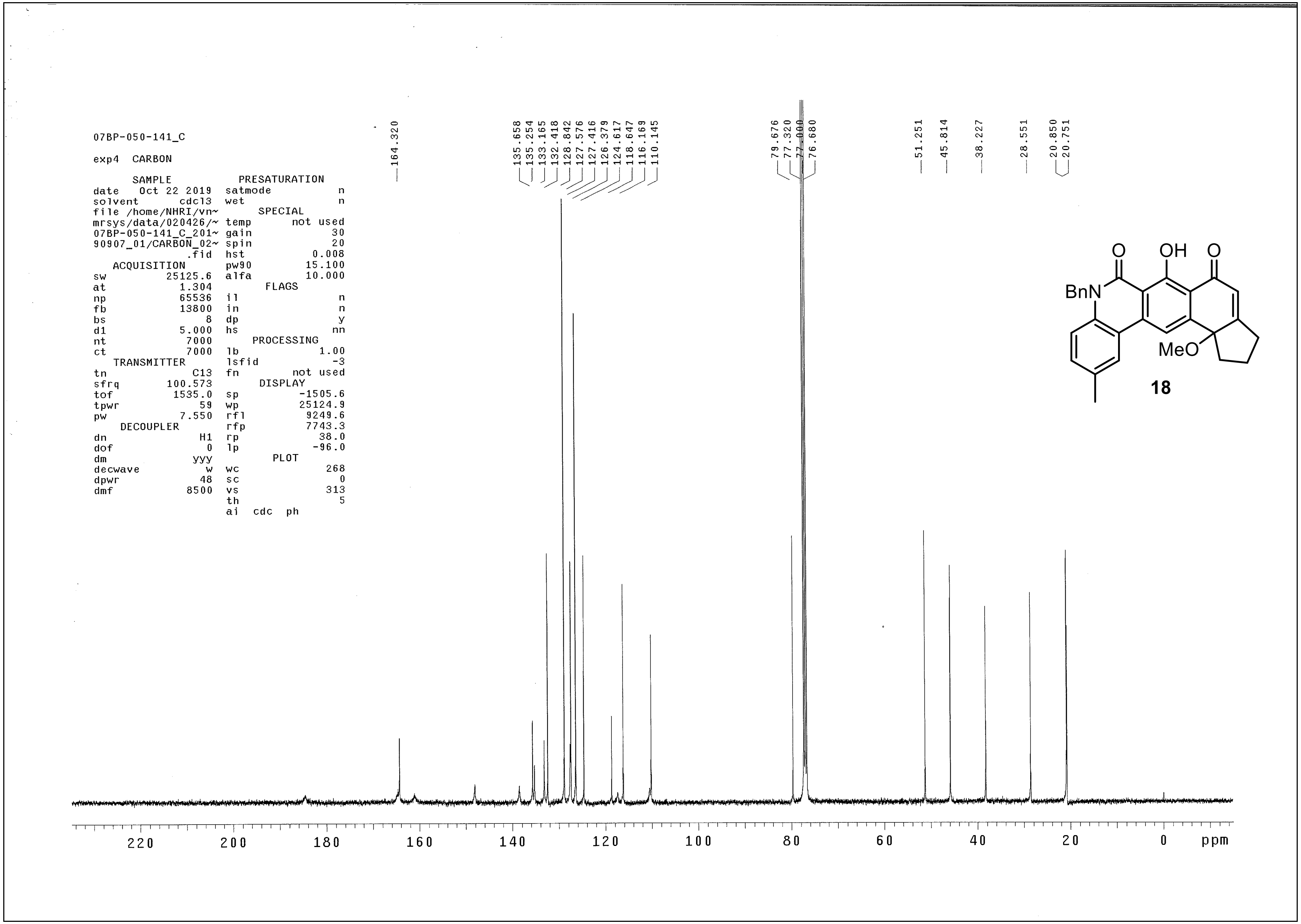

^{13}C NMR spectra for compound **18**

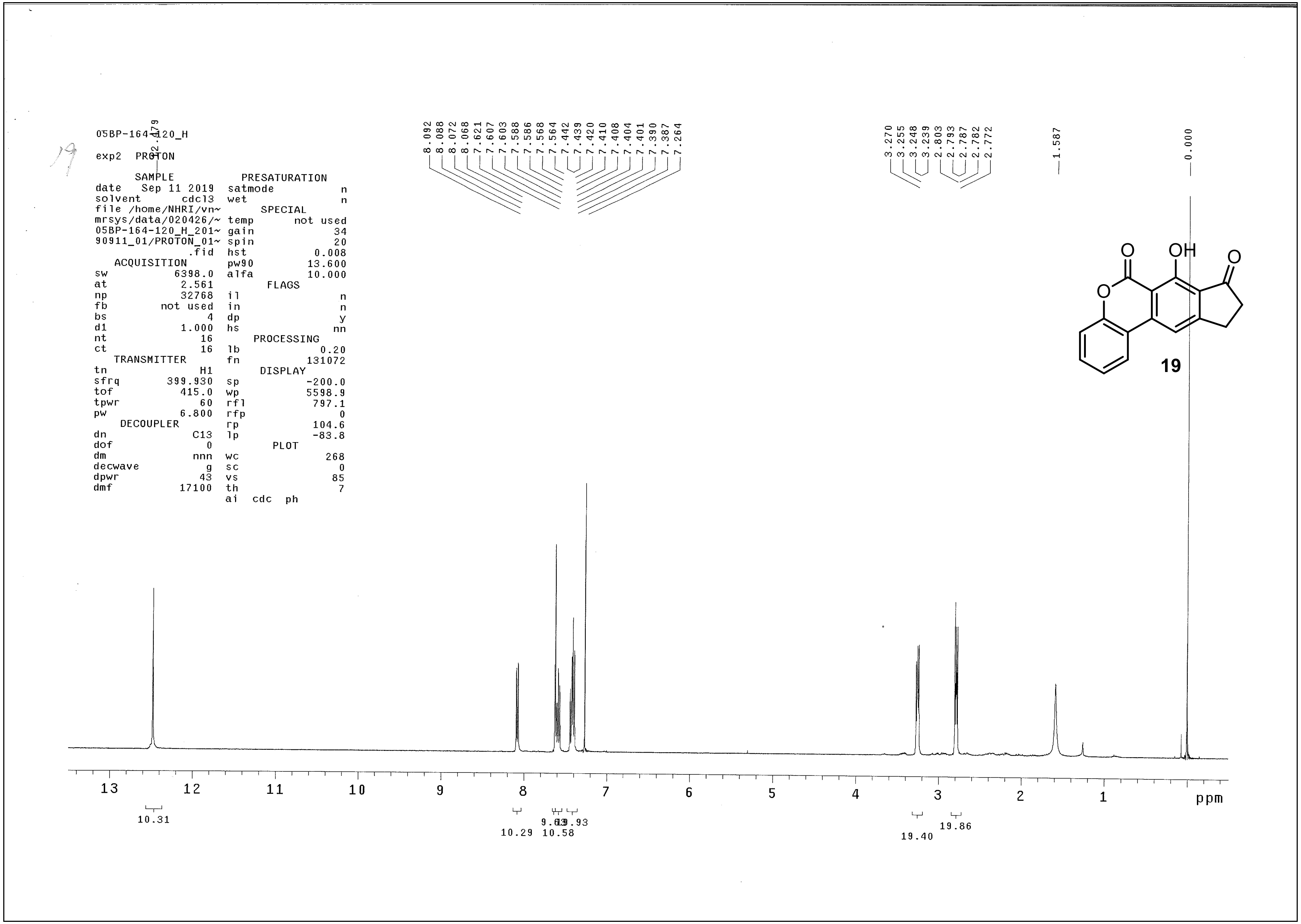

^{1}H NMR spectra for compound **19**

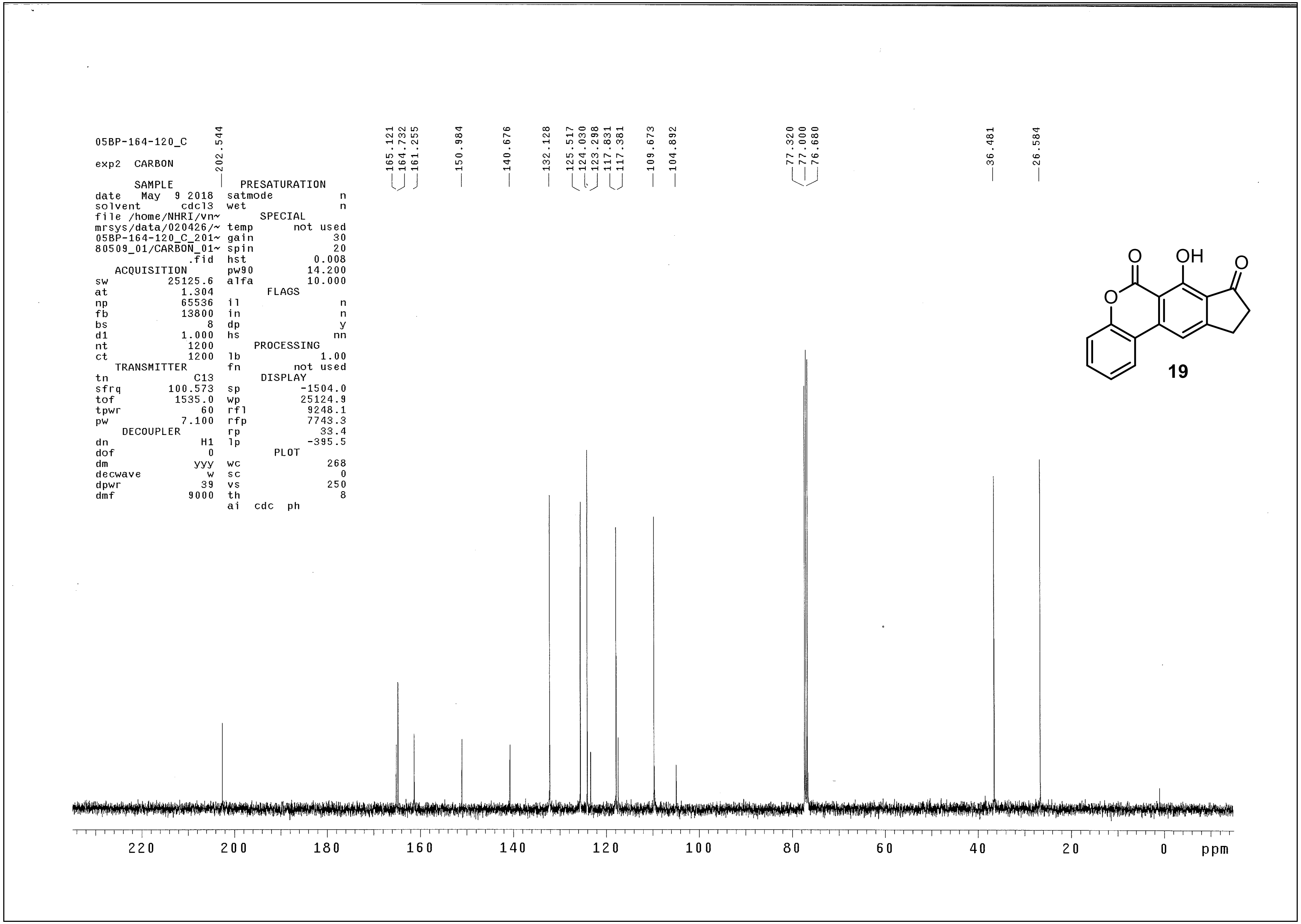

^{13}C NMR spectra for compound **19**

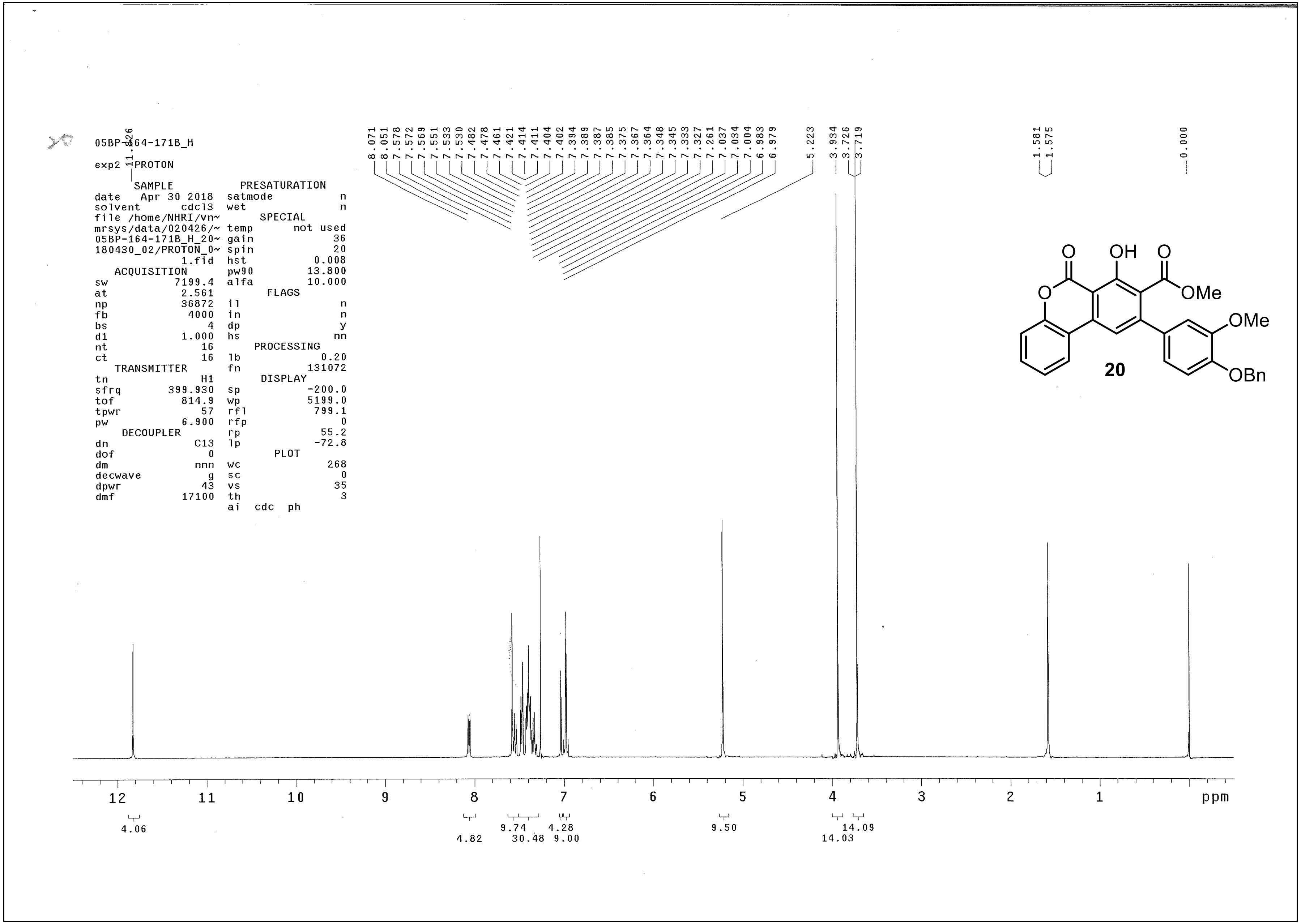

^{1}H NMR spectra for compound **20**

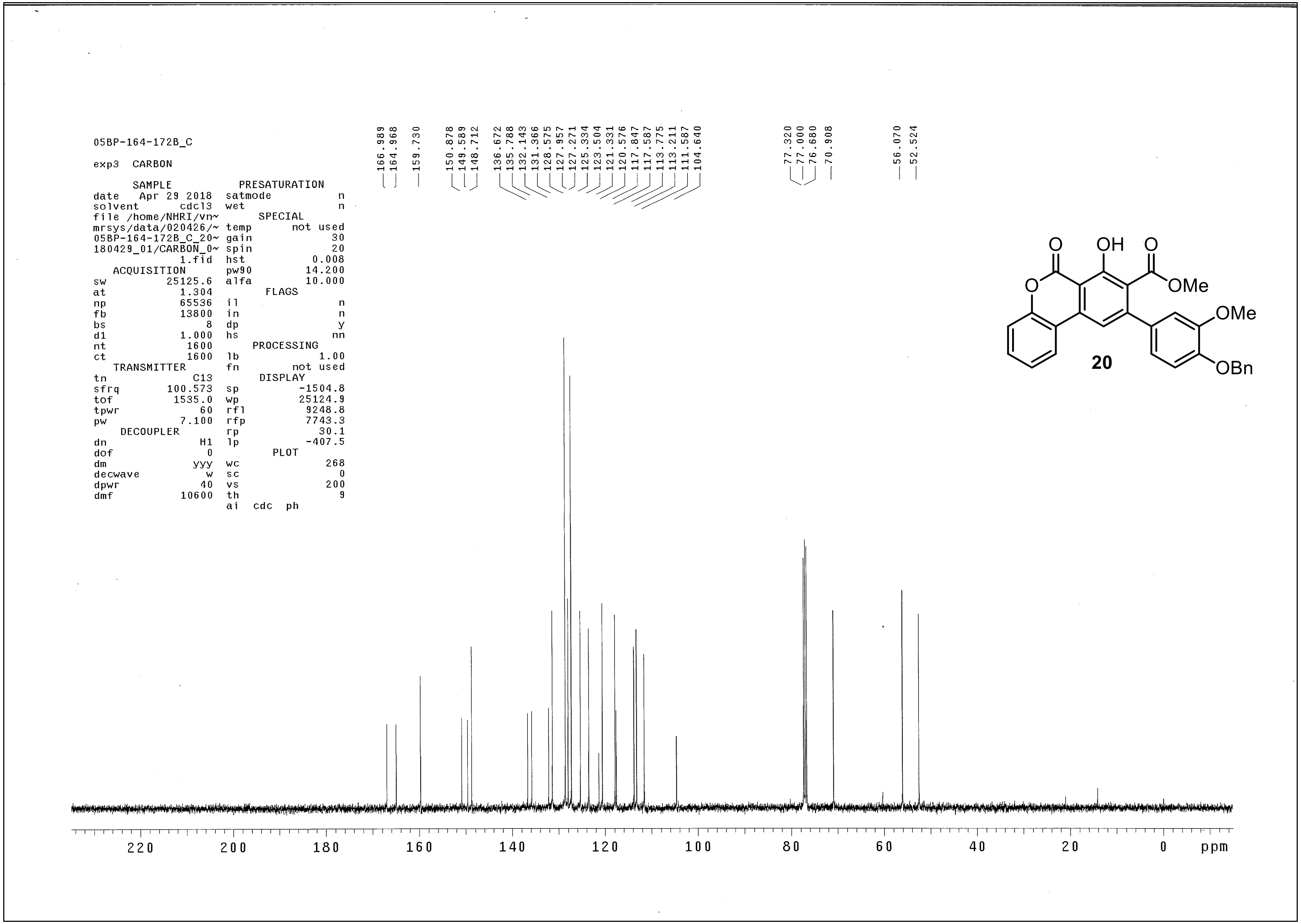

^{13}C NMR spectra for compound **20**

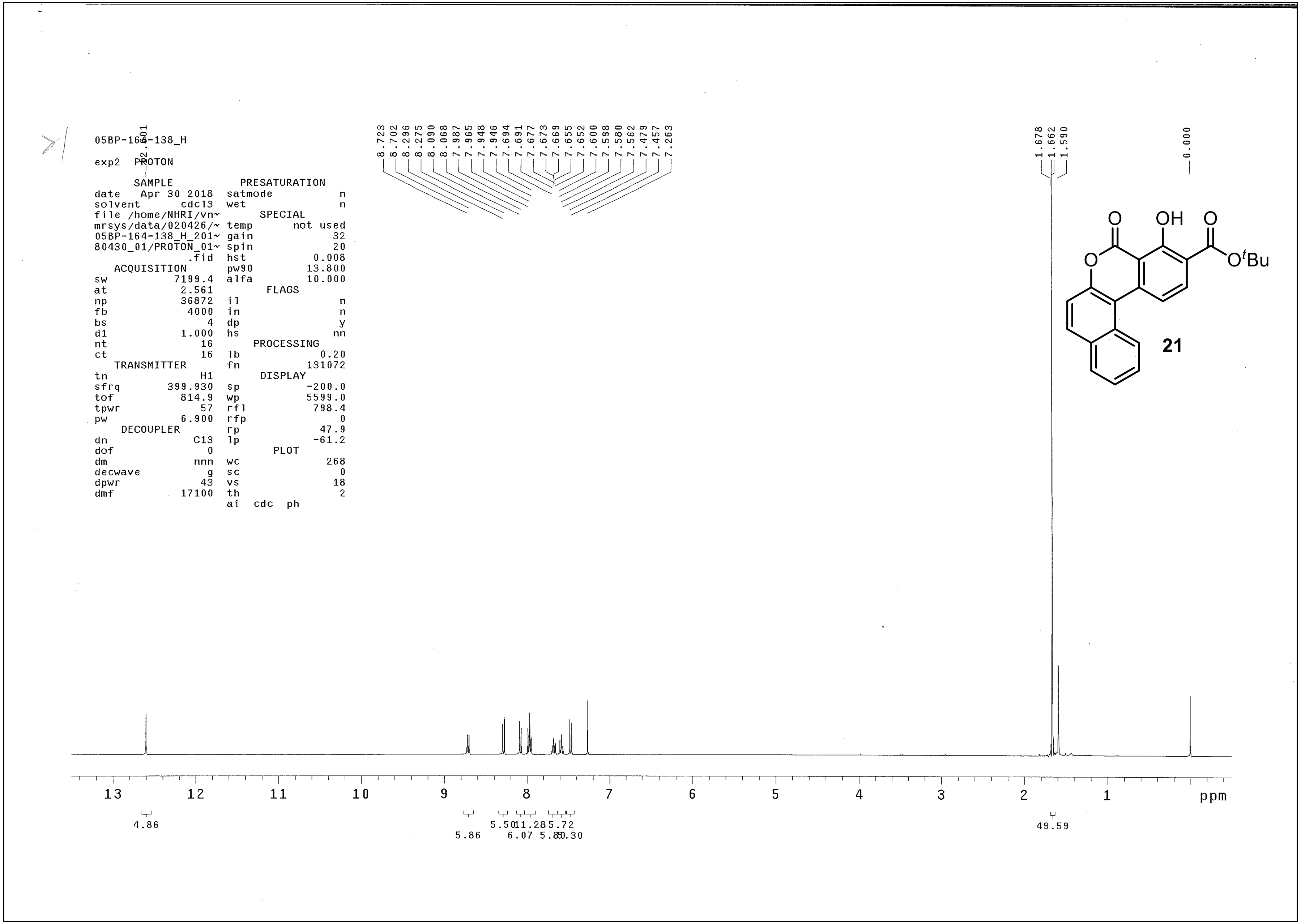

^{1}H NMR spectra for compound **21**

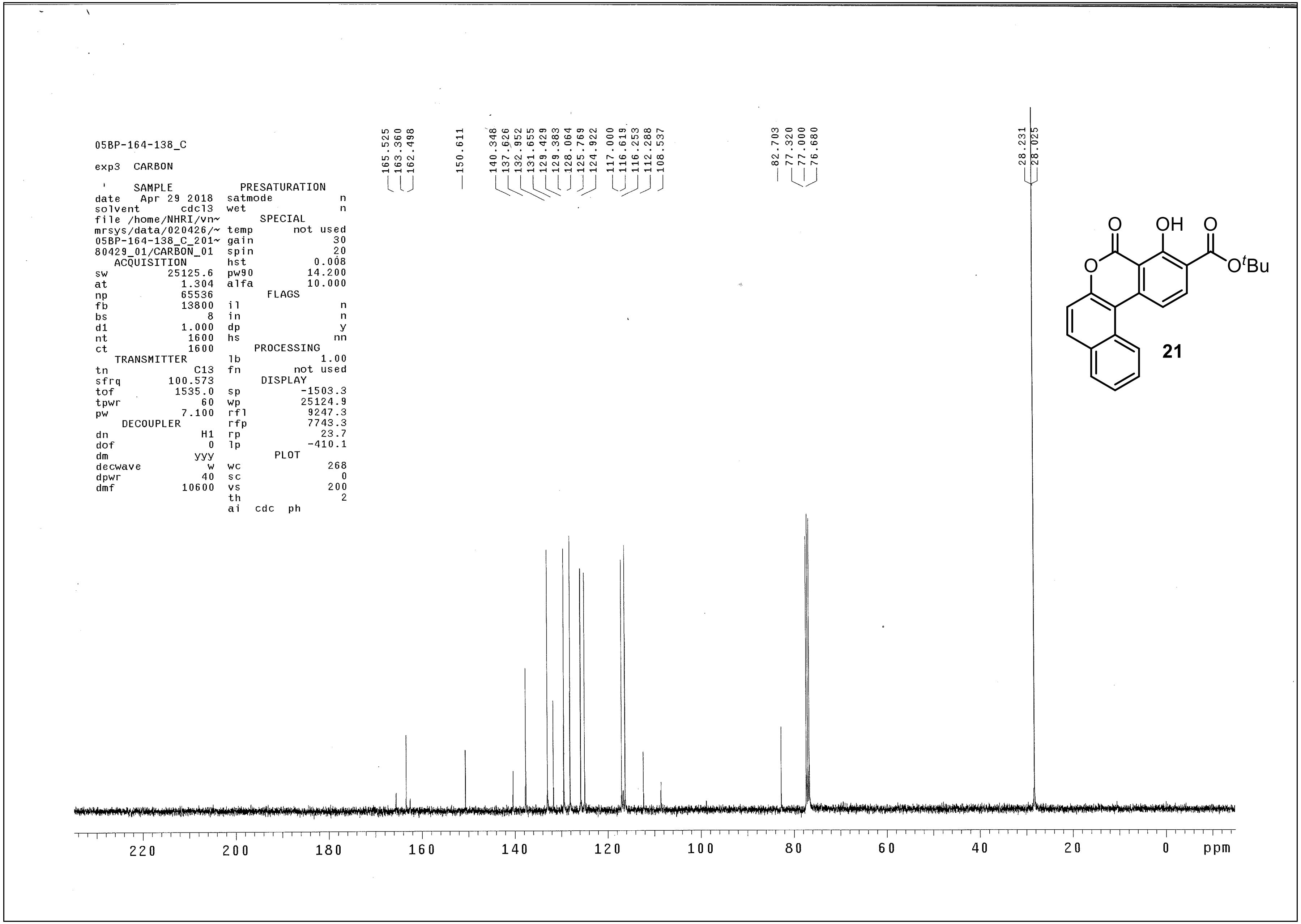

^{13}C NMR spectra for compound **21**

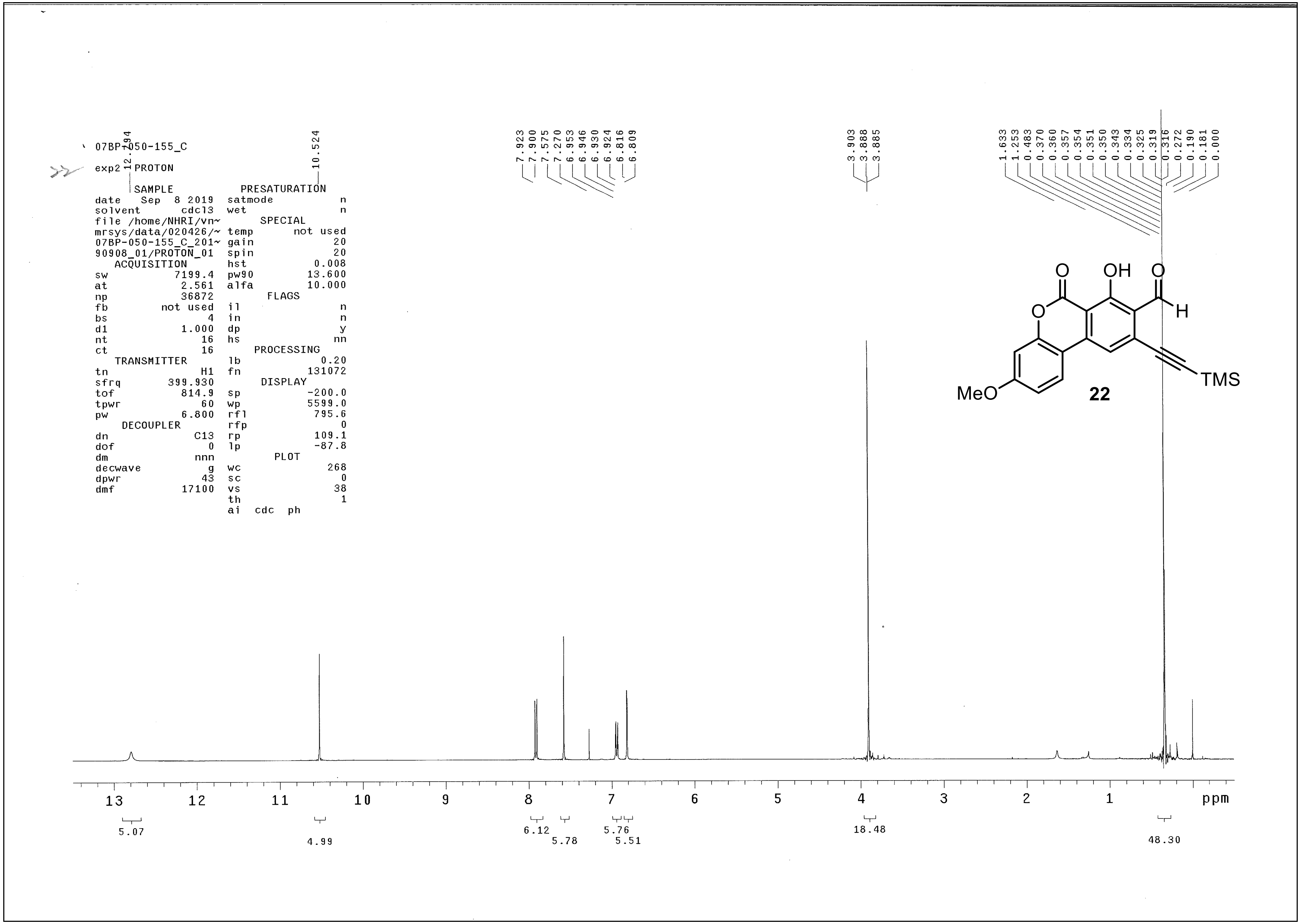

^{1}H NMR spectra for compound **22**

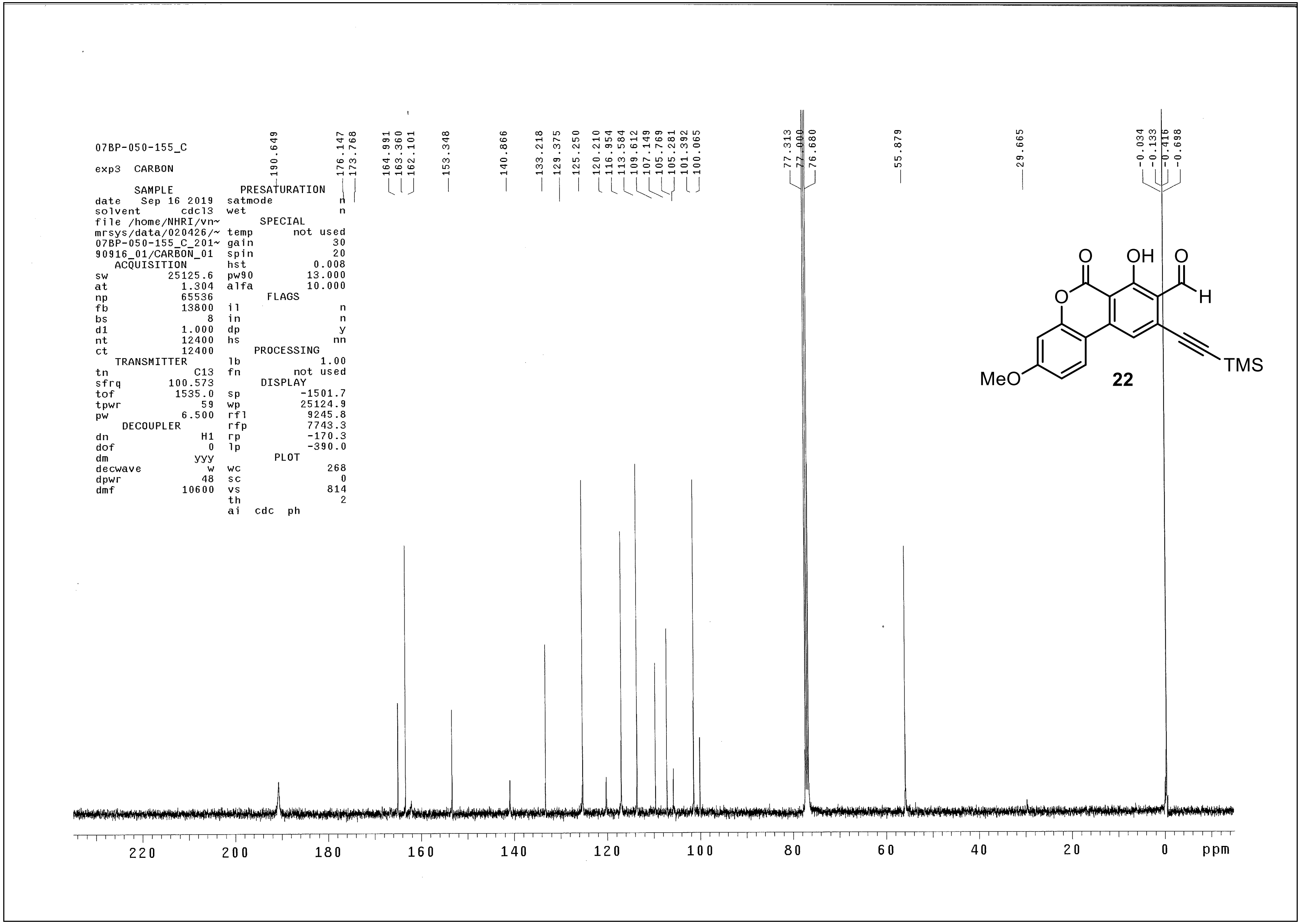

^{13}C NMR spectra for compound **22**

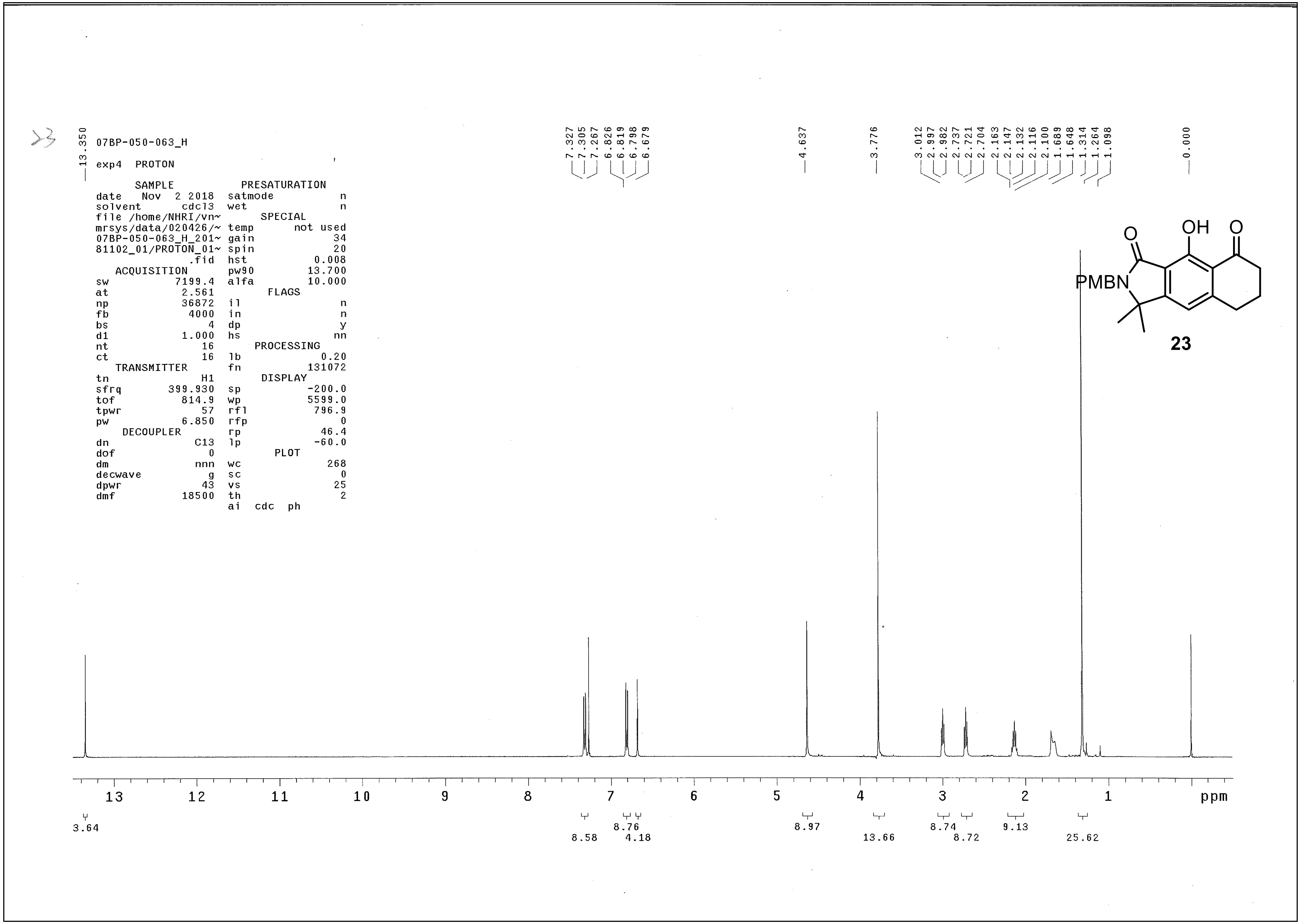

^{1}H NMR spectra for compound **23**

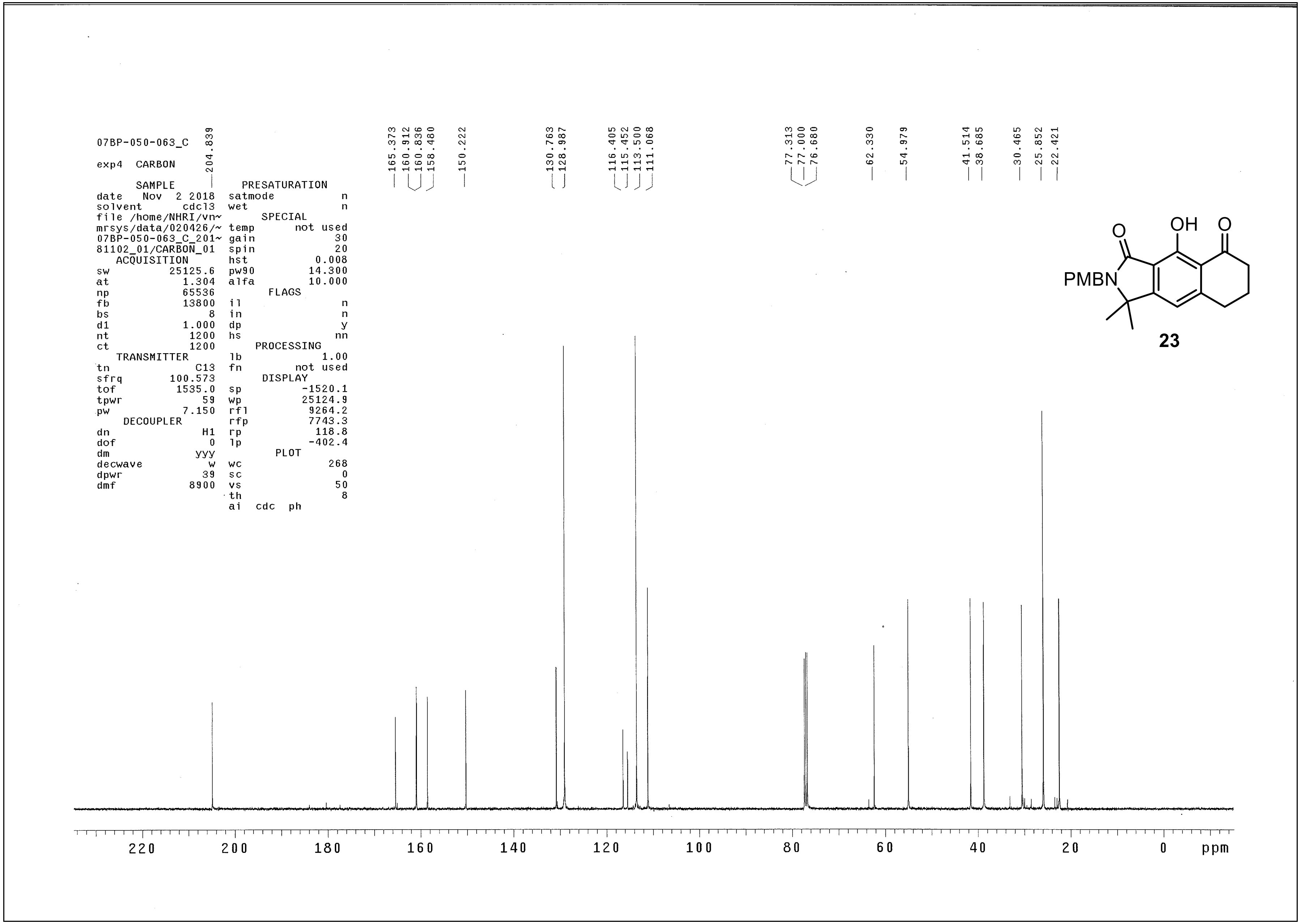

^{13}C NMR spectra for compound **23**

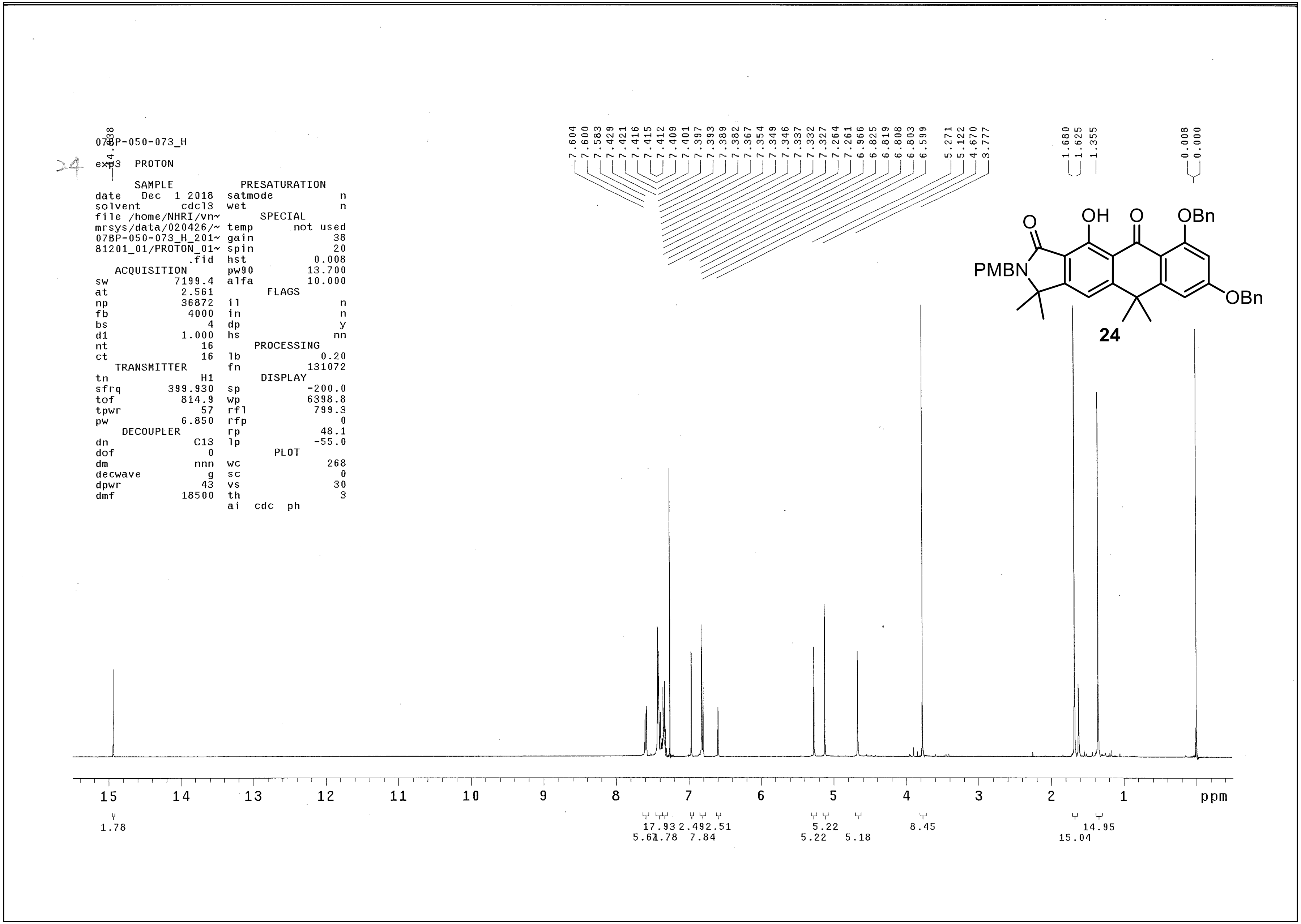

^{1}H NMR spectra for compound **24**

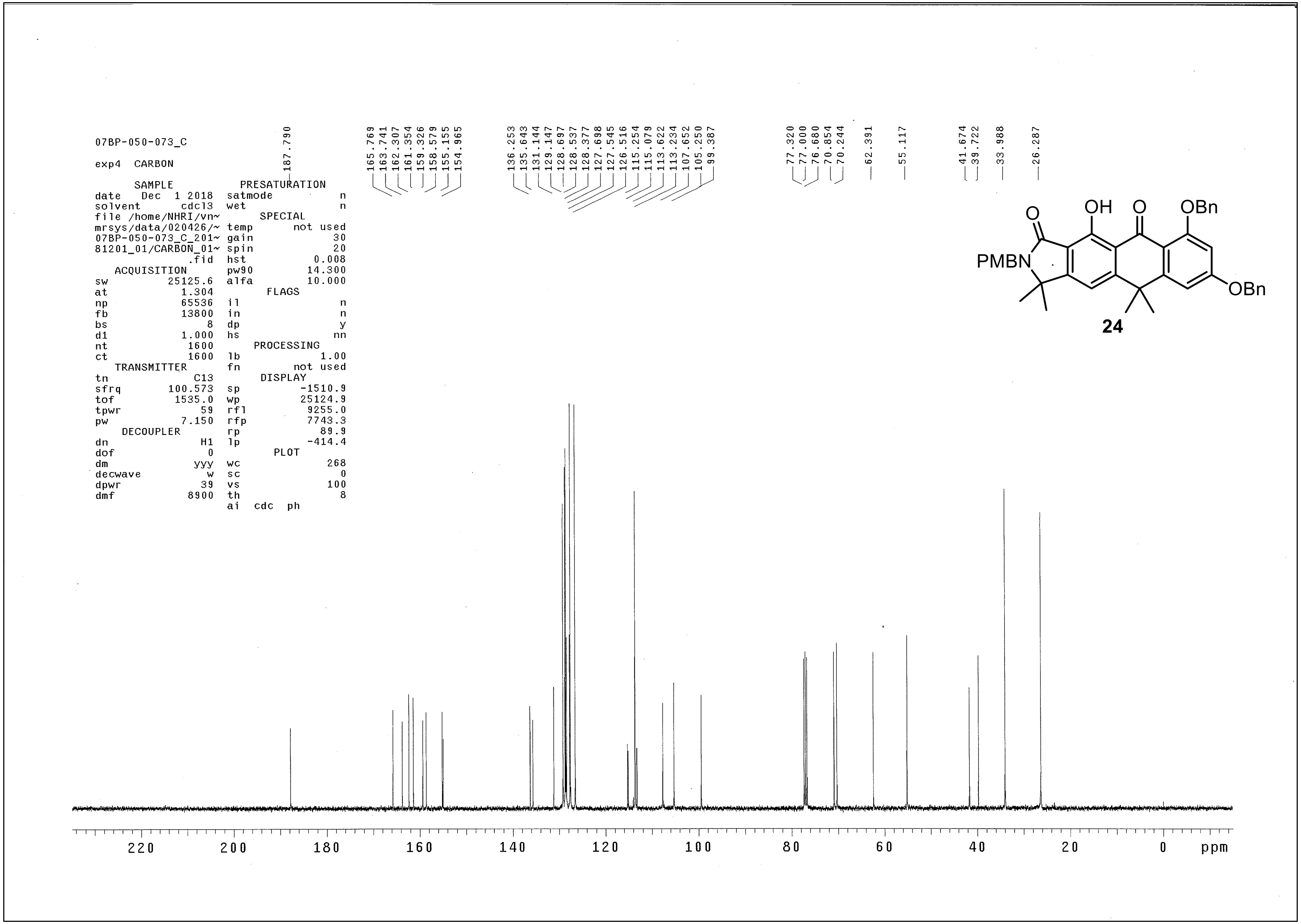

^{13}C NMR spectra for compound **24**

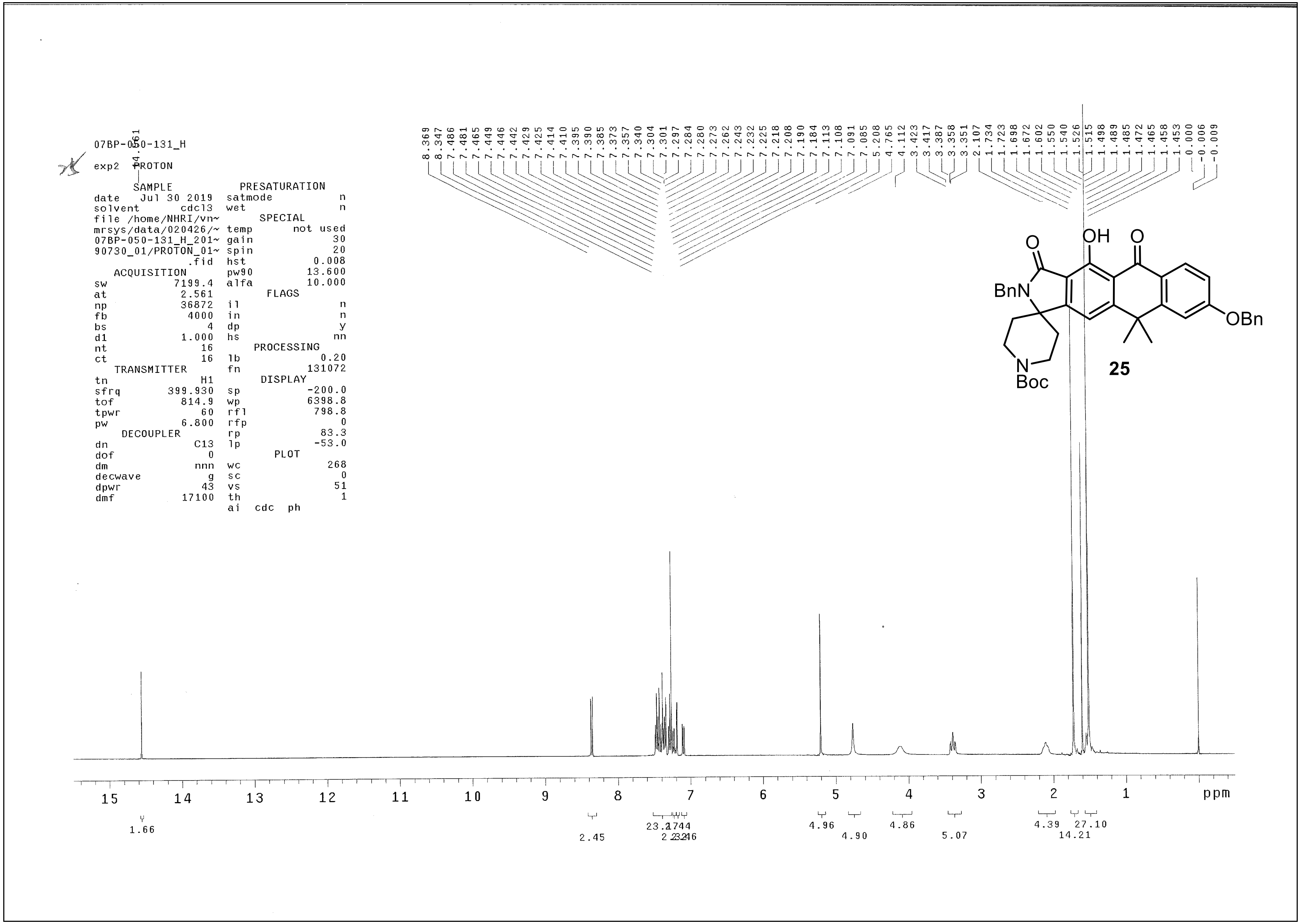

^{1}H NMR spectra for compound **25**

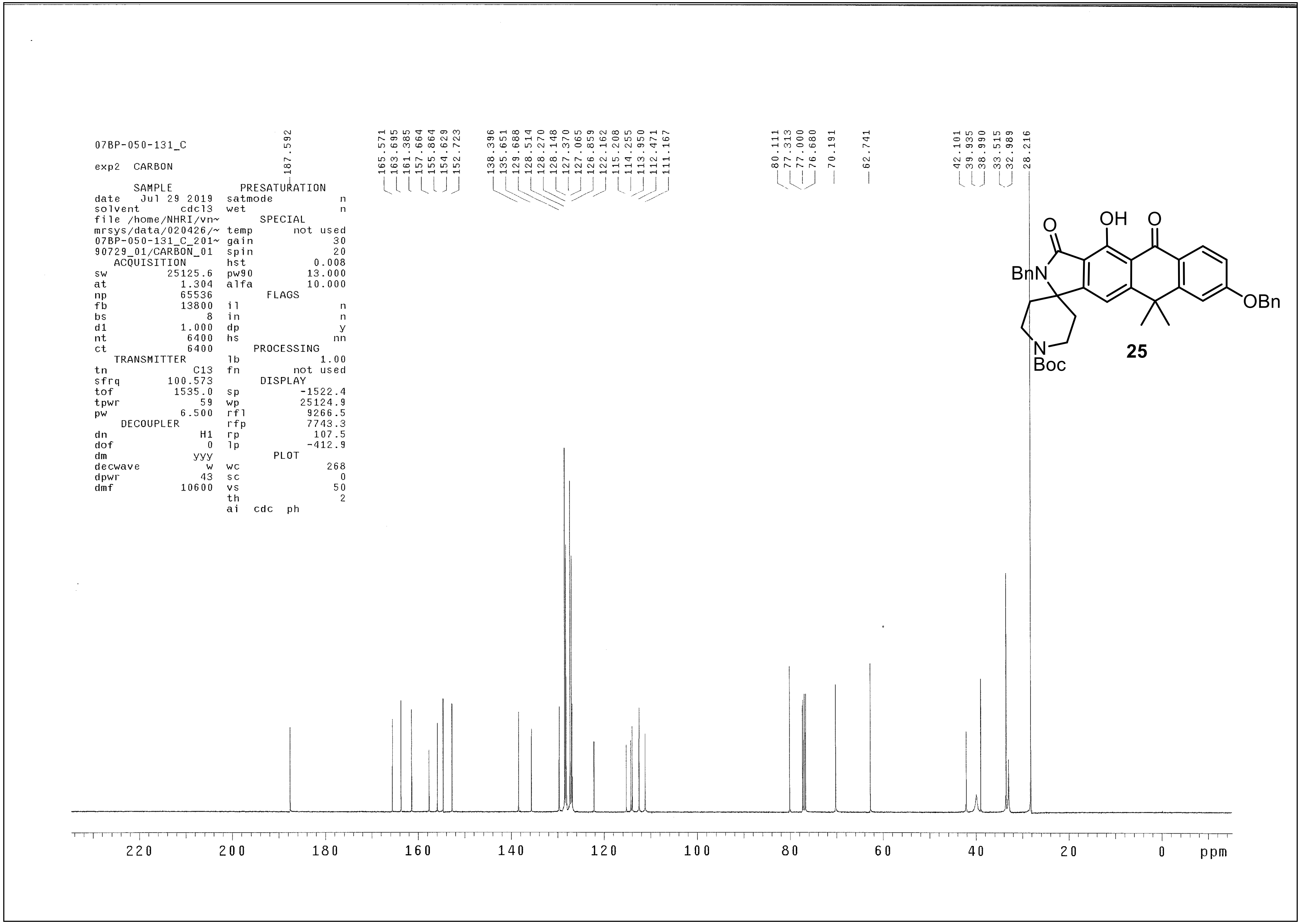

^{13}C NMR spectra for compound **25**

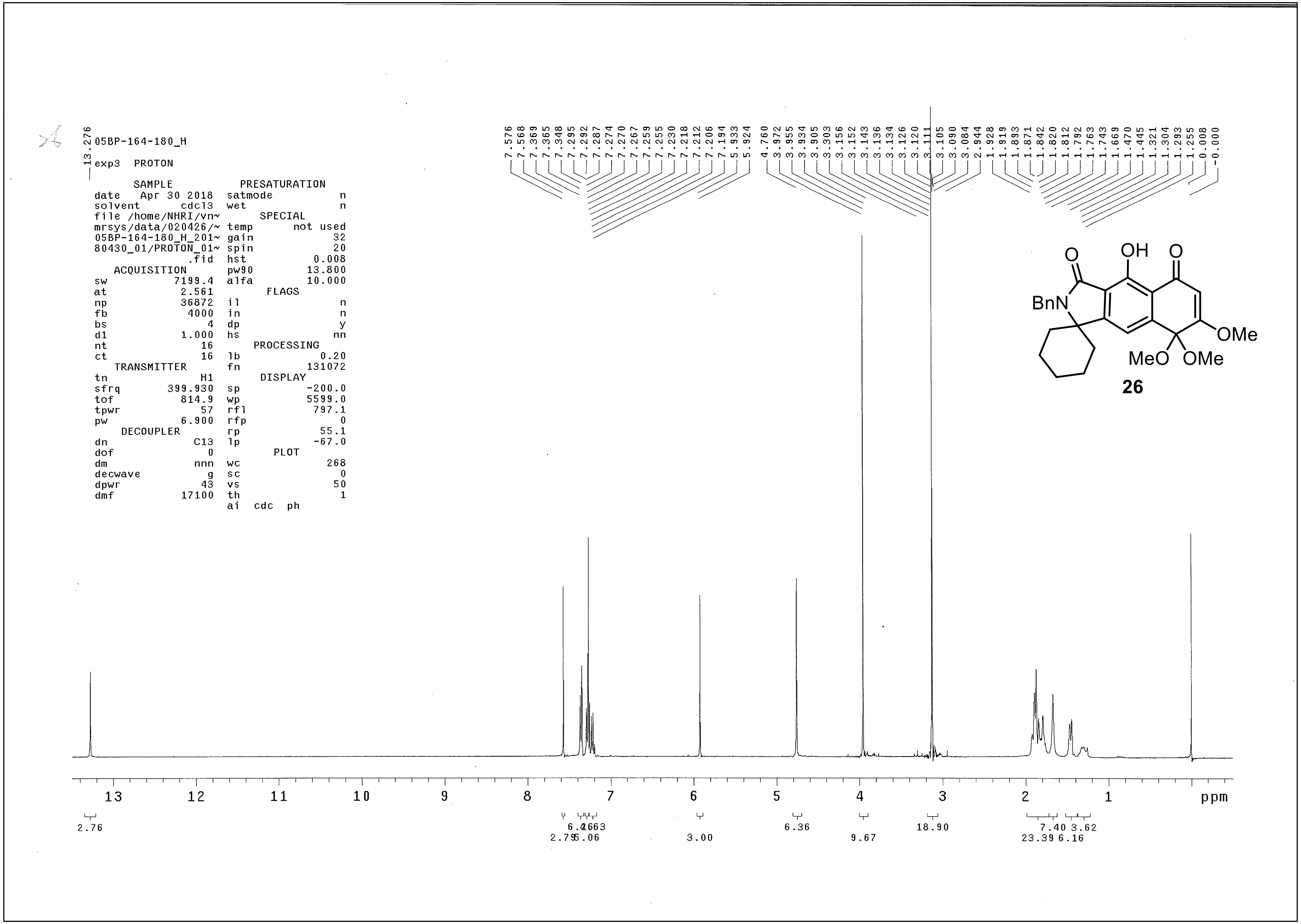

^{1}H NMR spectra for compound **26**

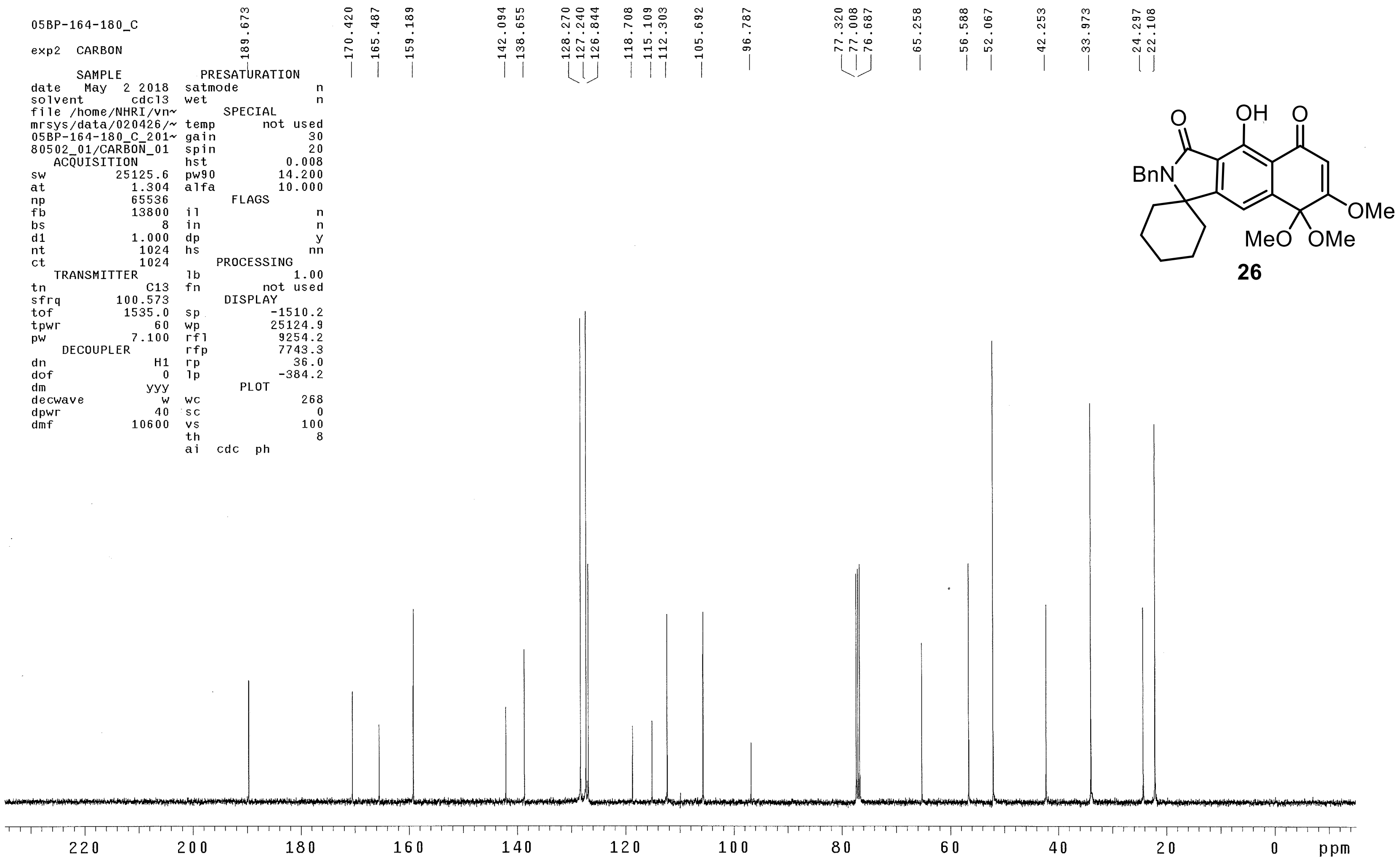

^{13}C NMR spectra for compound **26**

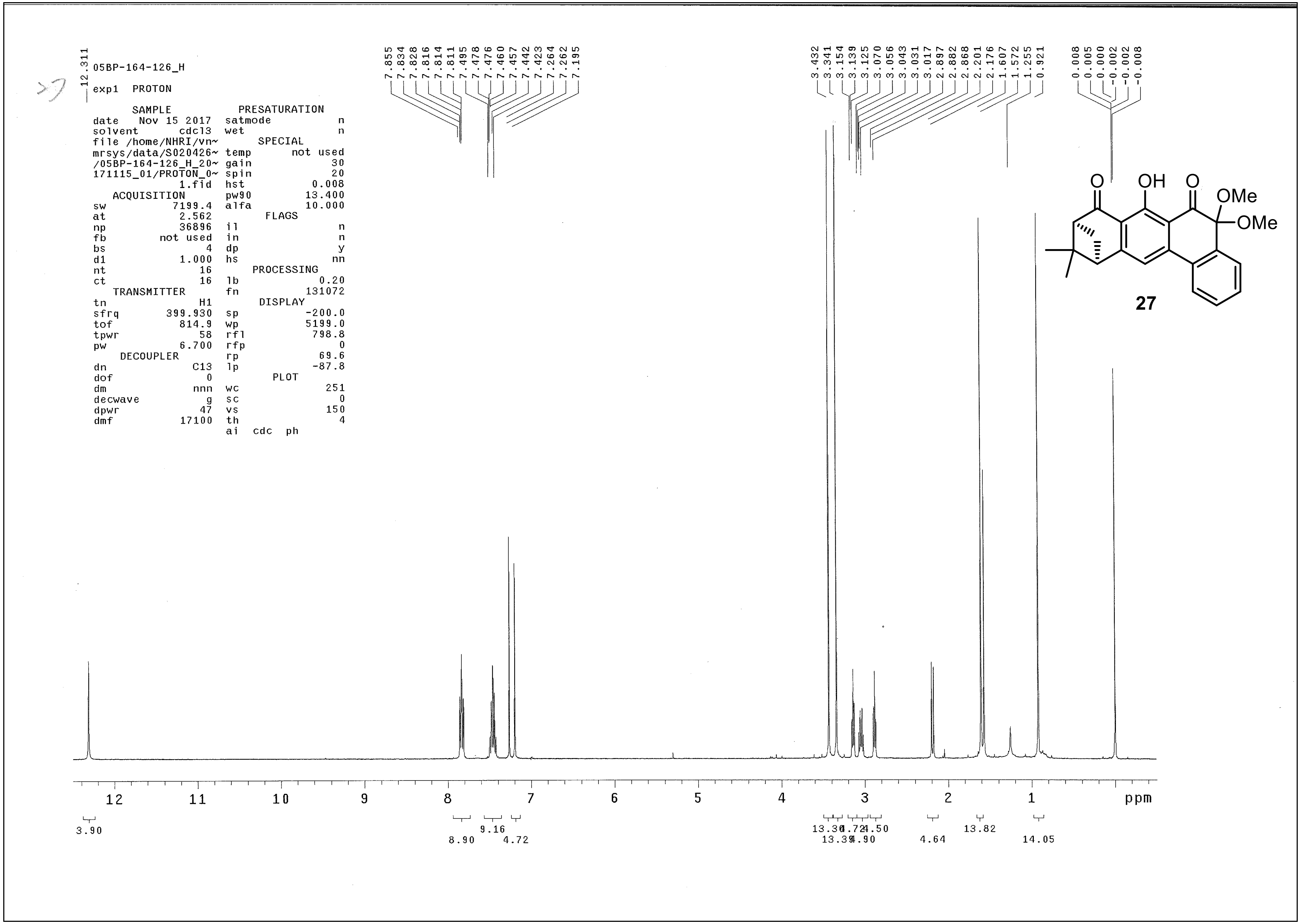

^{1}H NMR spectra for compound **27**

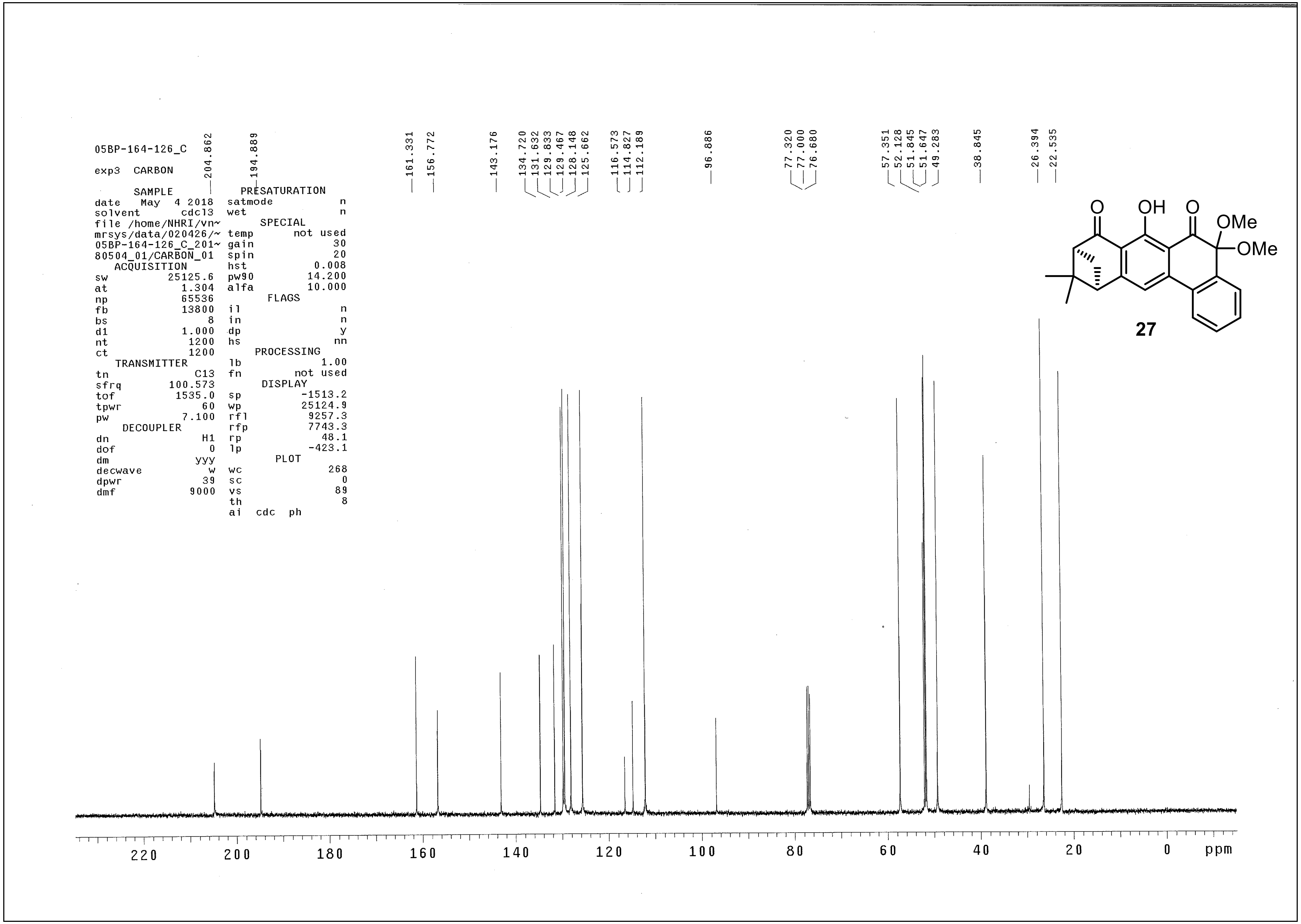

^{13}C NMR spectra for compound **27**

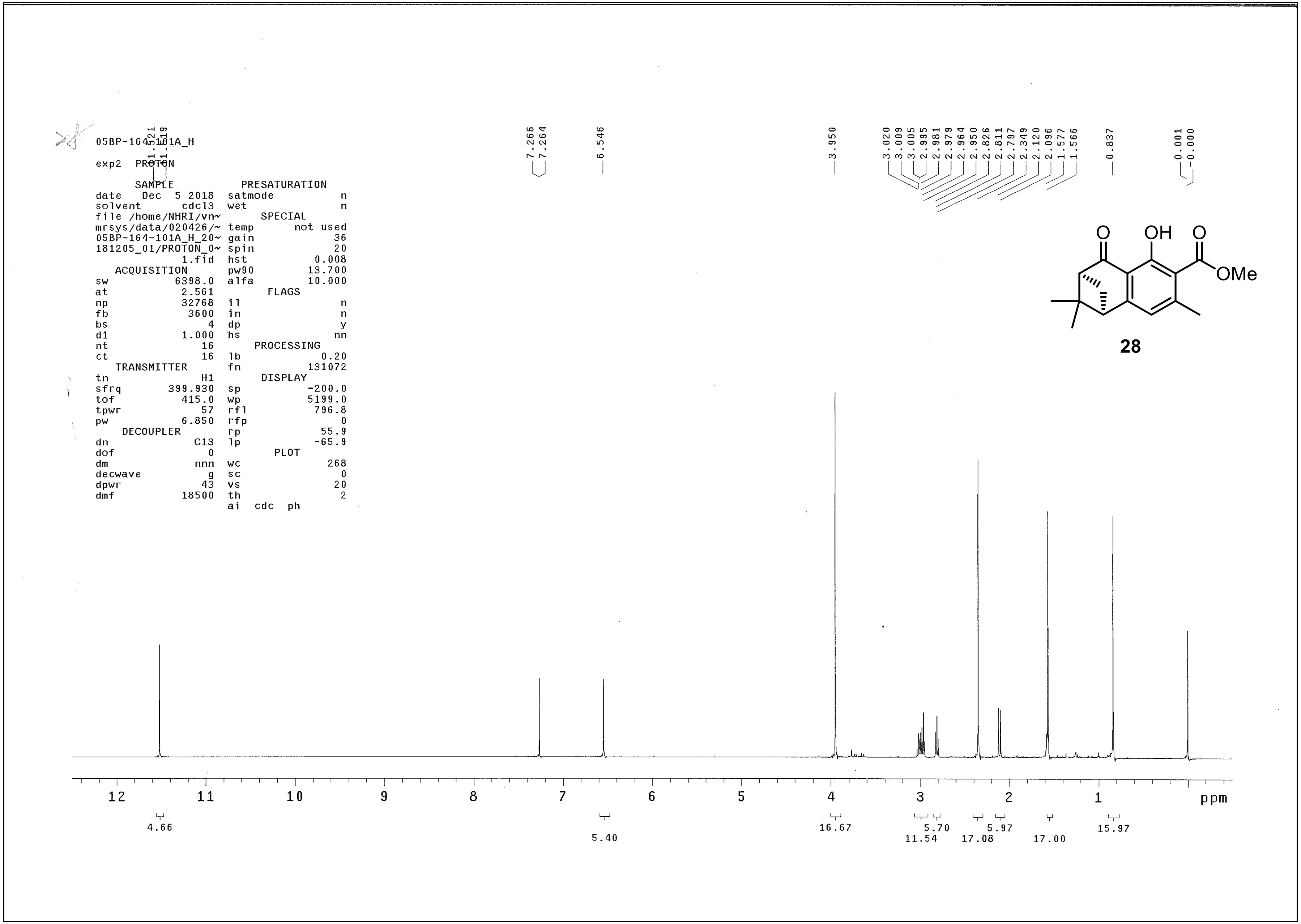

^{1}H NMR spectra for compound **28**

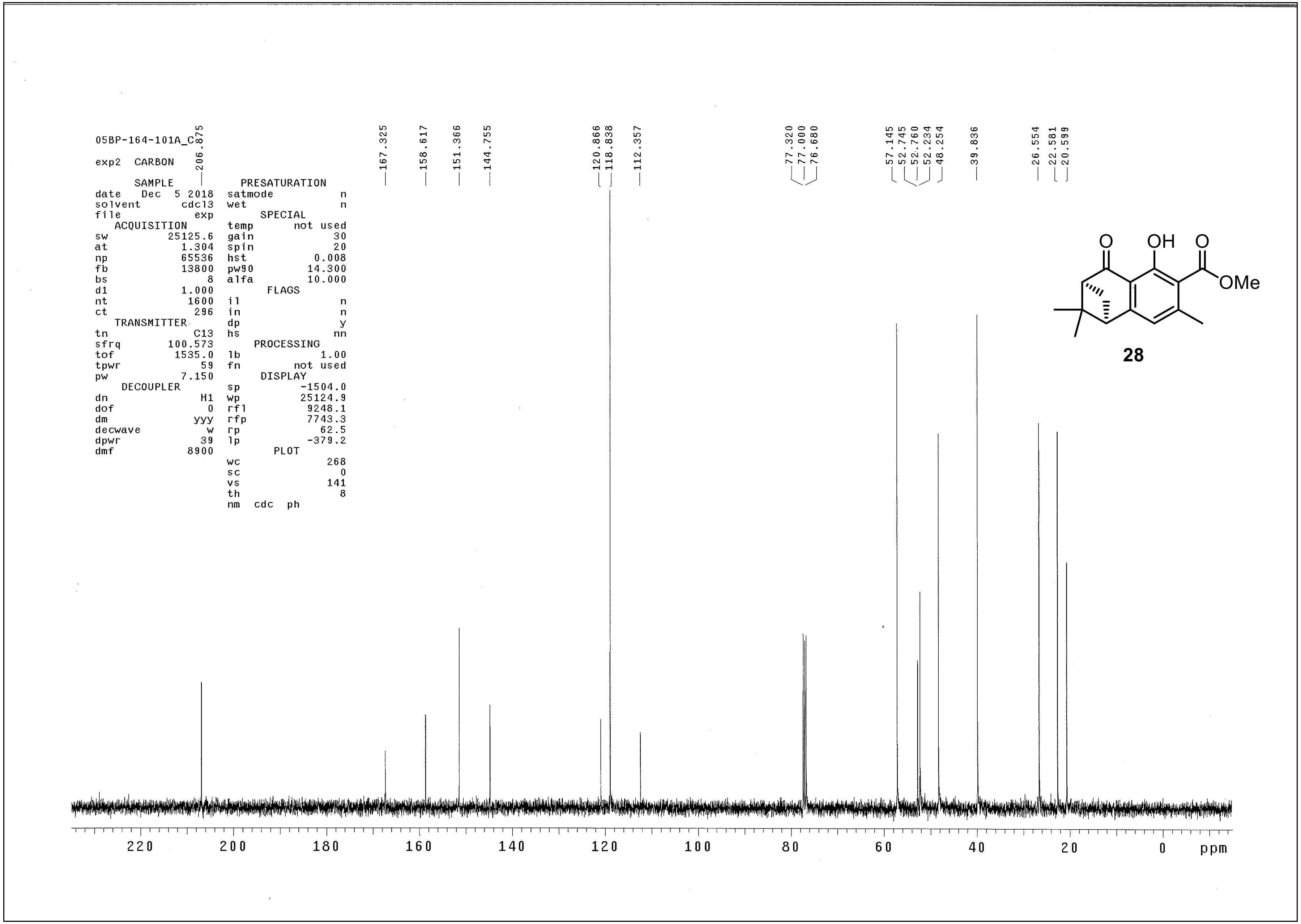

^{13}C NMR spectra for compound **28**

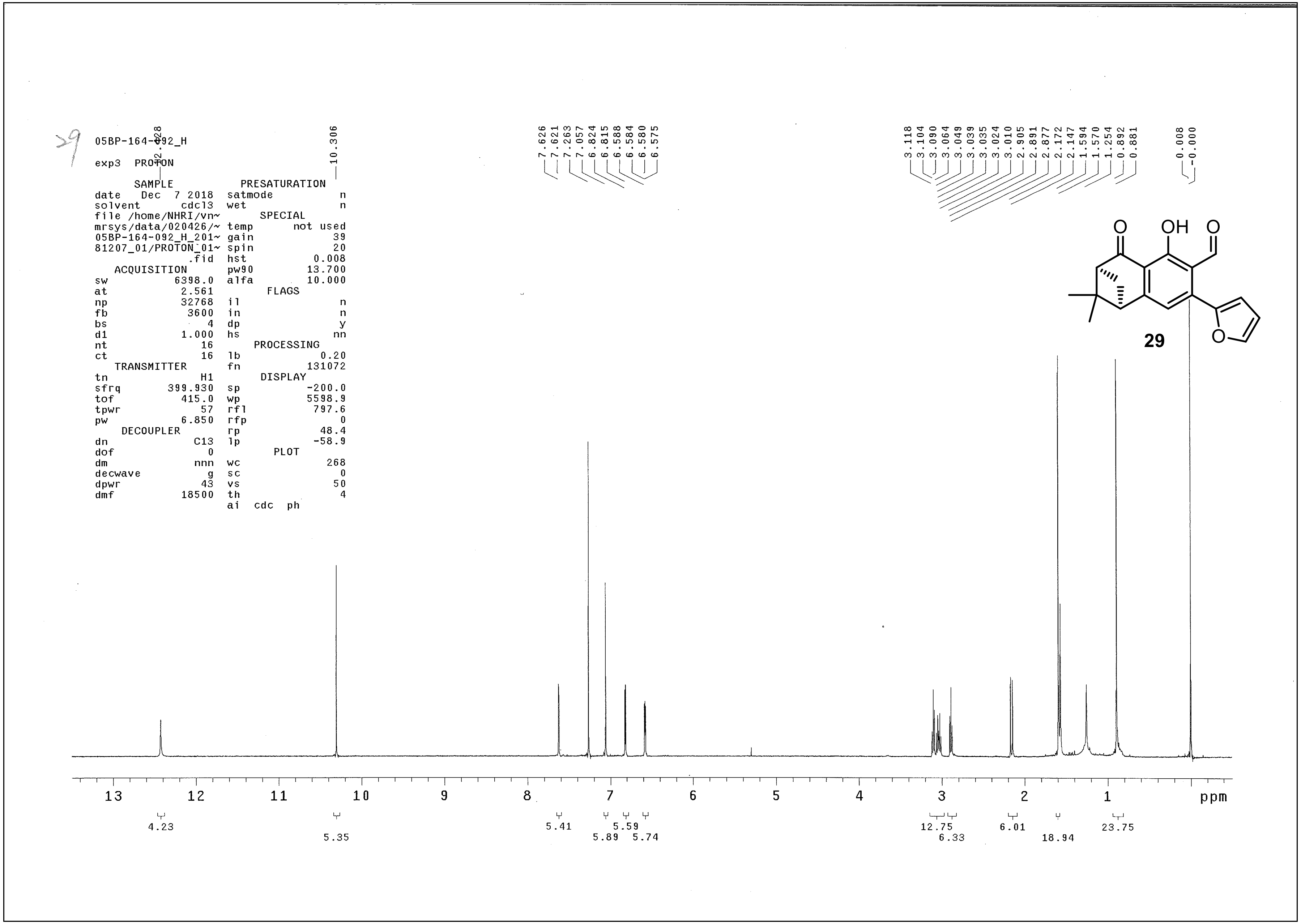

^{1}H NMR spectra for compound **29**

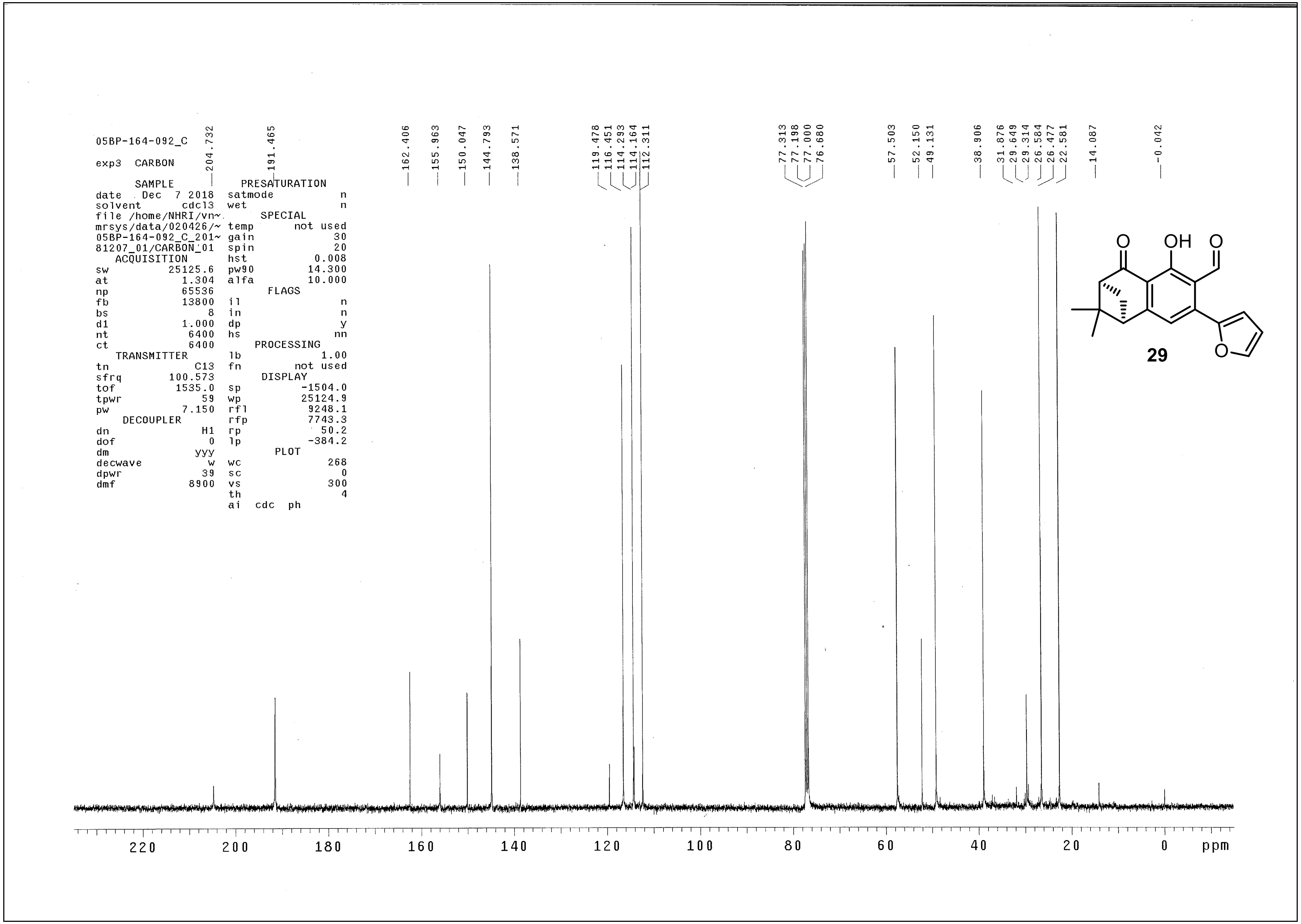

^{13}C NMR spectra for compound **29**

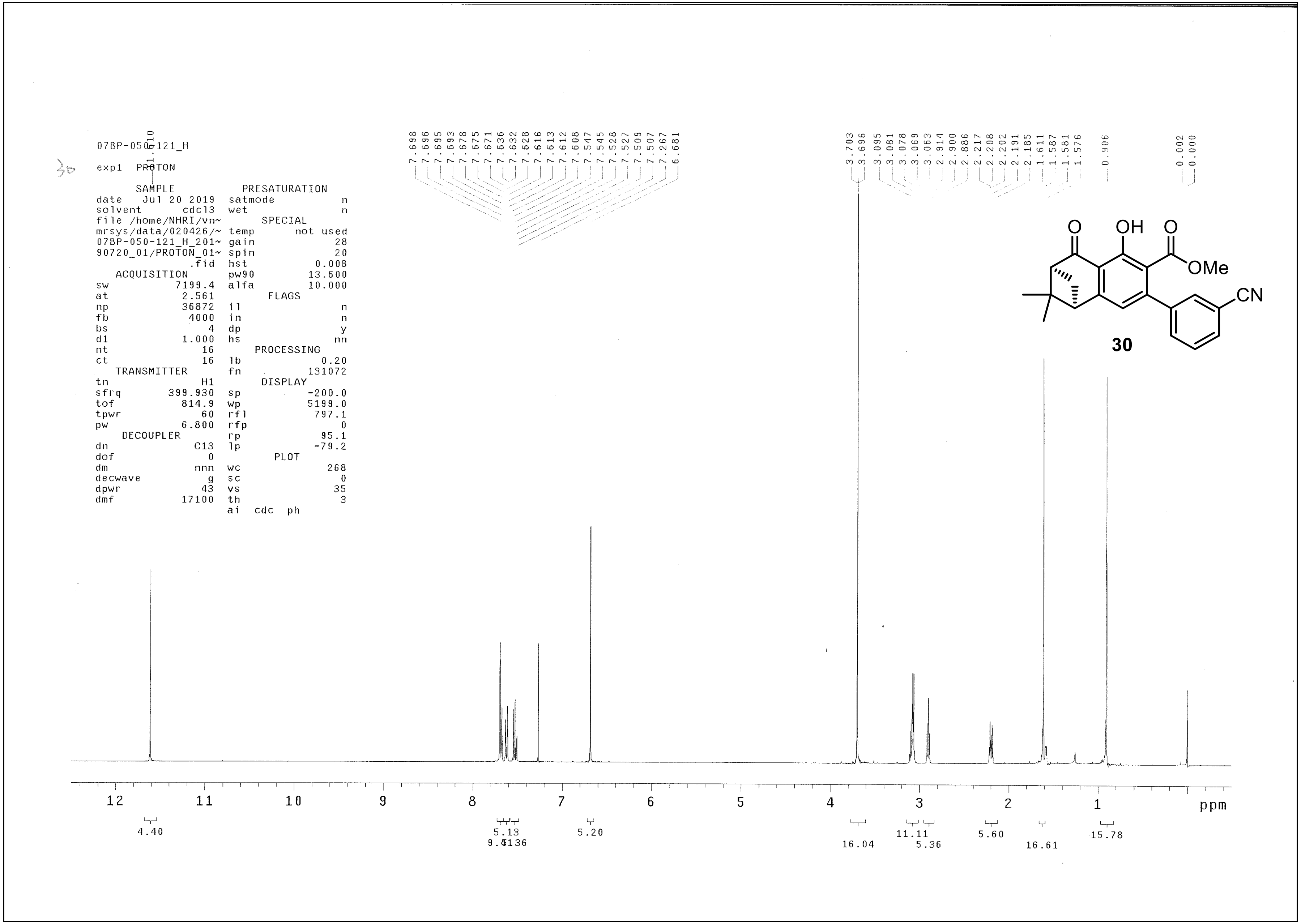

^{1}H NMR spectra for compound **30**

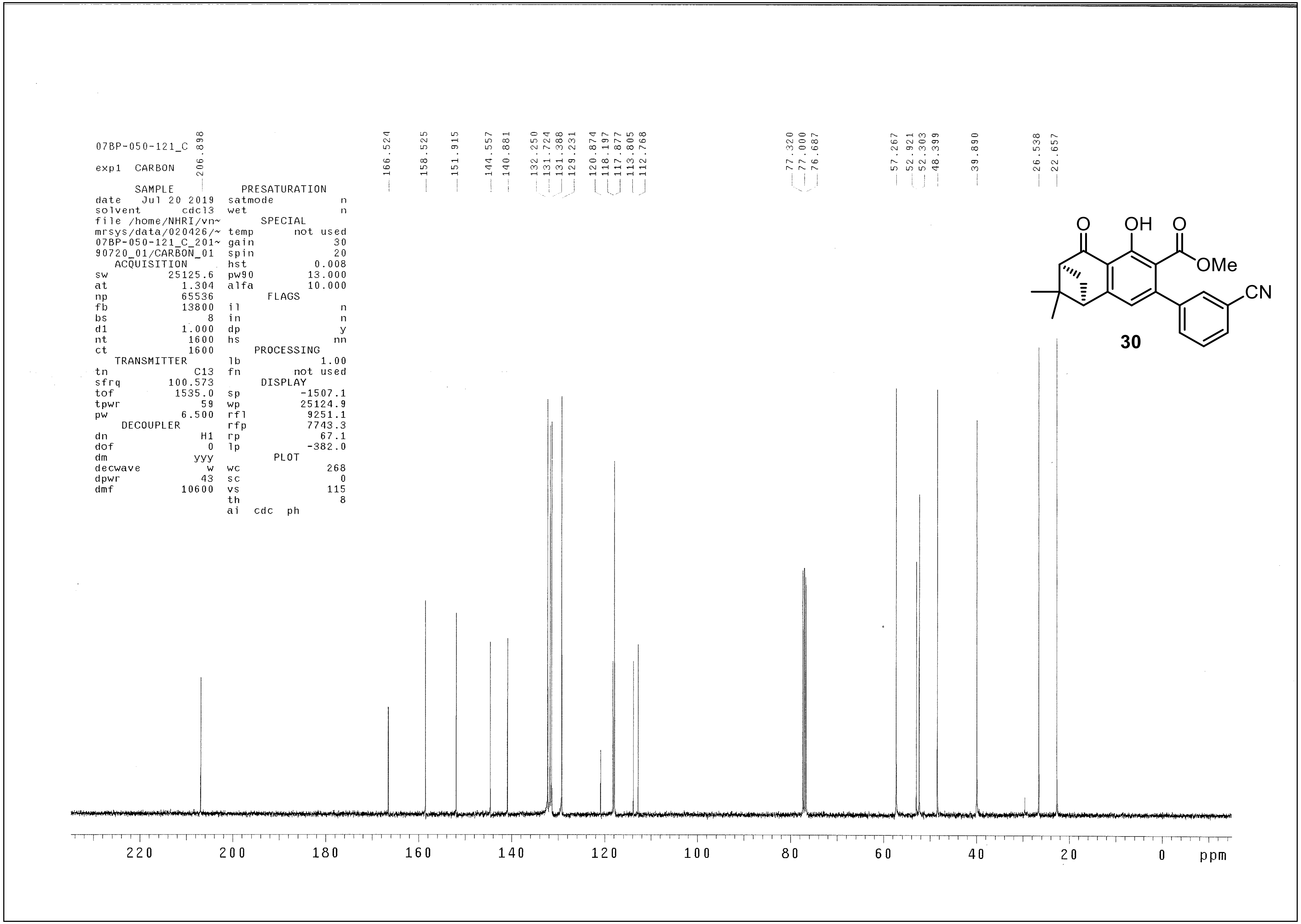

^{13}C NMR spectra for compound **30**

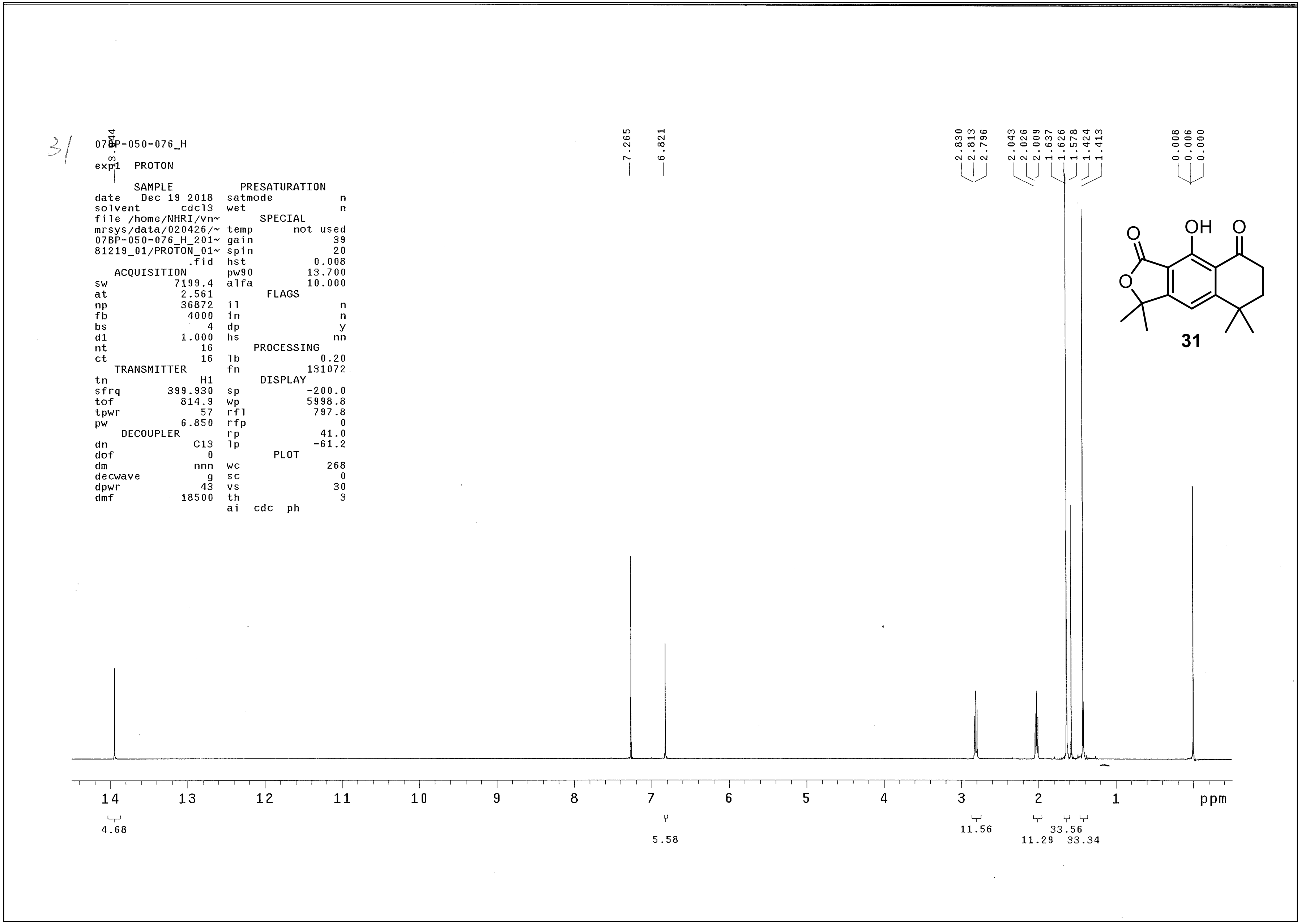

^{1}H NMR spectra for compound **31**

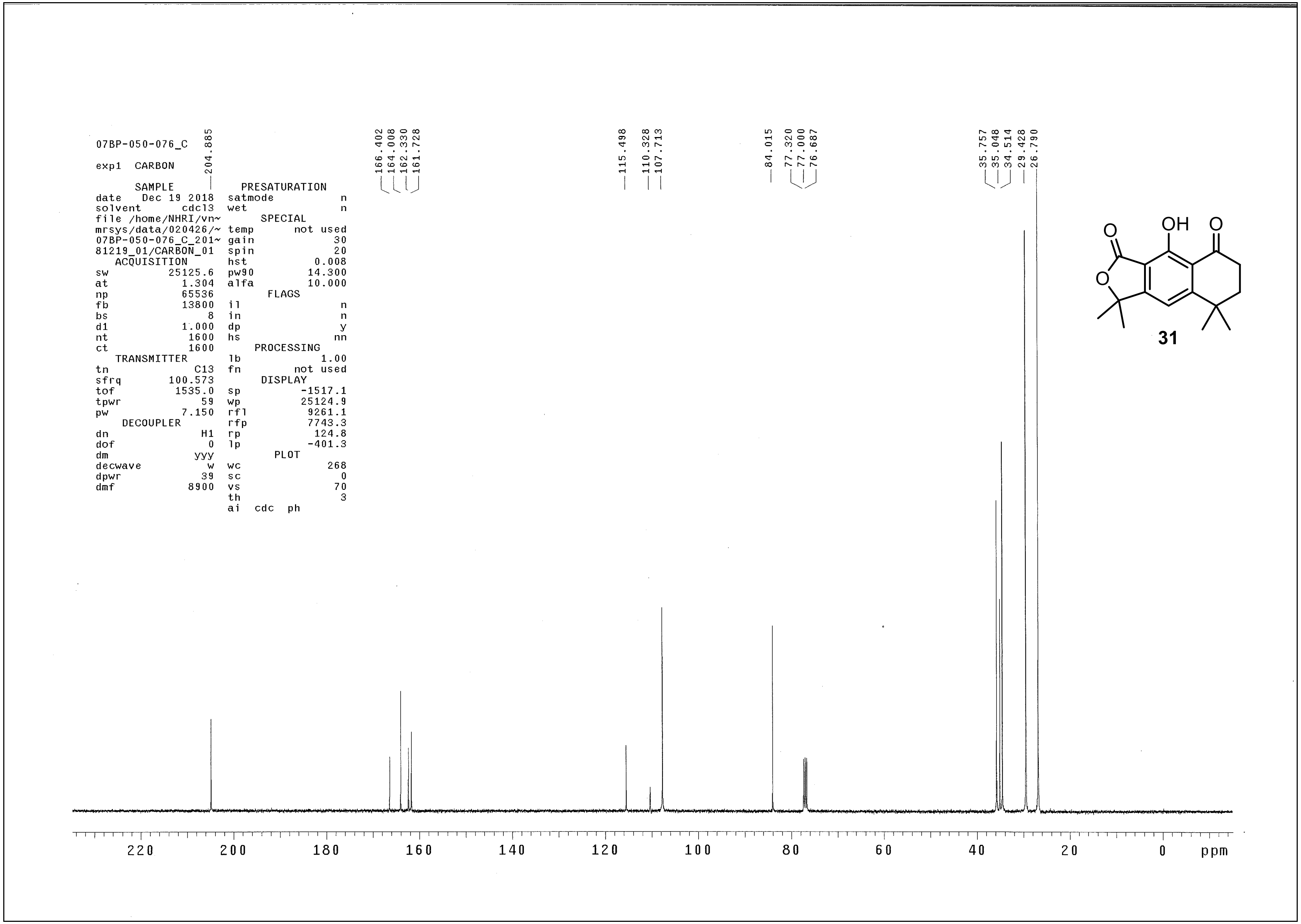

^{13}C NMR spectra for compound **31**

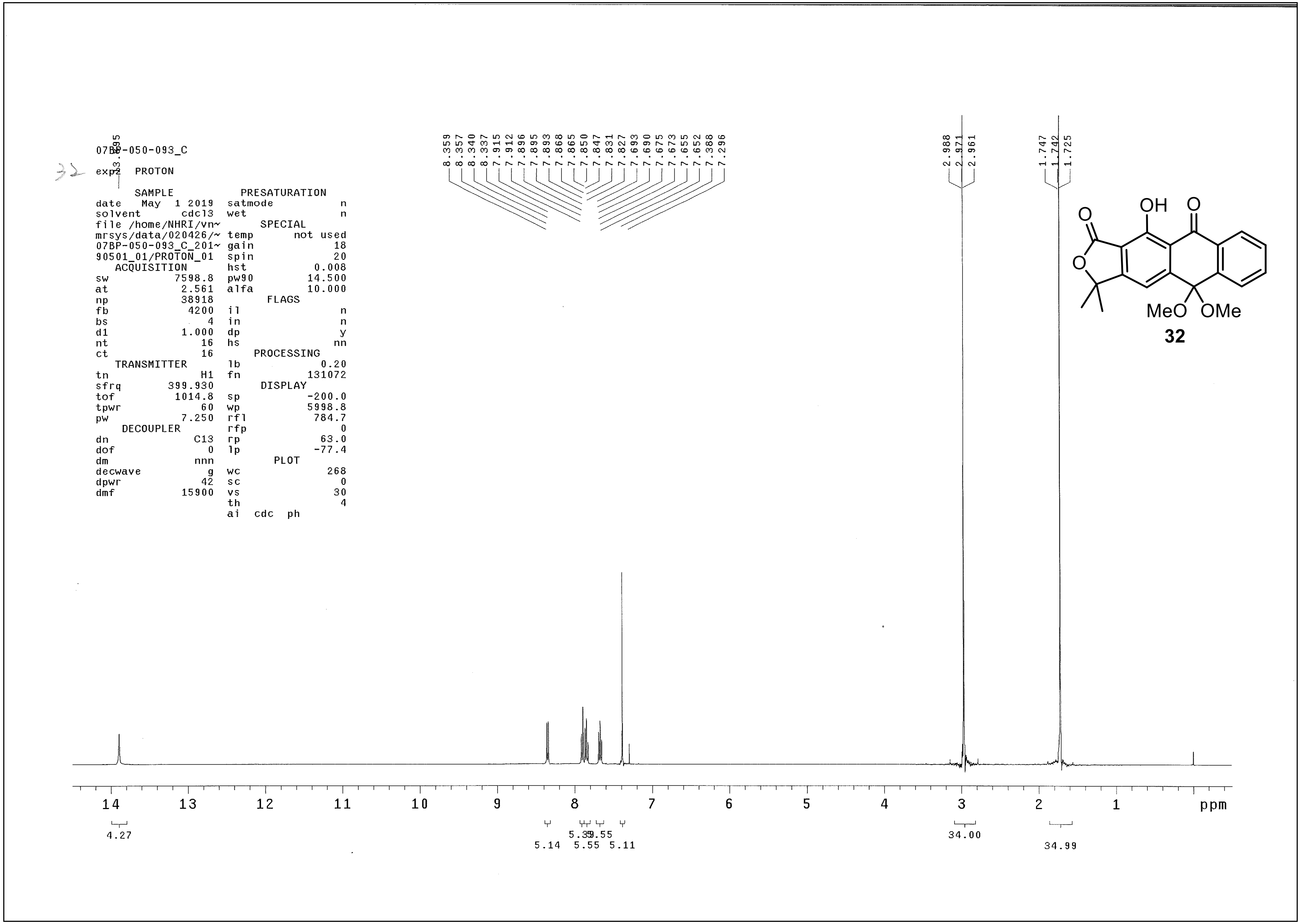

^{1}H NMR spectra for compound **32**

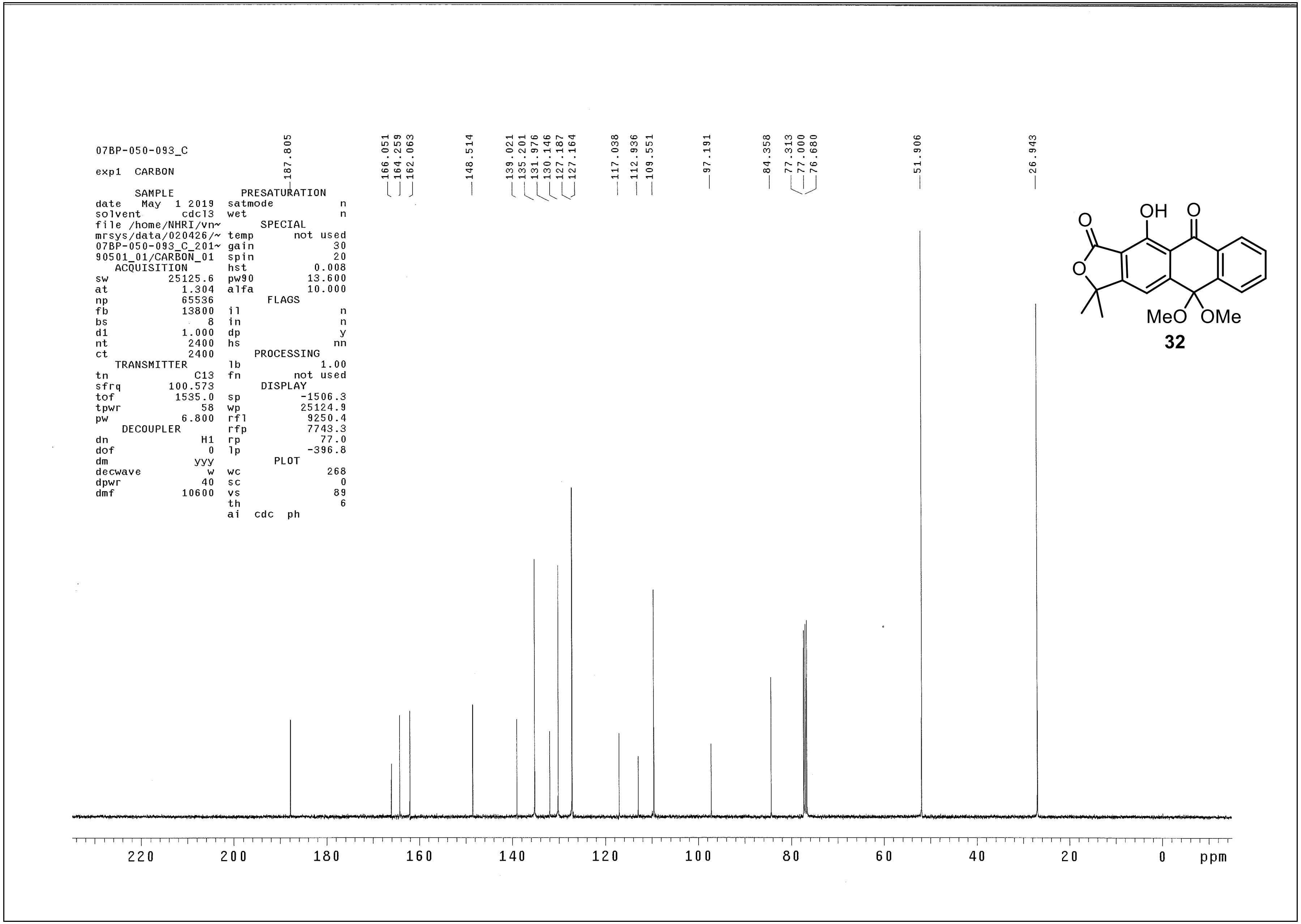

^{13}C NMR spectra for compound **32**

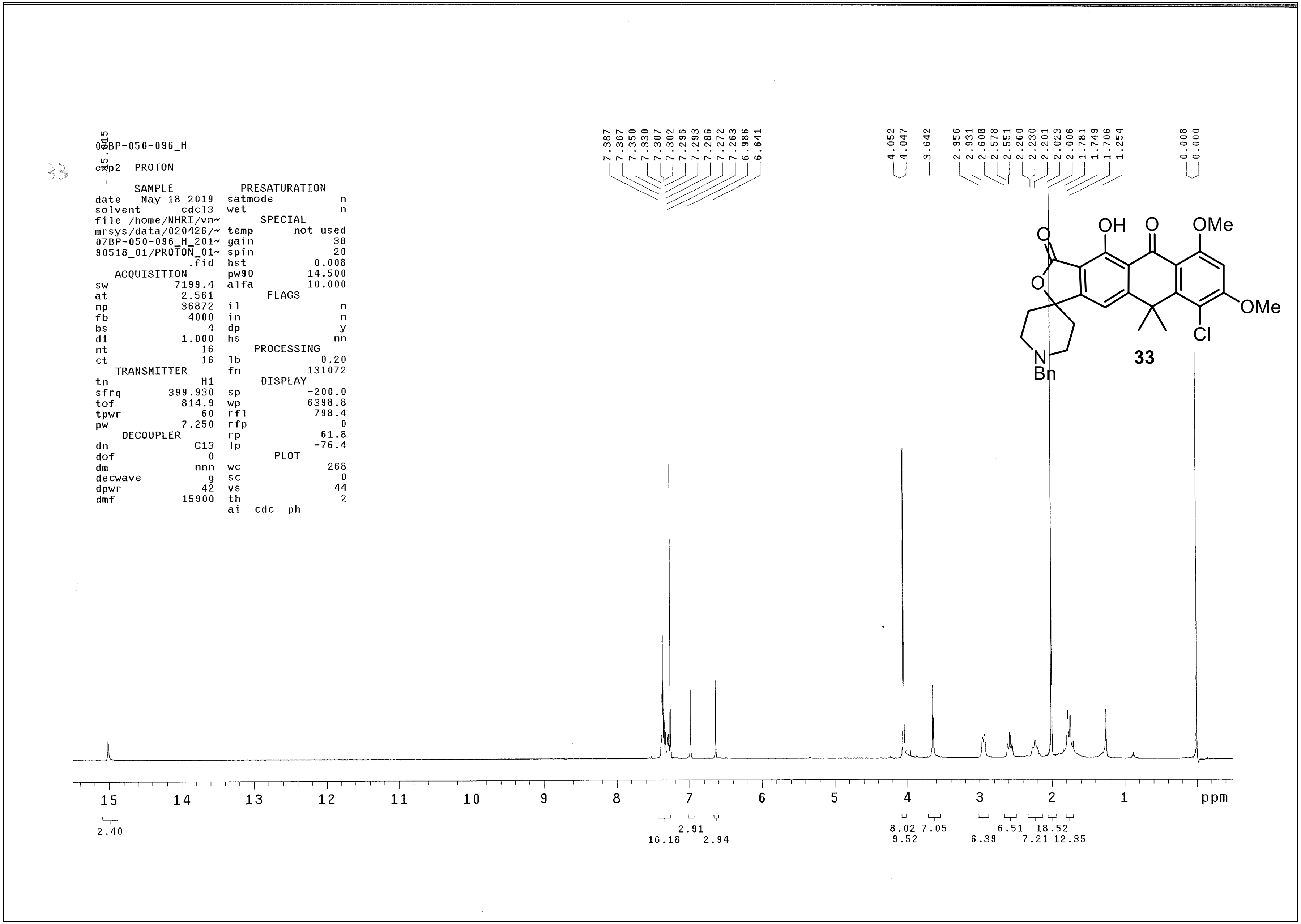

^{1}H NMR spectra for compound **33**

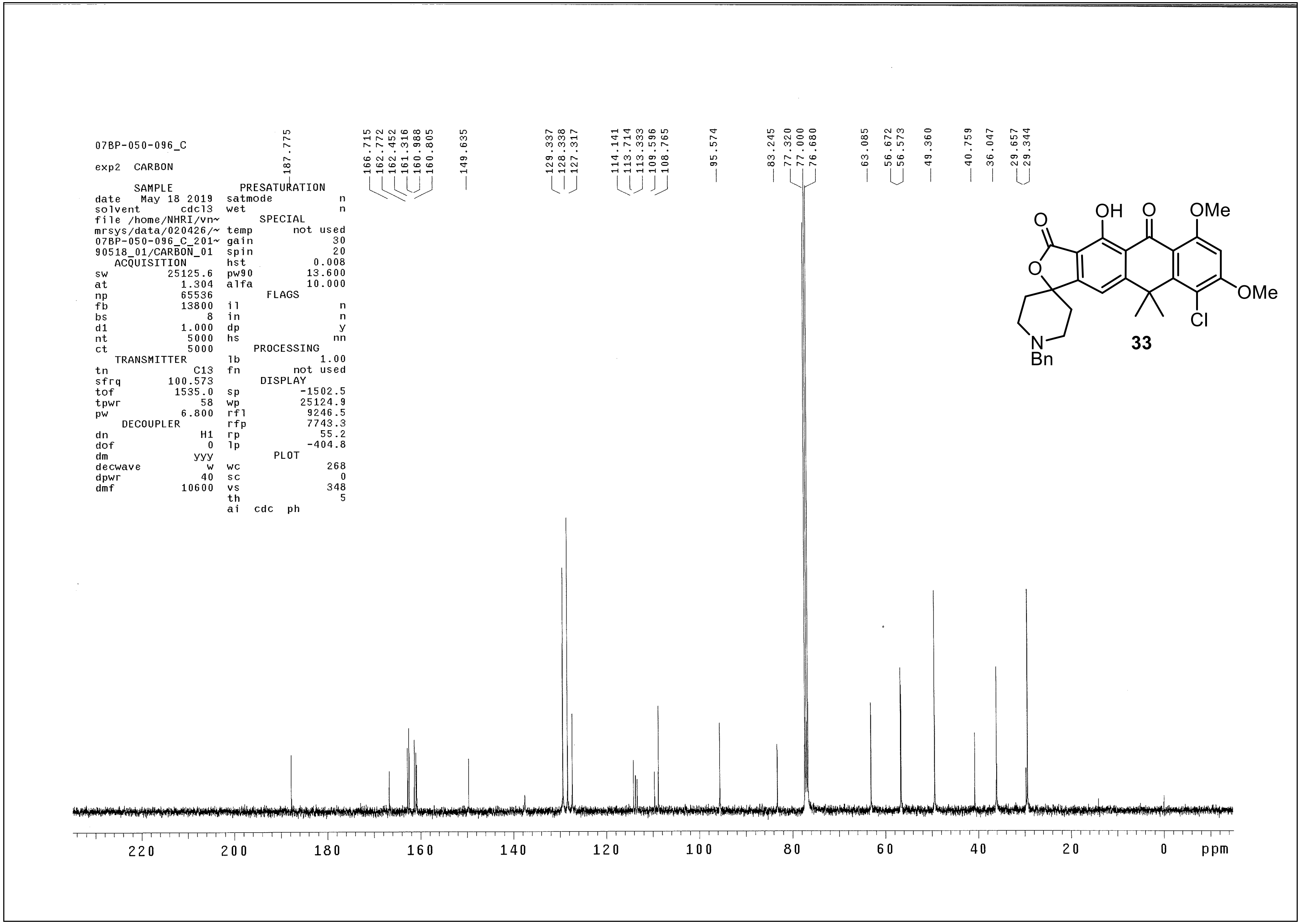

^{13}C NMR spectra for compound **33**

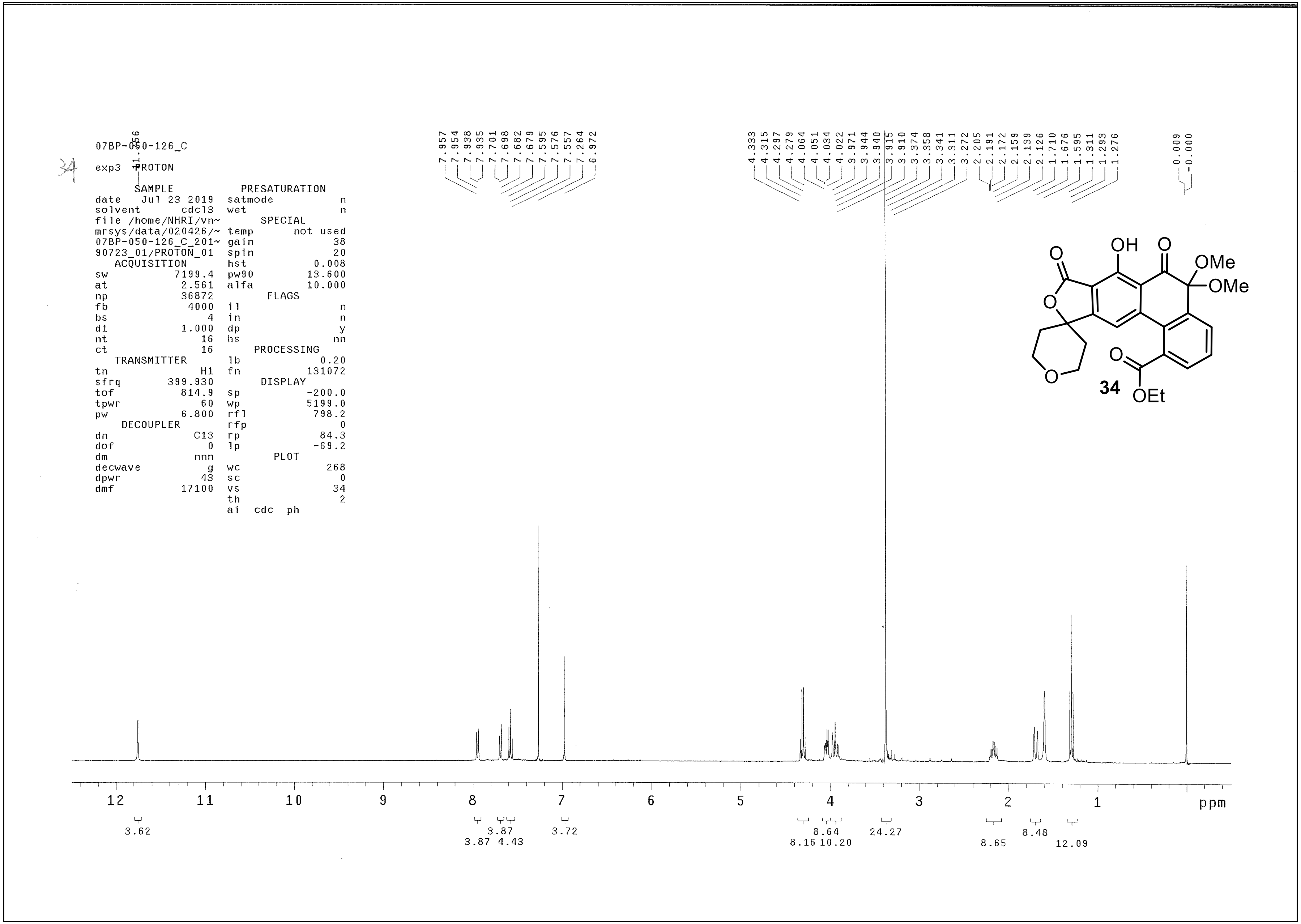

^{1}H NMR spectra for compound **34**

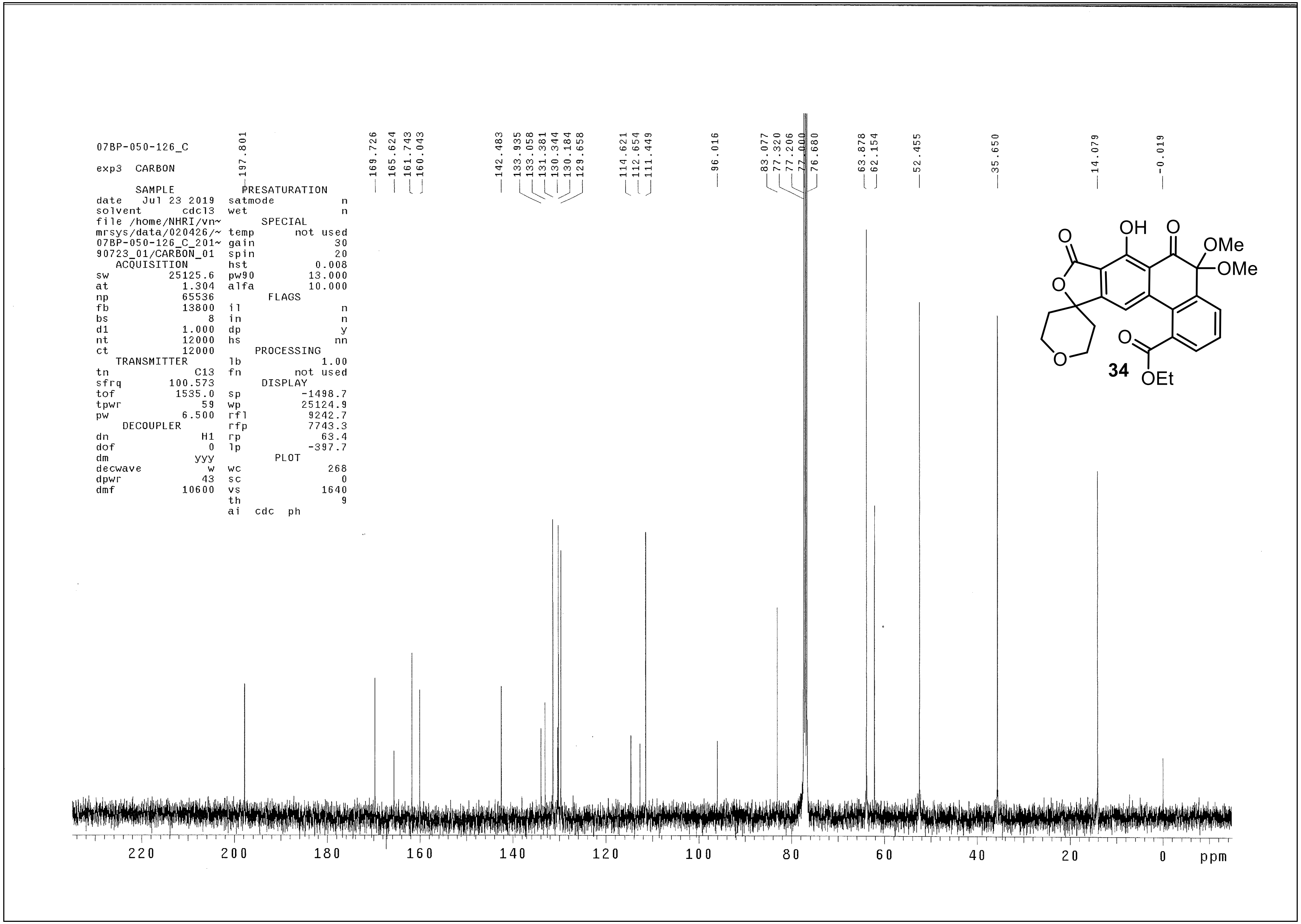

^{13}C NMR spectra for compound **34**

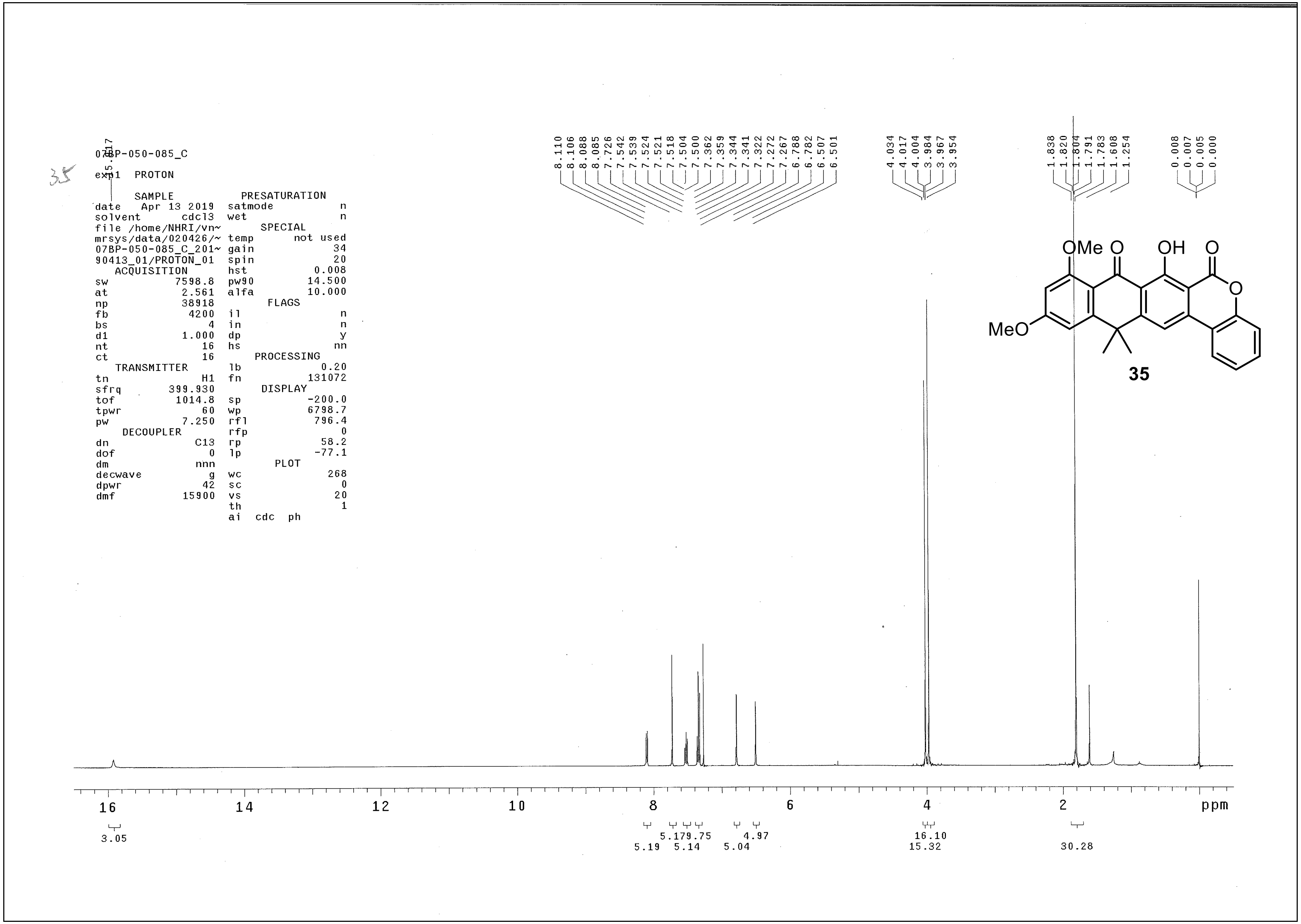

^{1}H NMR spectra for compound **35**

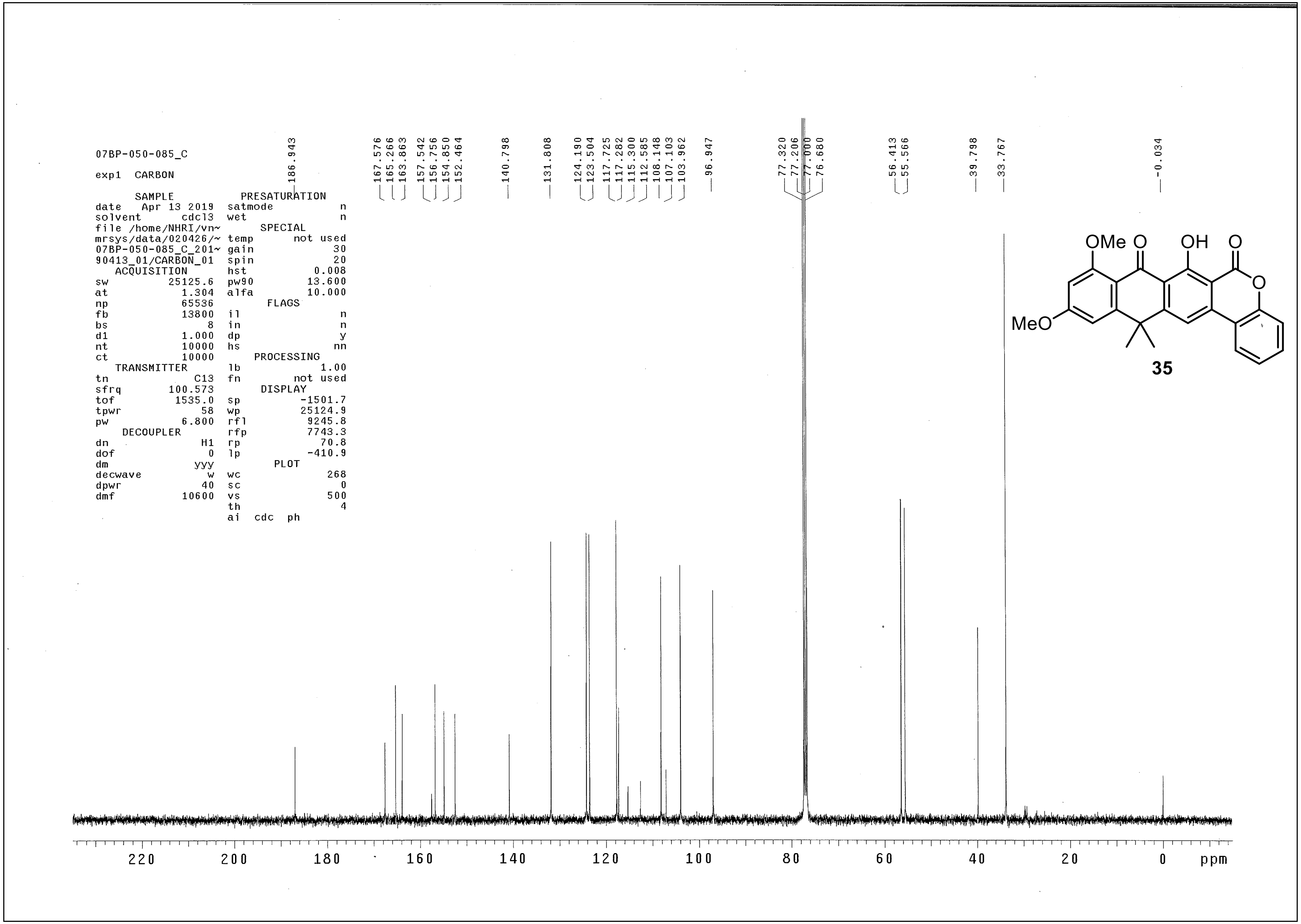

^{13}C NMR spectra for compound **35**

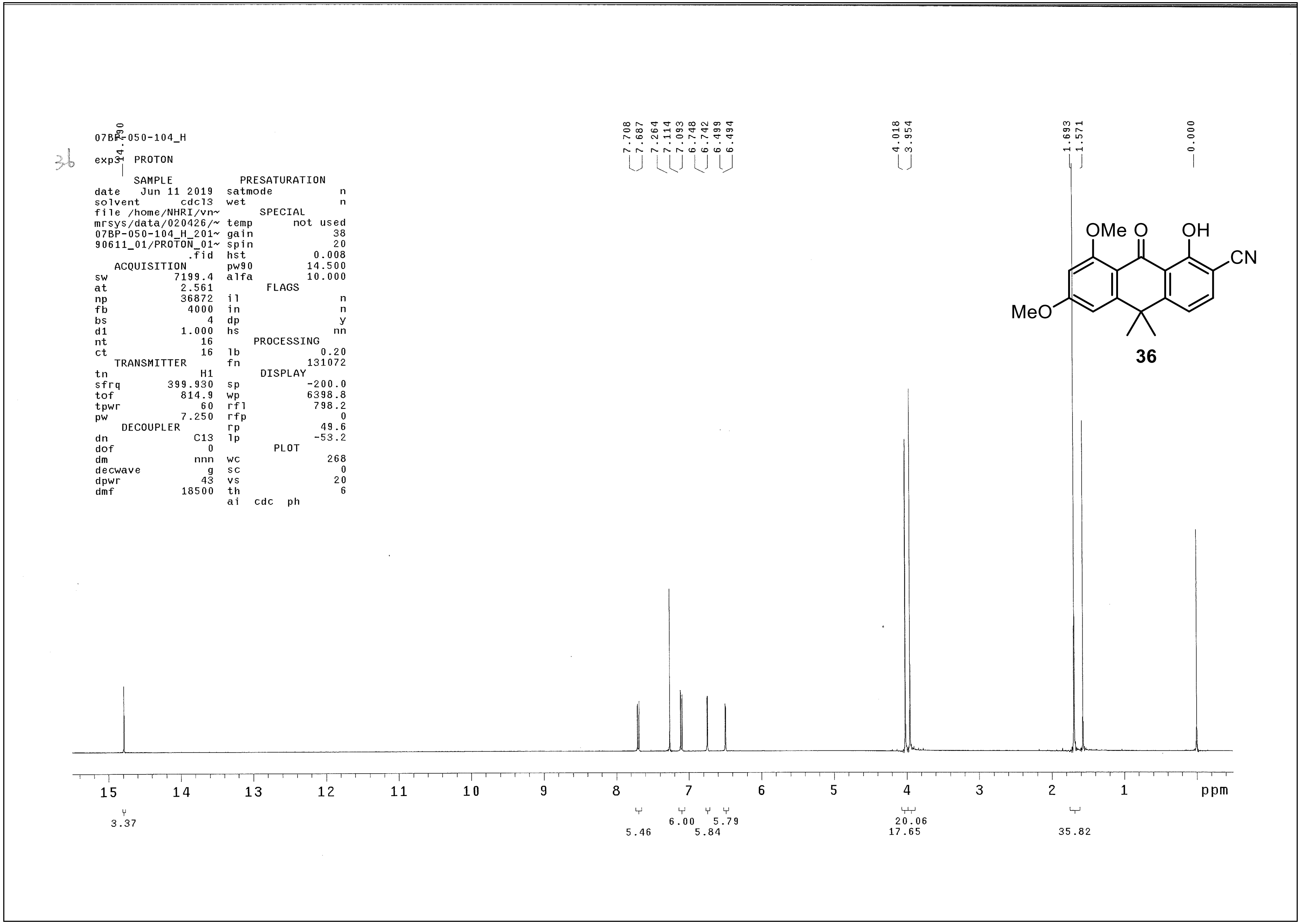

^{1}H NMR spectra for compound **36**

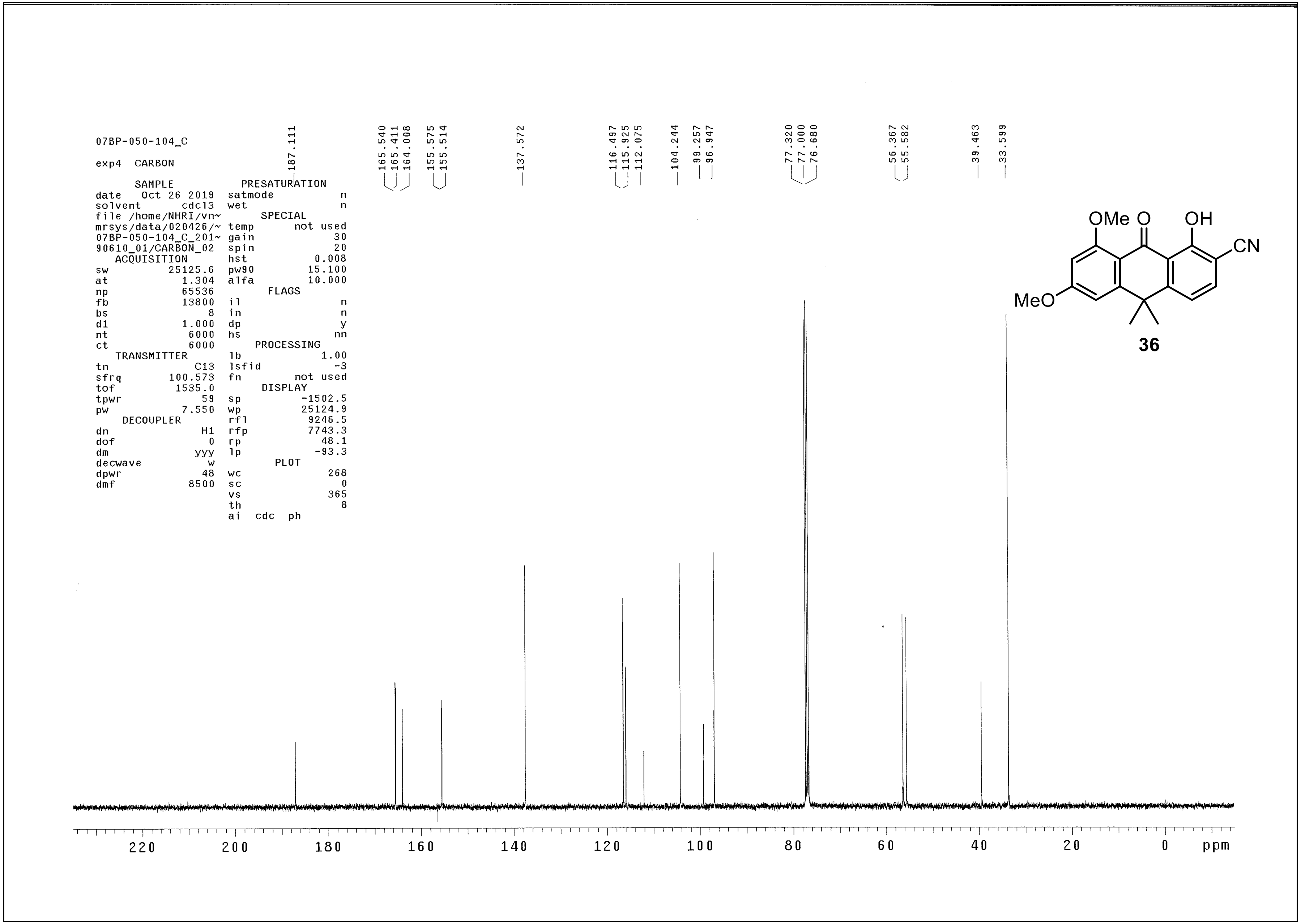

^{13}C NMR spectra for compound **36**

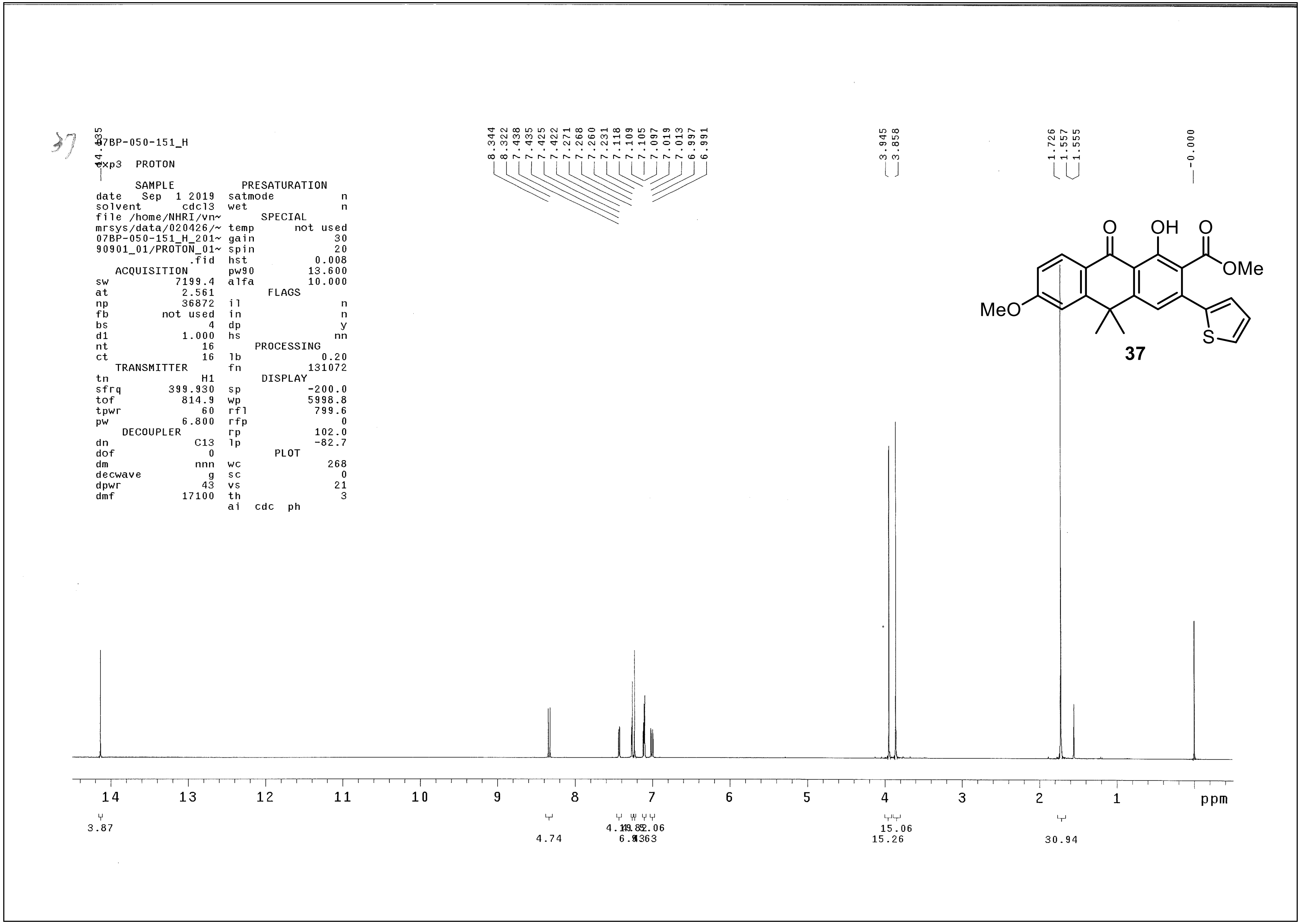

^{1}H NMR spectra for compound **37**

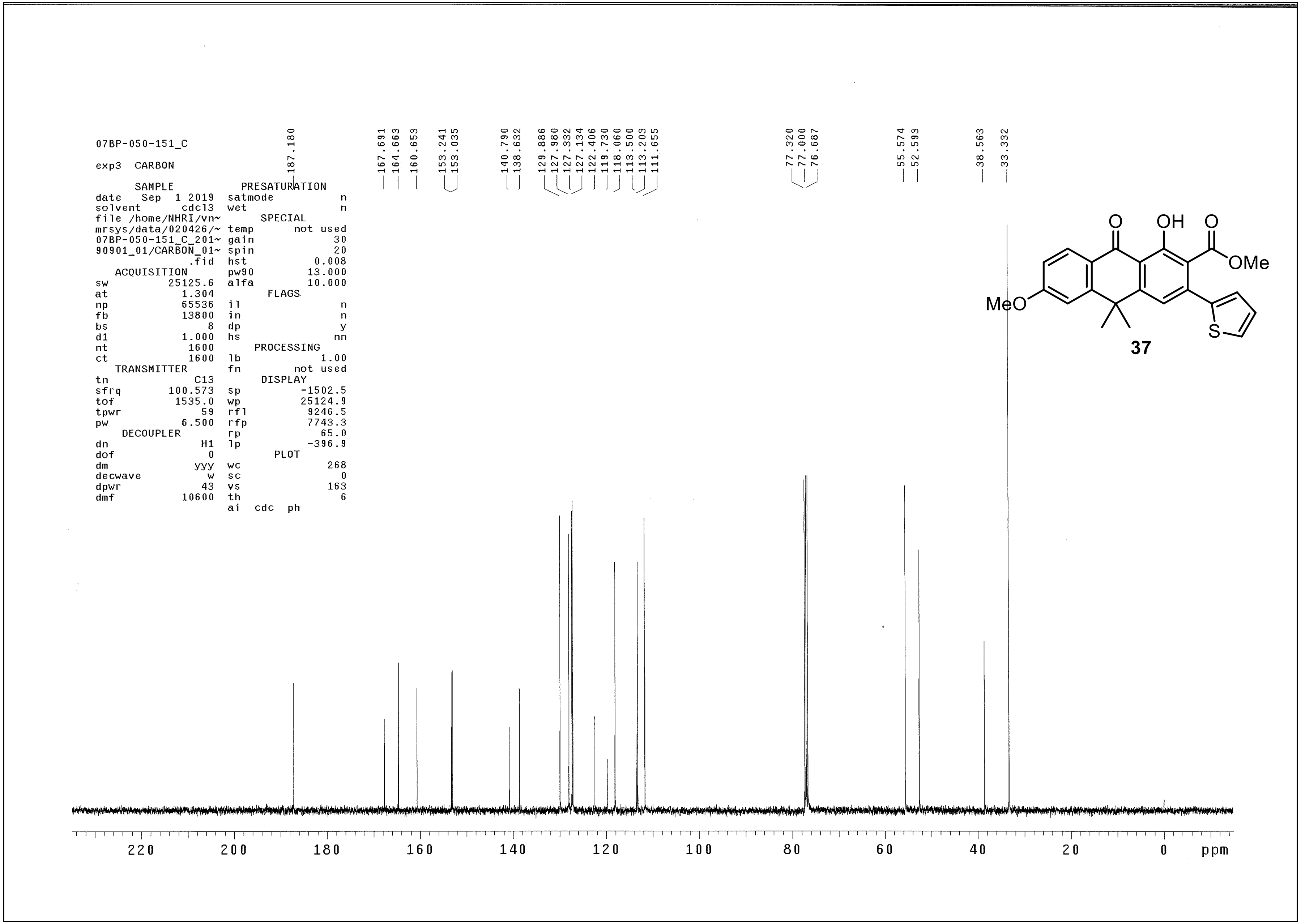

^{13}C NMR spectra for compound **37**

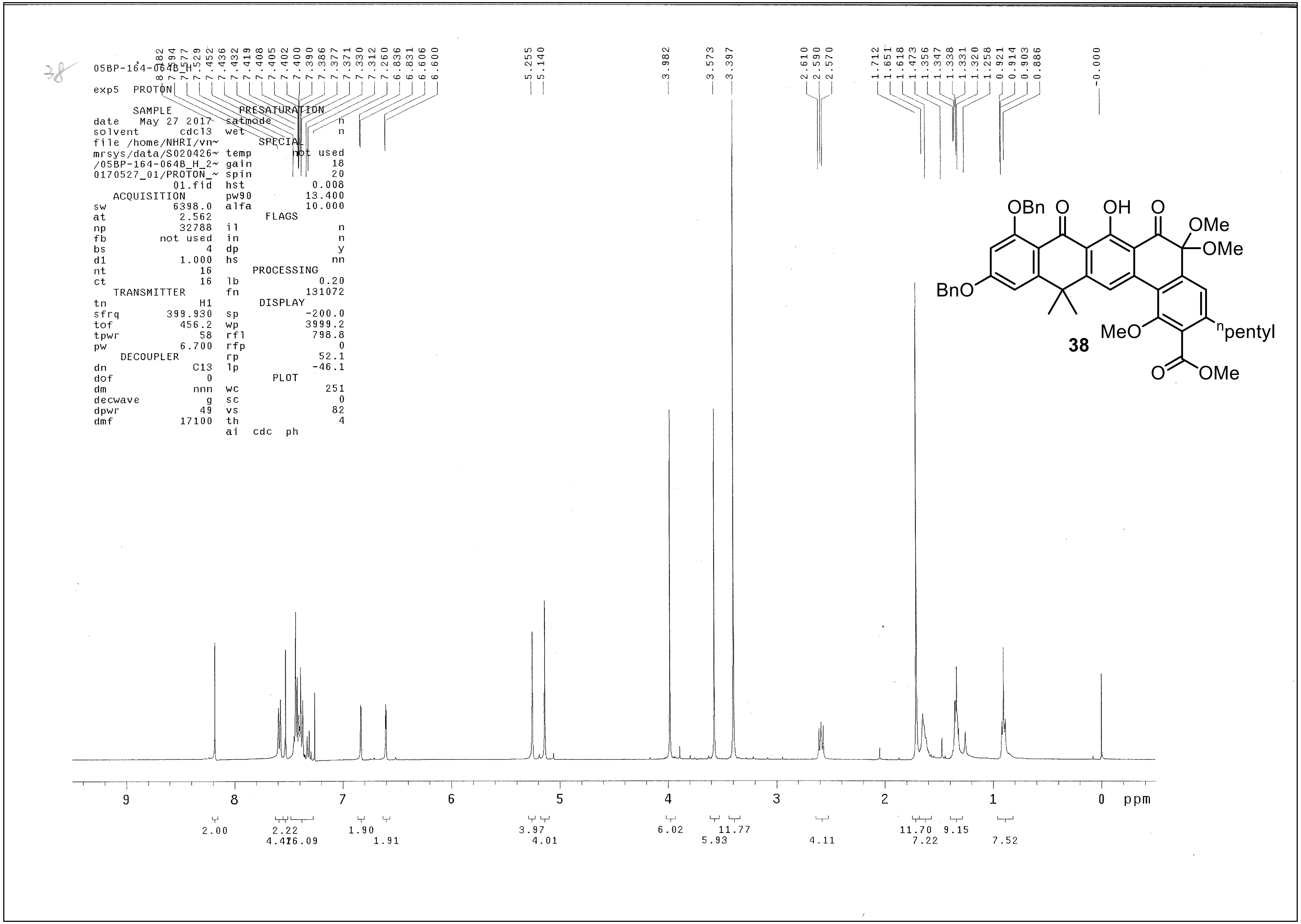

^{1}H NMR spectra for compound **38**

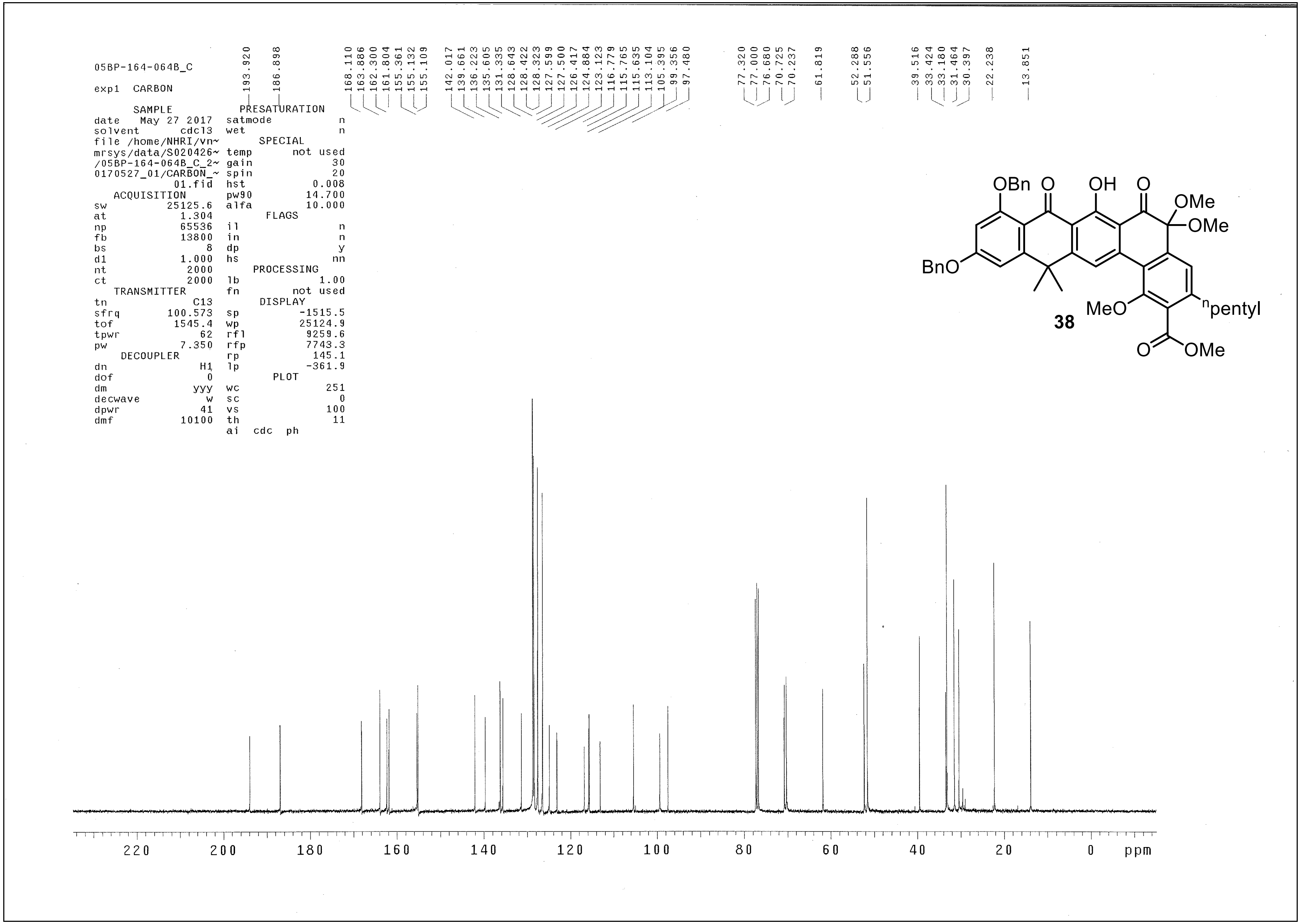

^{13}C NMR spectra for compound **38**

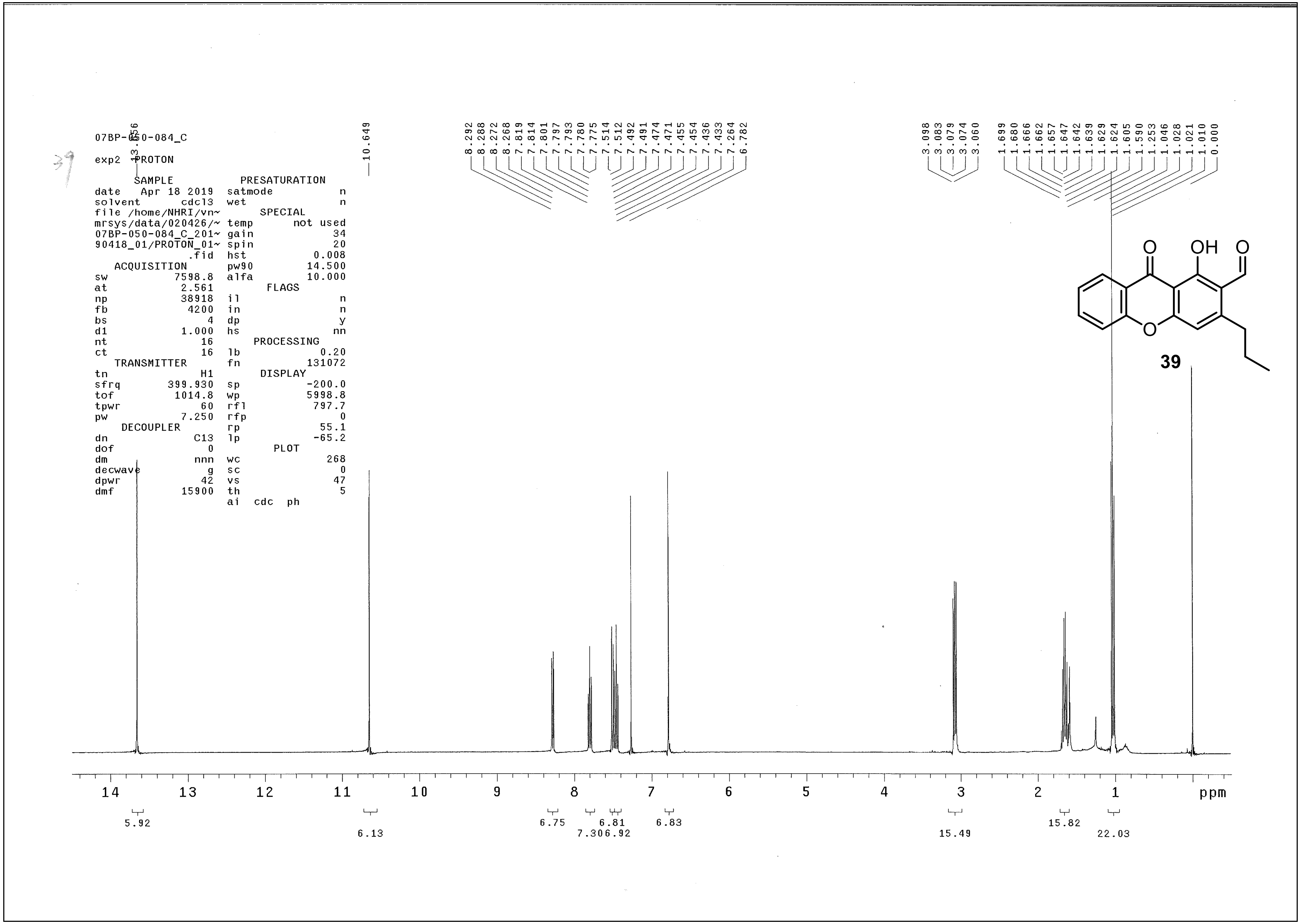

^{1}H NMR spectra for compound **39**

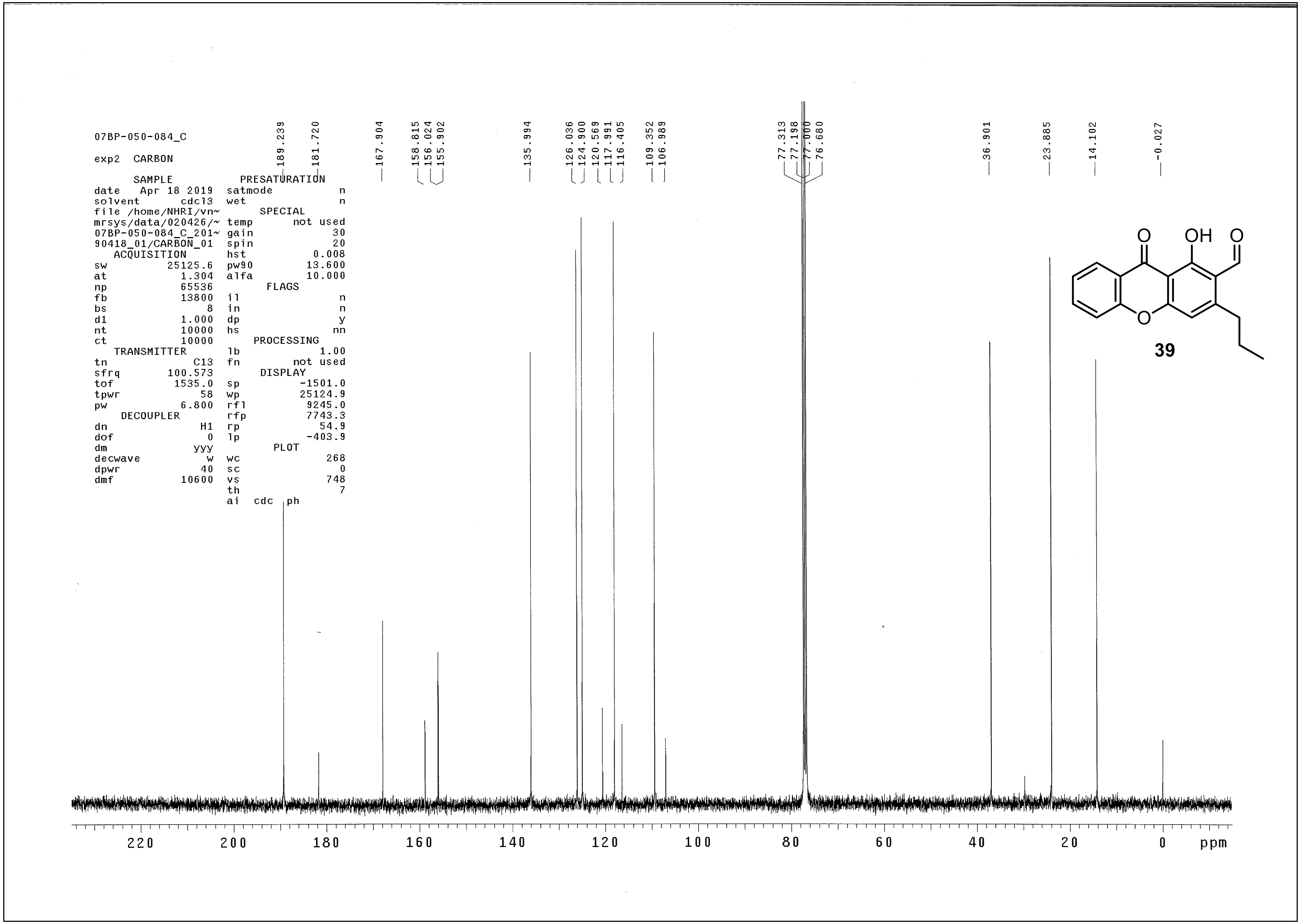

^{13}C NMR spectra for compound **39**

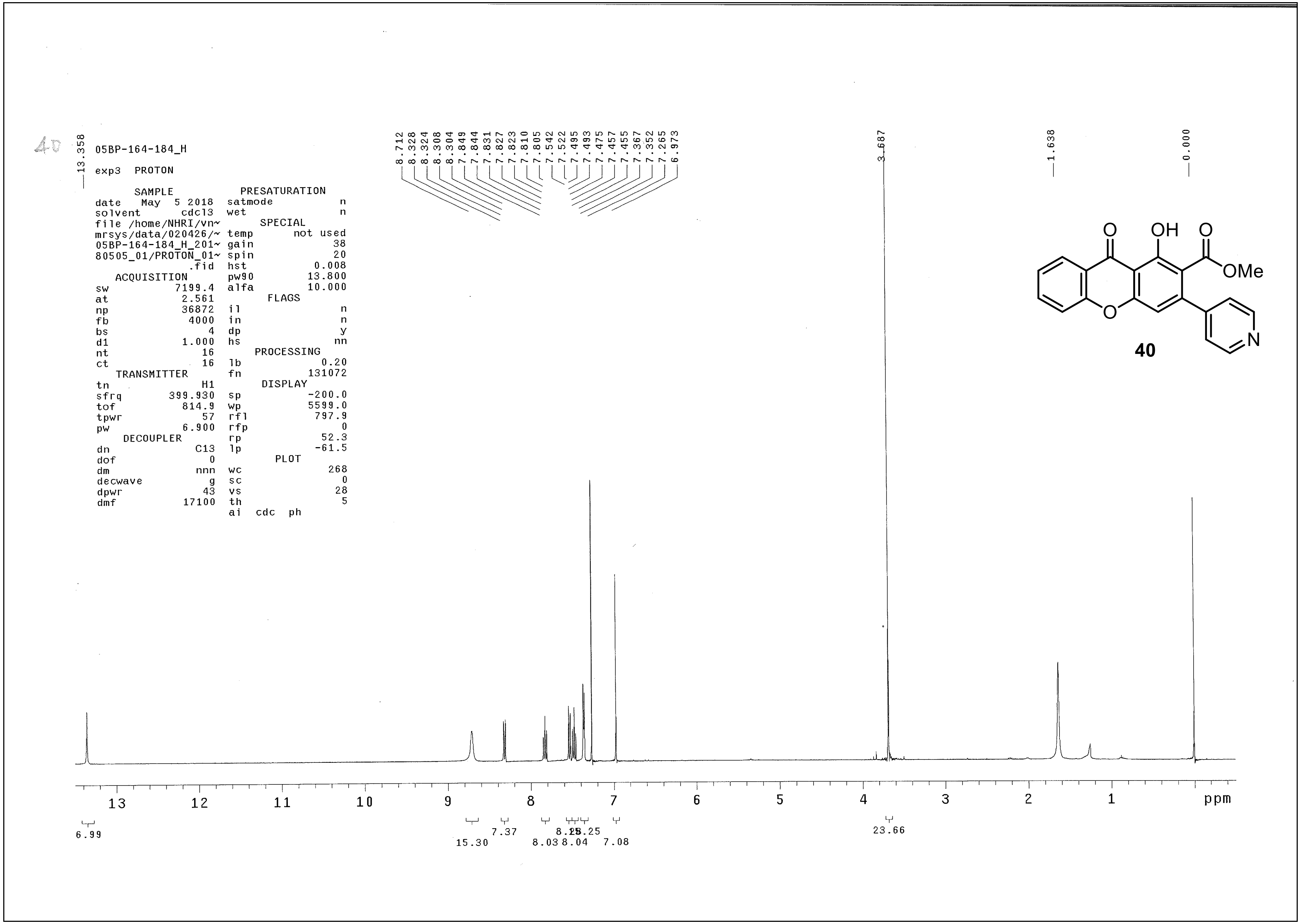

^{1}H NMR spectra for compound **40**

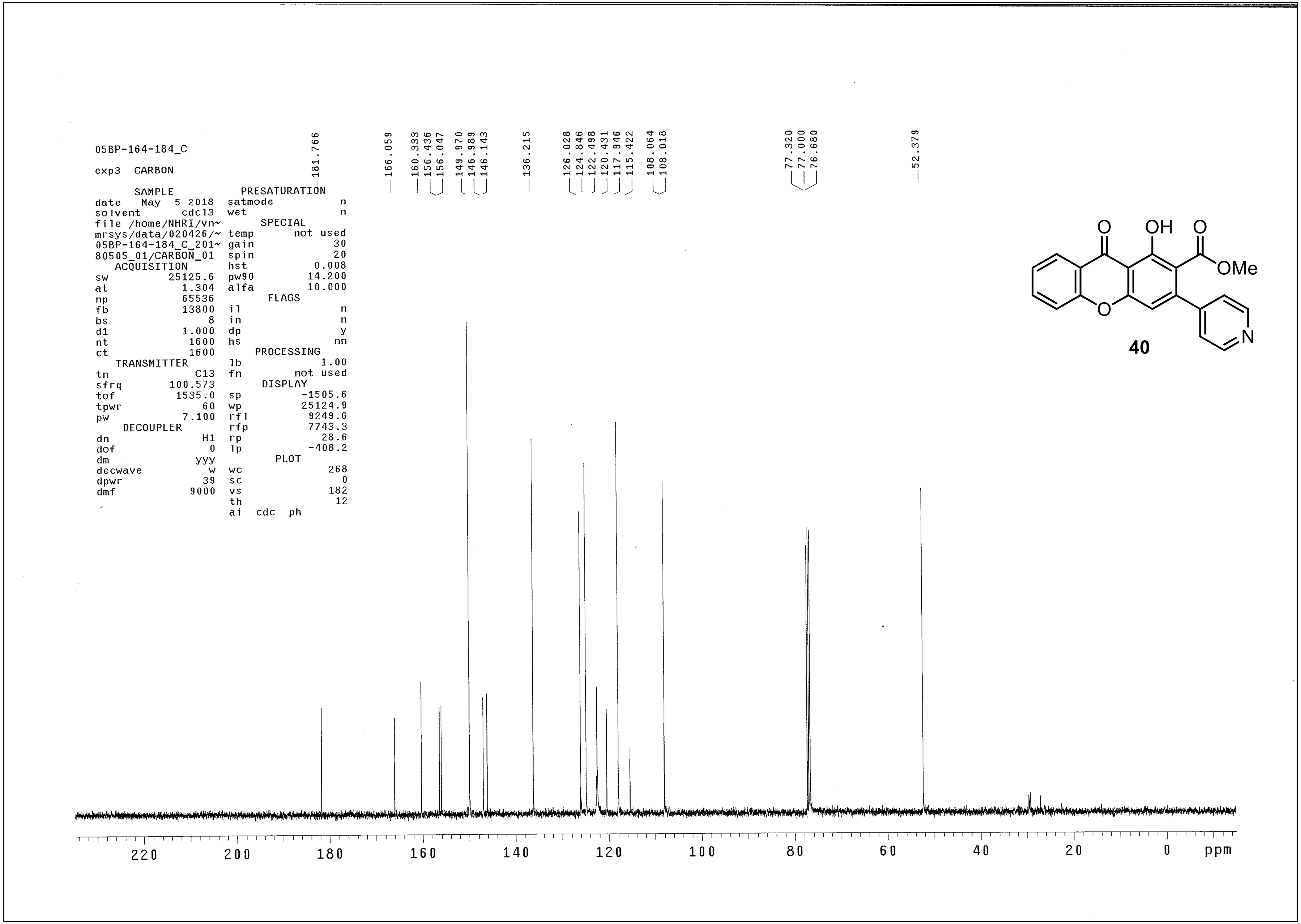

^{13}C NMR spectra for compound **40**

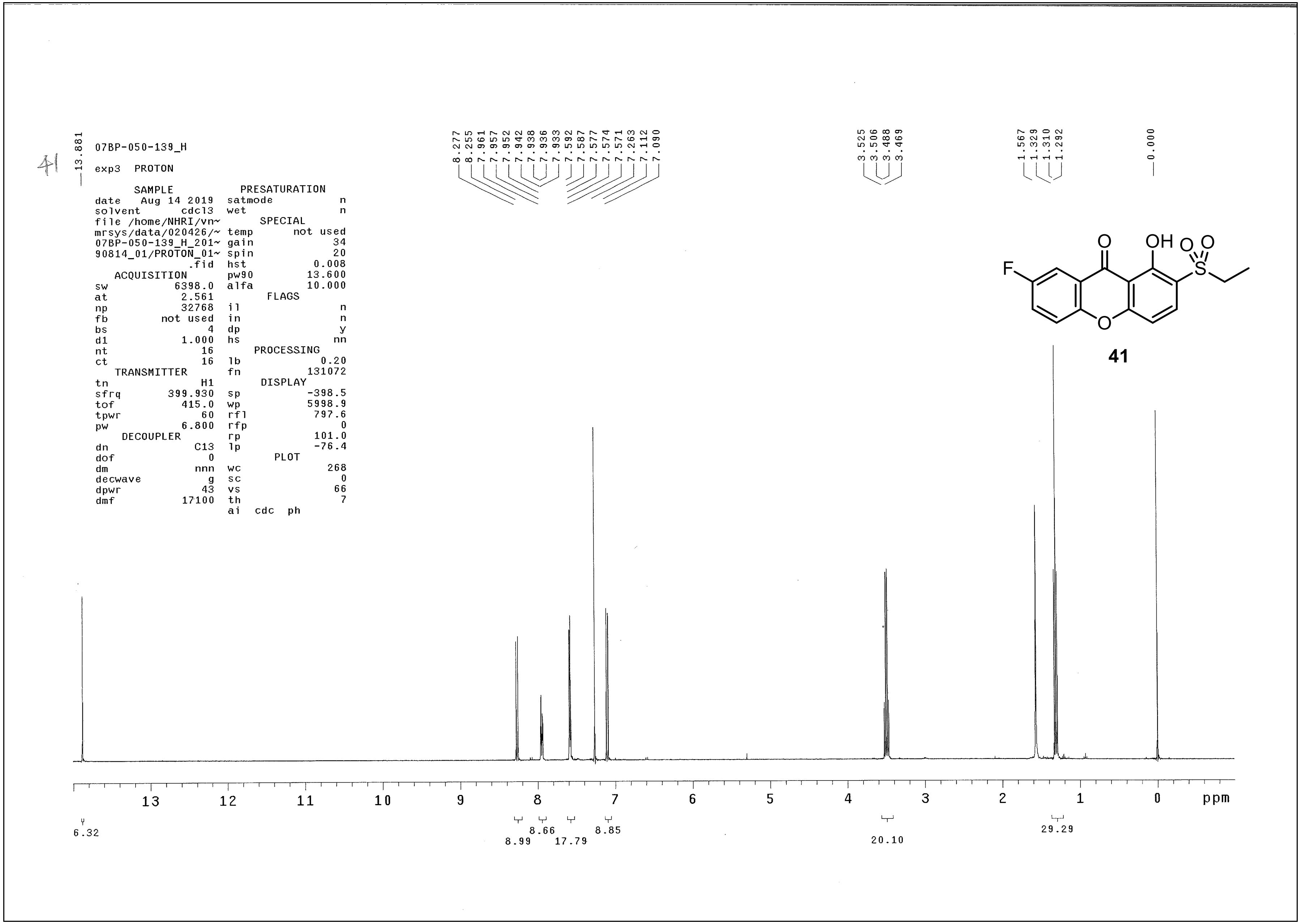

^{1}H NMR spectra for compound **41**

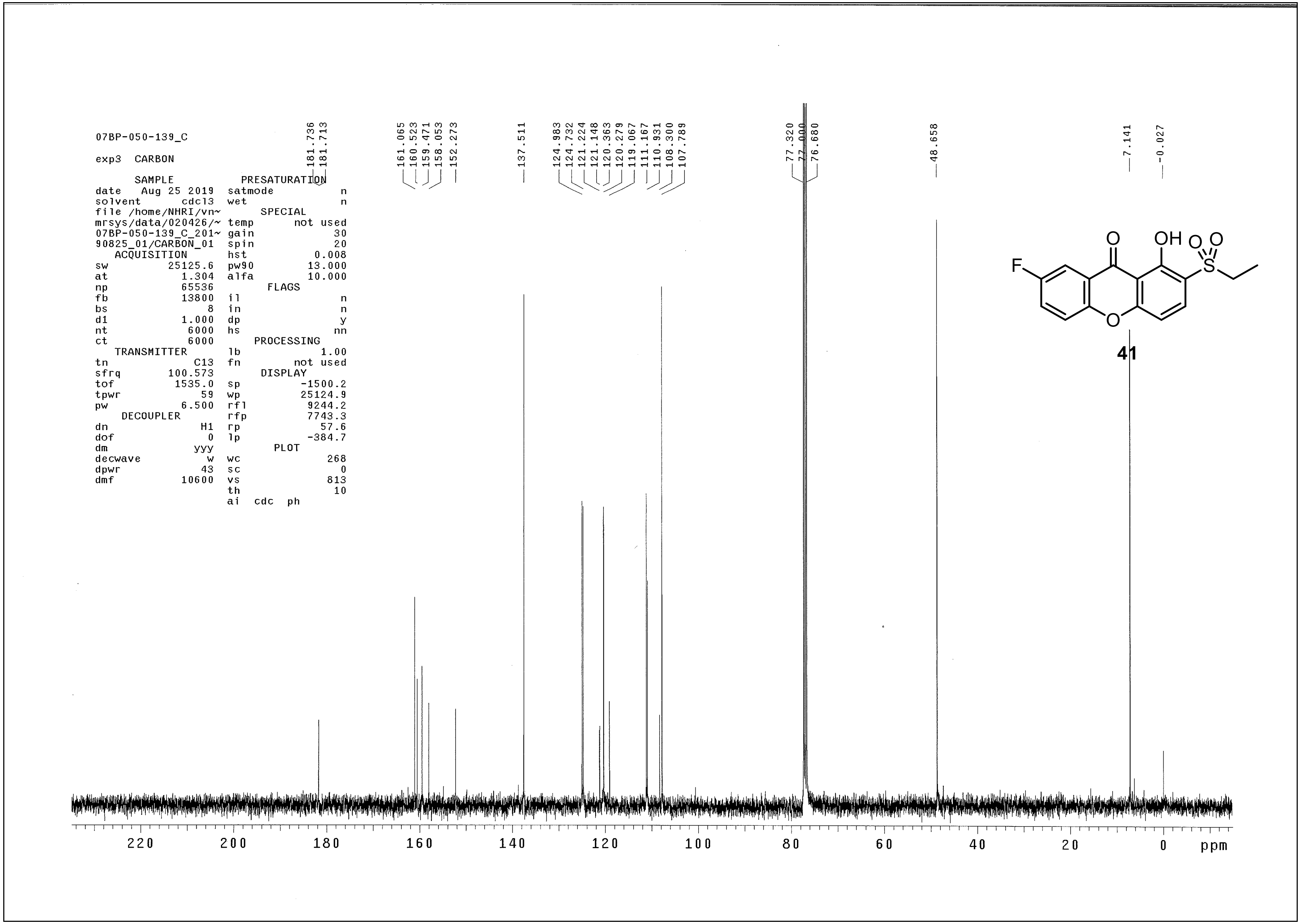

^{13}C NMR spectra for compound **41**

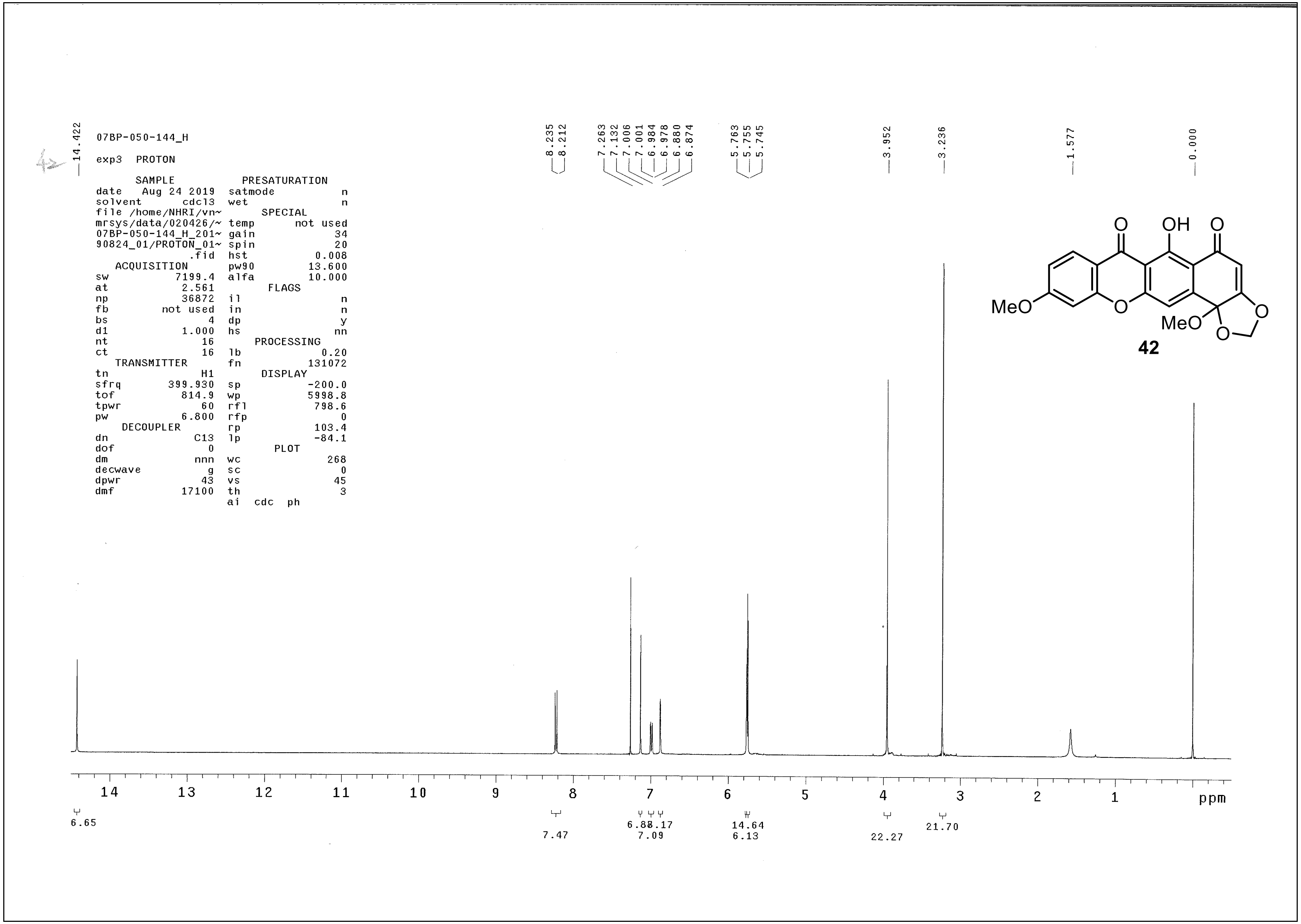

^{1}H NMR spectra for compound **42**

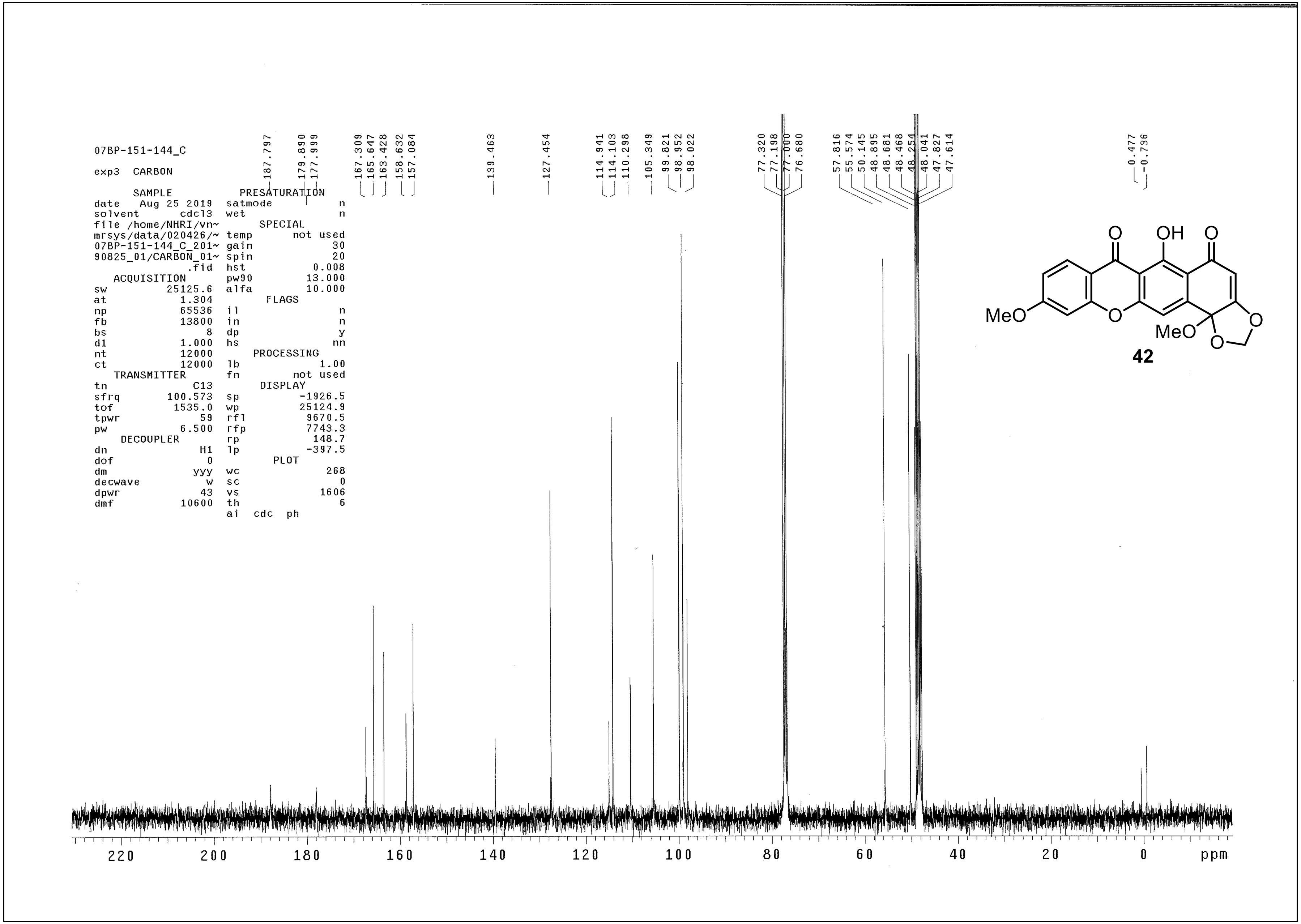

^{13}C NMR spectra for compound **42**

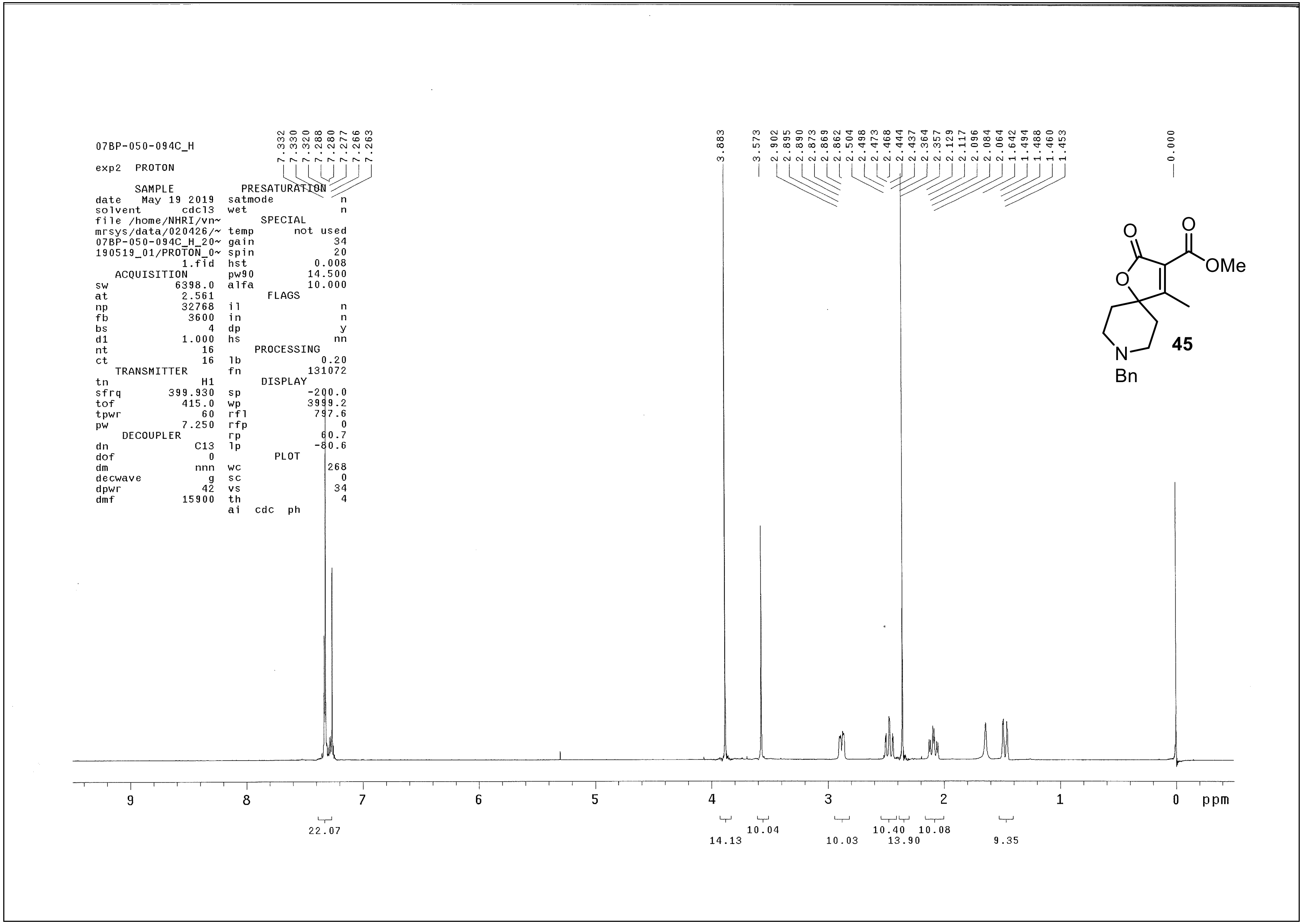

^{1}H NMR spectra for compound **45**

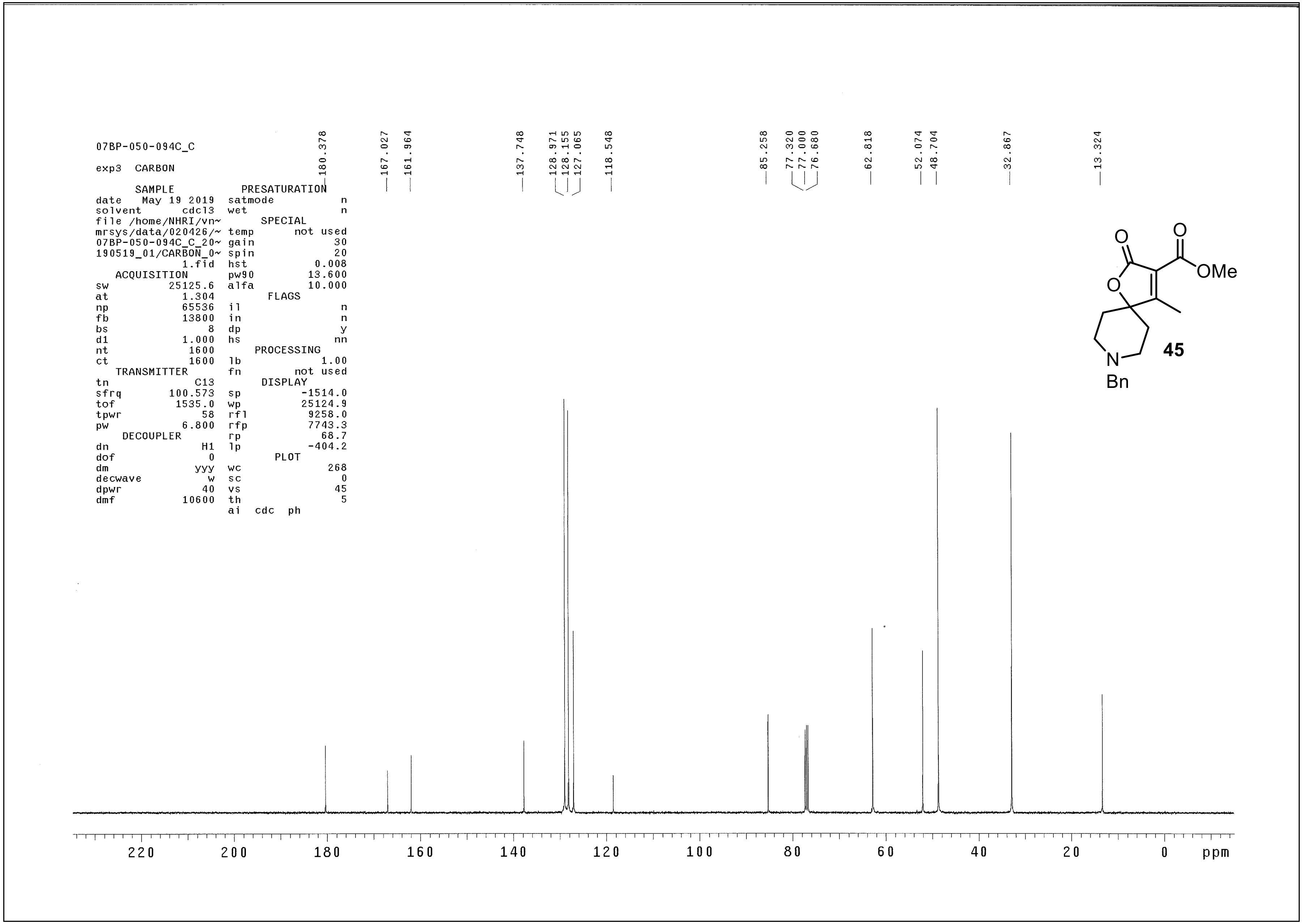

^{13}C NMR spectra for compound **45**

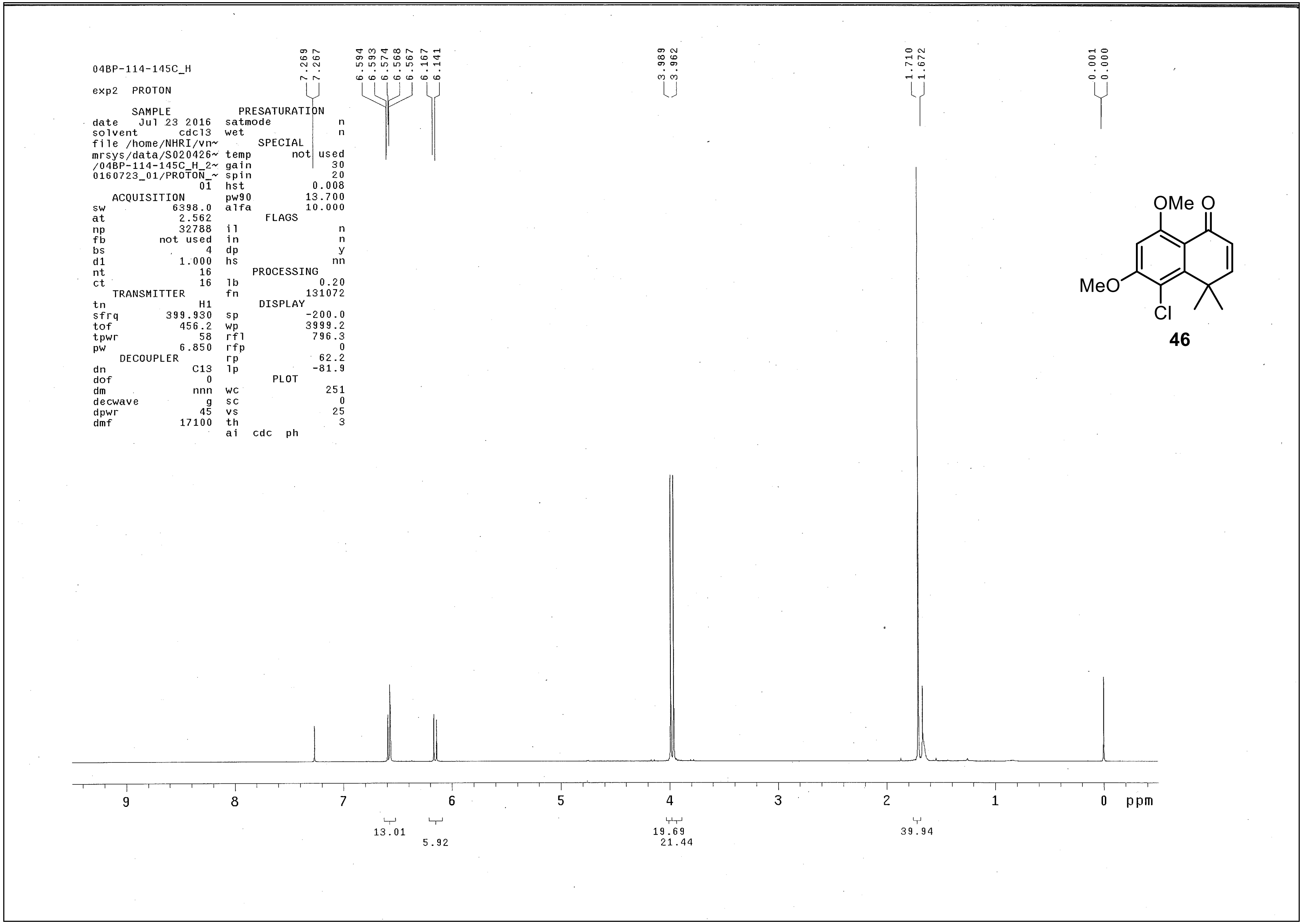

^{1}H NMR spectra for compound **46**

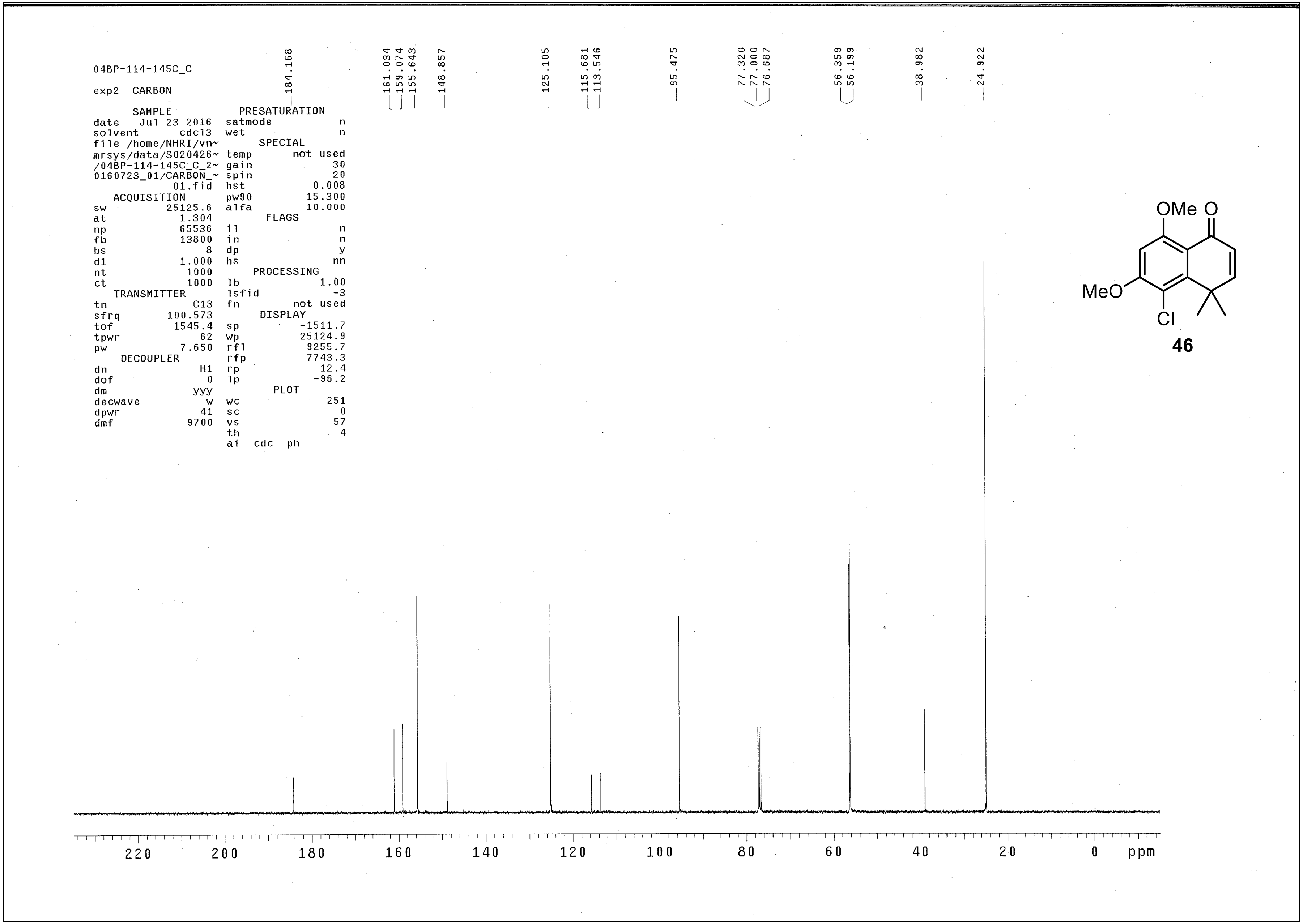

^{13}C NMR spectra for compound **46**

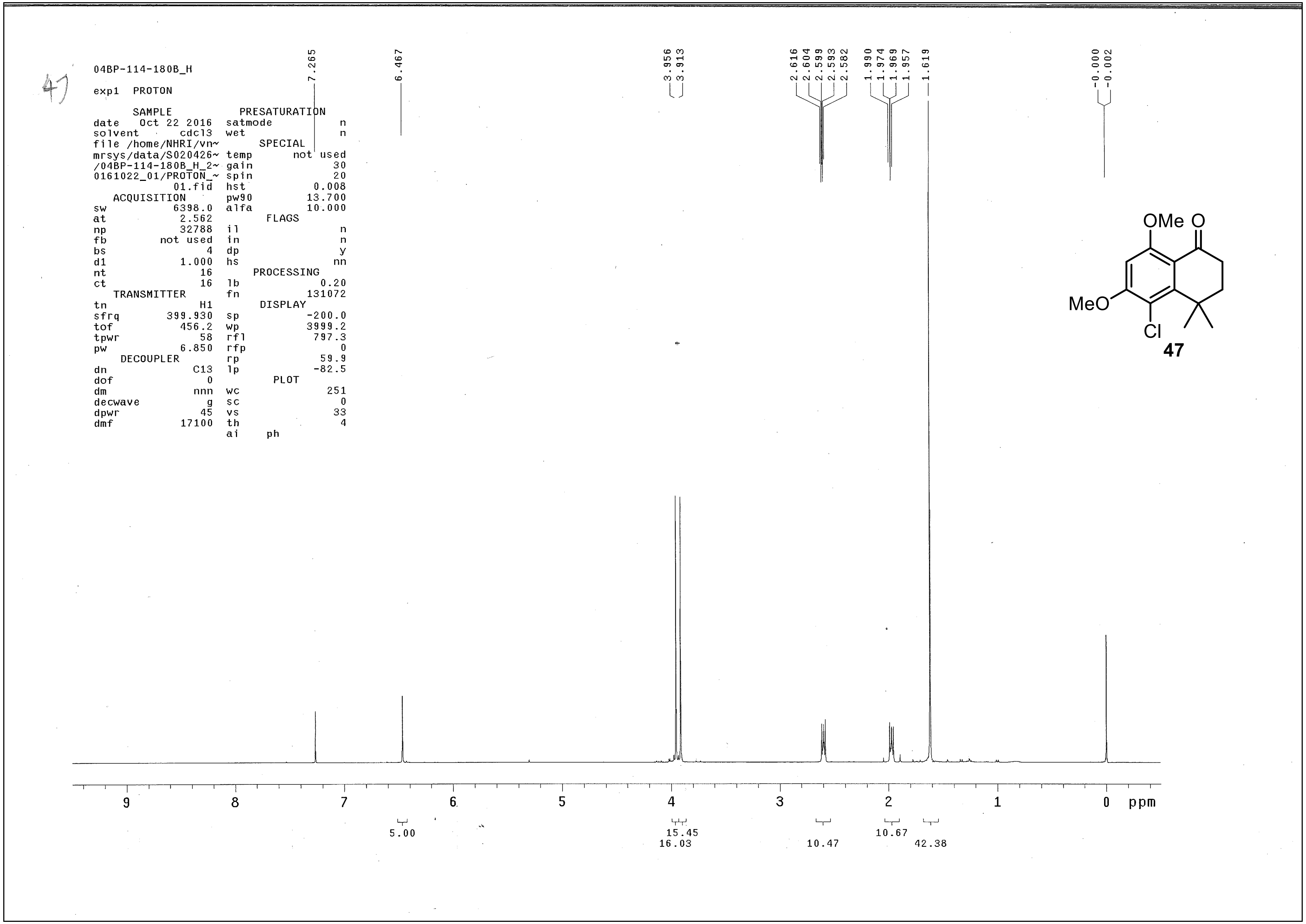

^{1}H NMR spectra for compound **47**

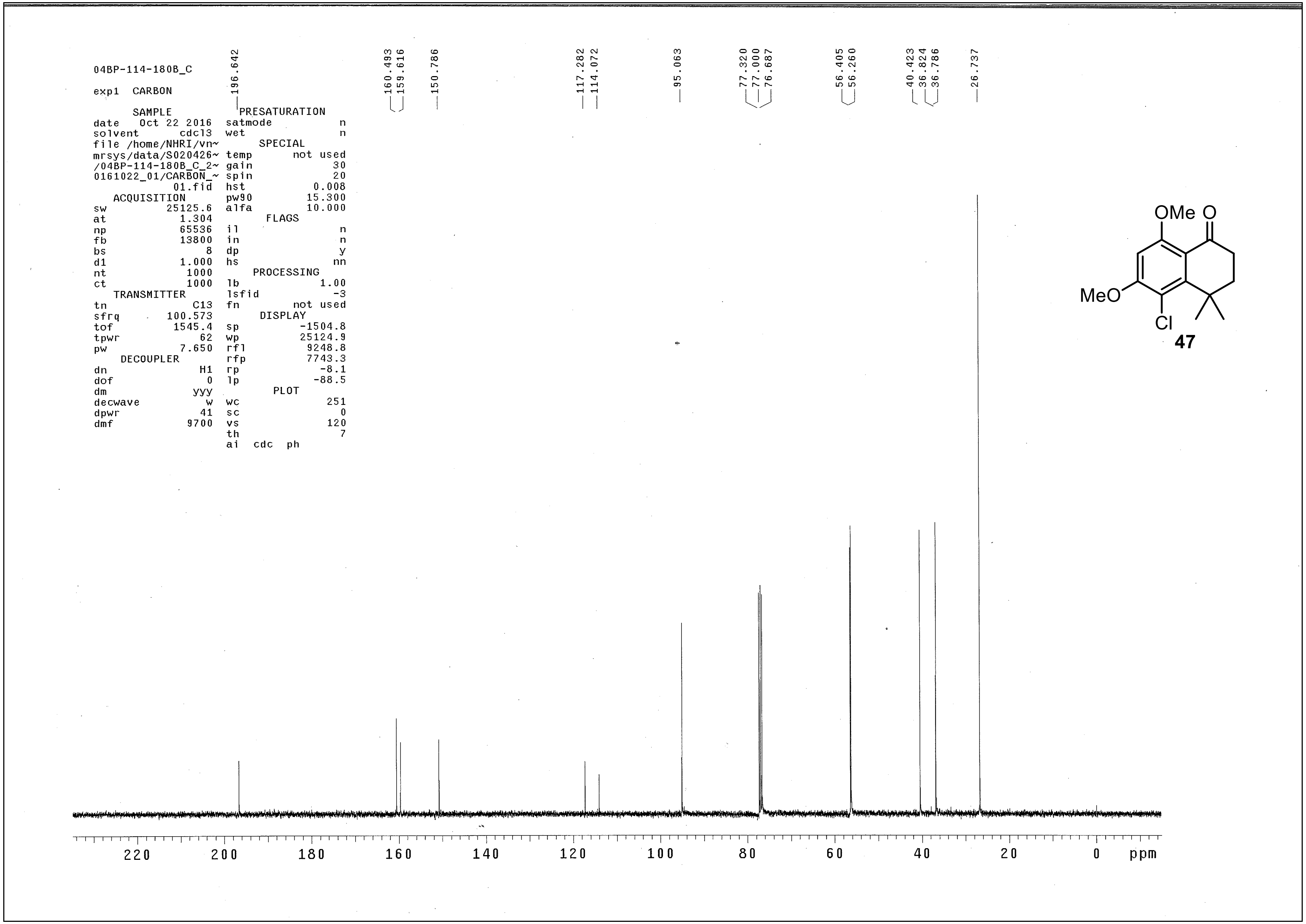

^{13}C NMR spectra for compound **47**

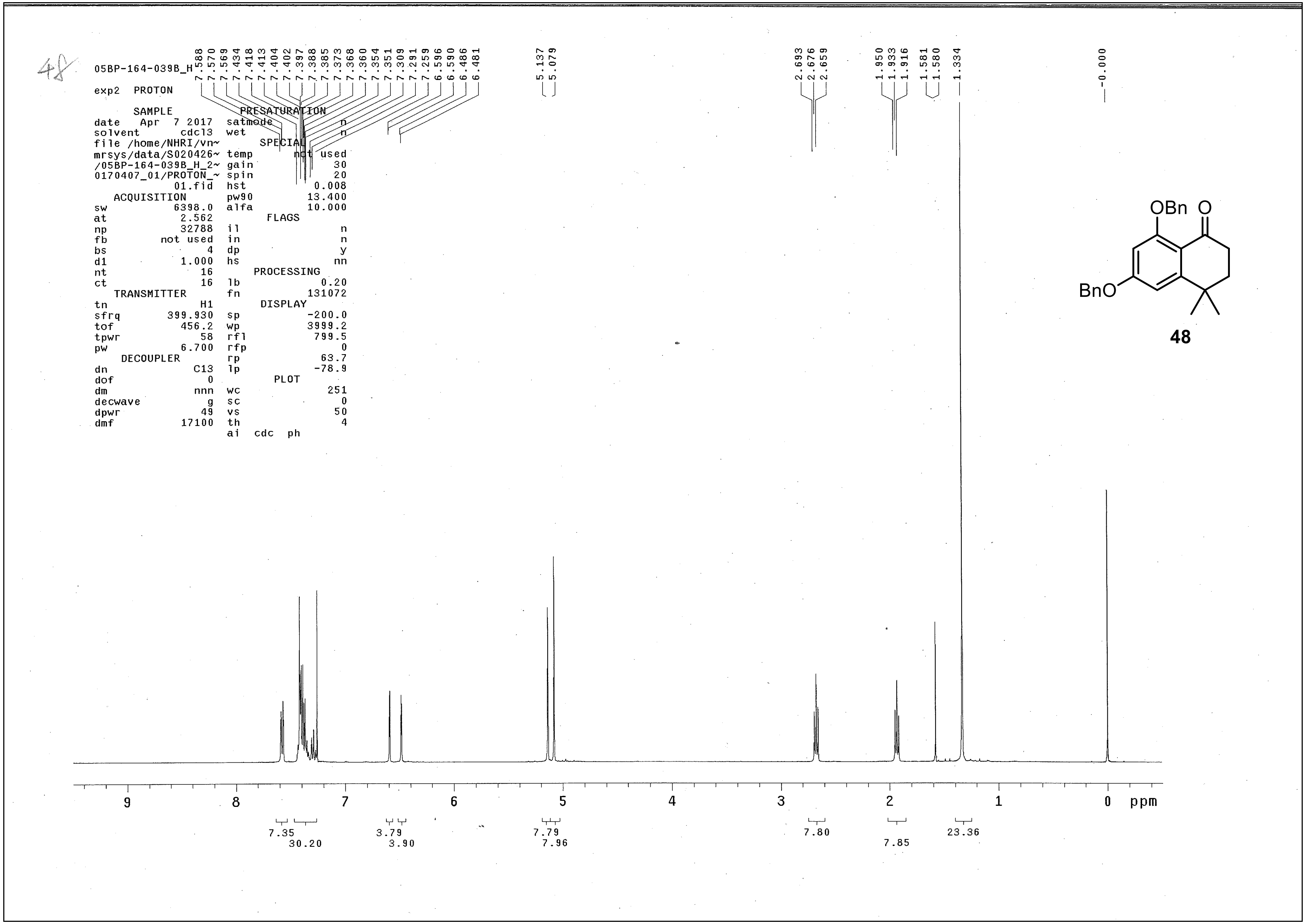

^{1}H NMR spectra for compound **48**

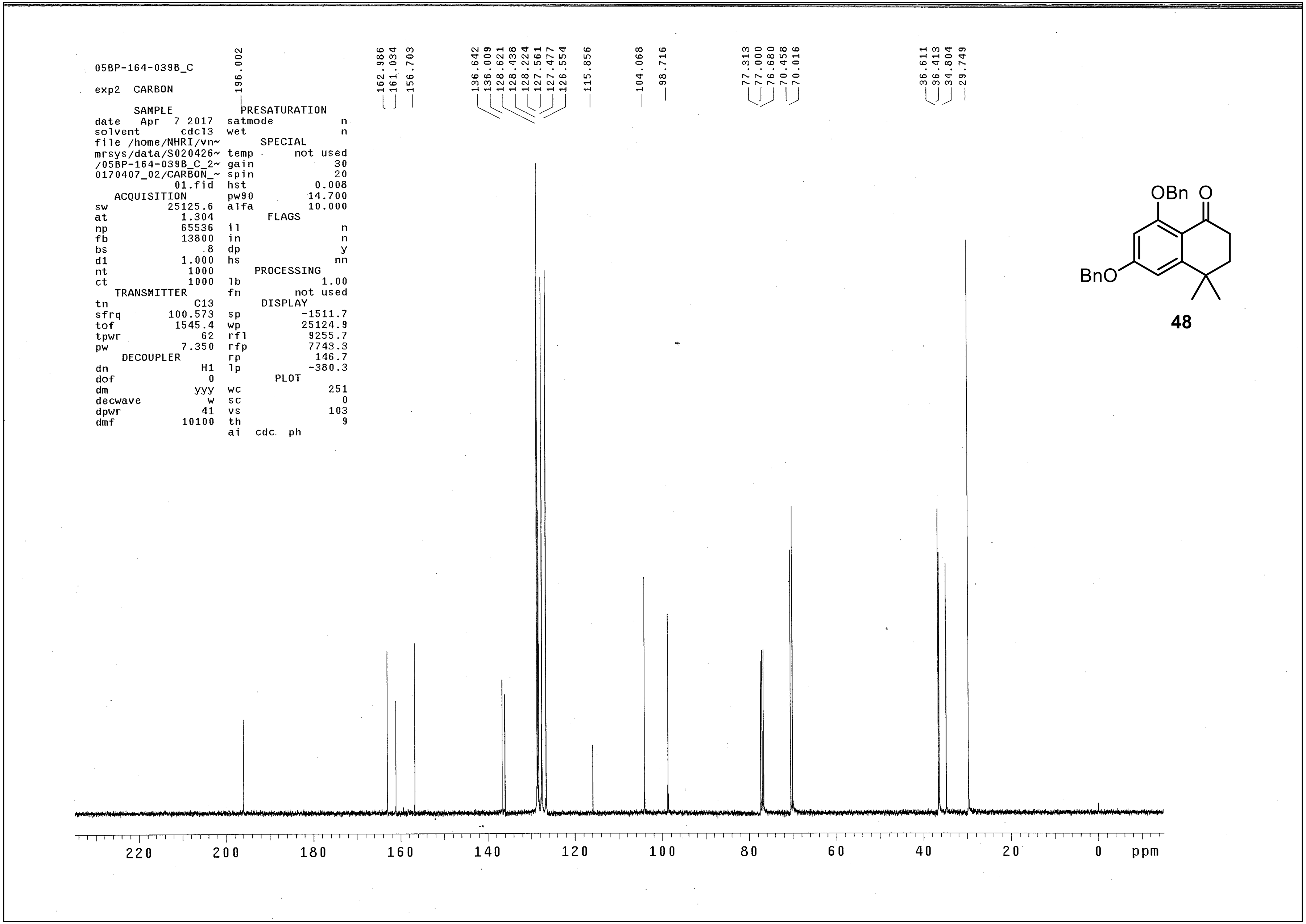

^{13}C NMR spectra for compound **48**

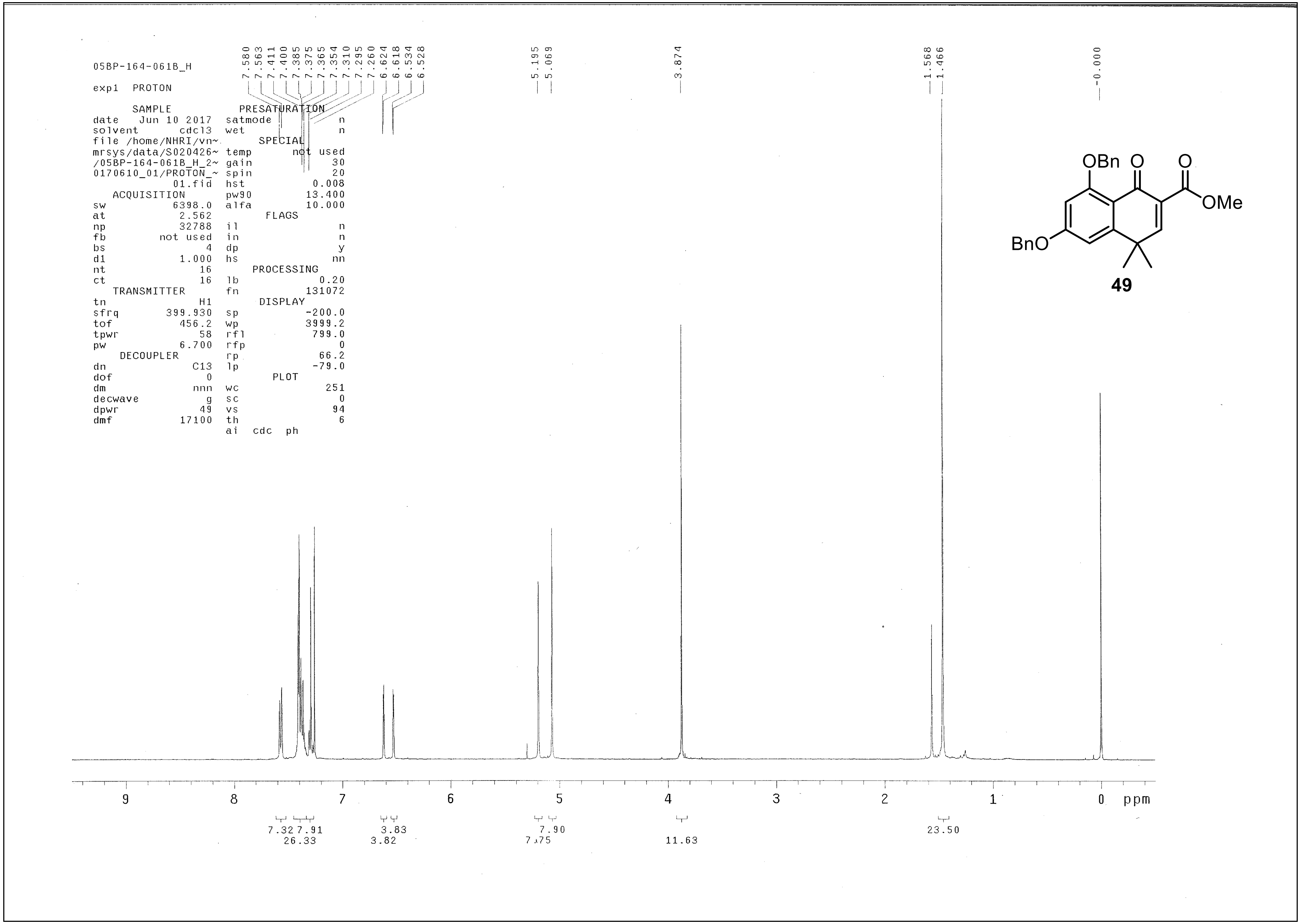

^{1}H NMR spectra for compound **49**

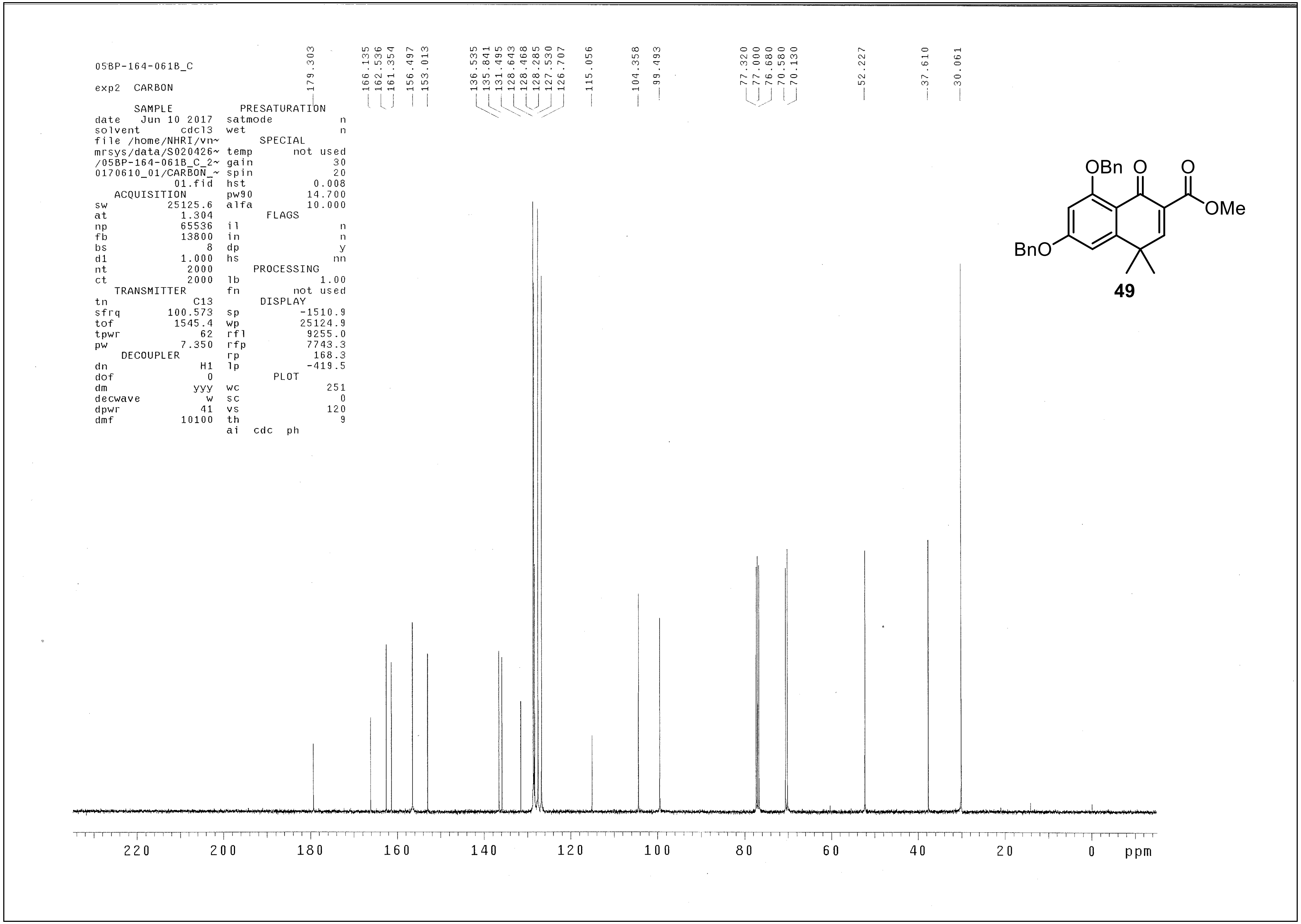

^{13}C NMR spectra for compound **49**

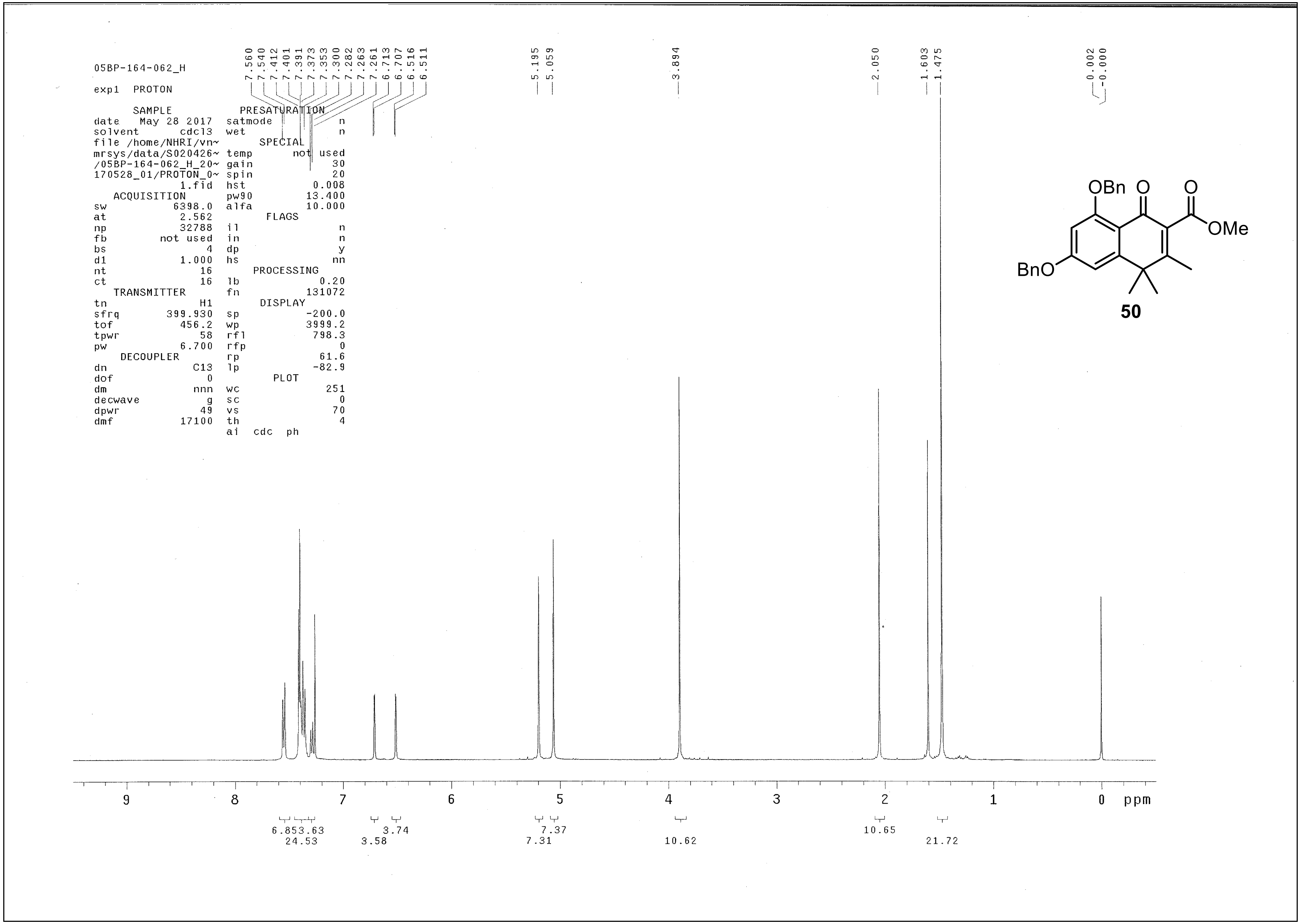

^{1}H NMR spectra for compound **50**

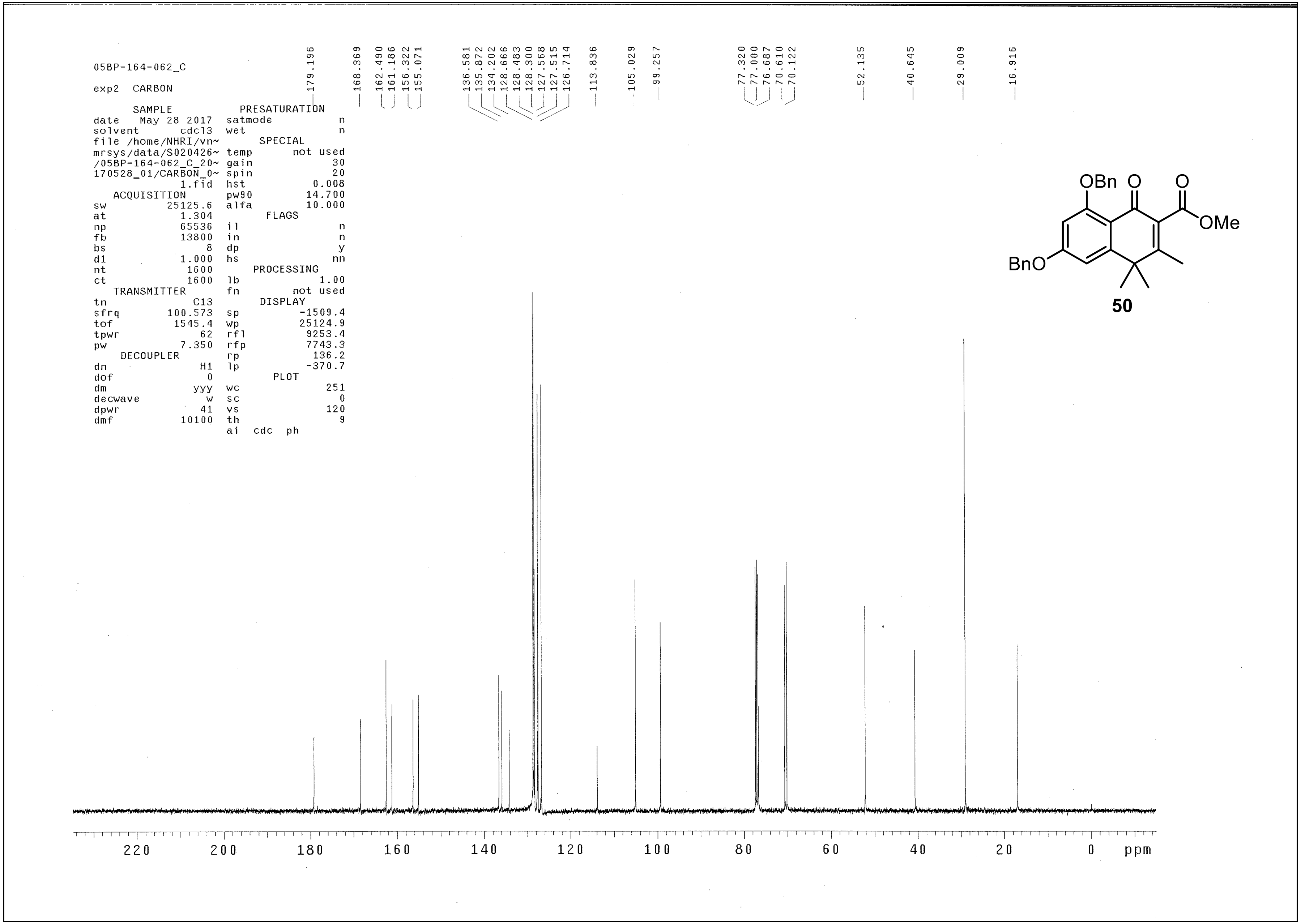

^{13}C NMR spectra for compound **50**

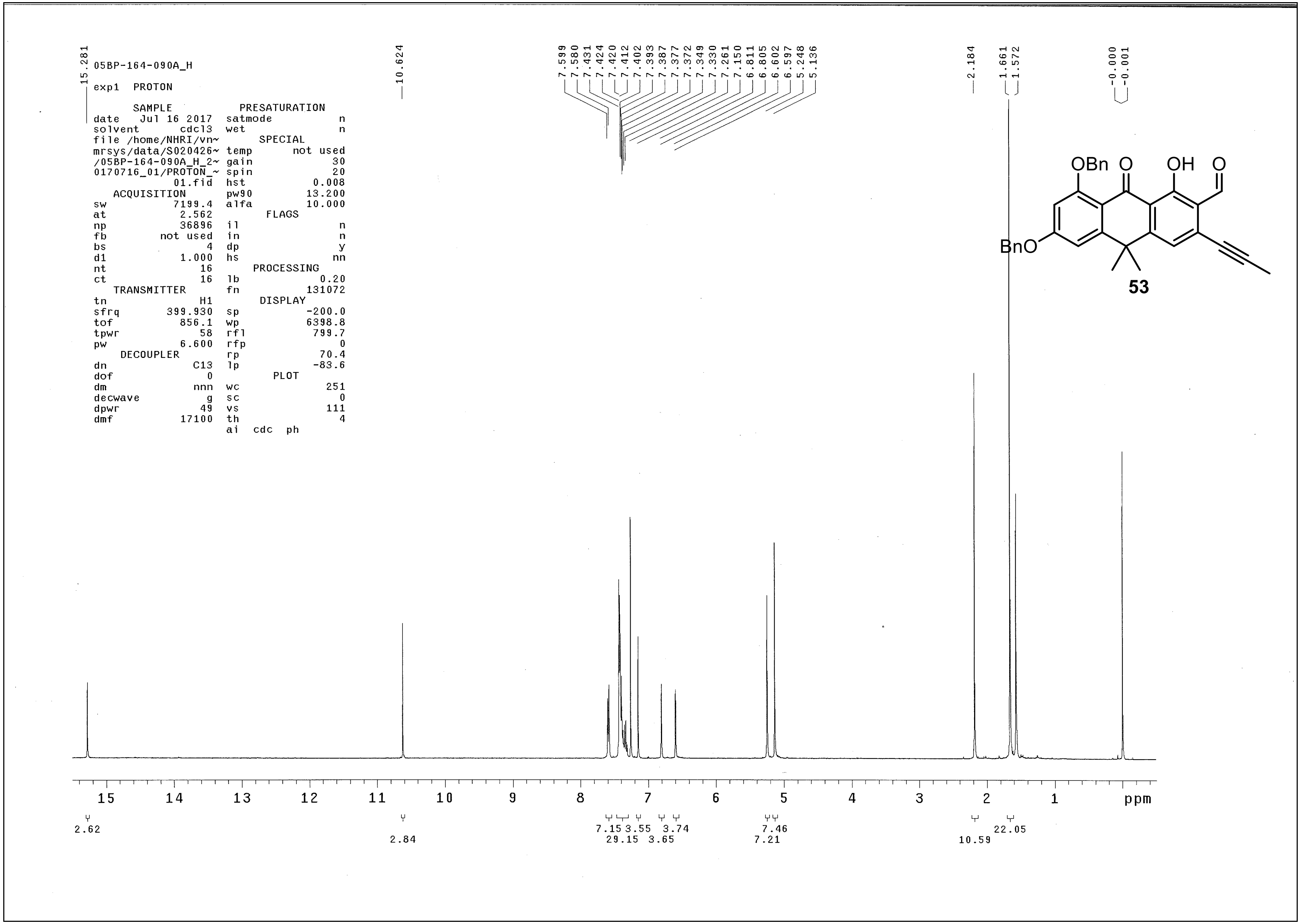

^{1}H NMR spectra for compound **53**

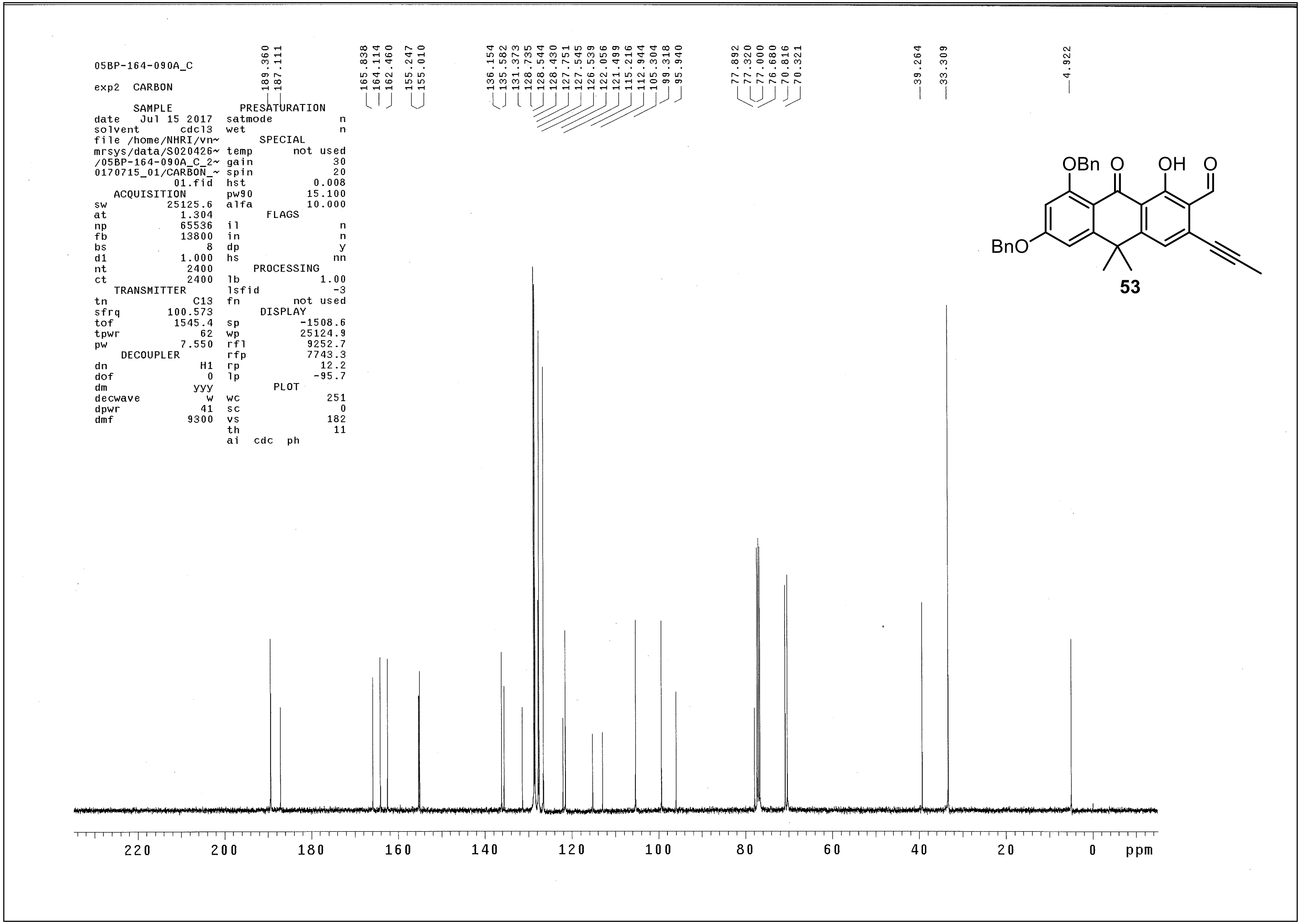

^{13}C NMR spectra for compound **53**

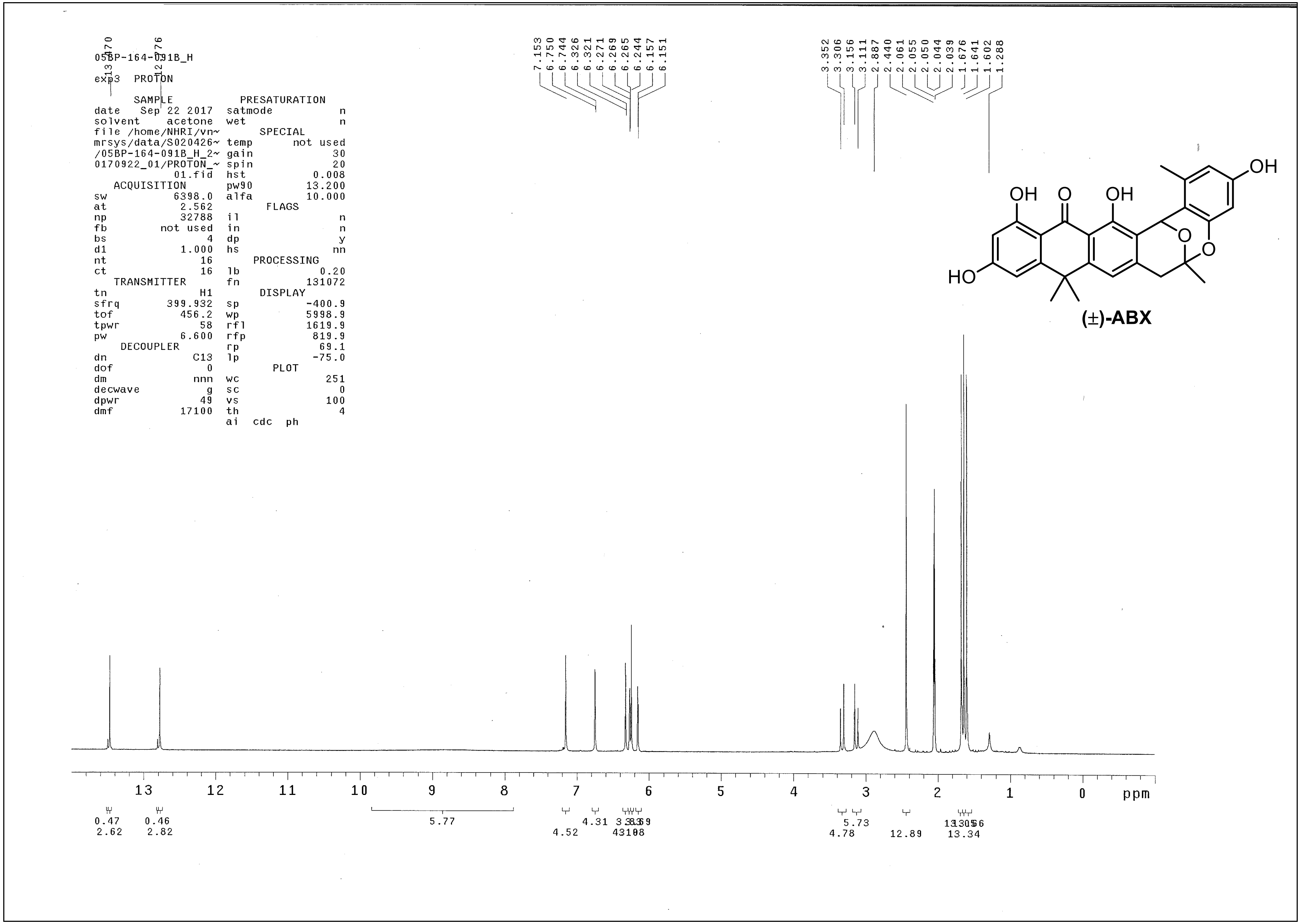

^{1}H NMR spectra for compound **(±)-ABX** in acetone-d_6

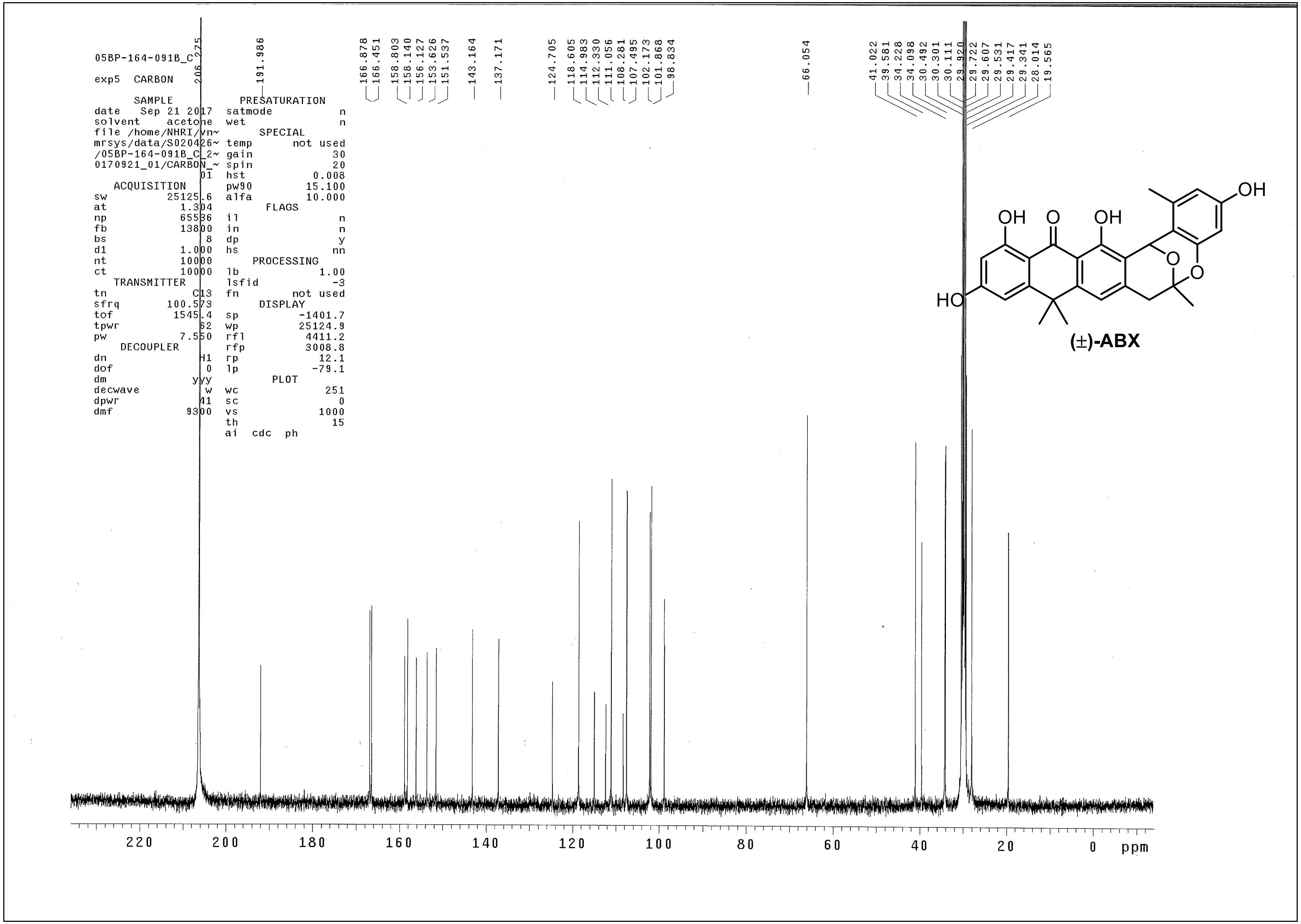

^{13}C NMR spectra for compound **(±)-ABX** in acetone-d_6

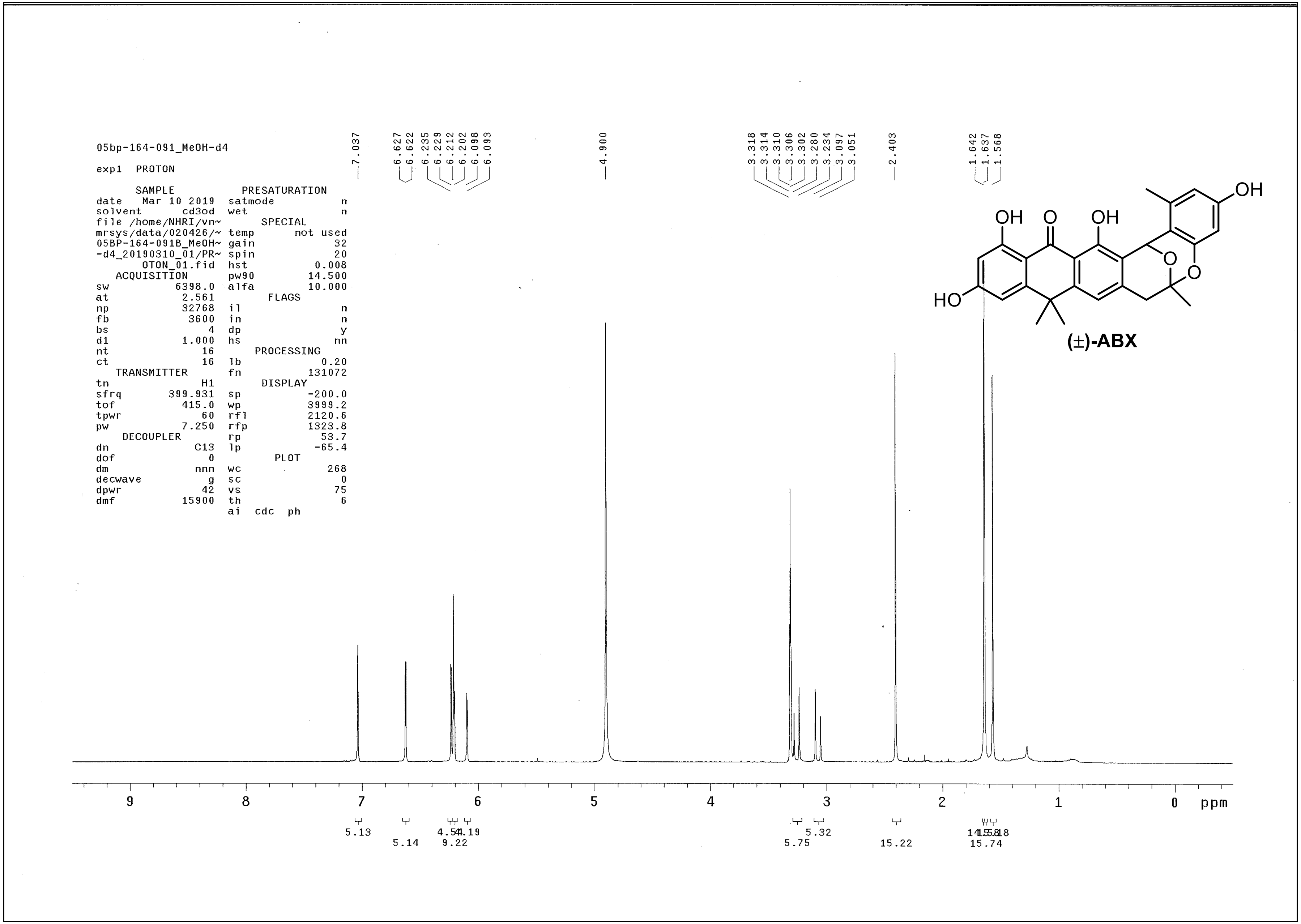

^{1}H NMR spectra for compound **(±)-ABX** in methanol-d$_4$

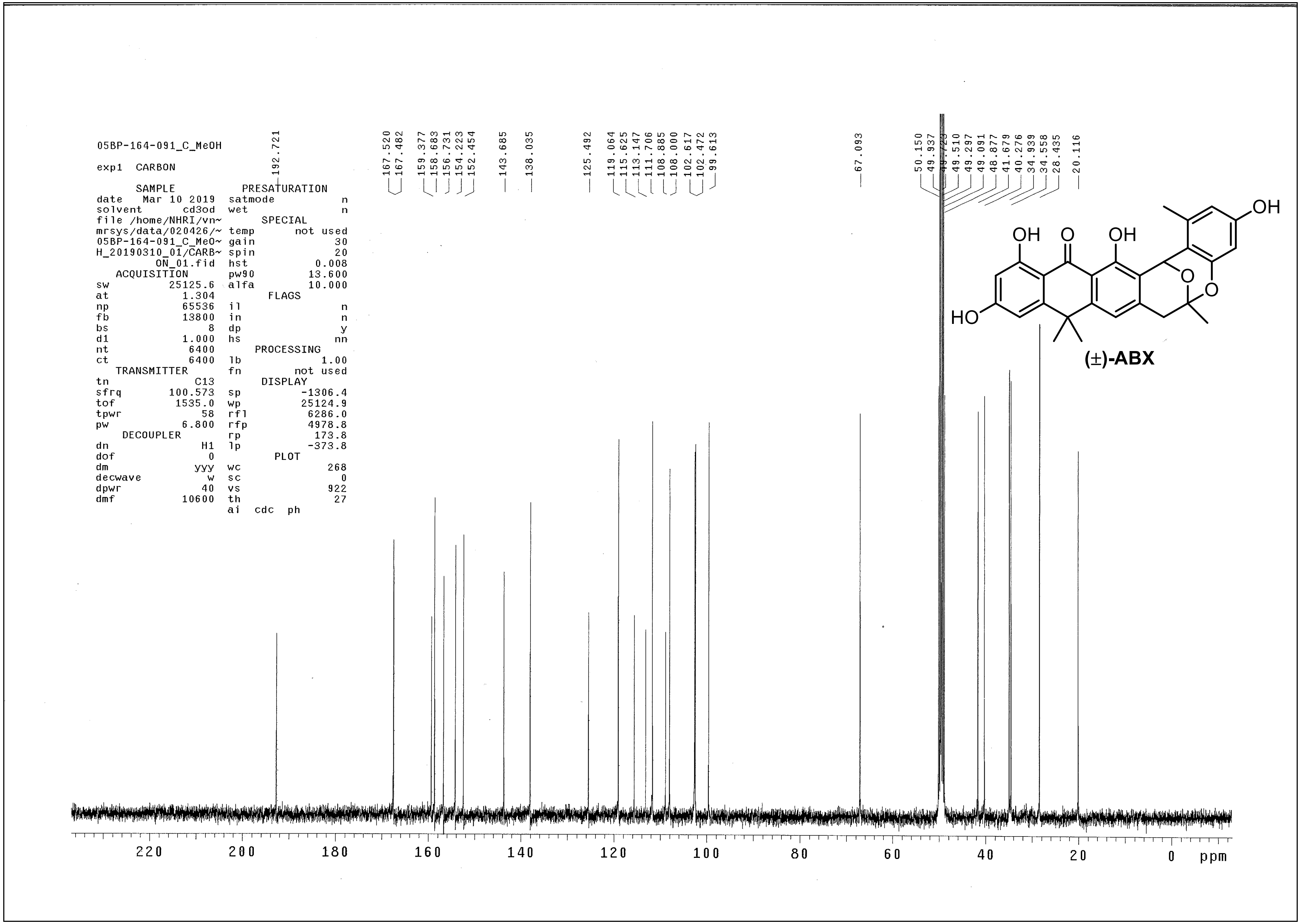

^{13}C NMR spectra for compoun**d (±)-ABX** in methanol- d_4

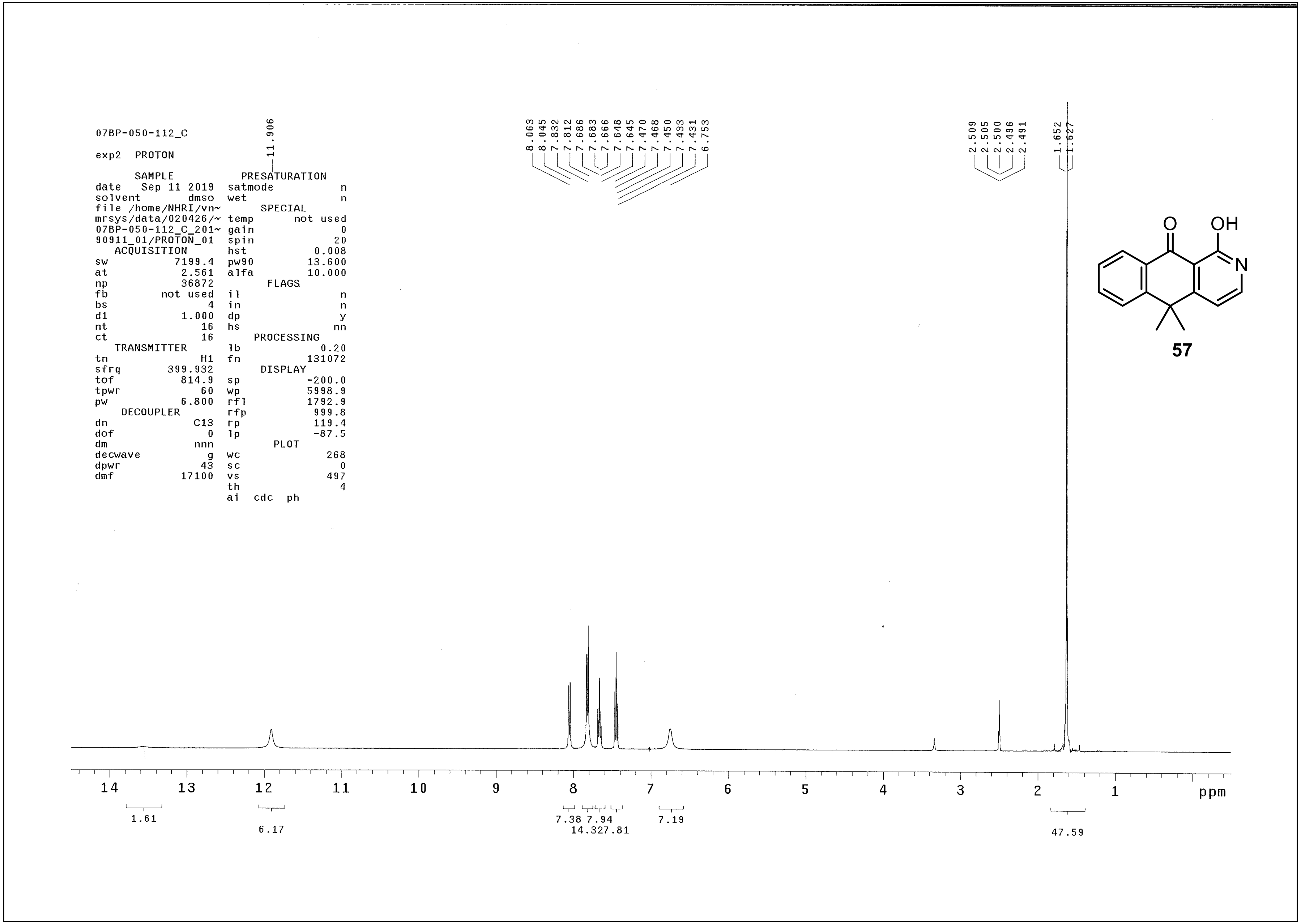

^{1}H-NMR spectra for compound **57**

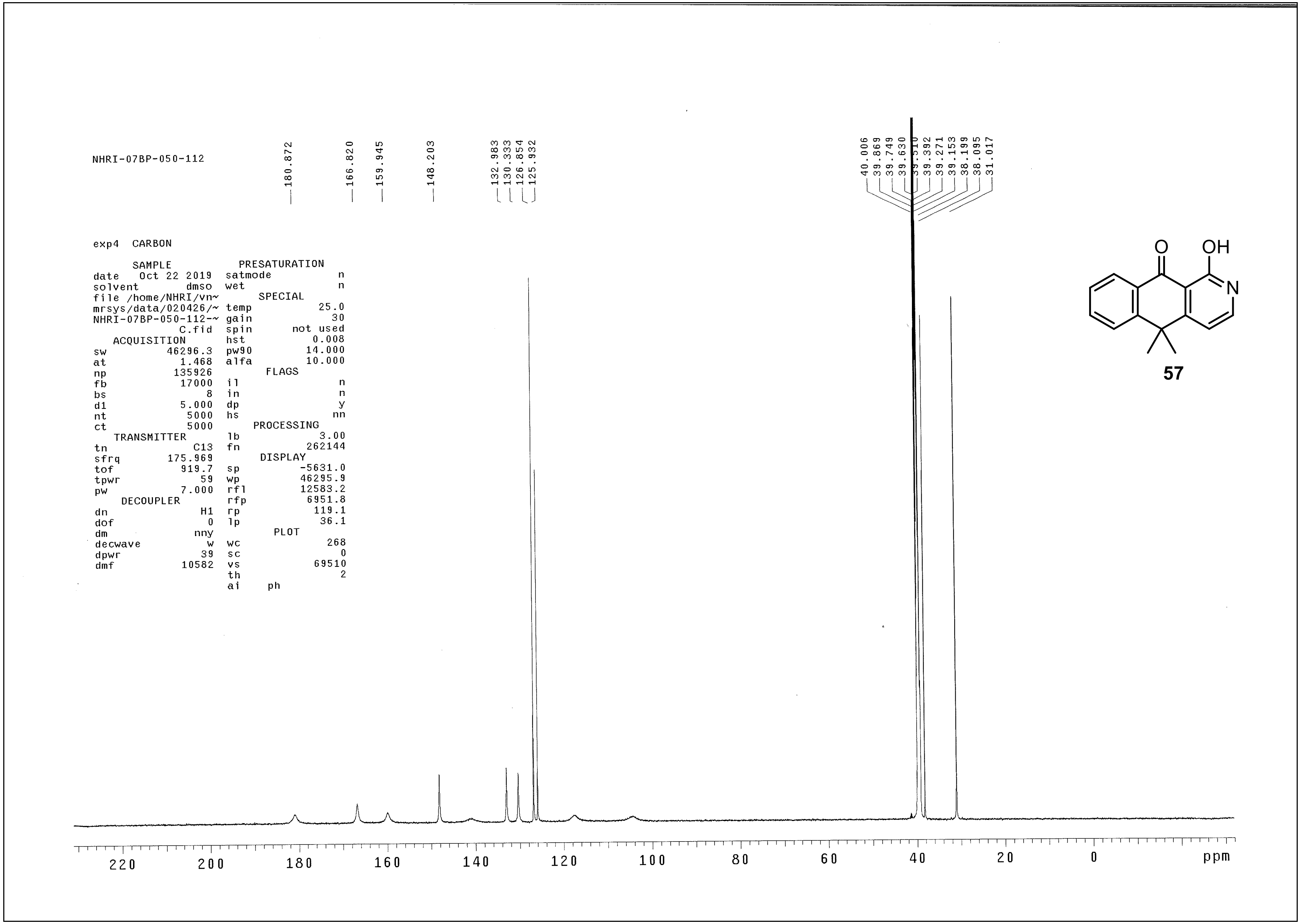

^{13}C-NMR spectra for compound **57**

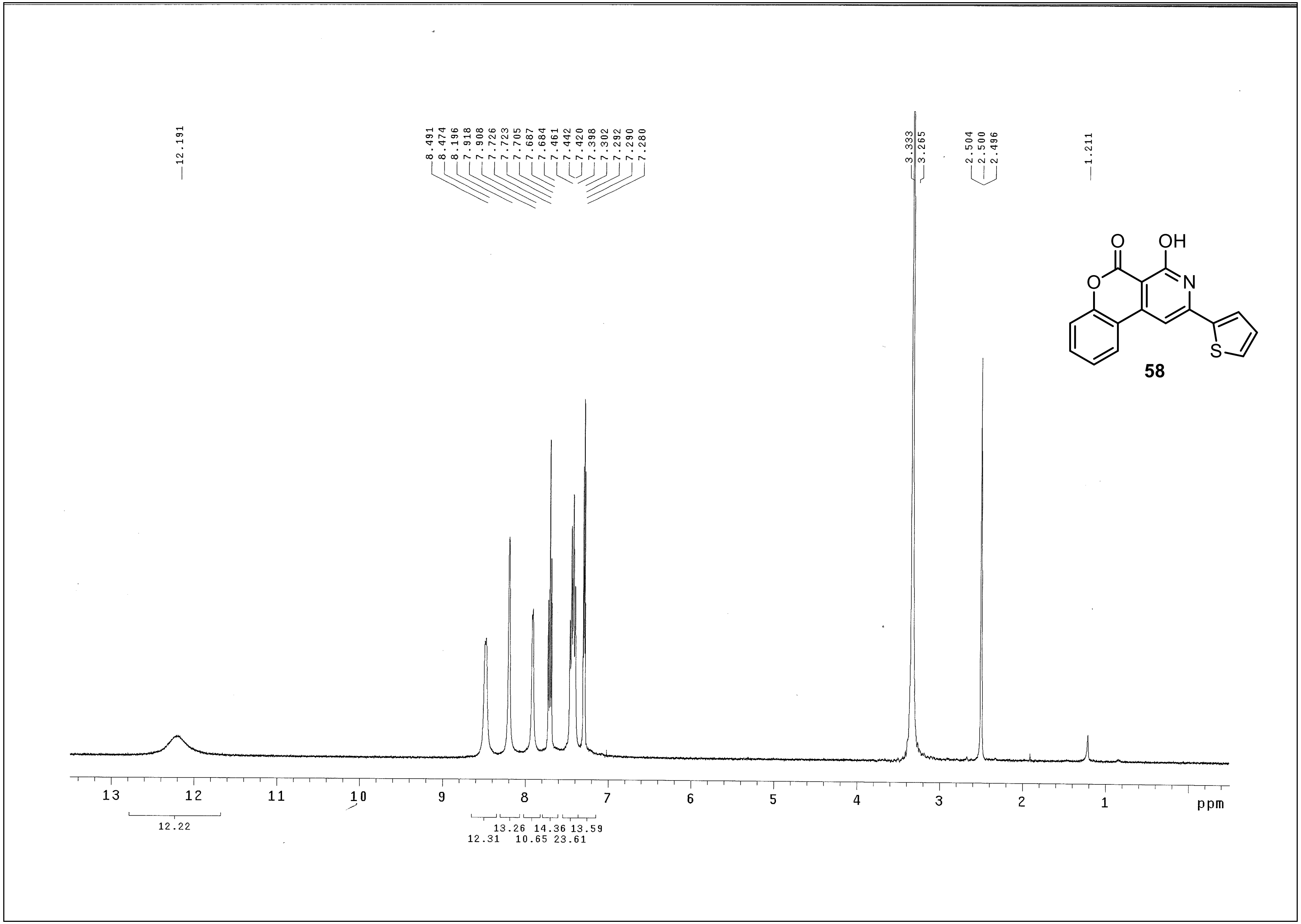

^1H NMR spectra for compound **58** in DMSO-d_6

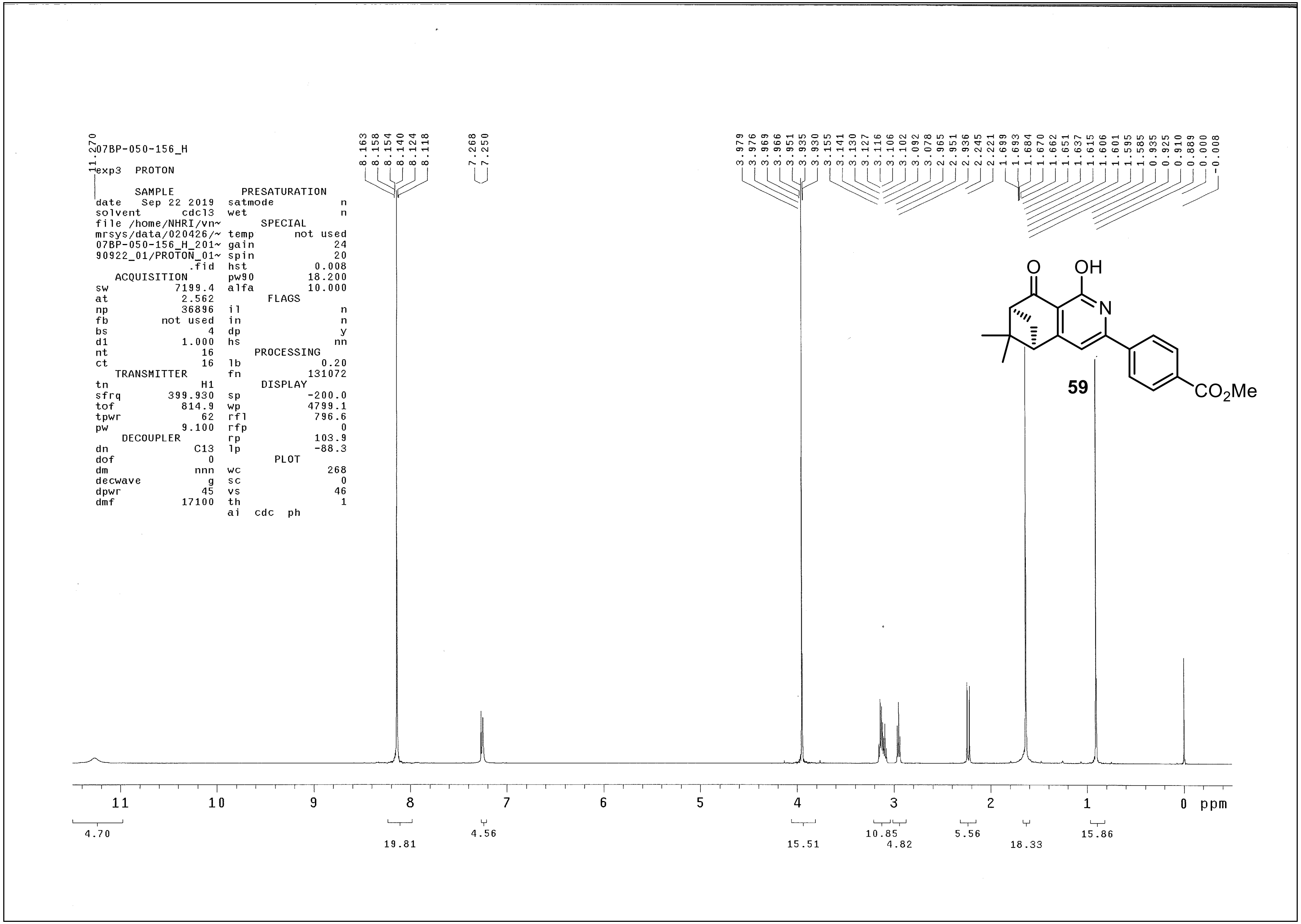

^{1}H NMR spectra for compound **59**

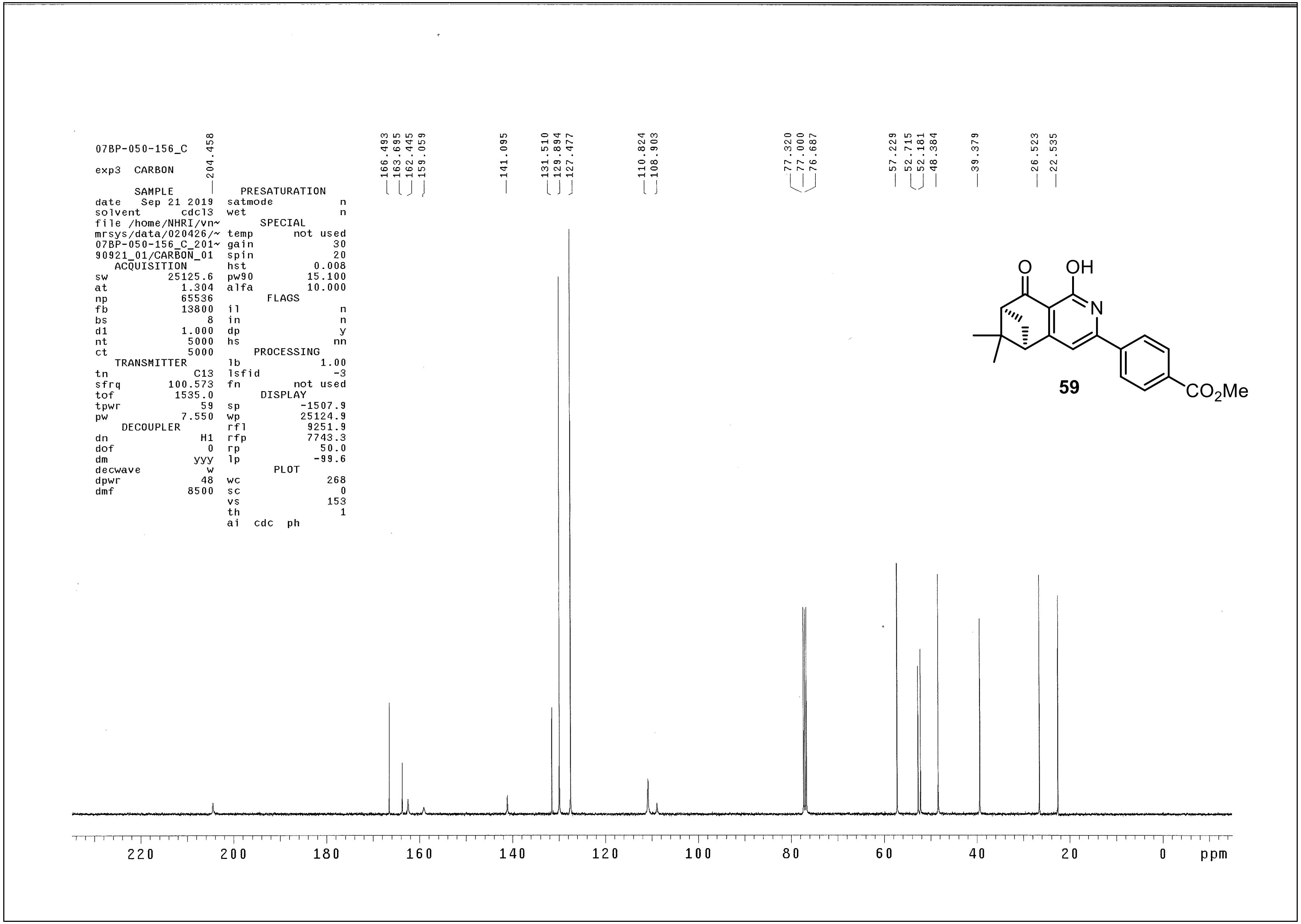

^{13}C NMR spectra for compound **59**

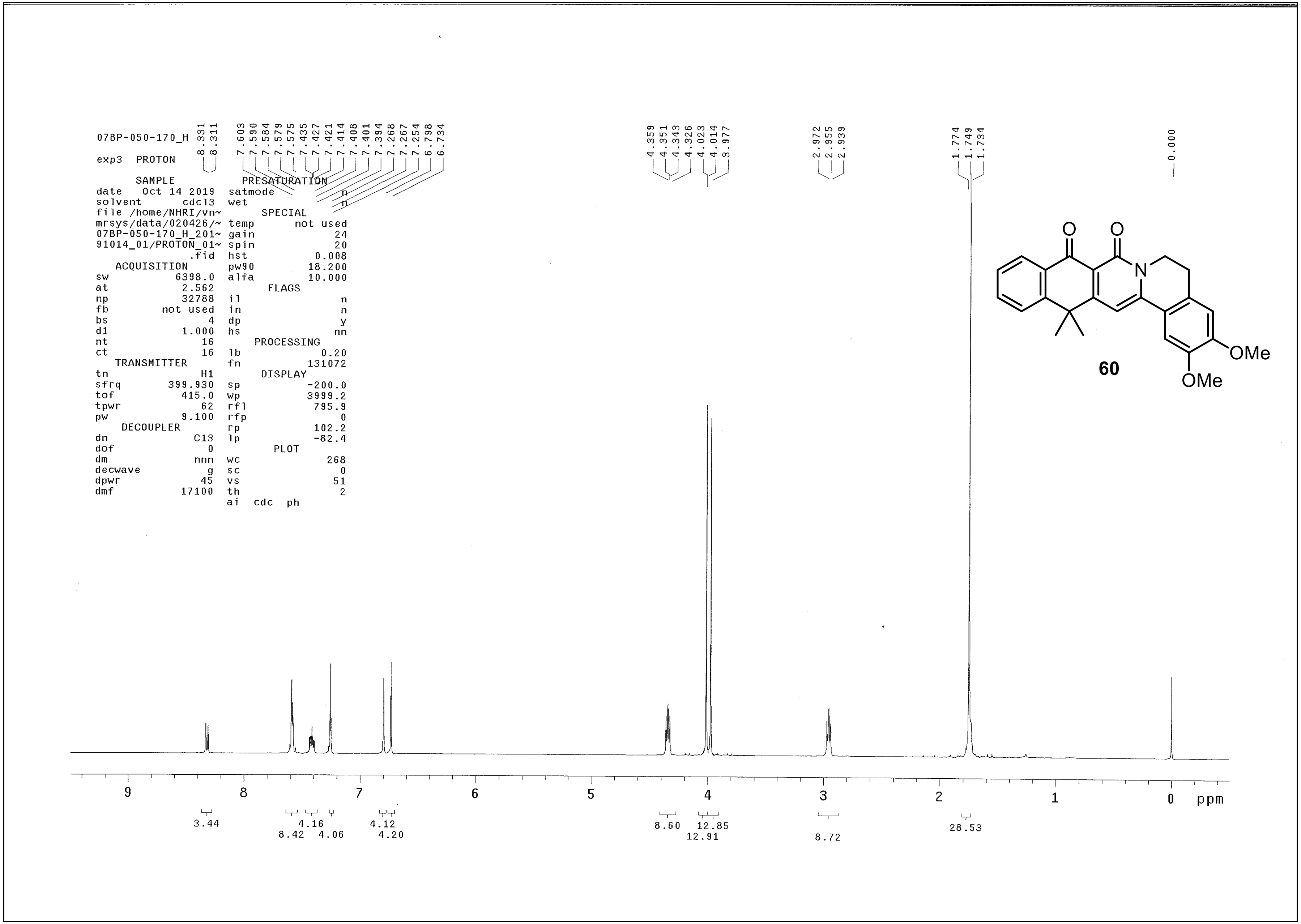

^{1}H NMR spectra for compound **60**

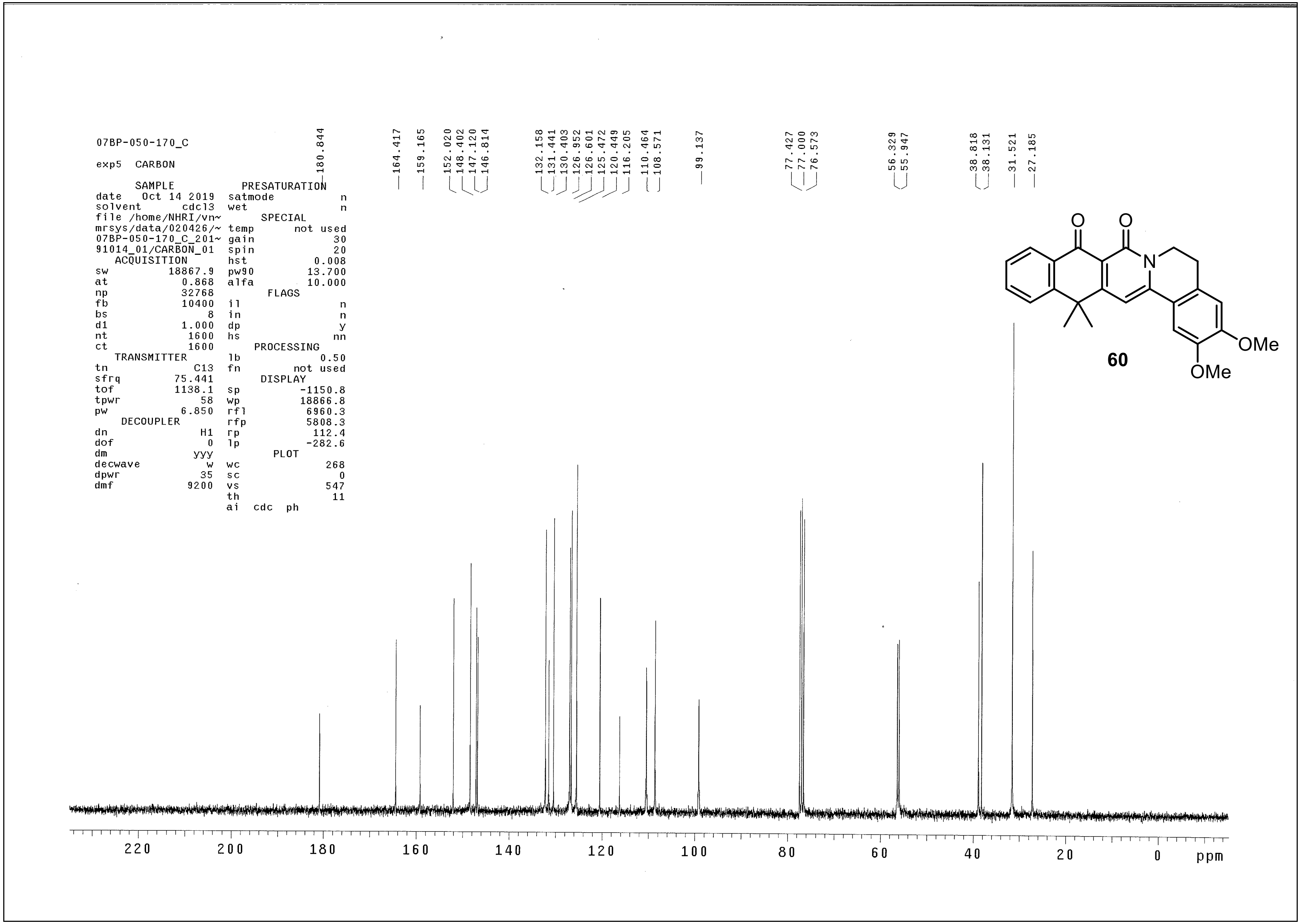

^{13}C NMR spectra for compound **60**

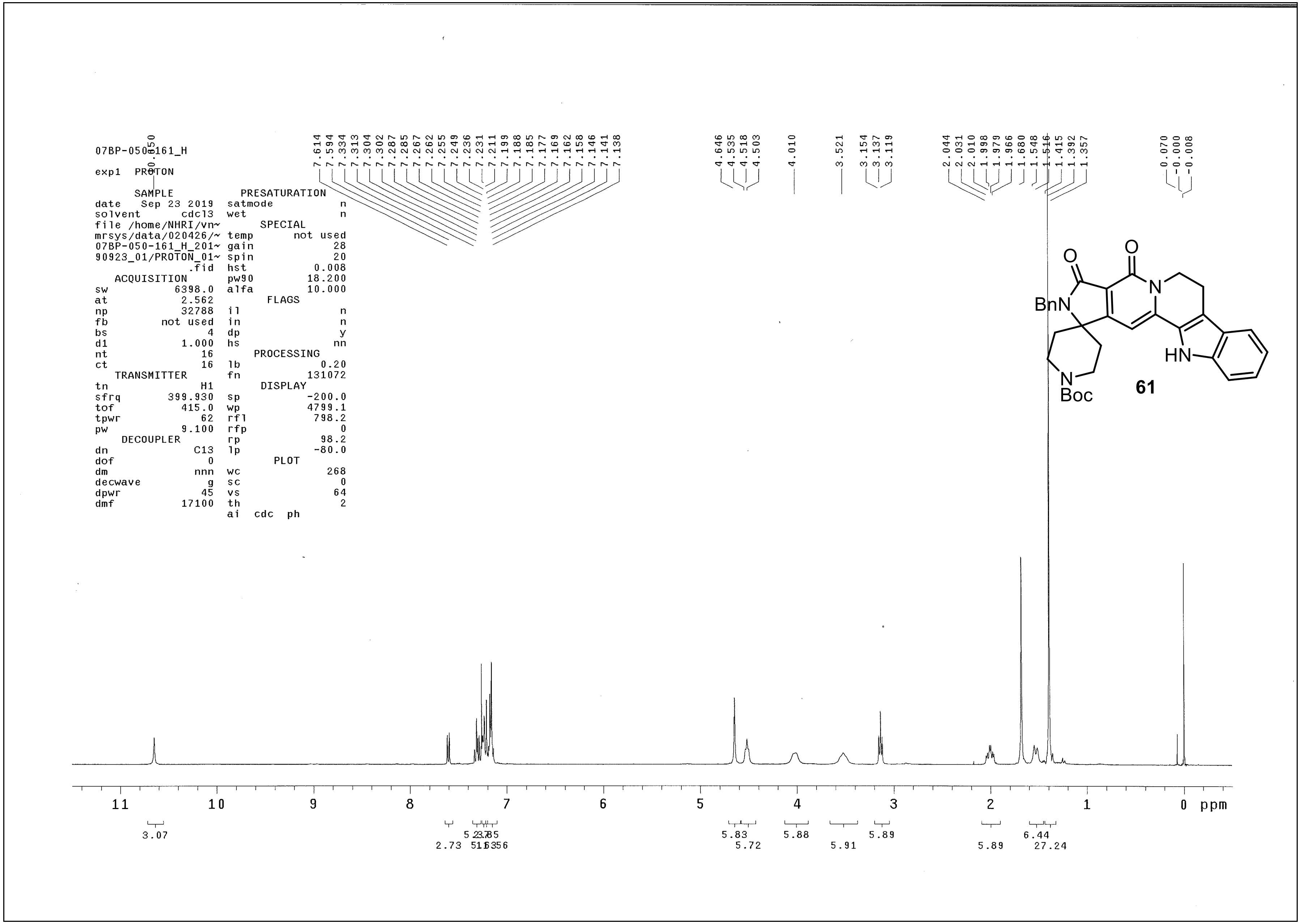

^{1}H NMR spectra for compound **61**

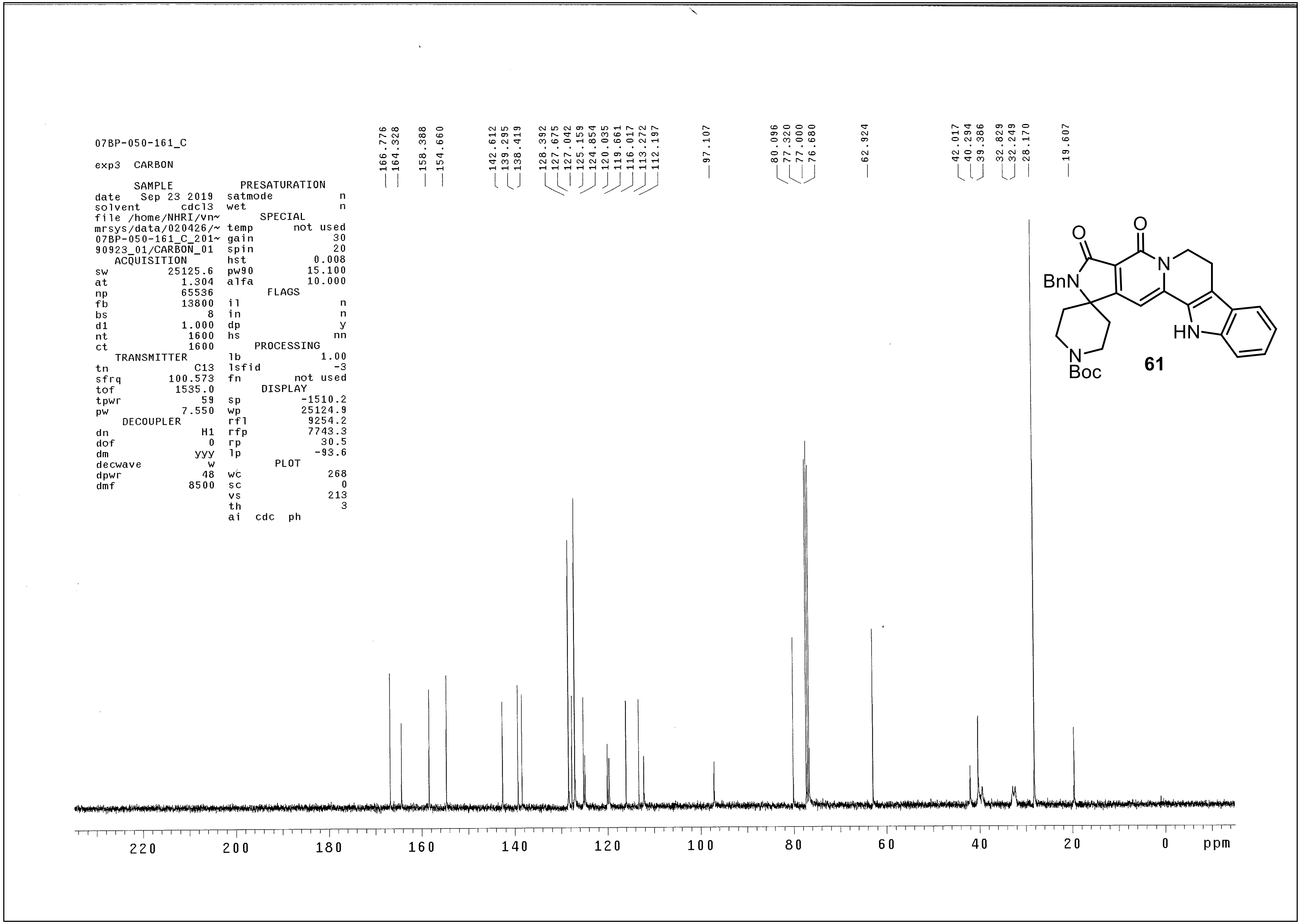

^{13}C NMR spectra for compound **61**

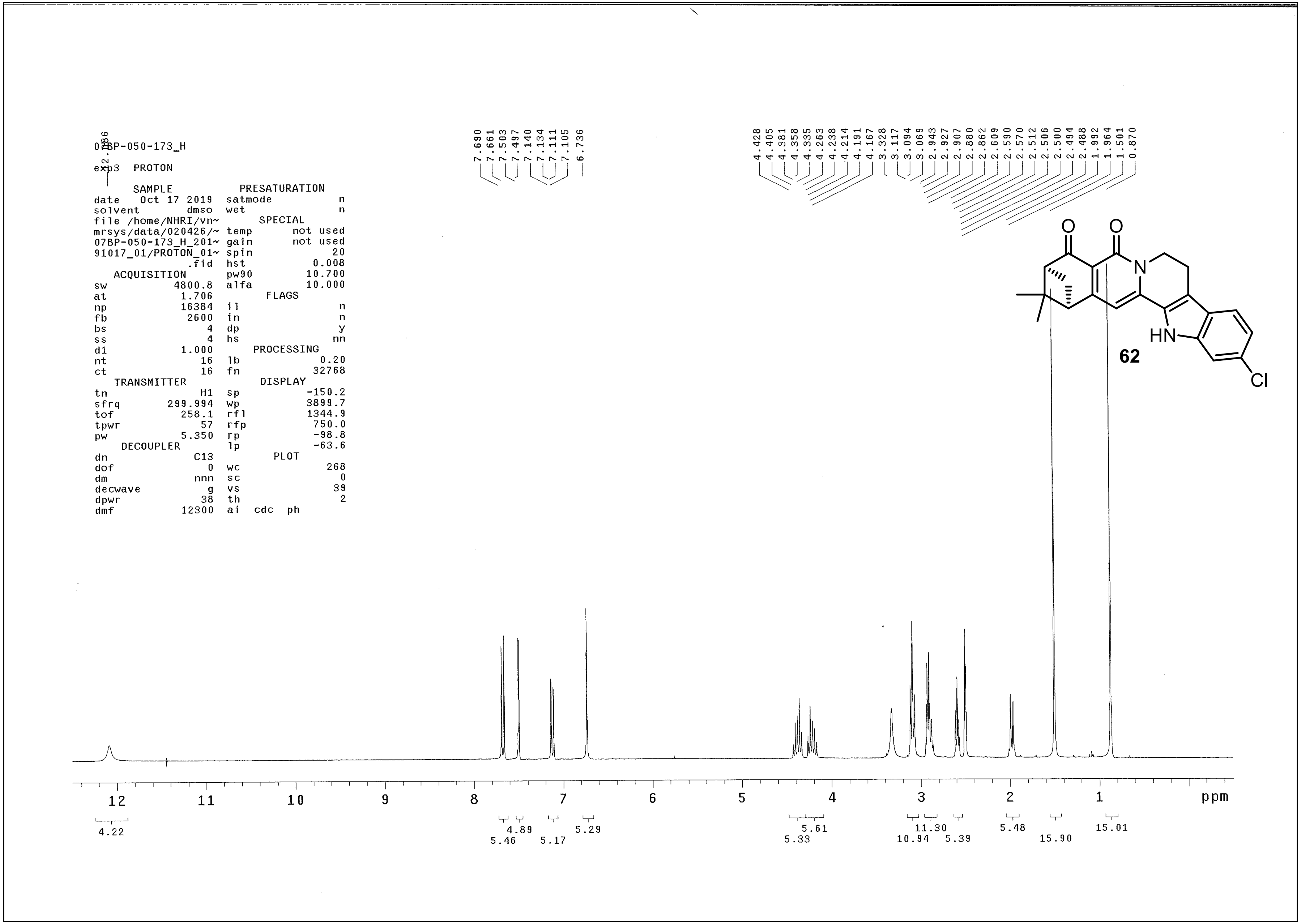

^{1}H NMR spectra for compound **62**

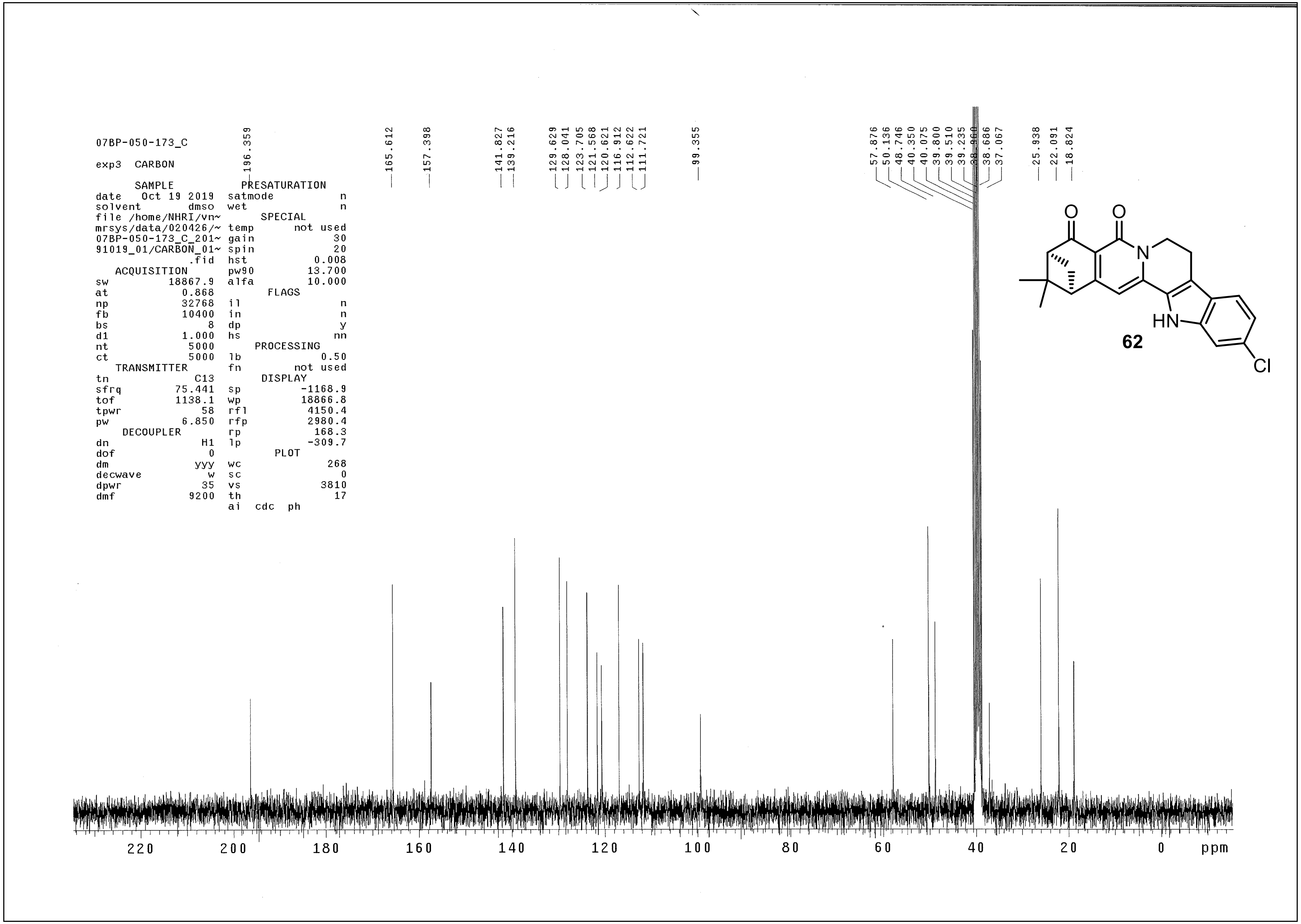

^{13}C NMR spectra for compound **62**

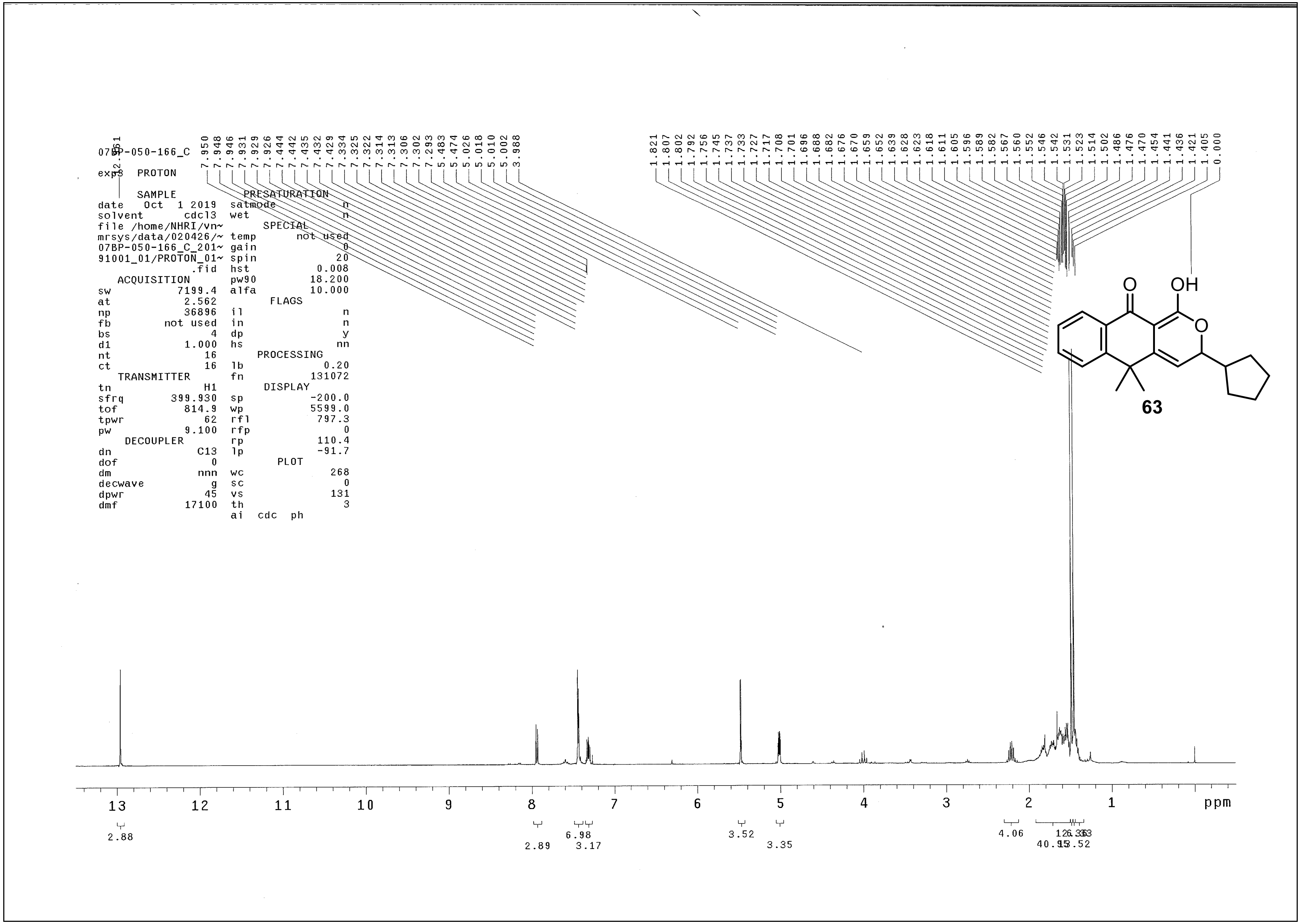

^{1}H NMR spectra for compound **63**

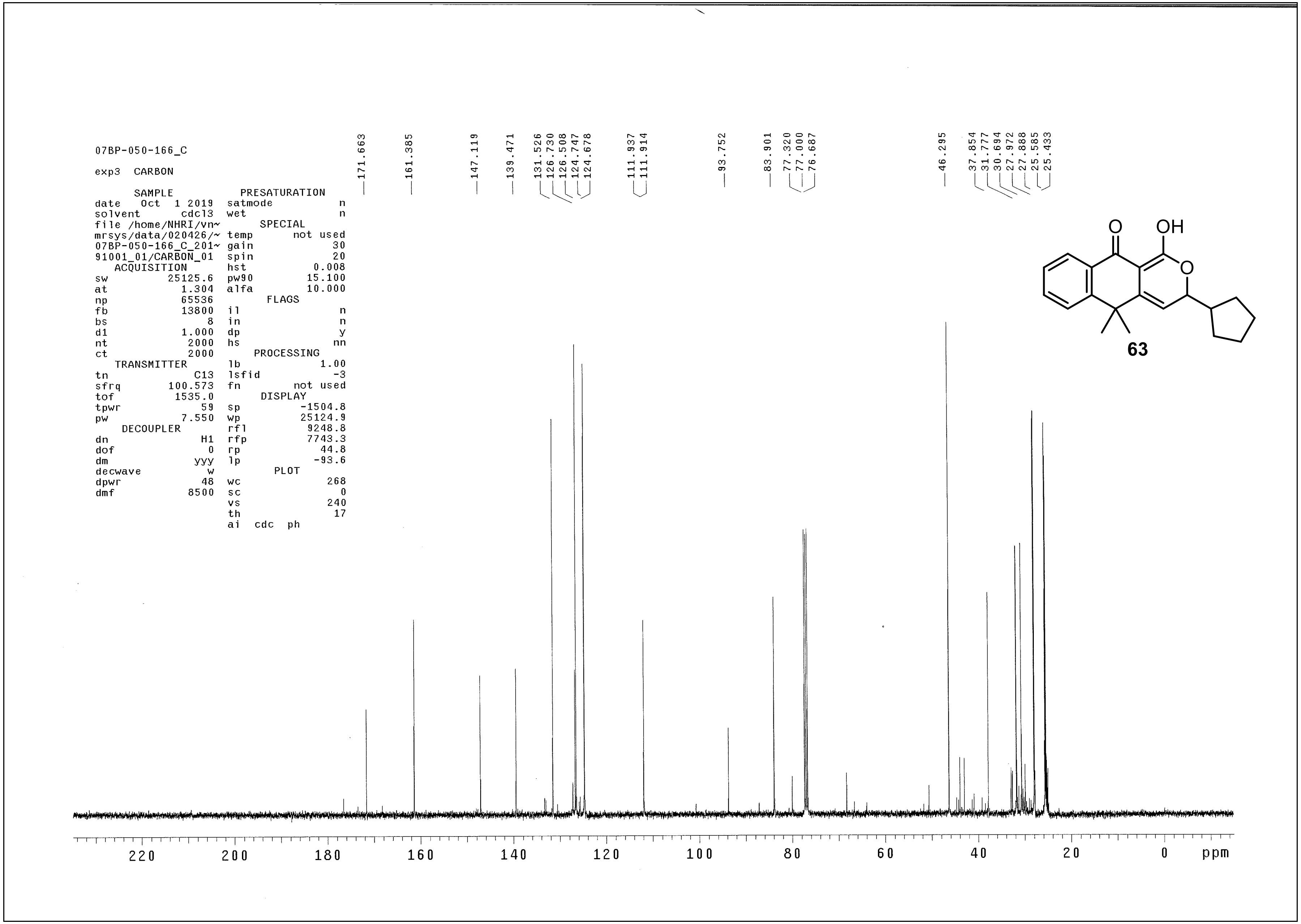

^{13}C NMR spectra for compound **63**

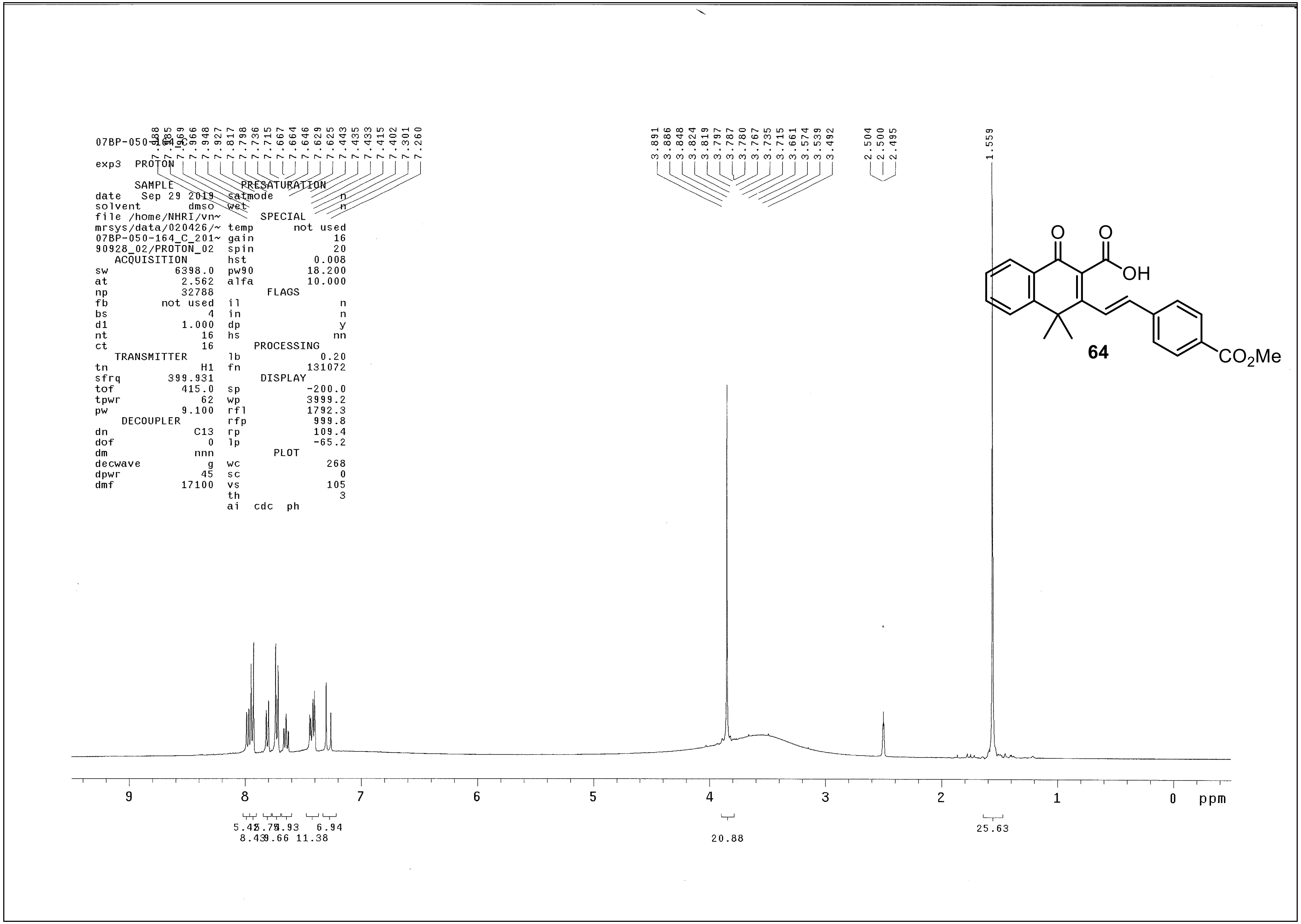

^{1}H NMR spectra for compound **64**

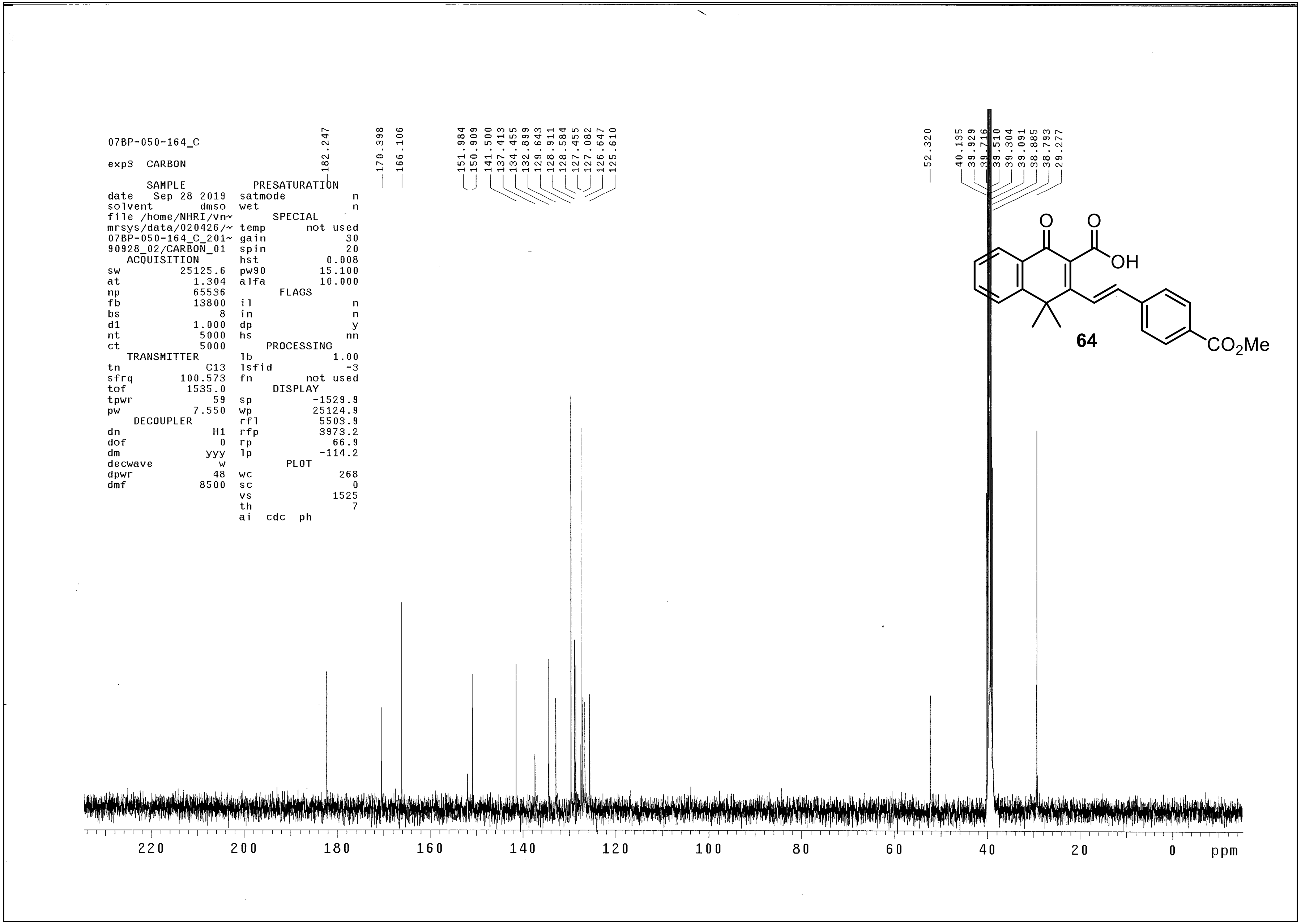

^{13}C NMR spectra for compound **64**

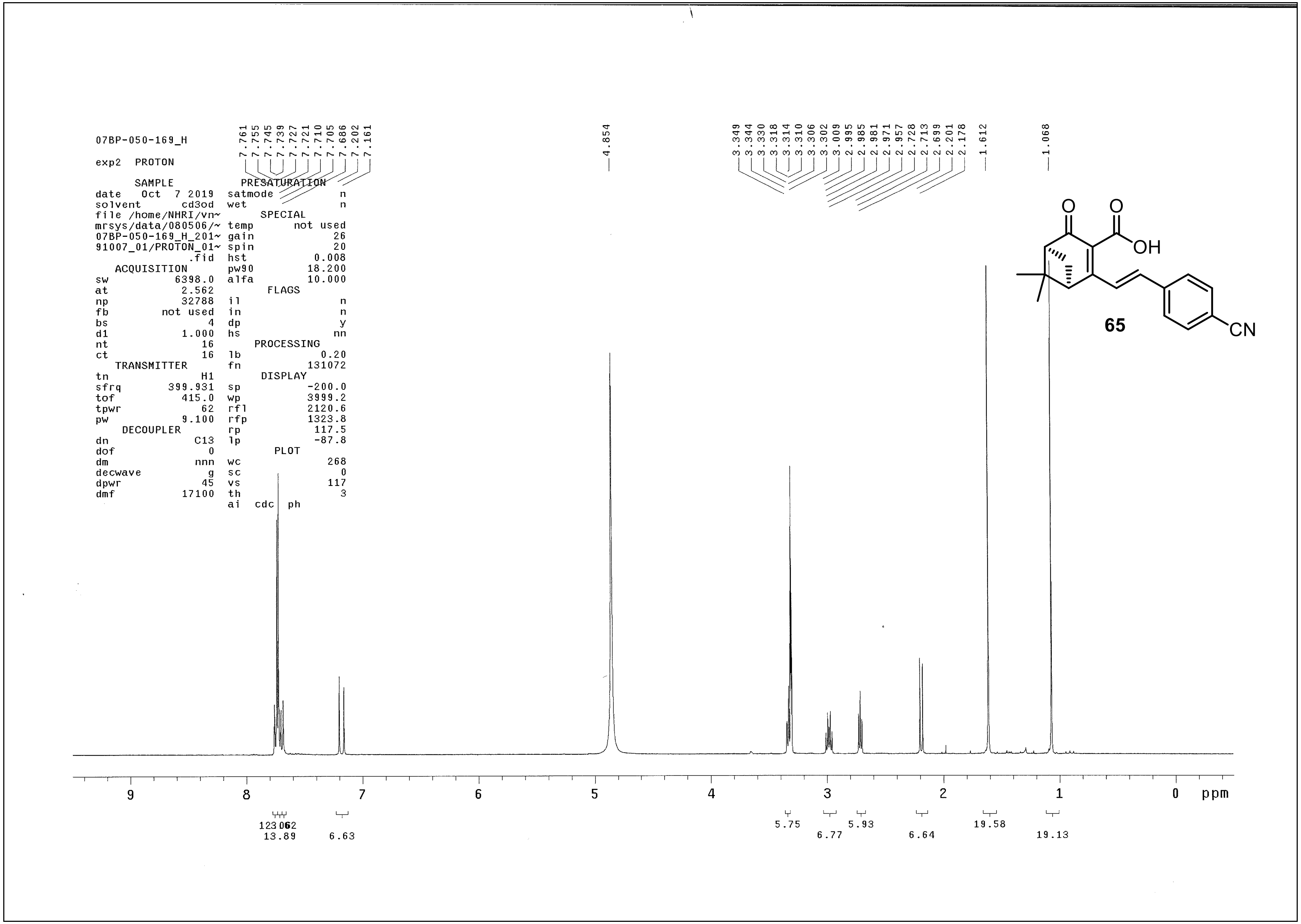

^{1}H NMR spectra for compound **65**

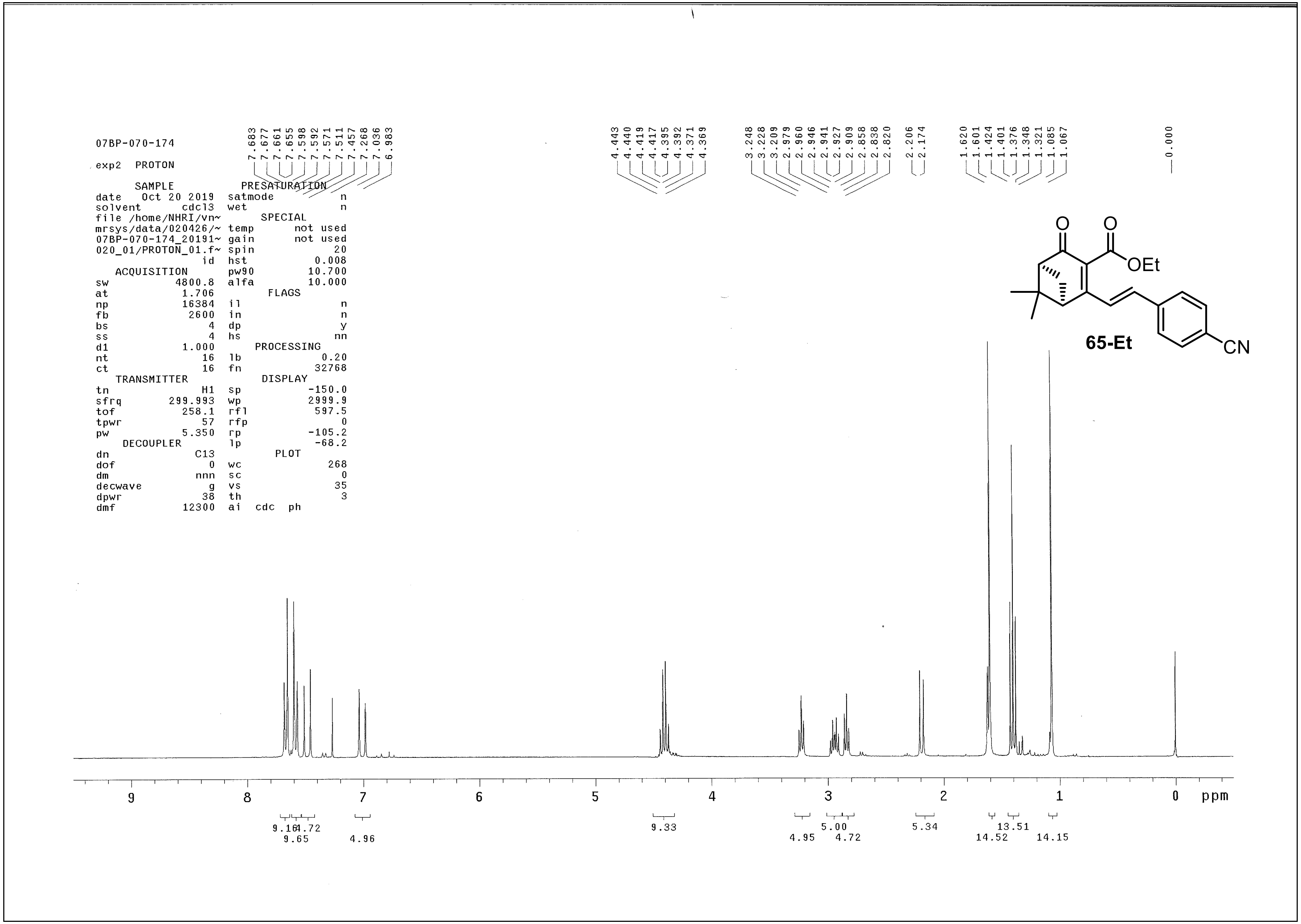

^{1}H NMR spectra for compound **65-Et**

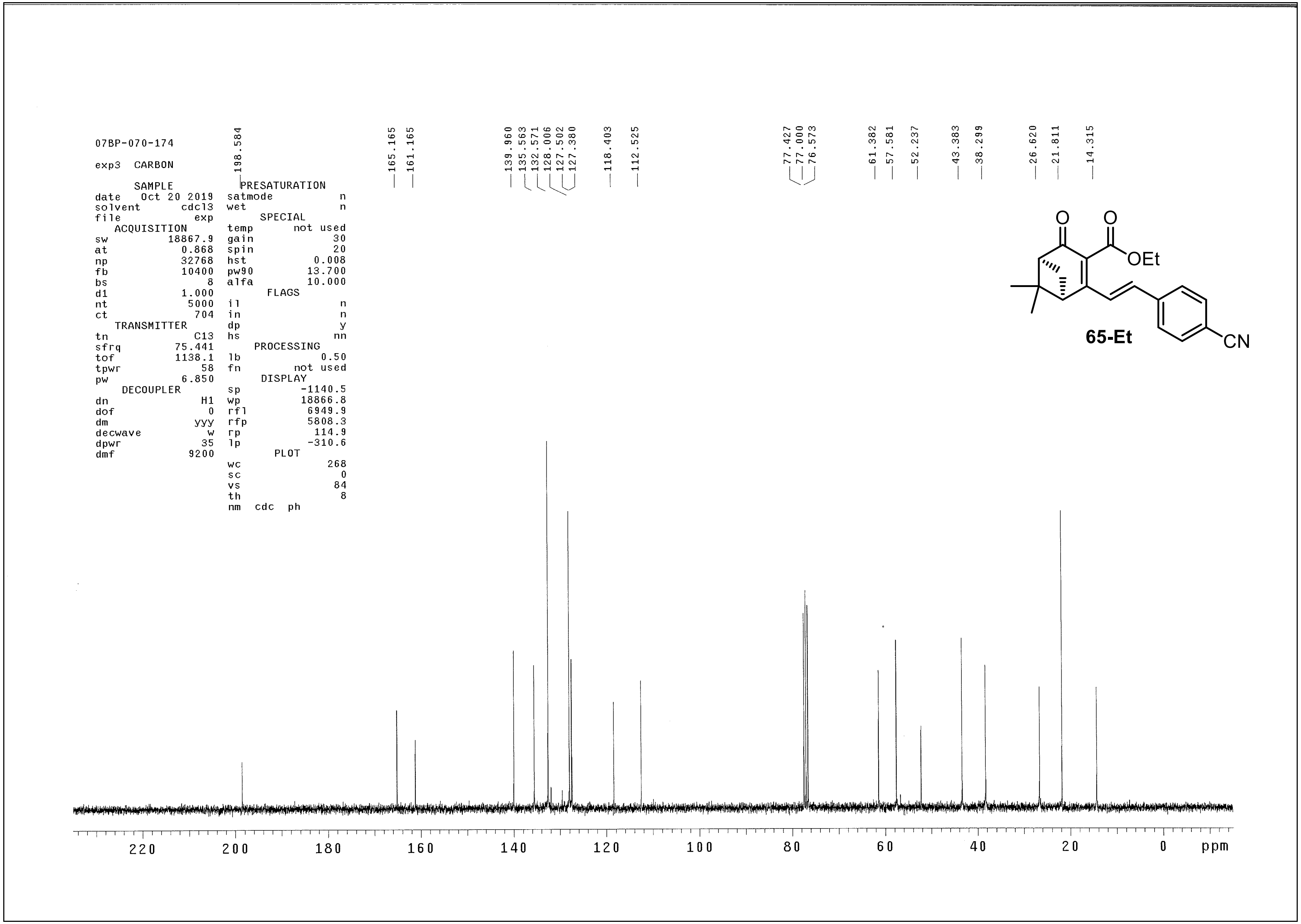

^{13}C NMR spectra for compound **65-Et**

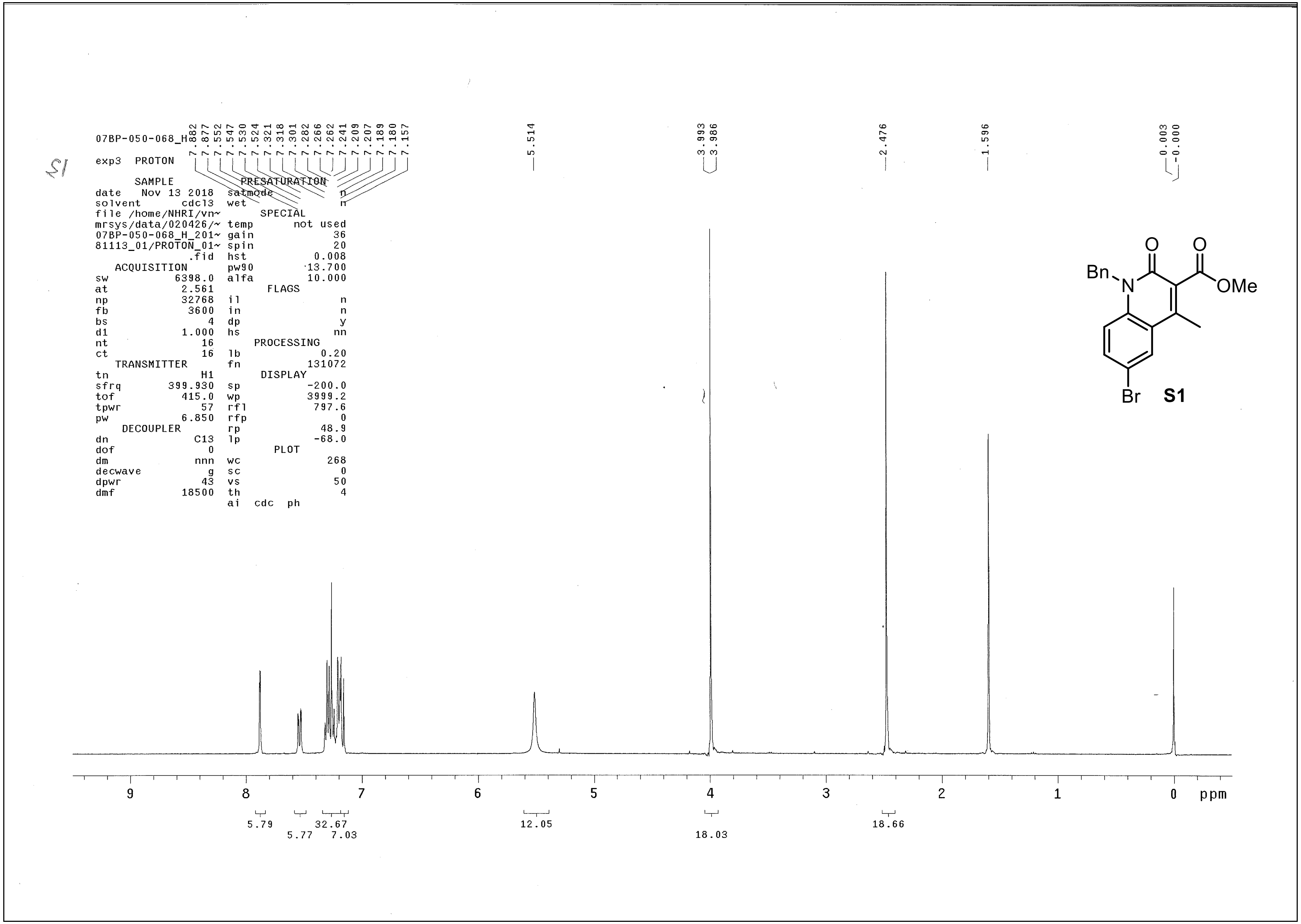

^{1}H NMR spectra for compound **S1**

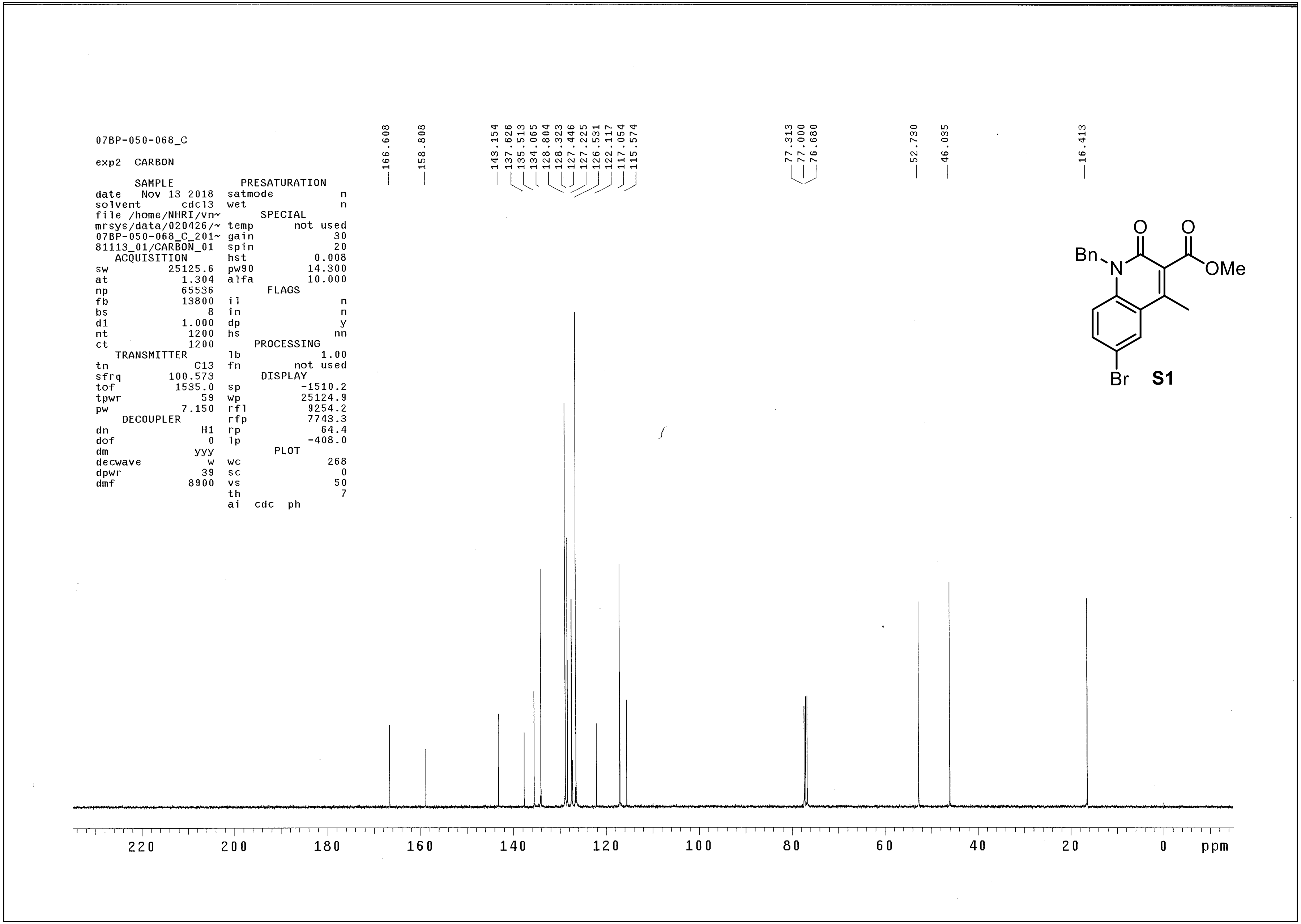

^{13}C NMR spectra for compound **S1**

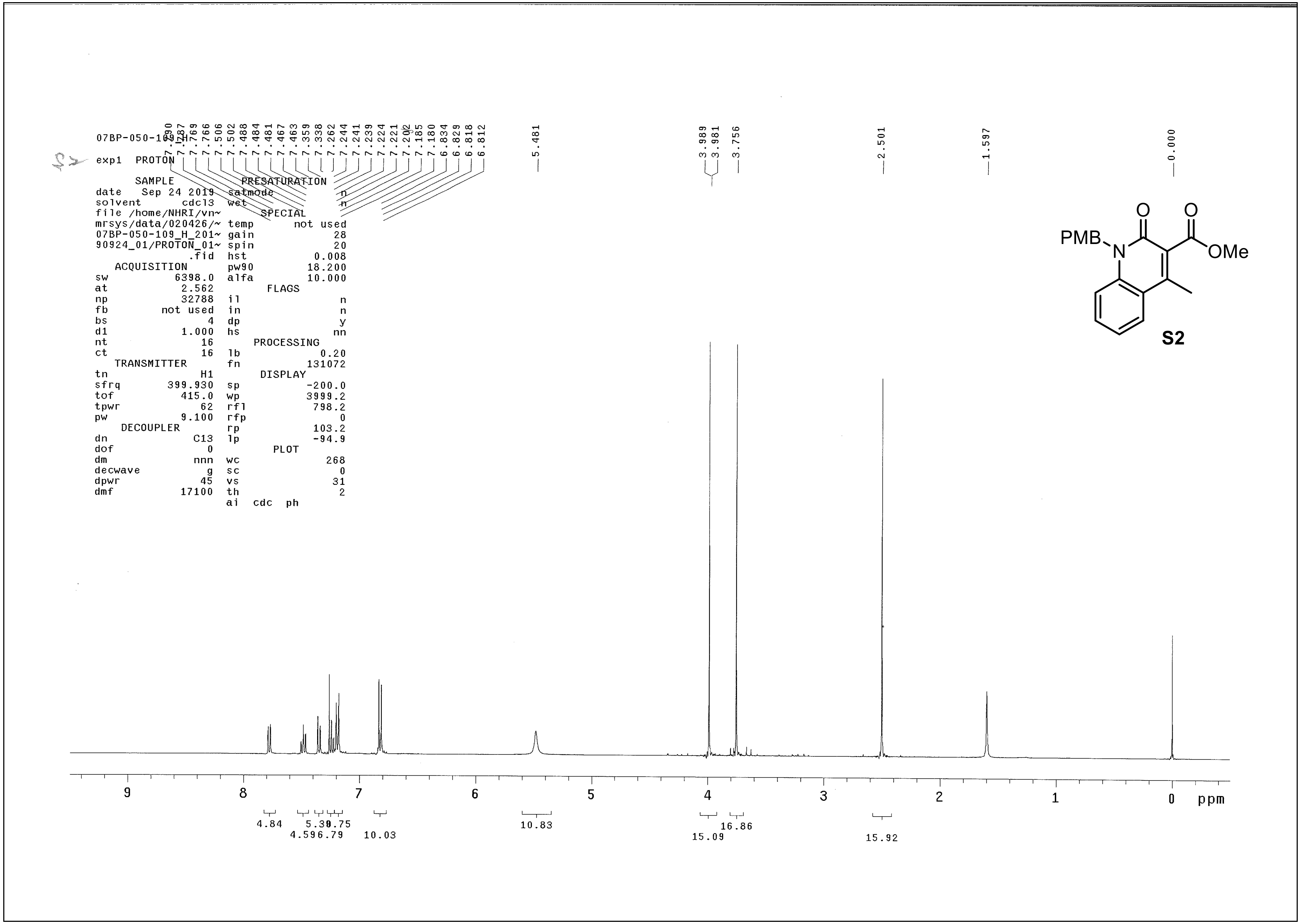

^{1}H NMR spectra for compound **S2**

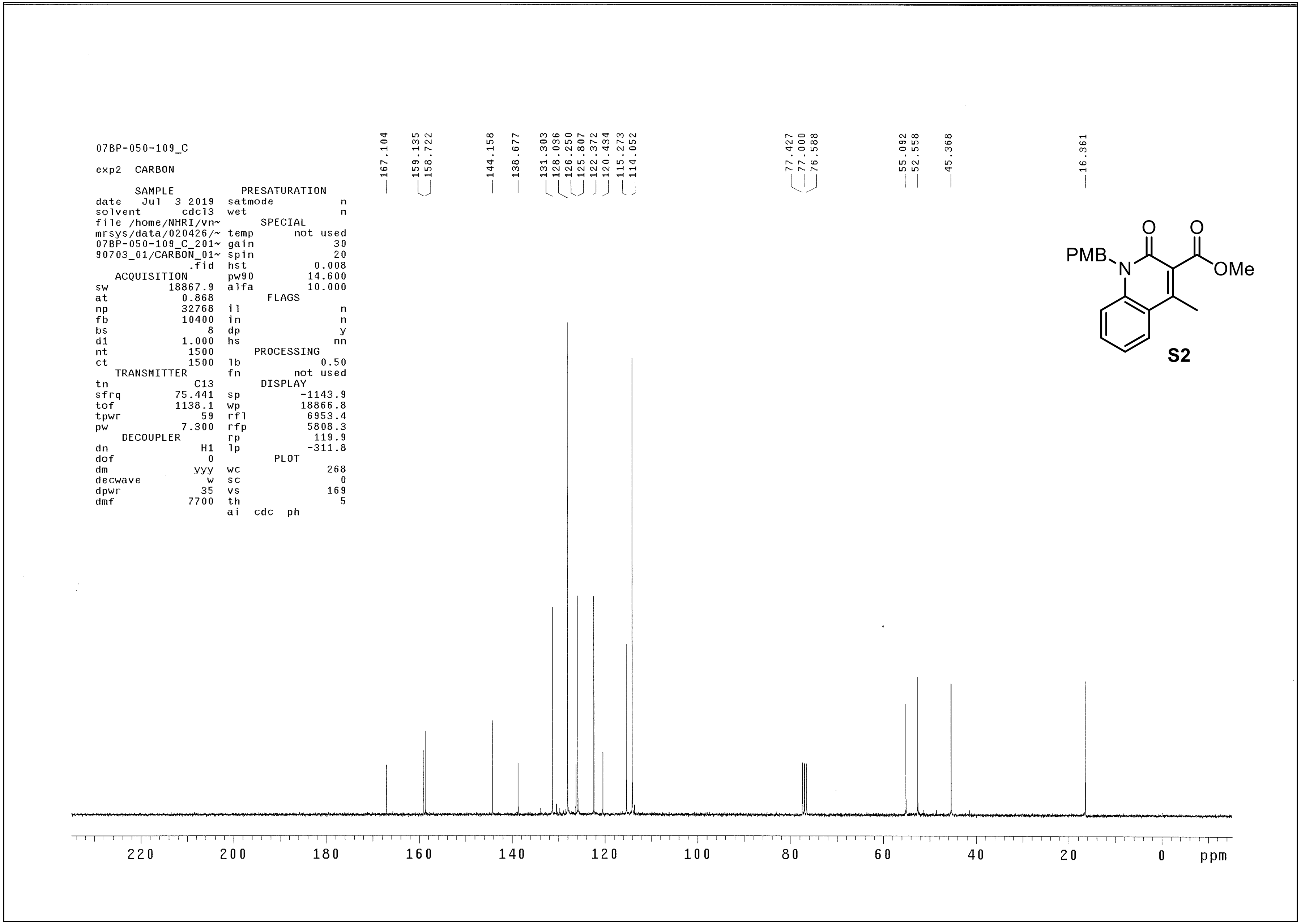

^{13}C NMR spectra for compound **S2**

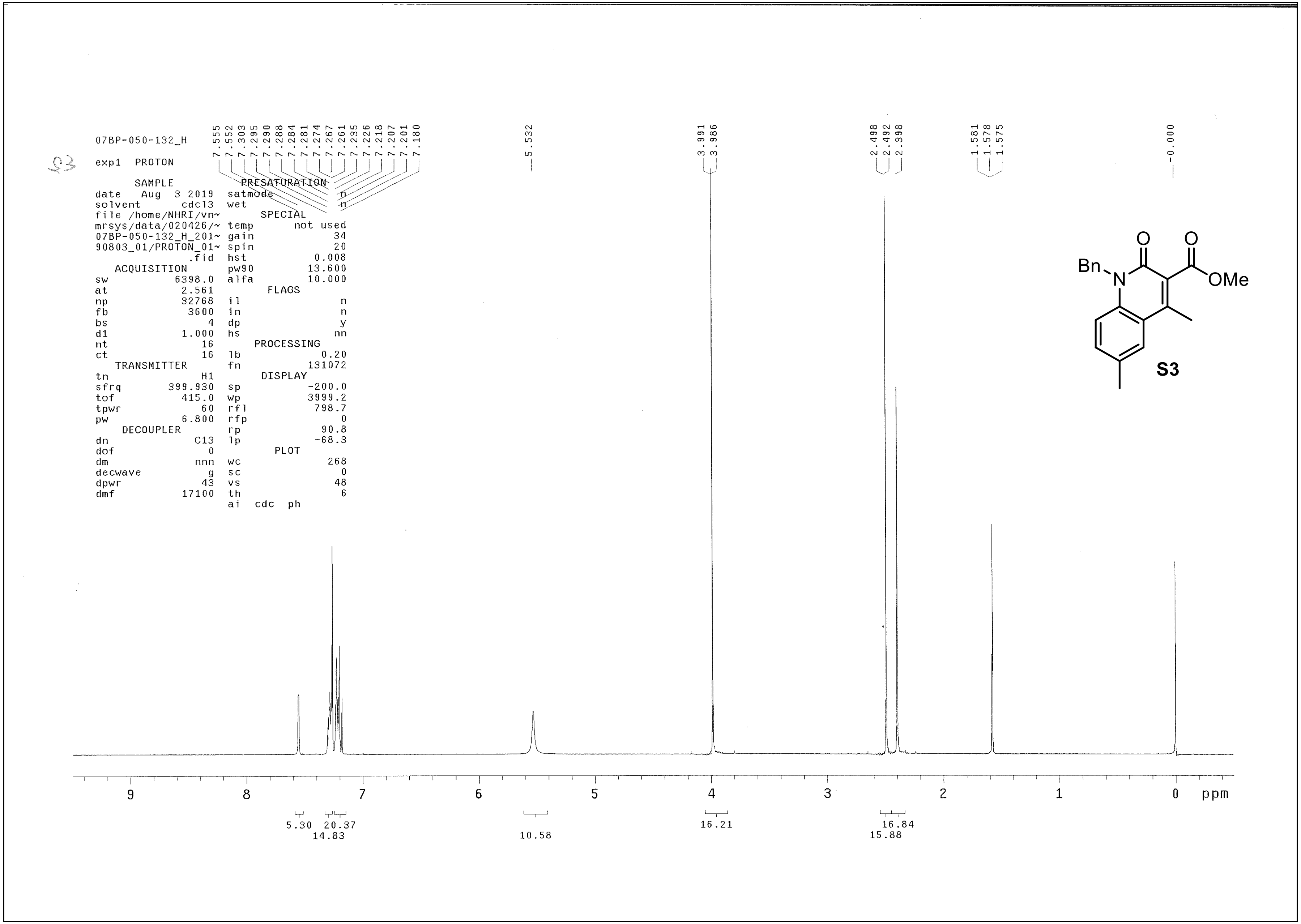

^{1}H NMR spectra for compound **S3**

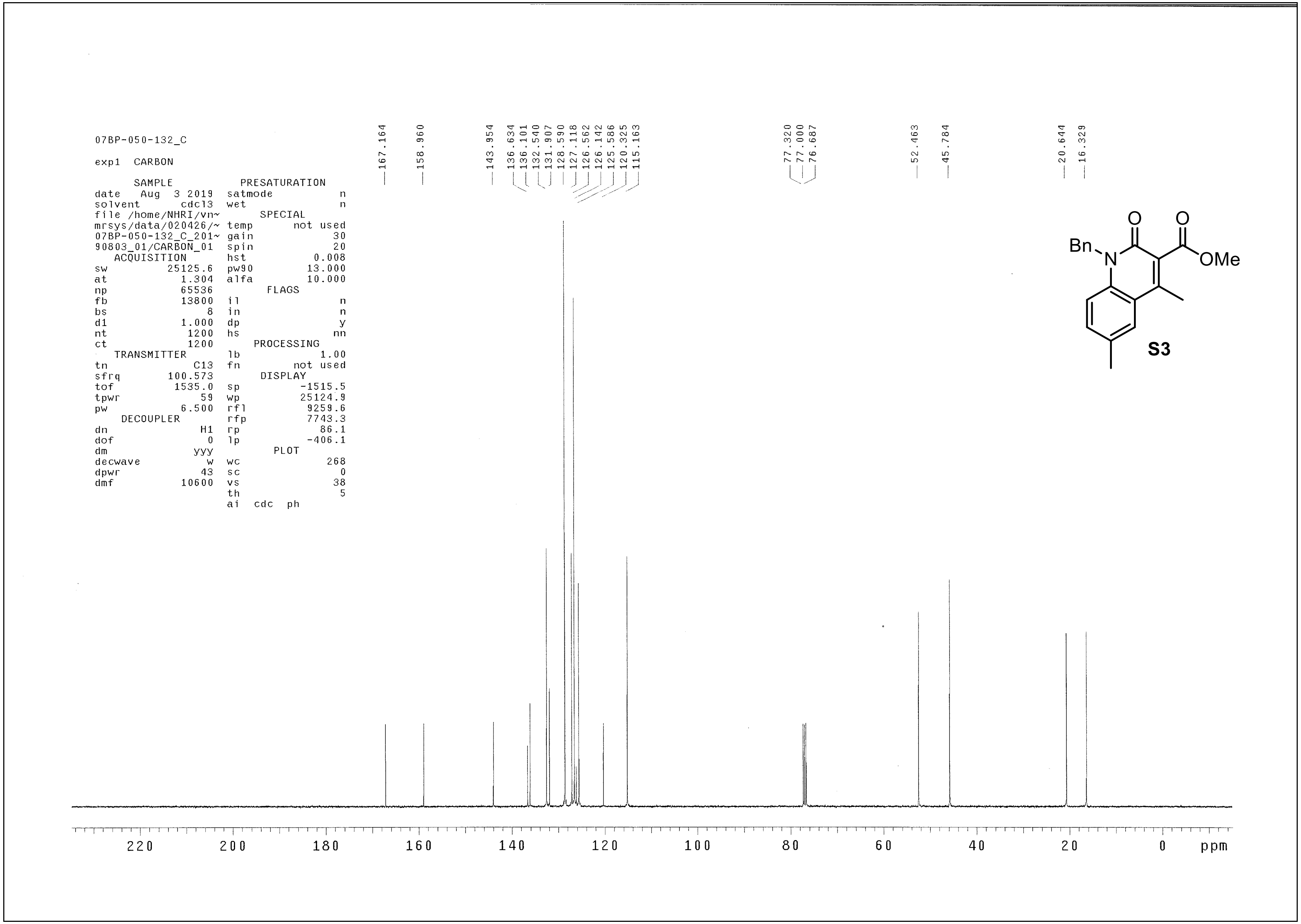

^{13}C NMR spectra for compound **S3**

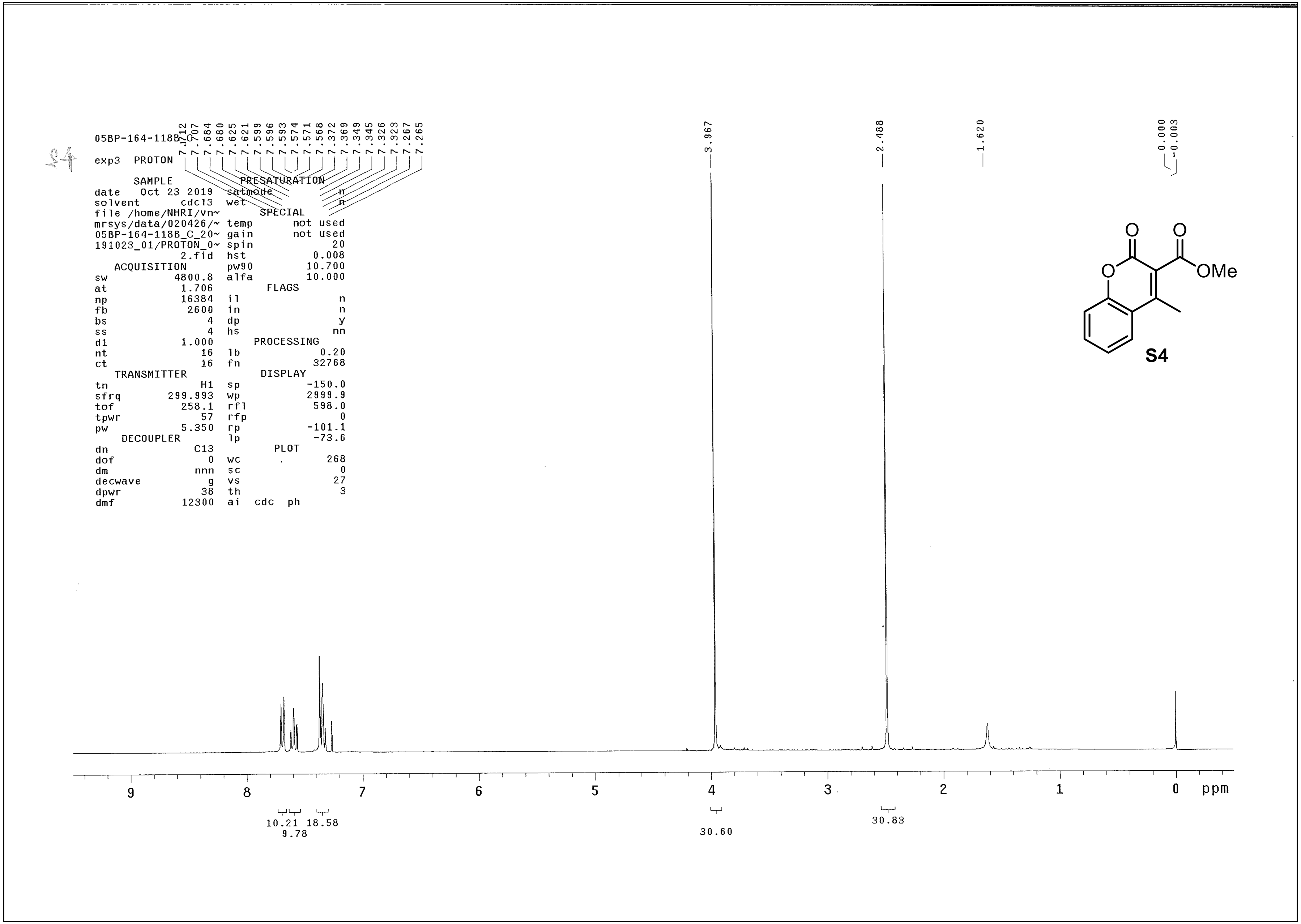

^{1}H NMR spectra for compound **S4**

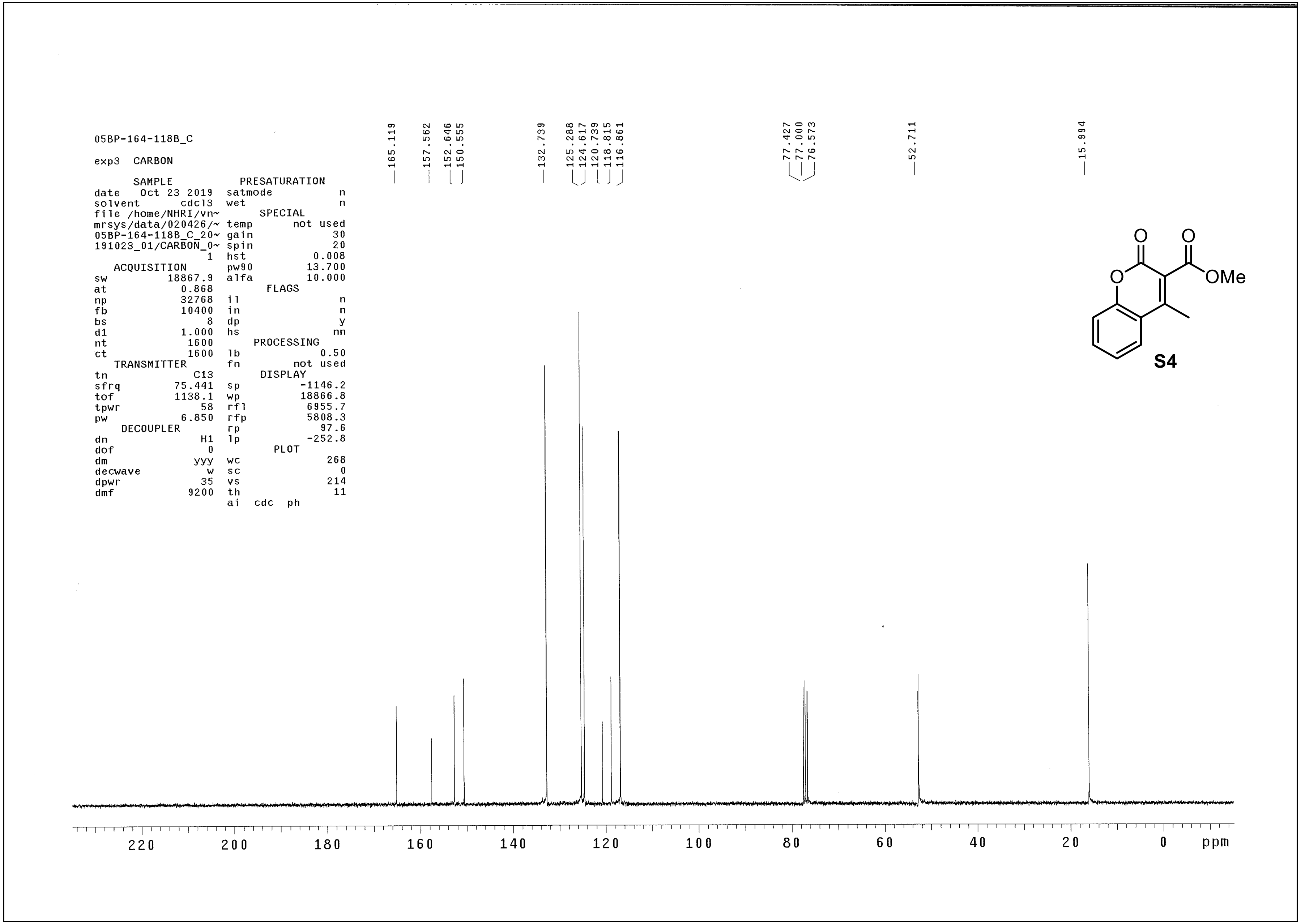

^{13}C NMR spectra for compound **S4**

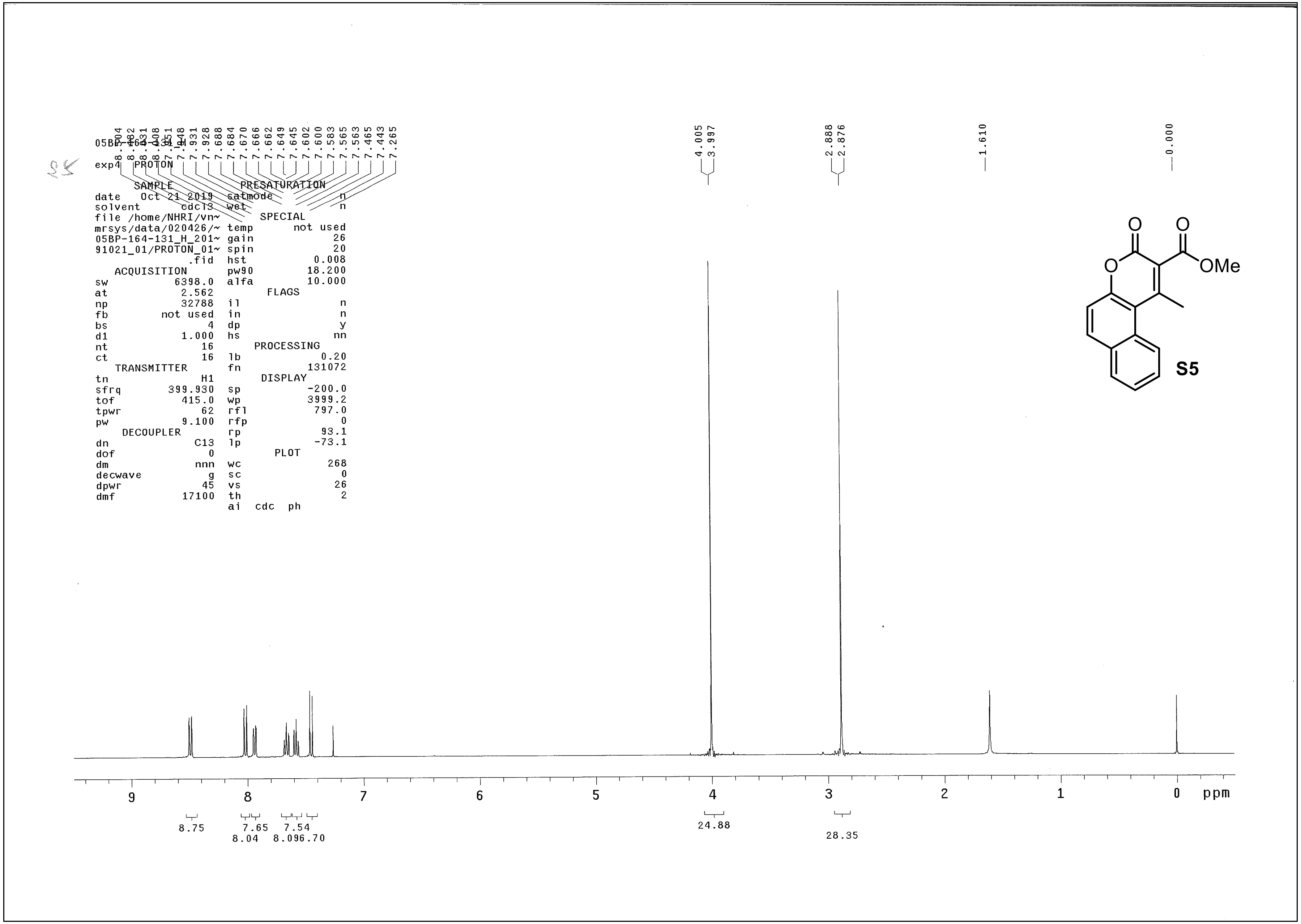

^{1}H NMR spectra for compound **S5**

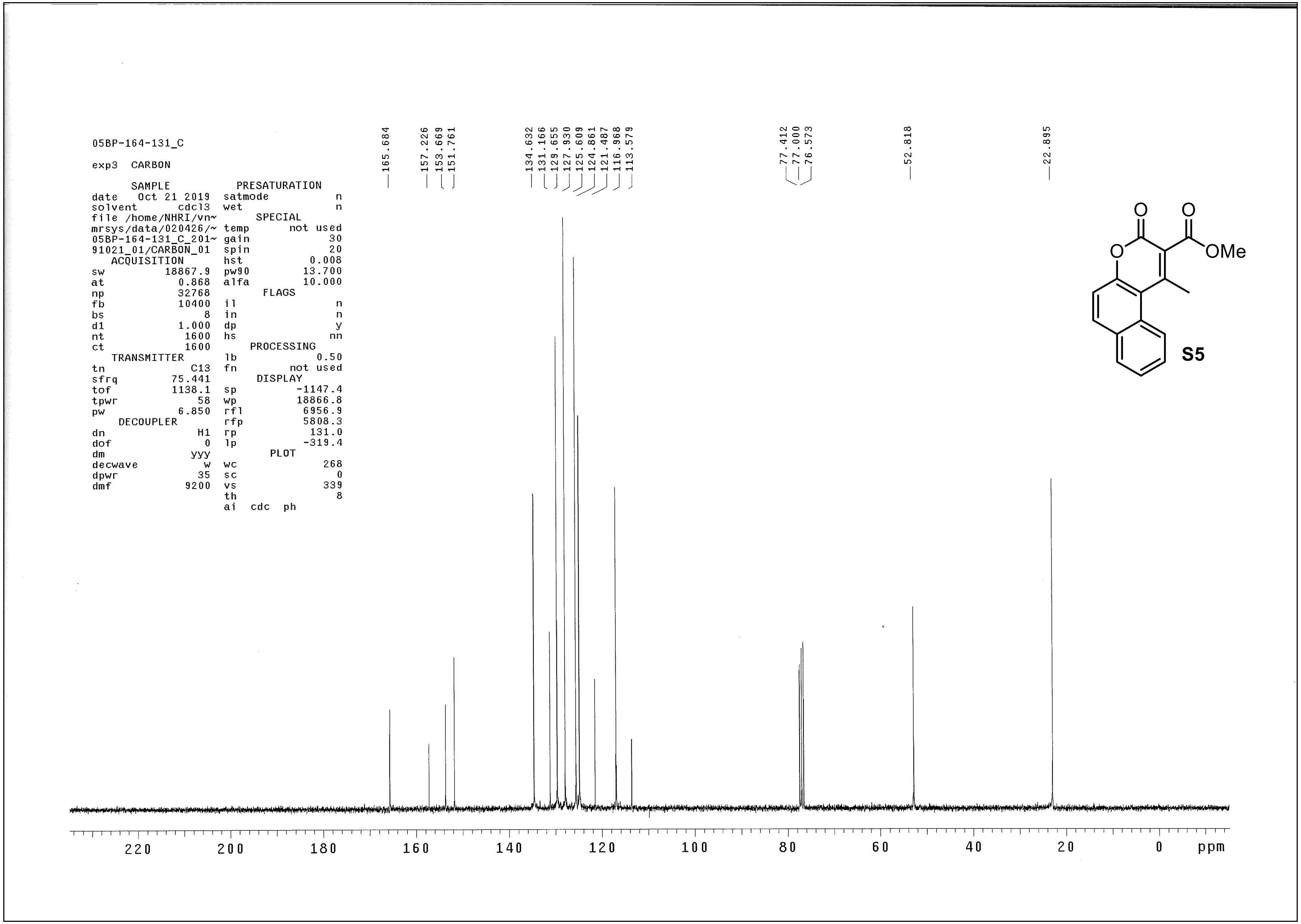

^{13}C NMR spectra for compound **S5**

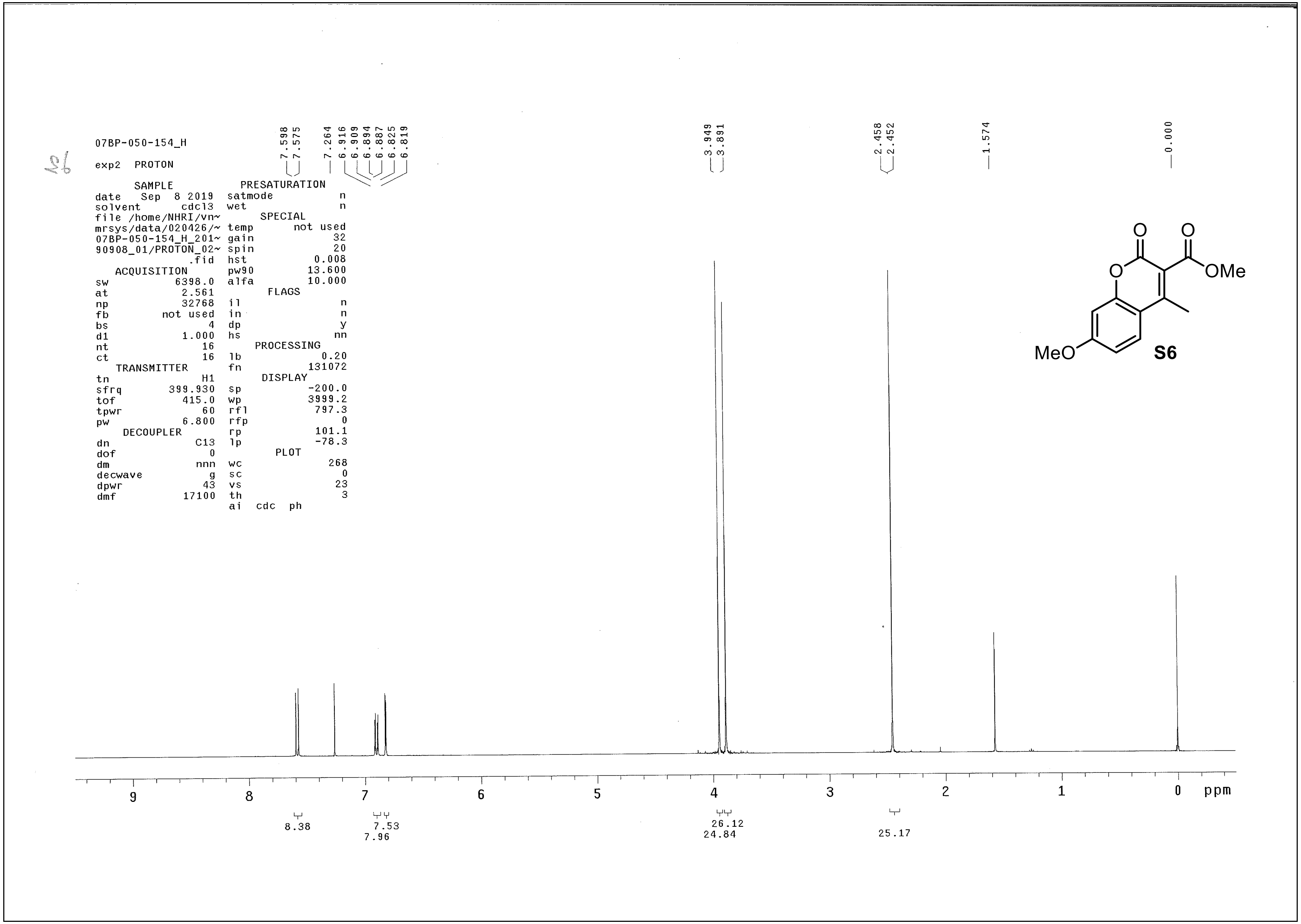

^{1}H NMR spectra for compound **S6**

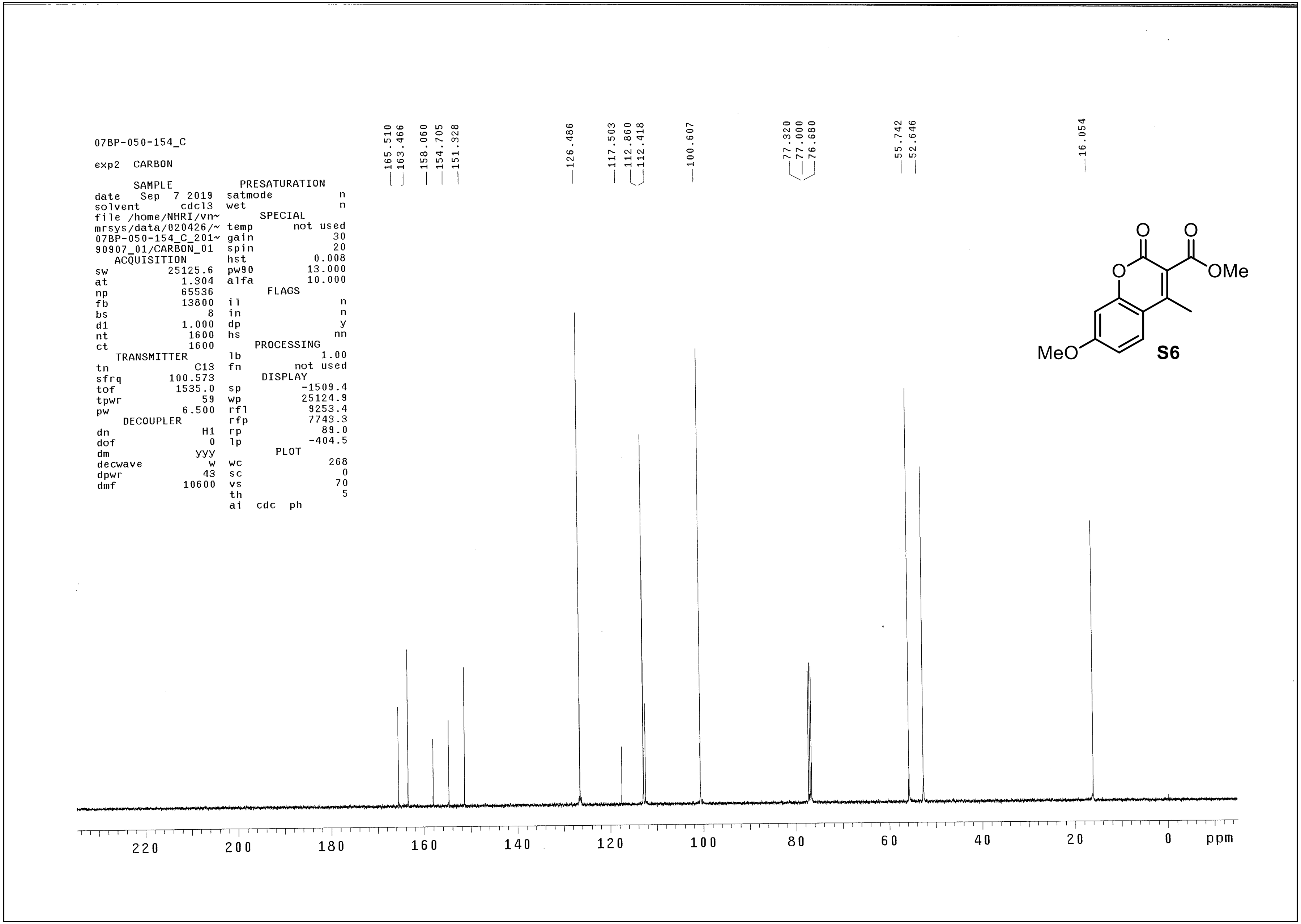

^{13}C NMR spectra for compound **S6**

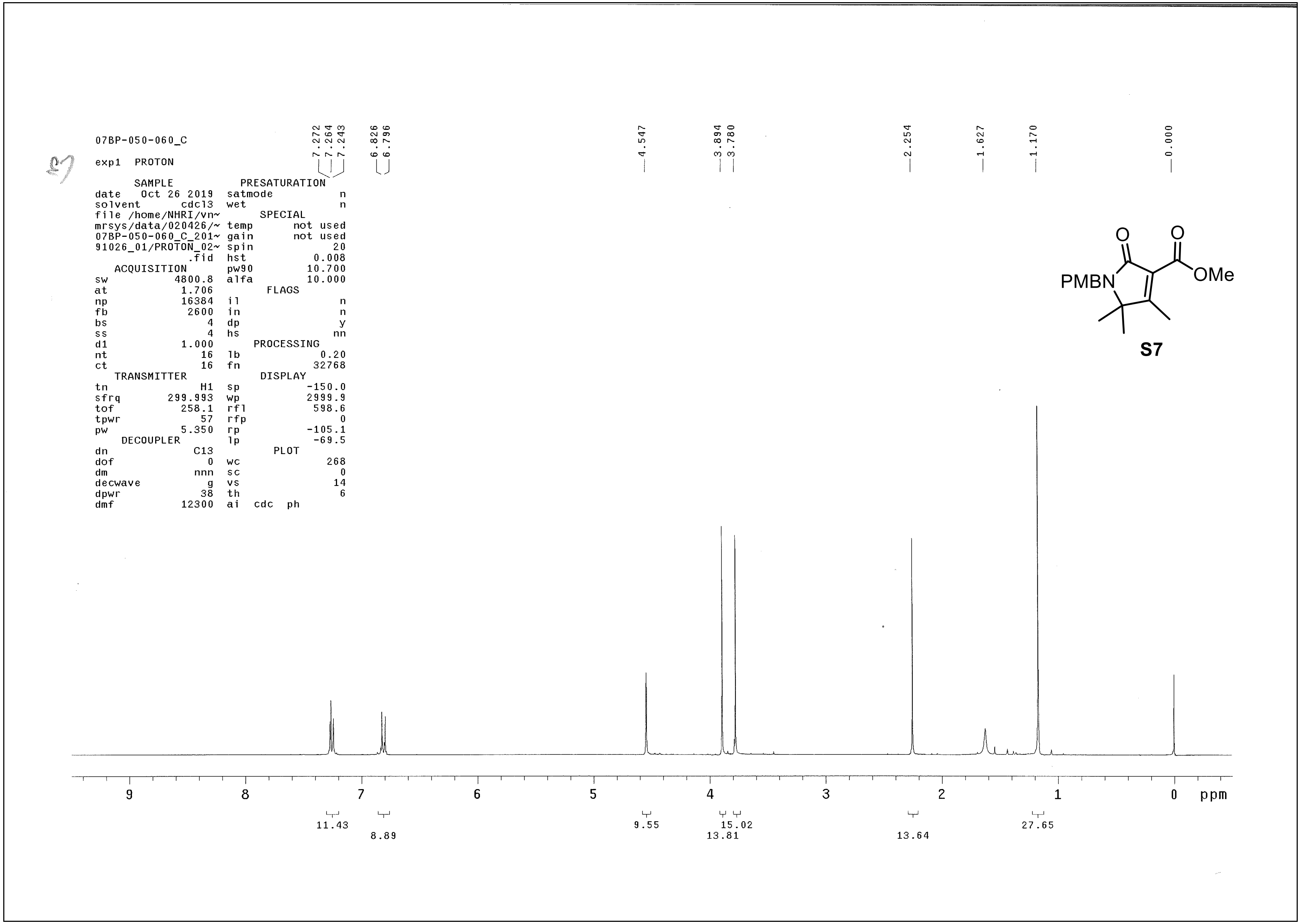

^{1}H NMR spectra for compound **S7**

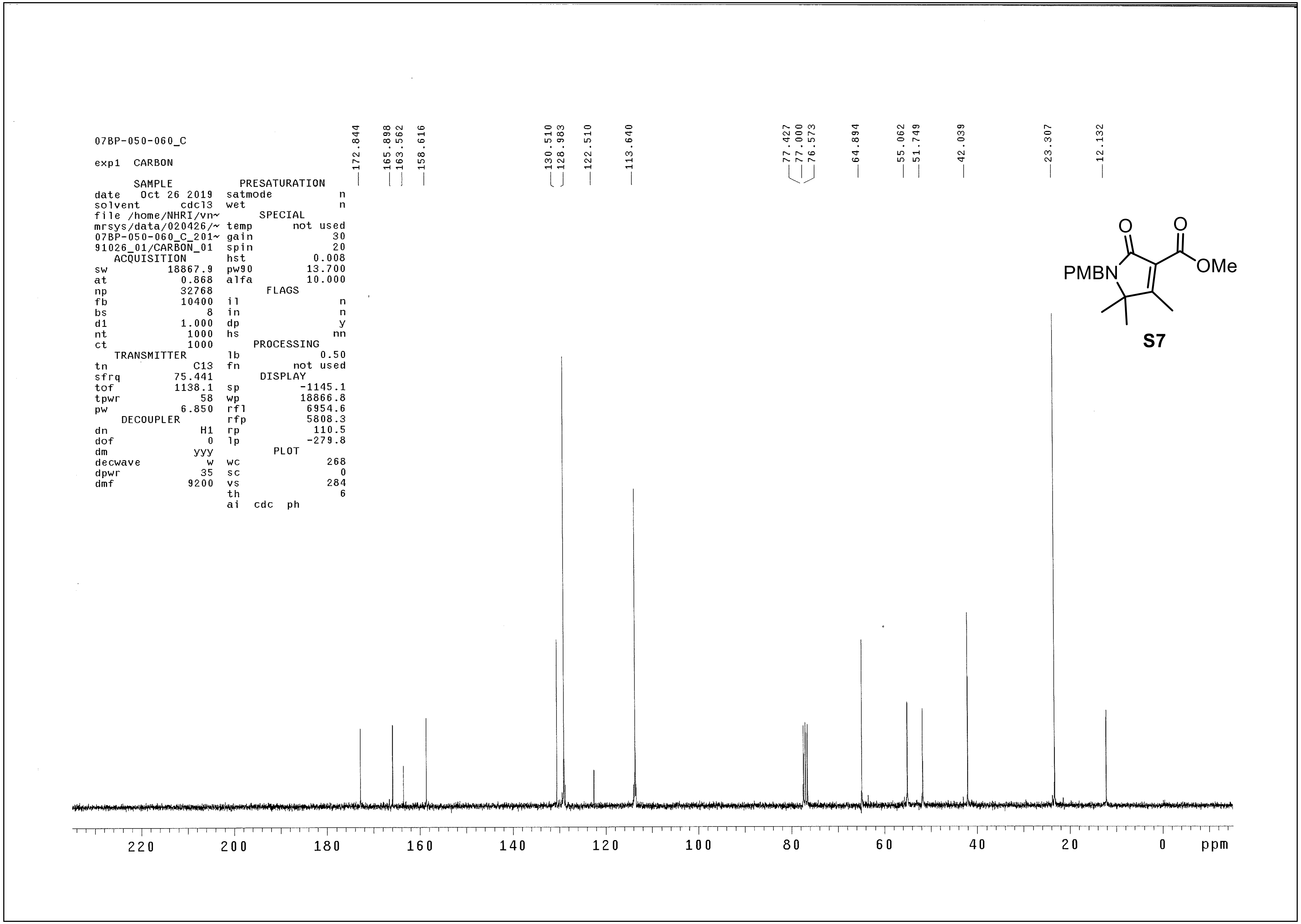

^{13}C NMR spectra for compound **S7**

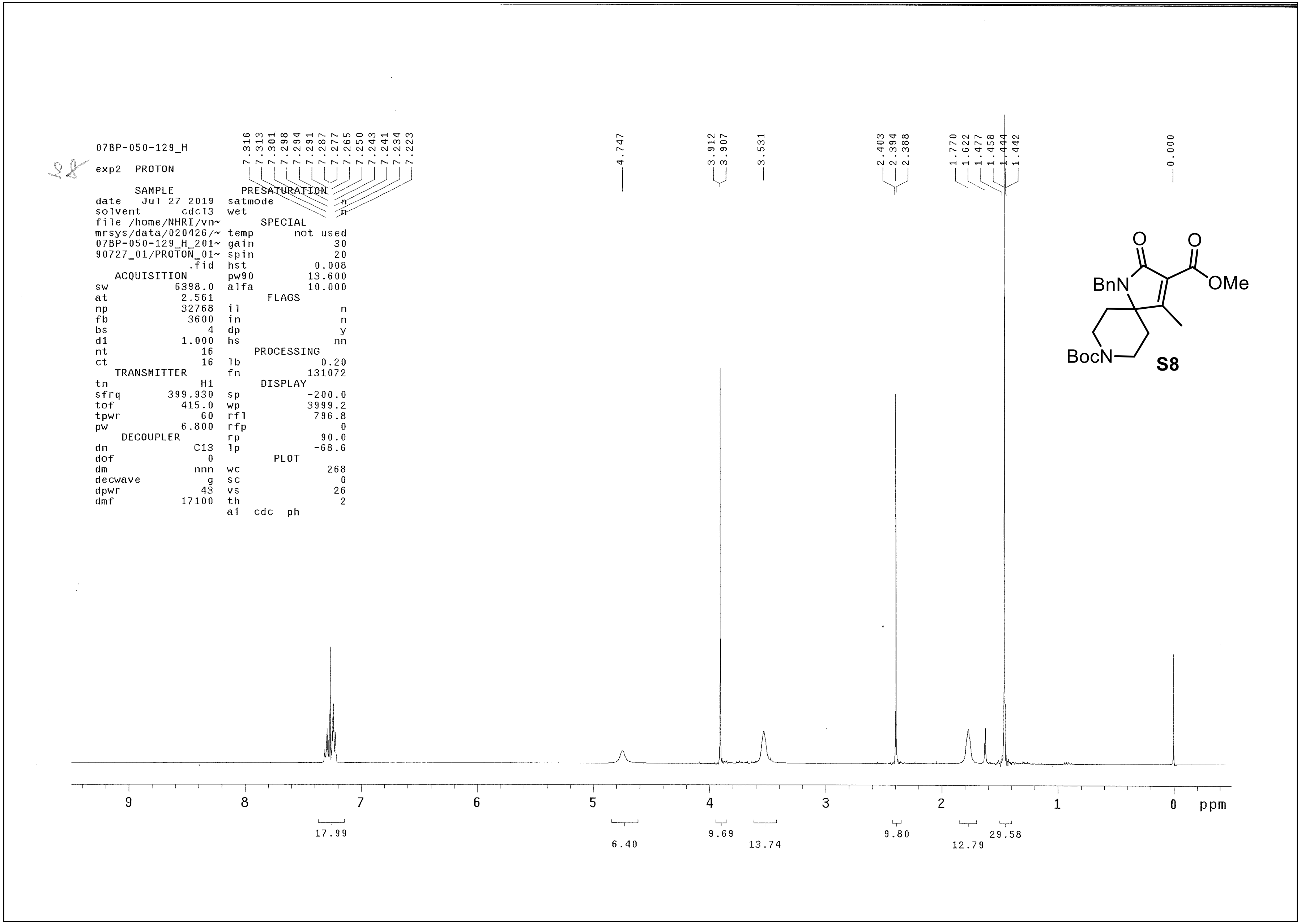

^{1}H NMR spectra for compound **S8**

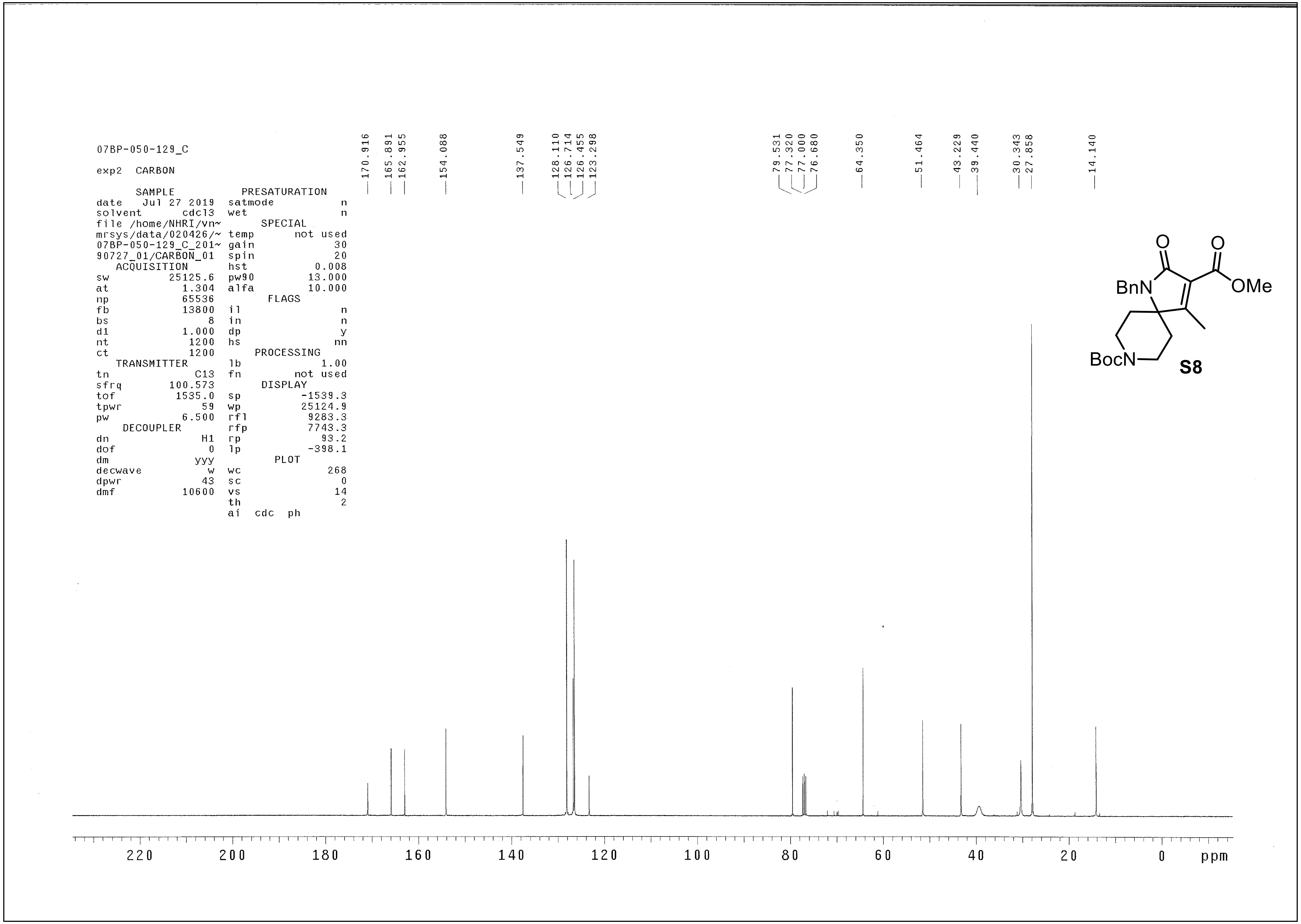

^{13}C NMR spectra for compound **S8**

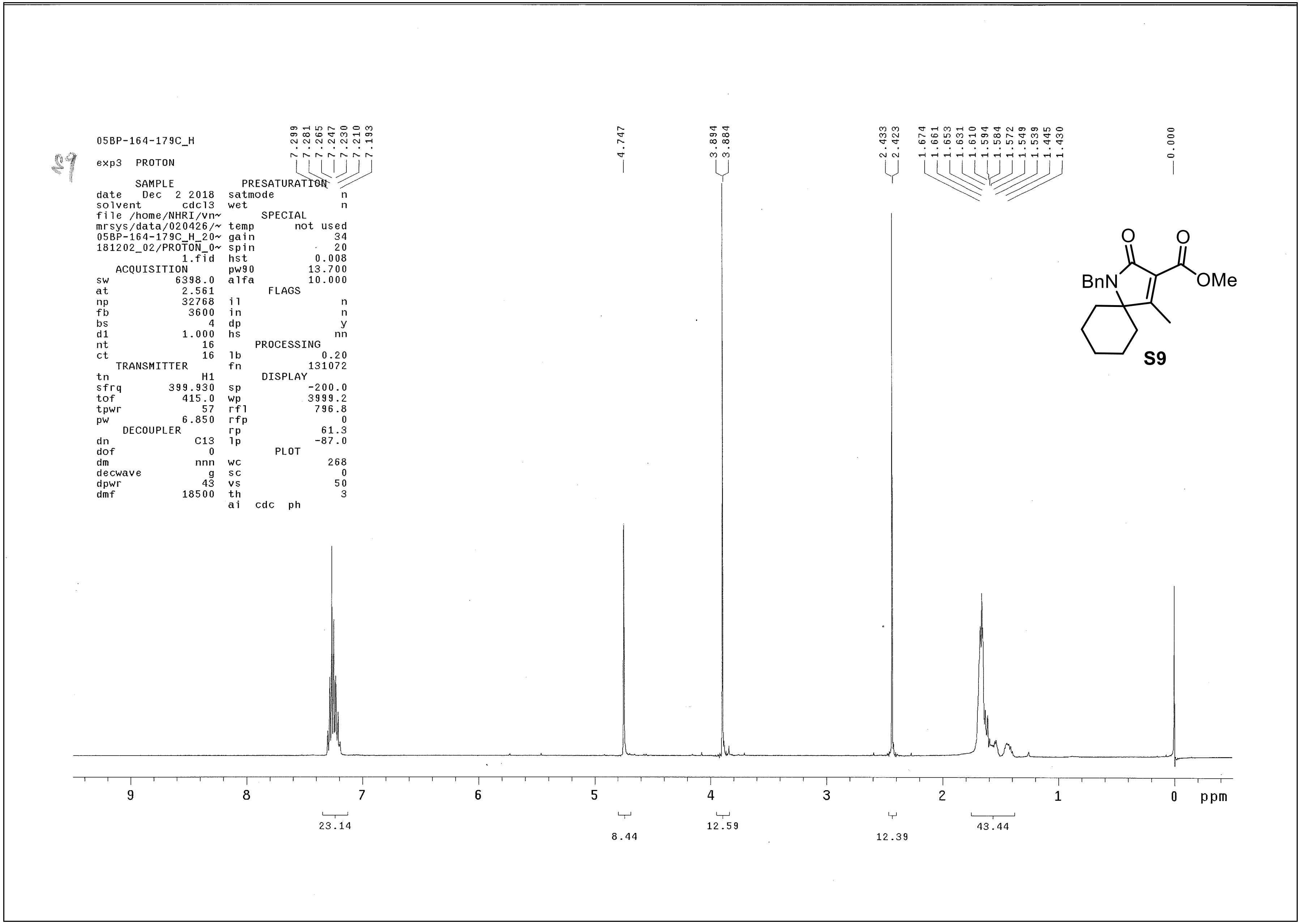

^{1}H NMR spectra for compound **S9**

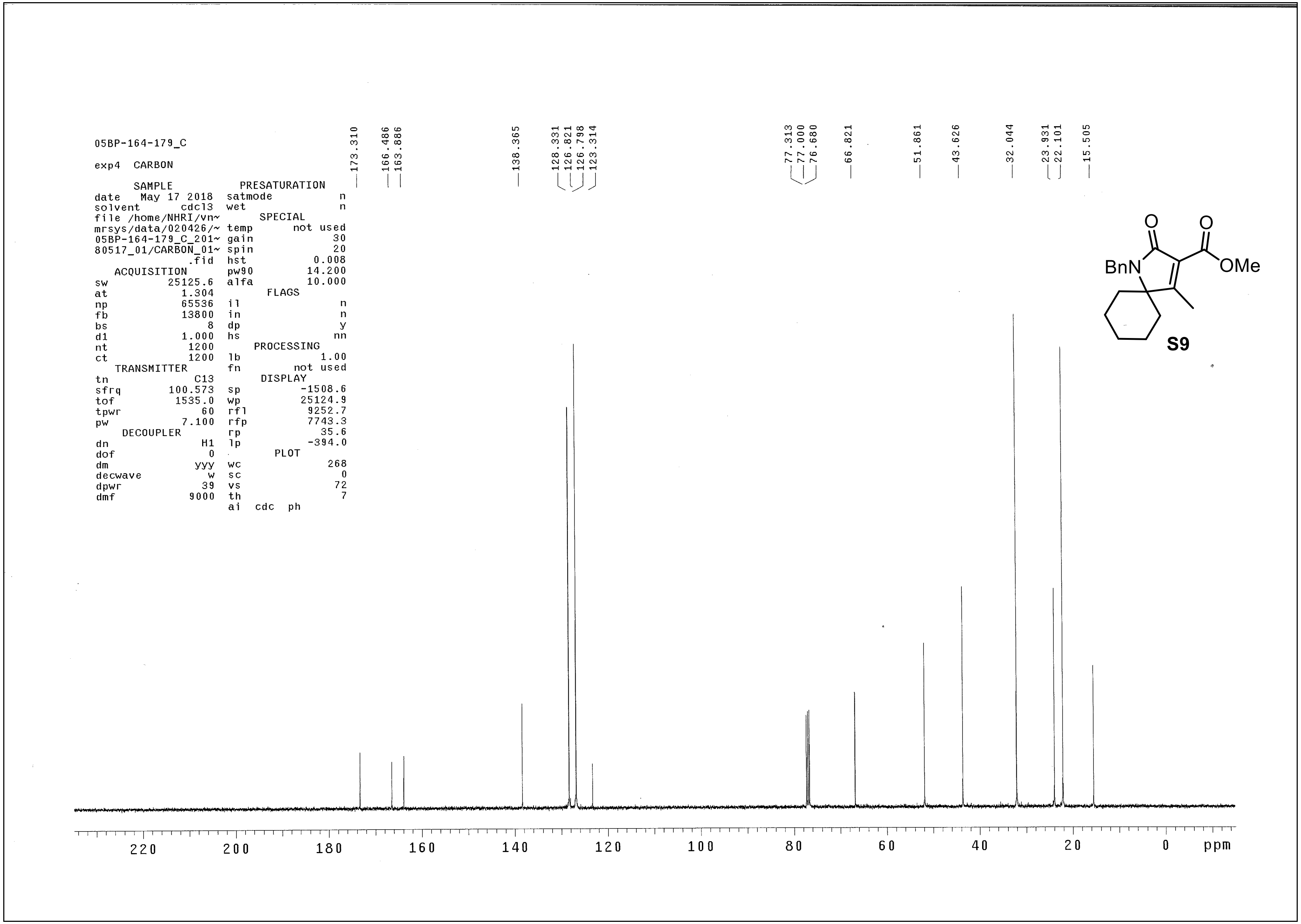

^{13}C NMR spectra for compound **S9**

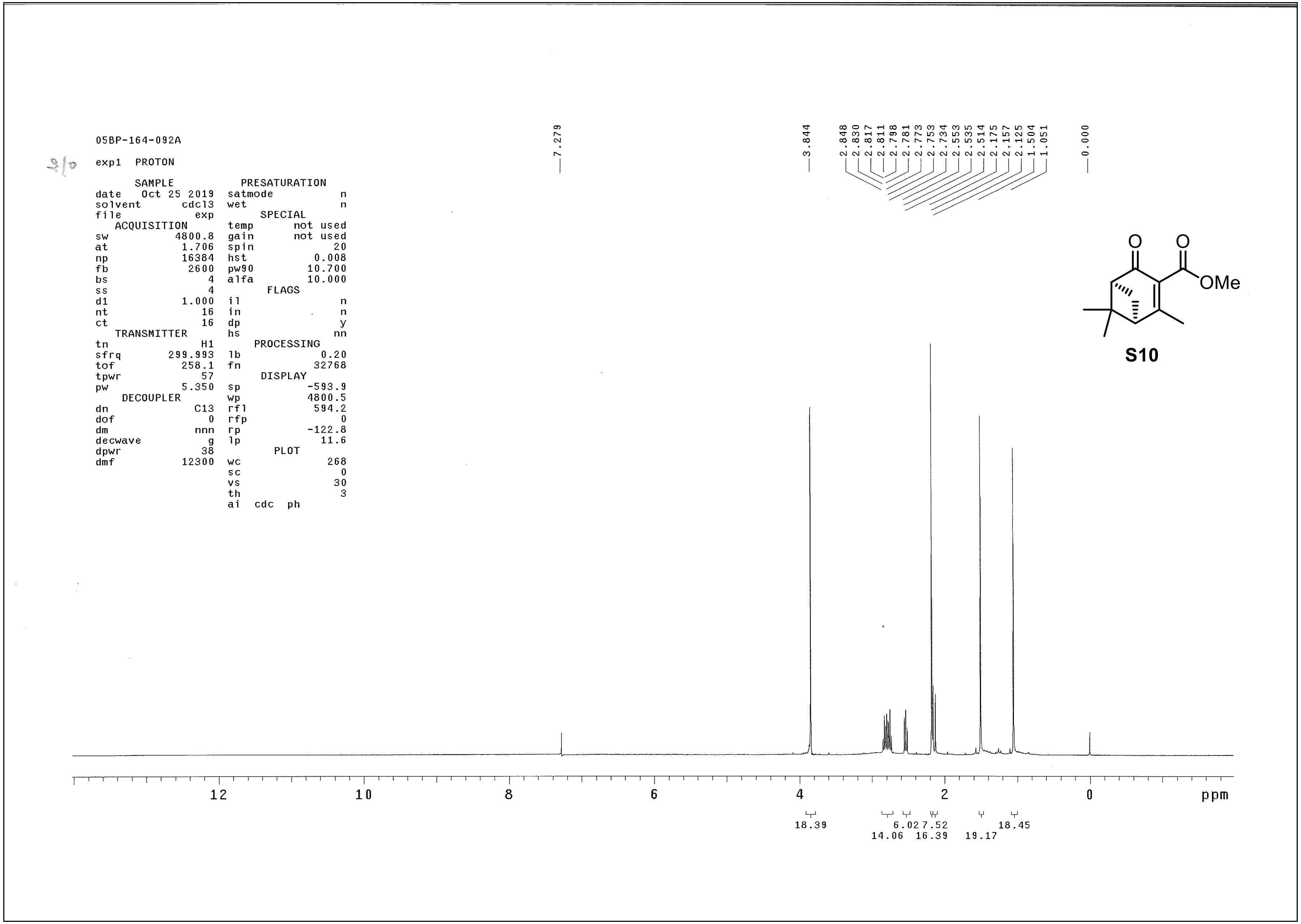

^{1}H NMR spectra for compound **S10**

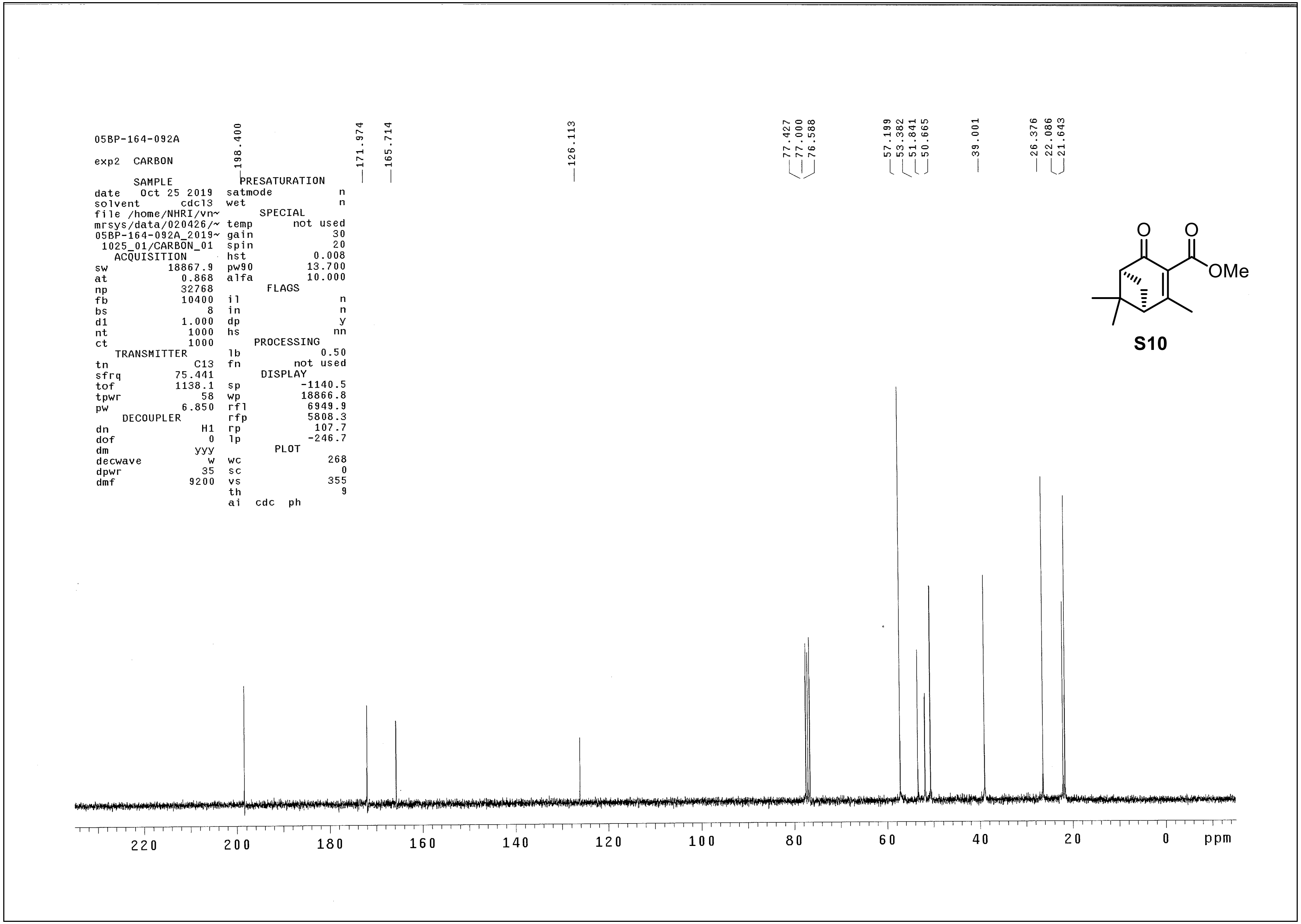

^{13}C NMR spectra for compound **S10**

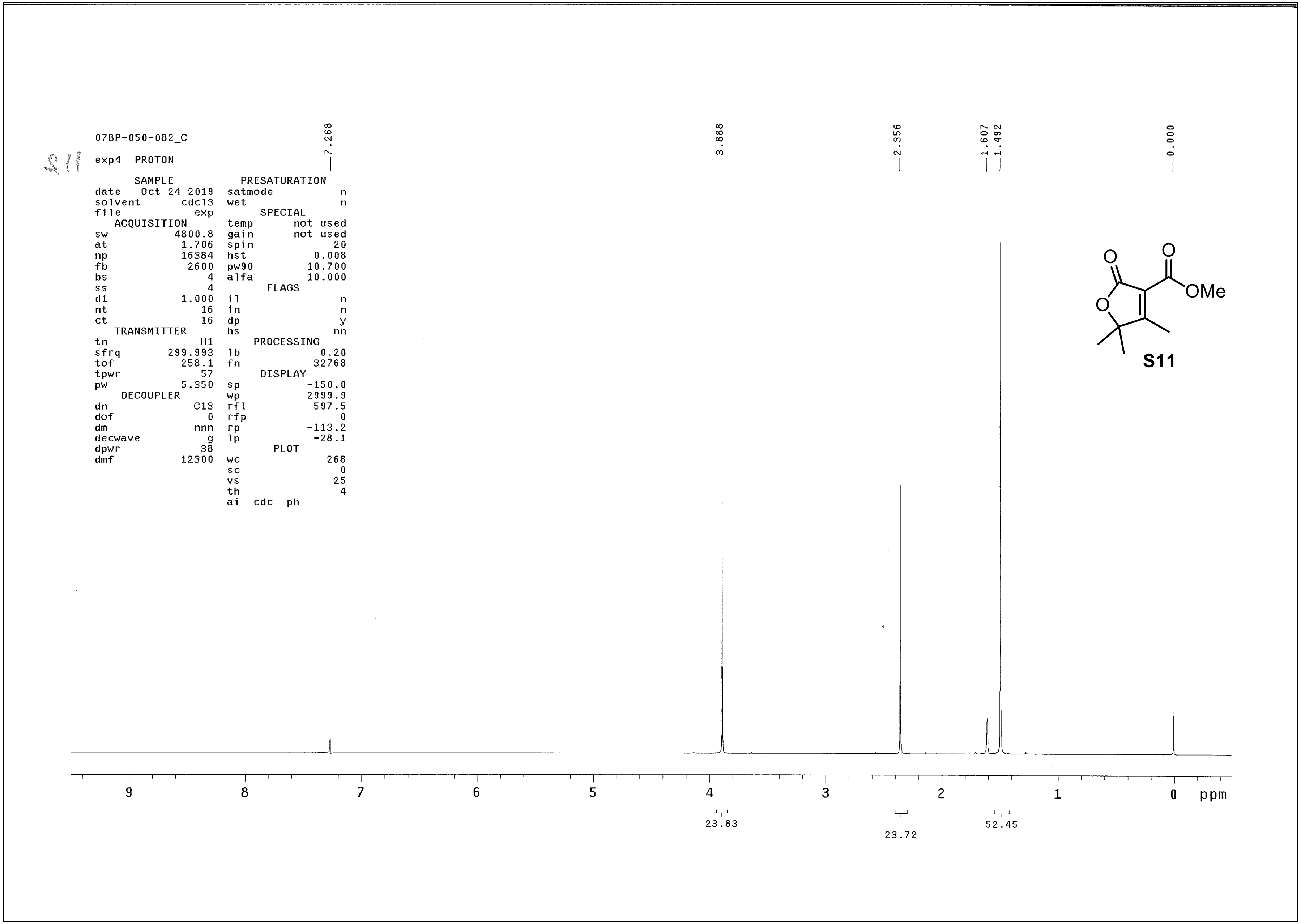

^{1}H NMR spectra for compound **S11**

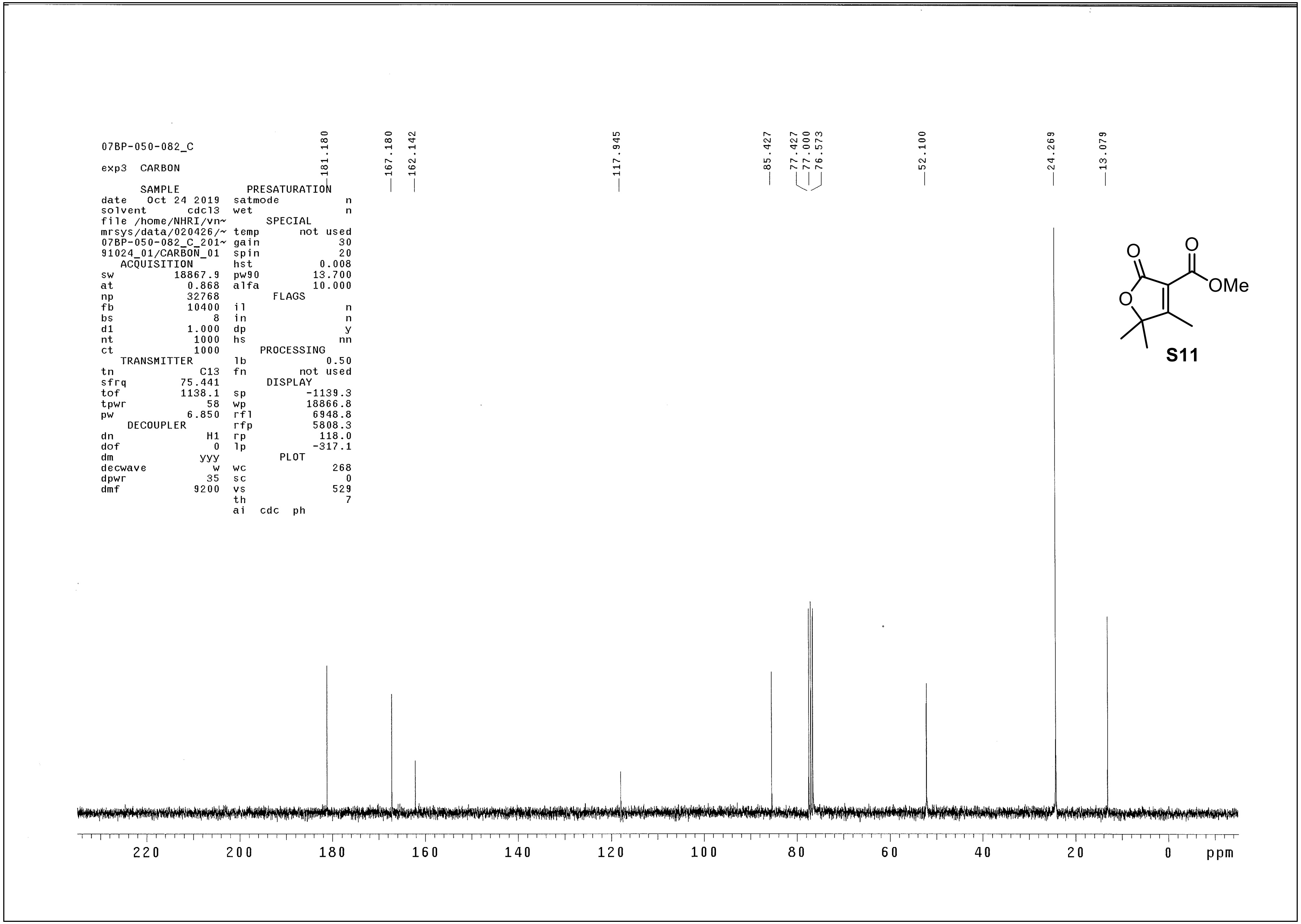

^{13}C NMR spectra for compound **S11**

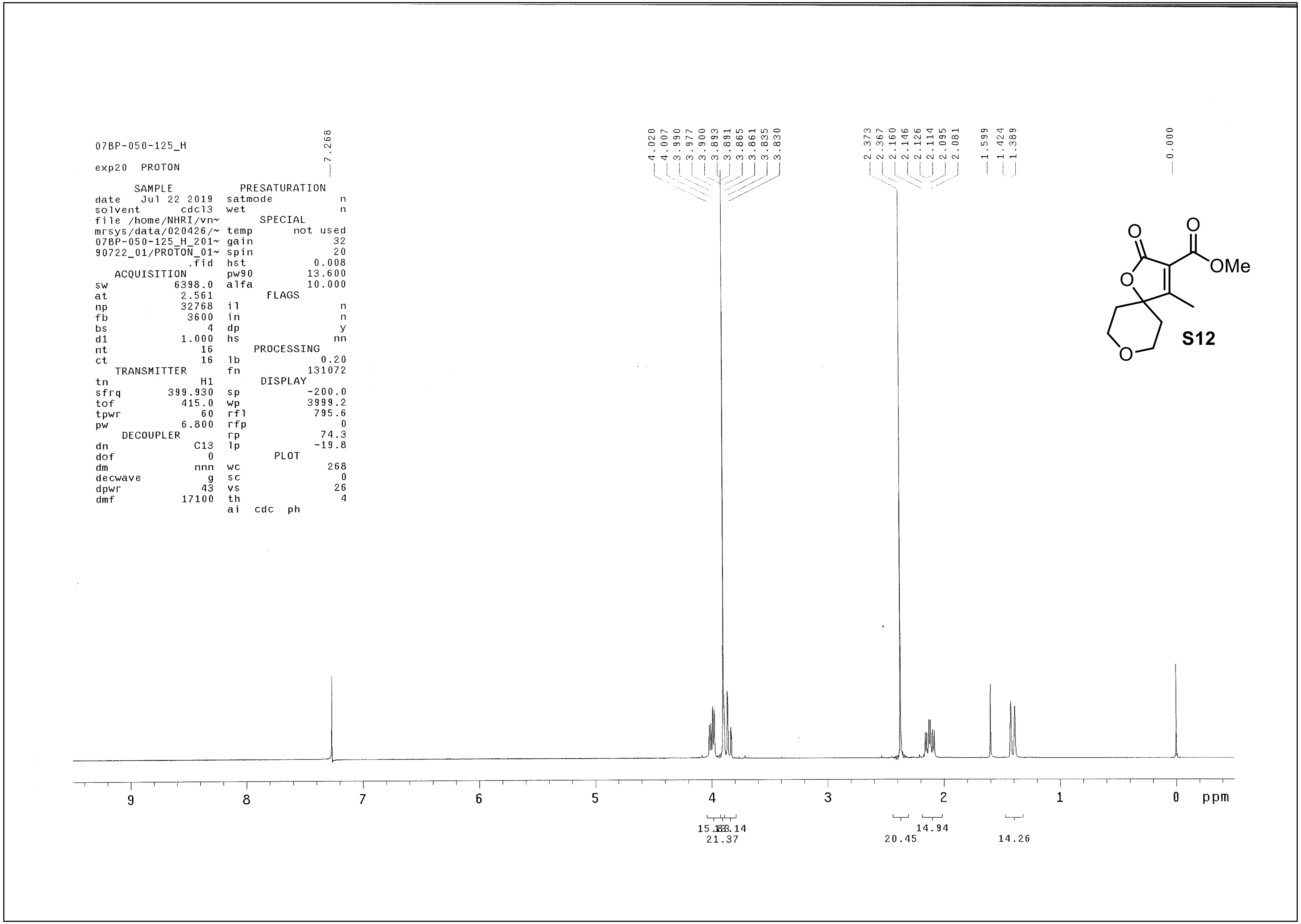

^{1}H NMR spectra for compound **S12**

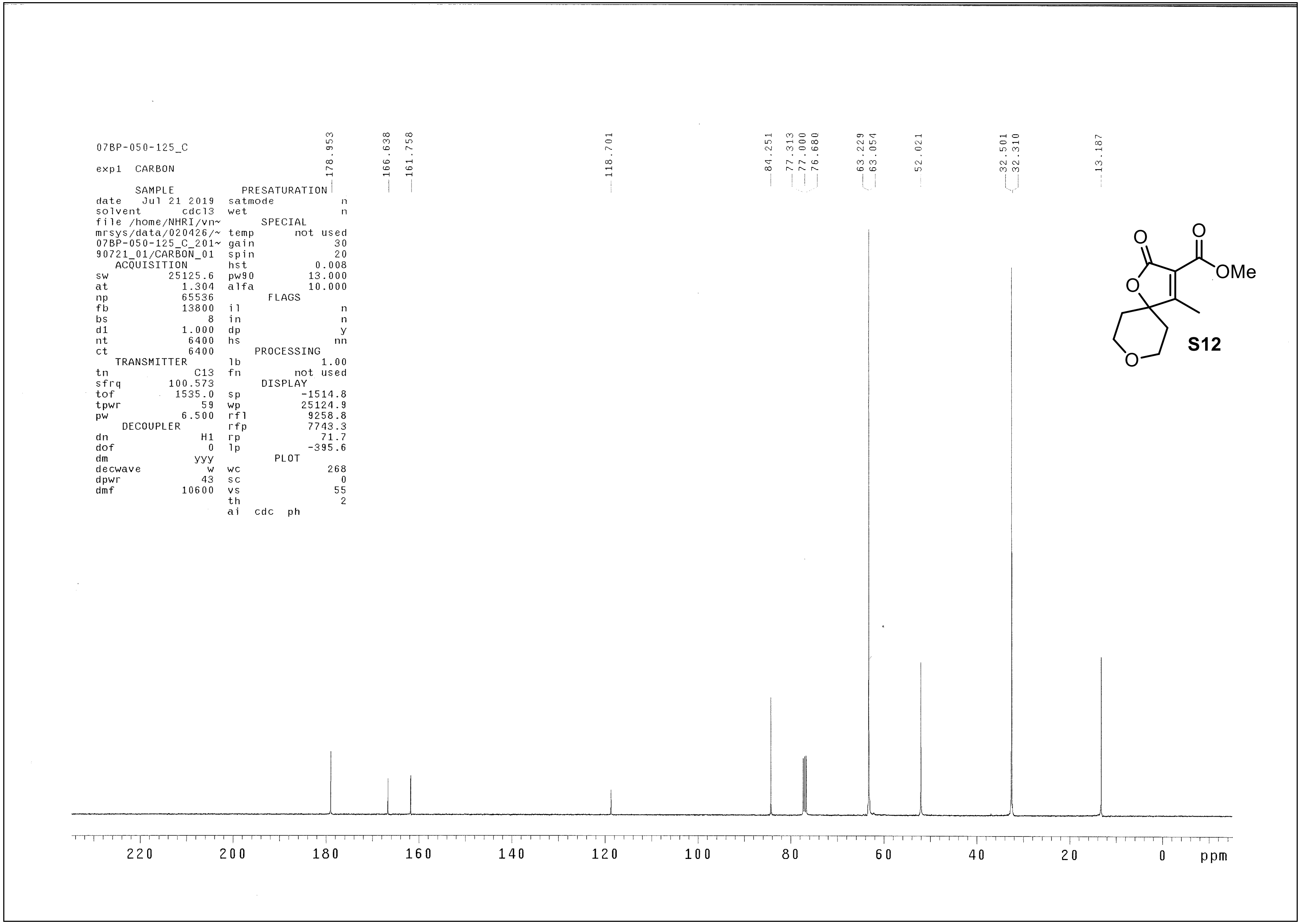

^{13}C NMR spectra for compound **S12**

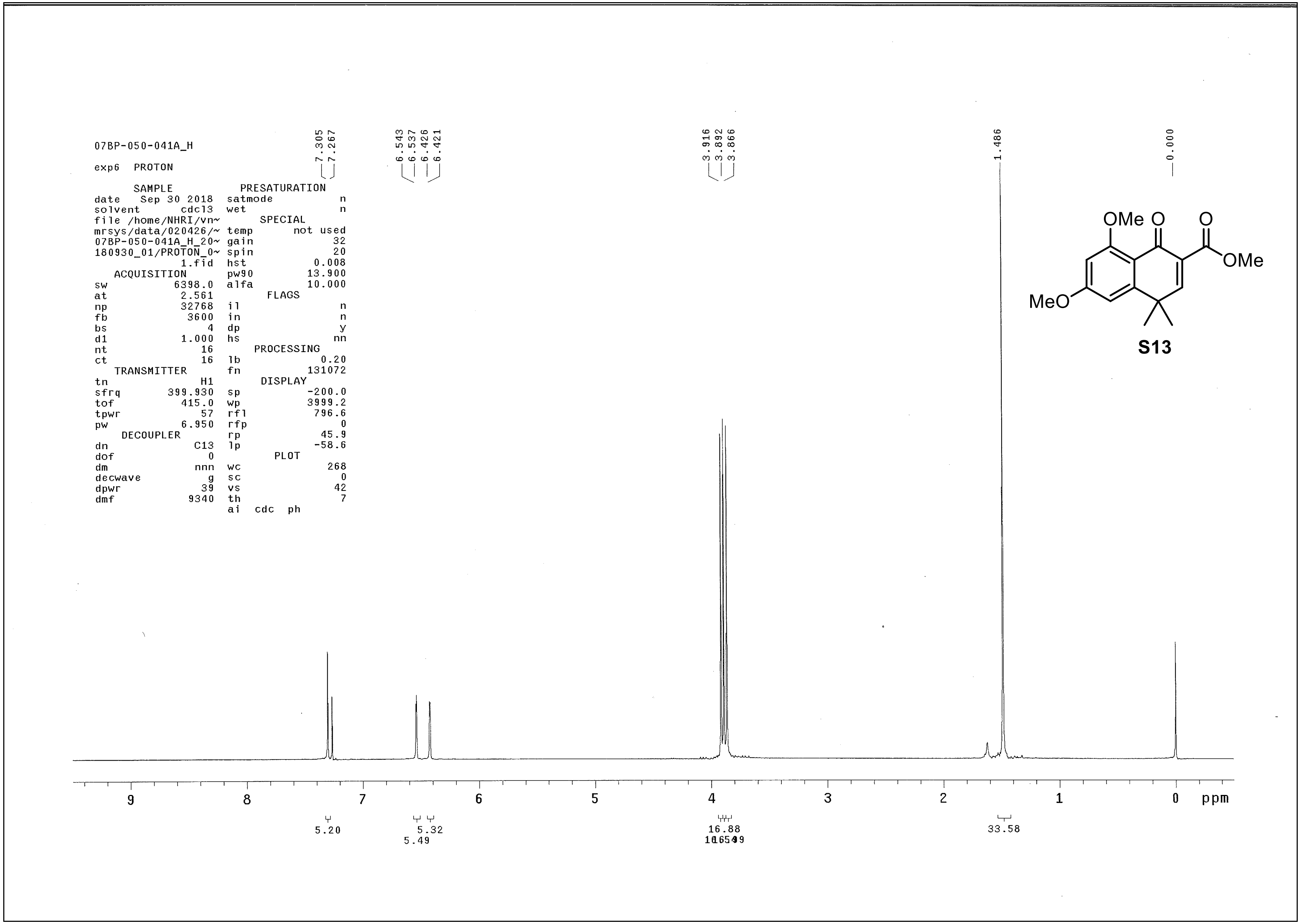

^{1}H NMR spectra for compoun**d S13**

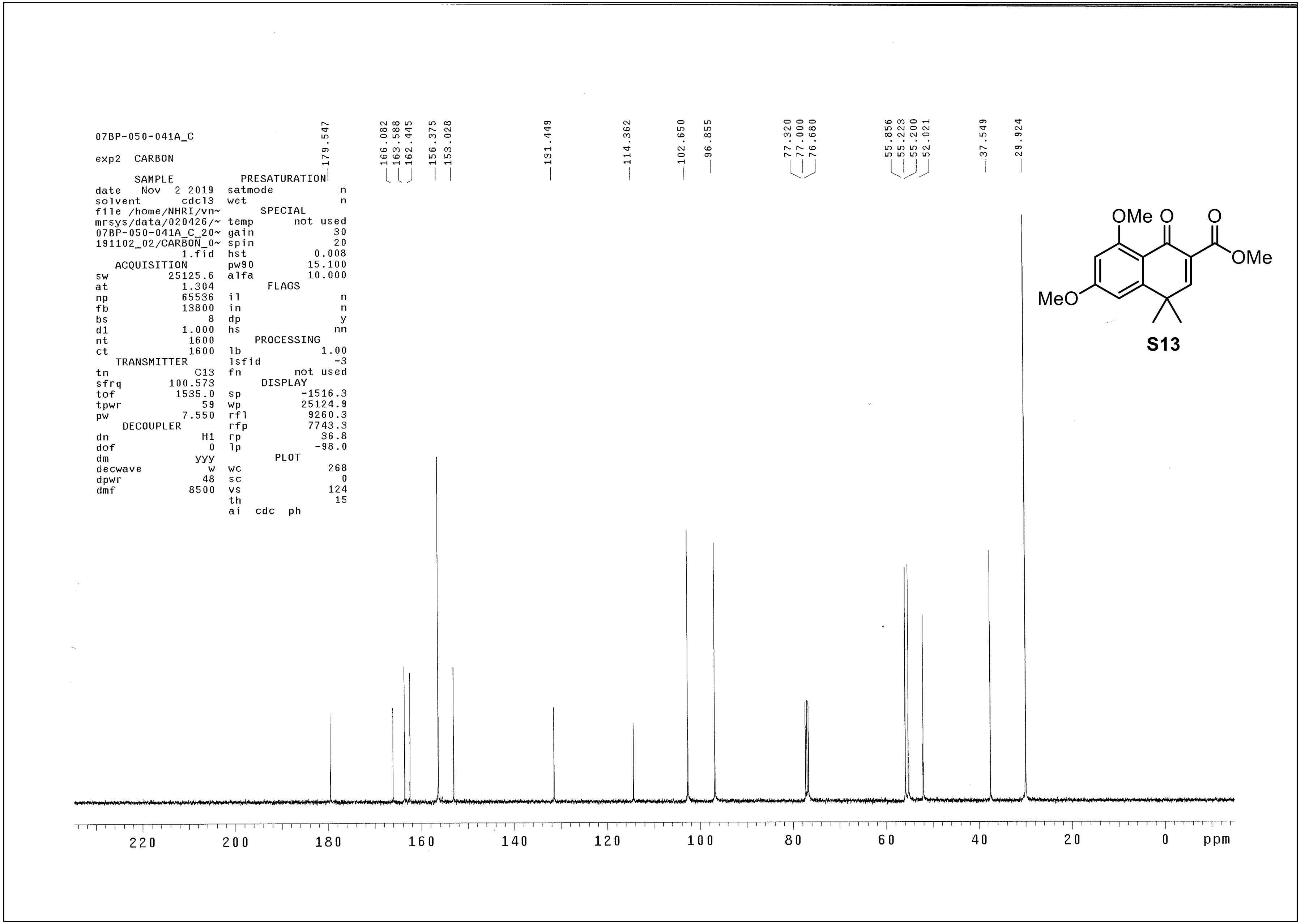

^{13}C NMR spectra for compound **S13**

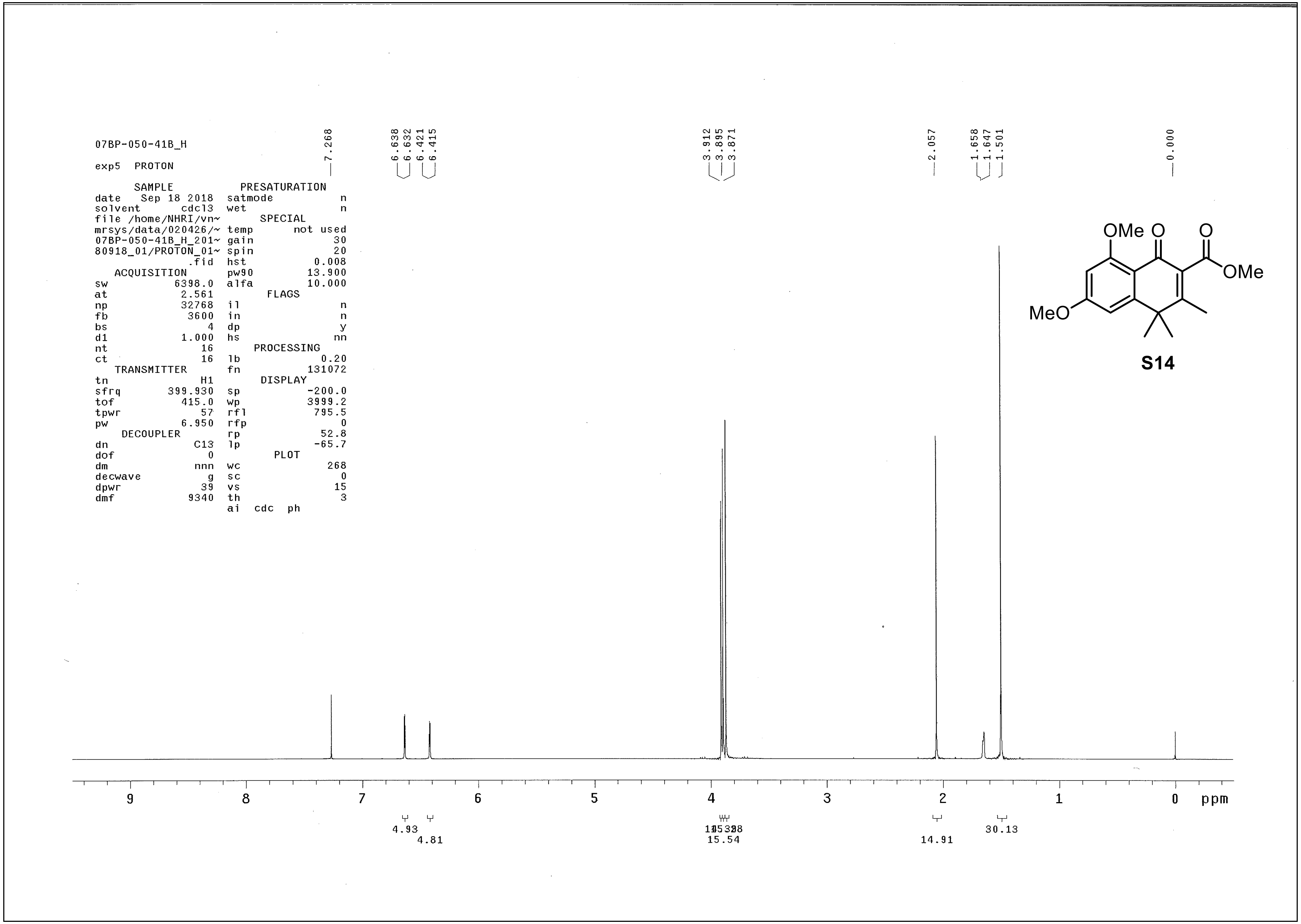

^{1}H NMR spectra for compound **S14**

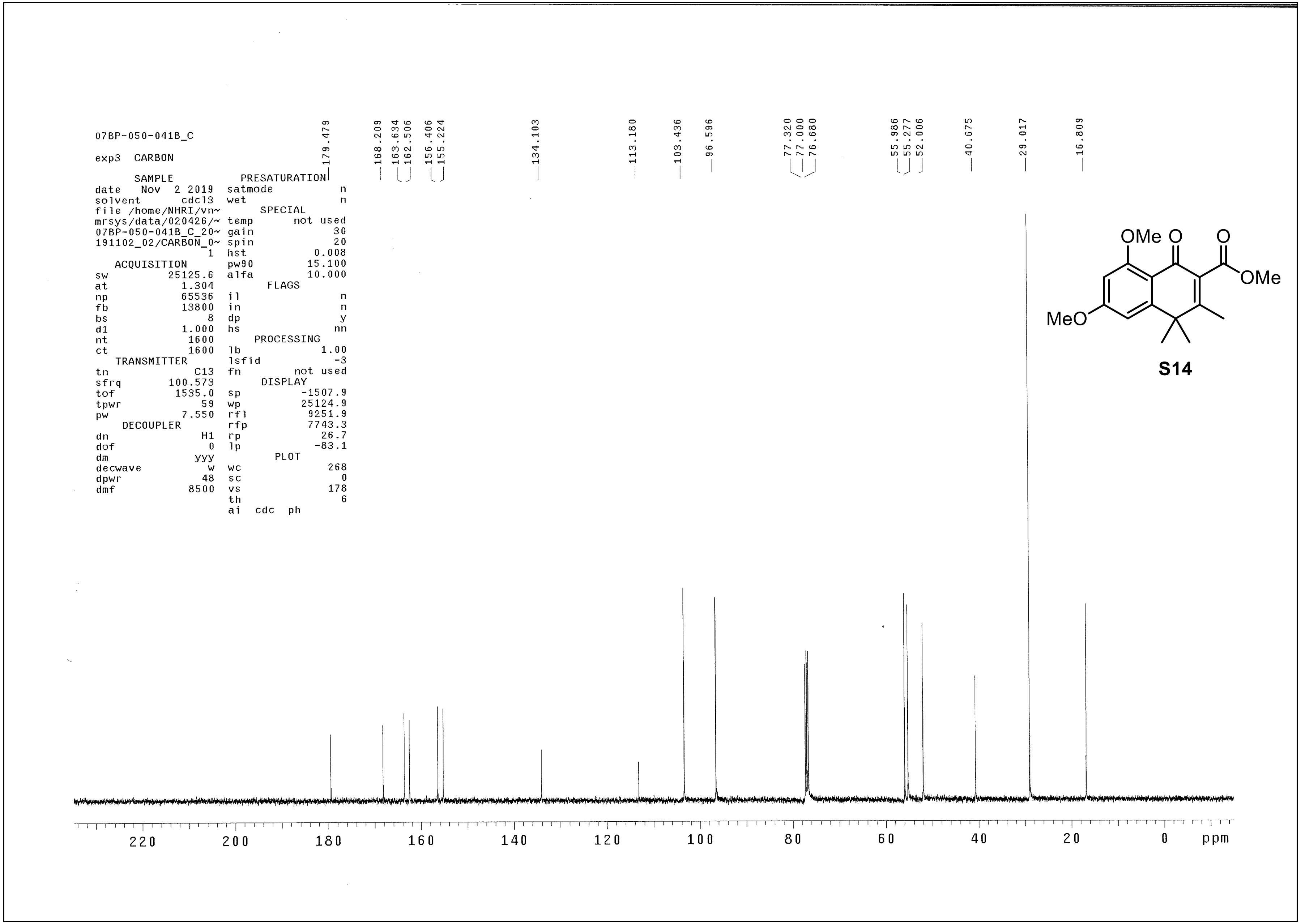

^{13}C NMR spectra for compound **S14**

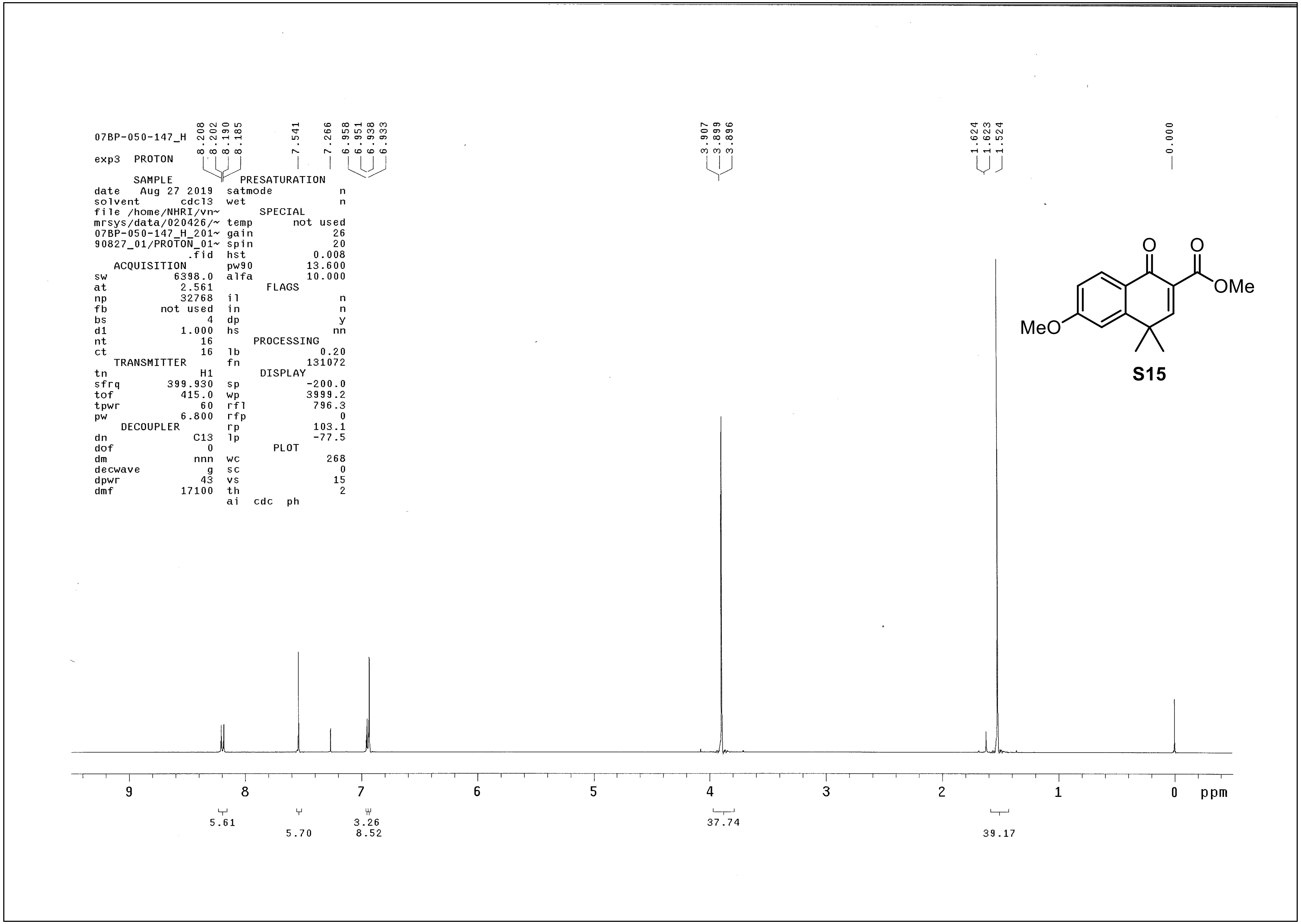

^{1}H NMR spectra for compound **S15**

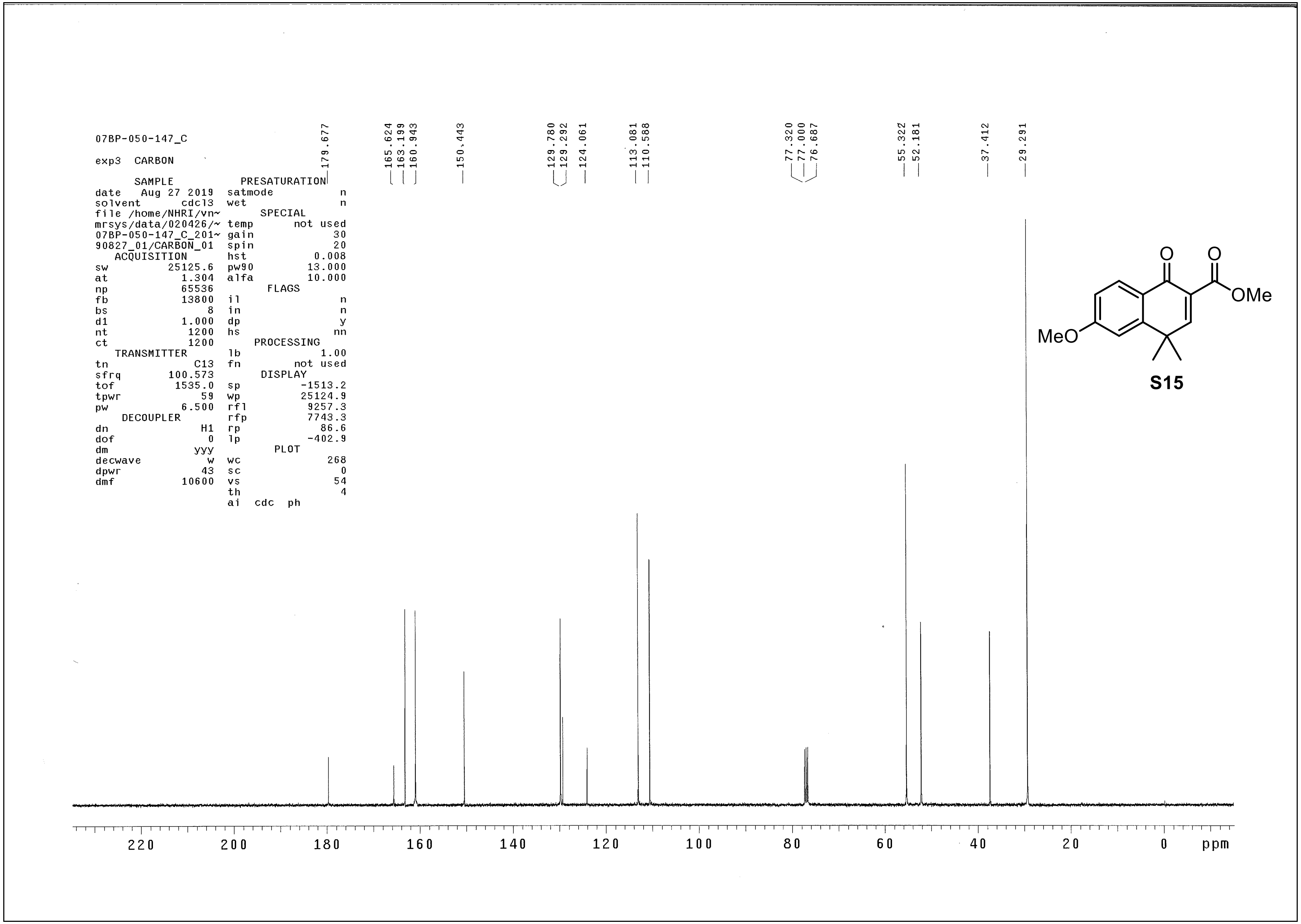

^{13}C NMR spectra for compound **S15**

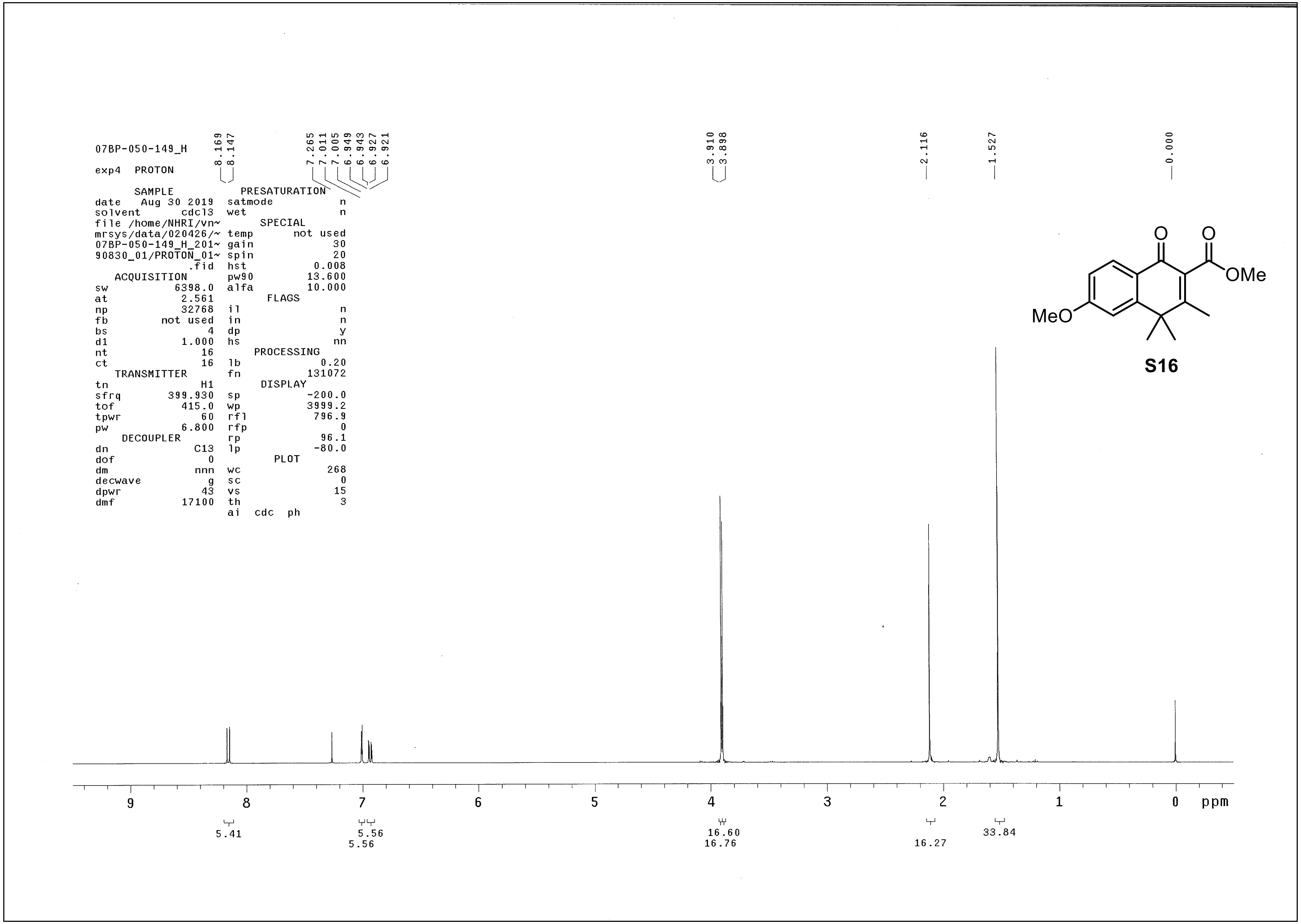

^{1}H NMR spectra for compound **S16**

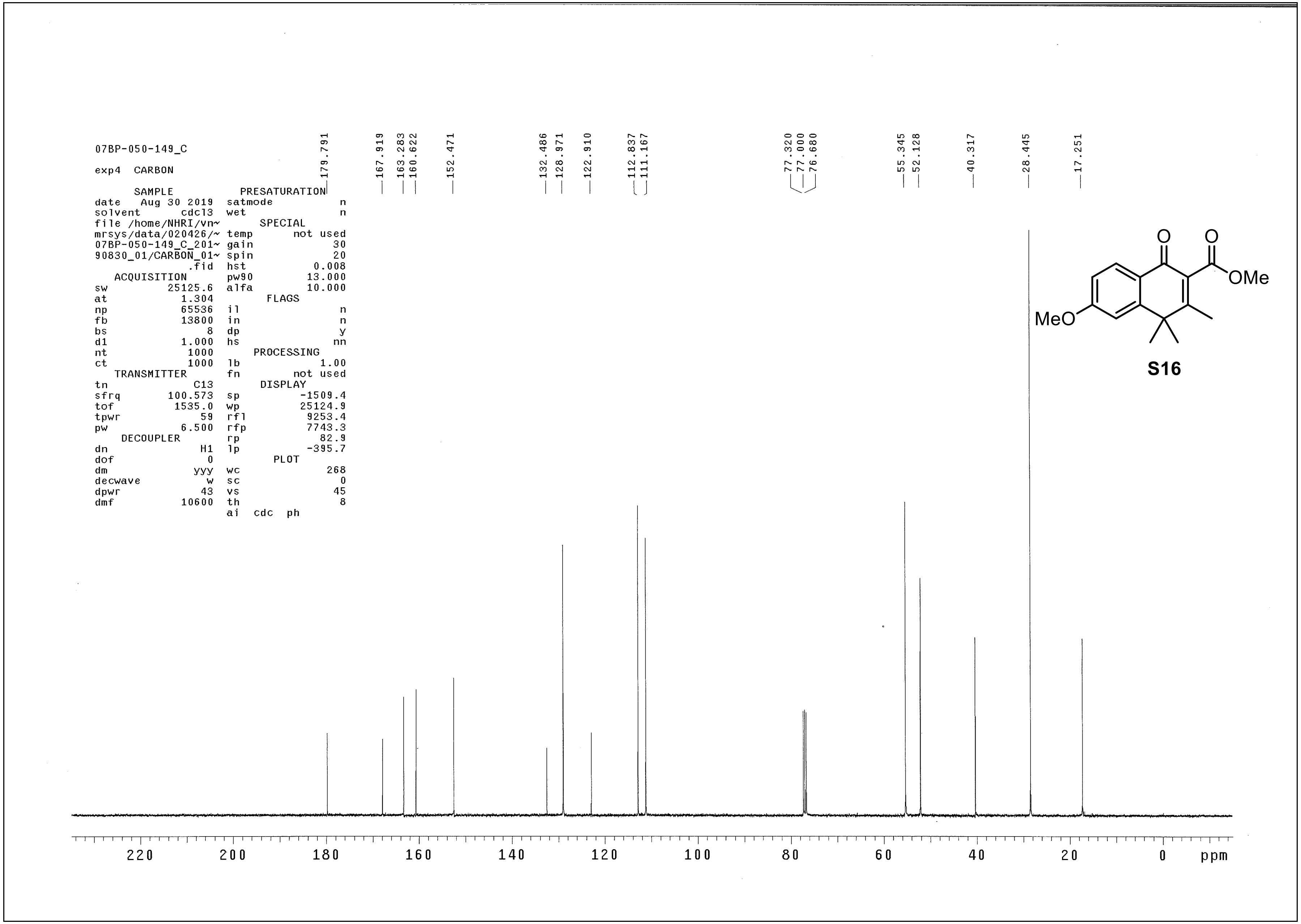

^{13}C NMR spectra for compound **S16**

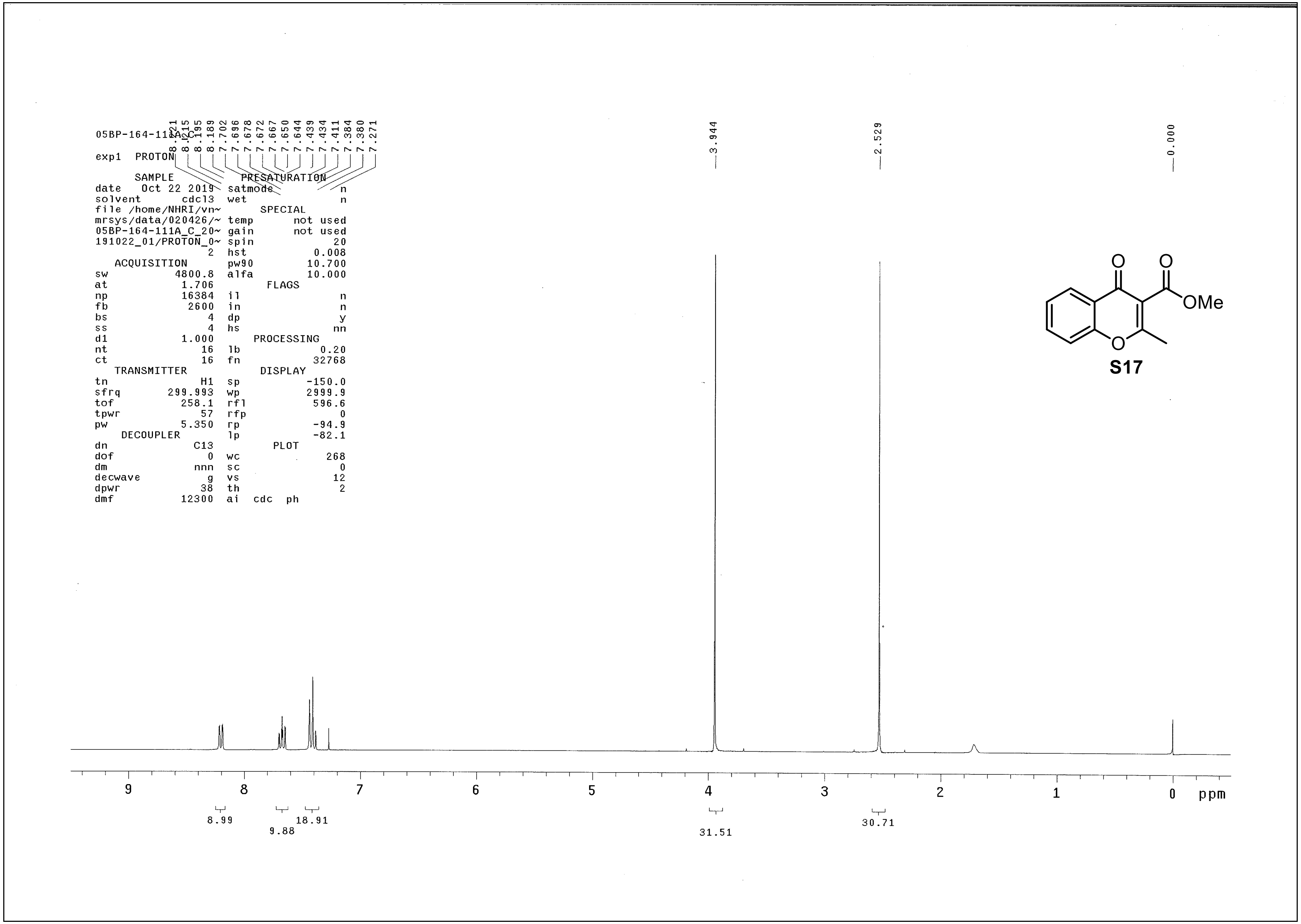

^{1}H NMR spectra for compound **S17**

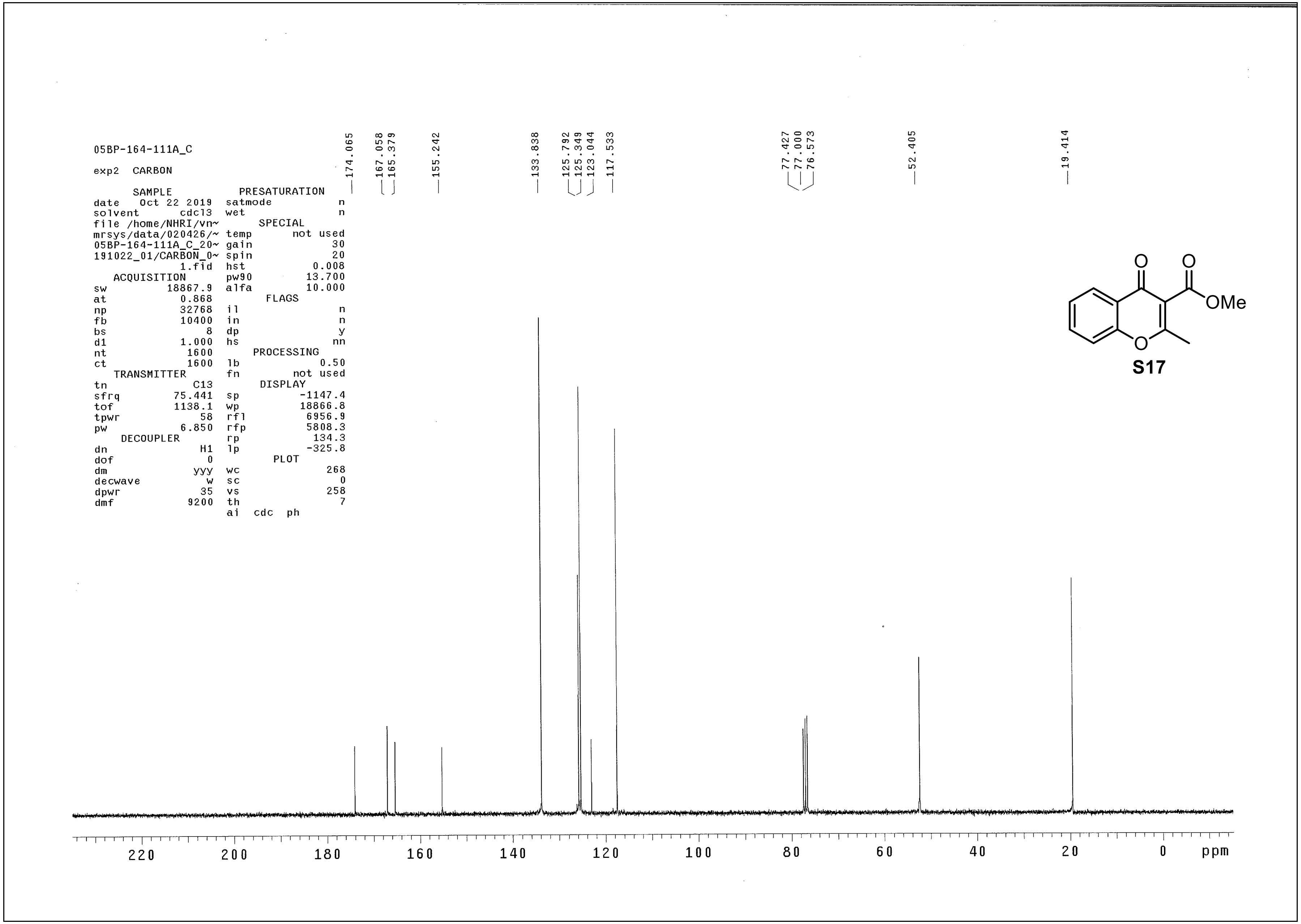

^{13}C NMR spectra for compound **S17**

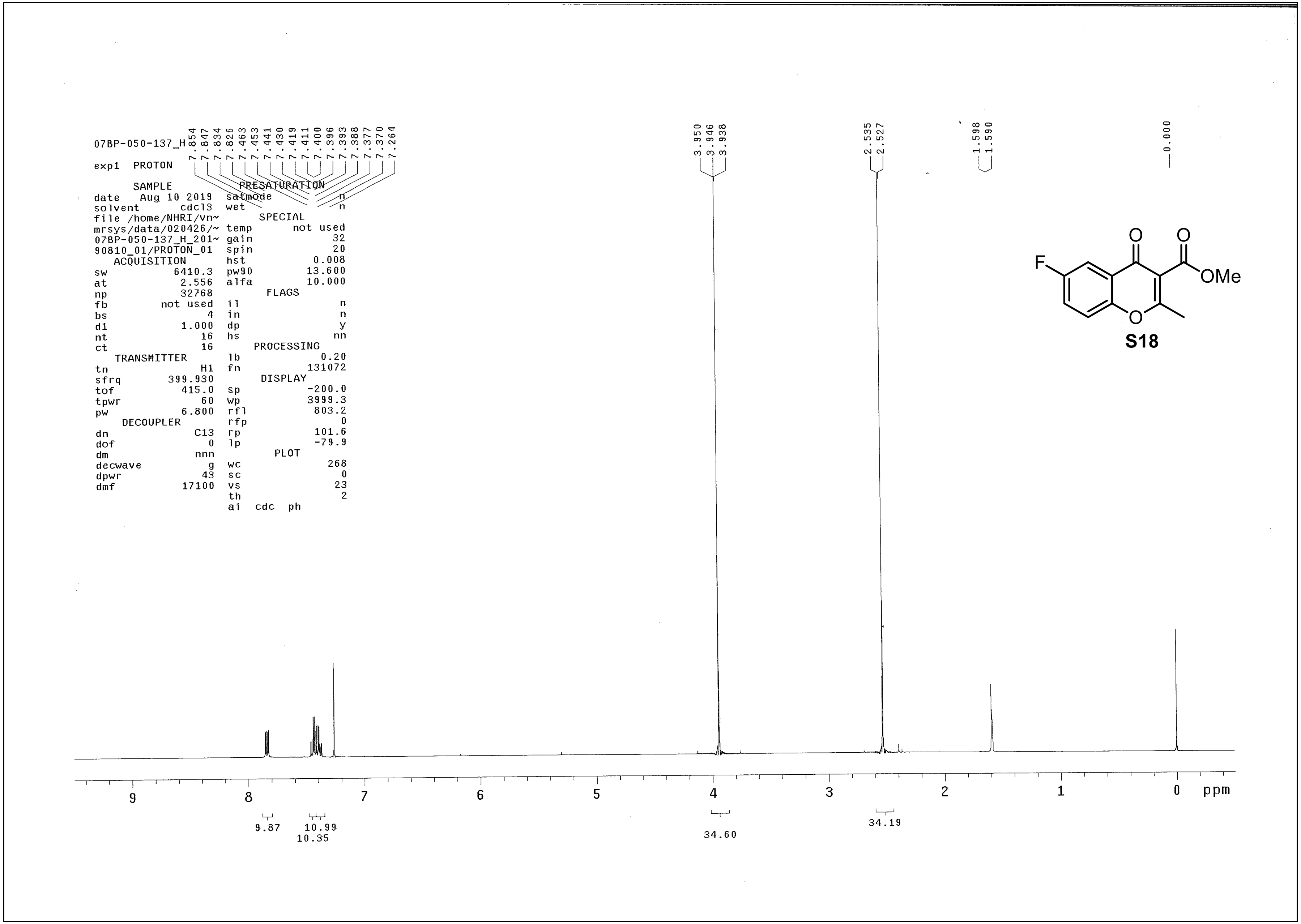

^{1}H NMR spectra for compound **S18**

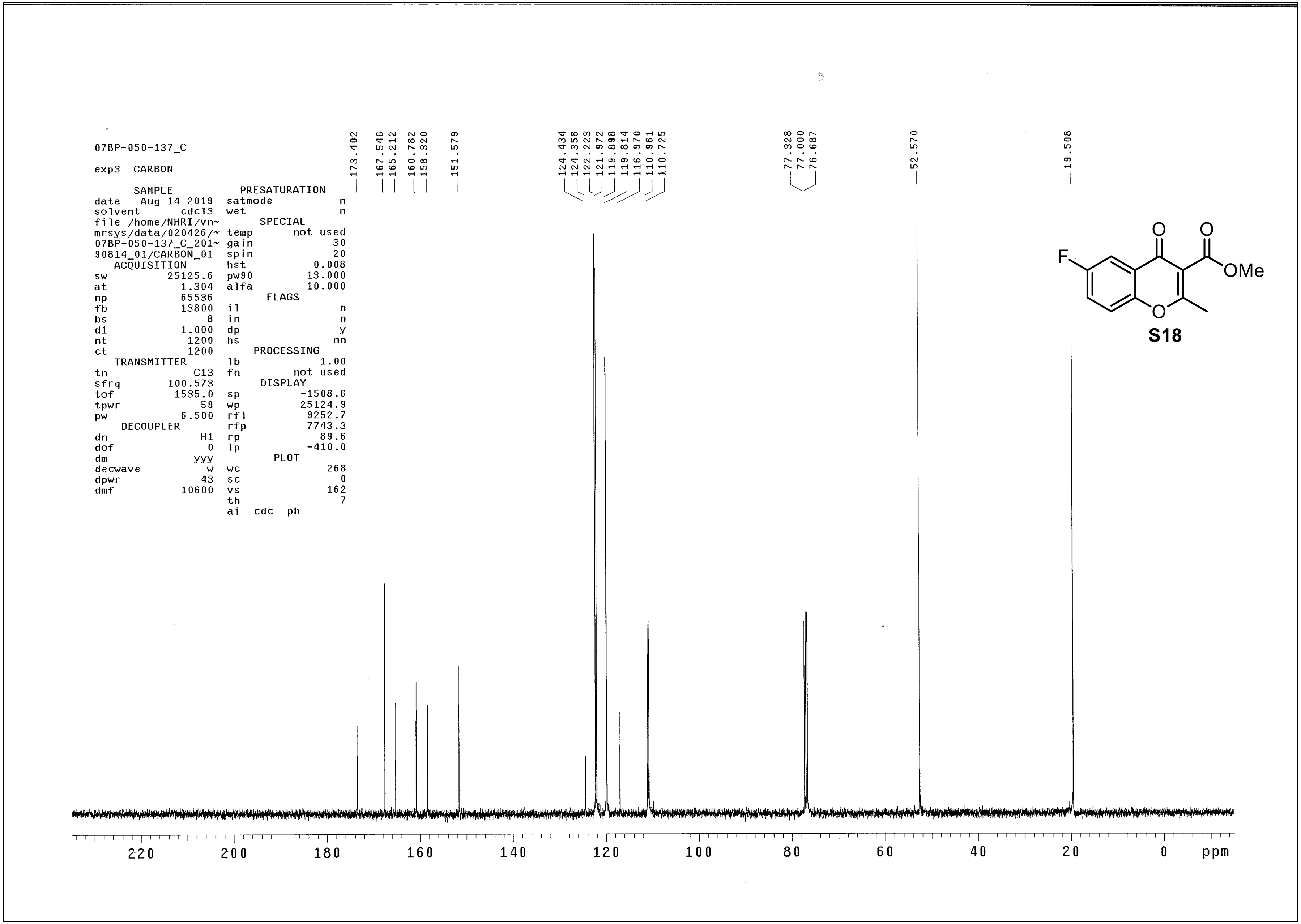

^{13}C NMR spectra for compound **S18**

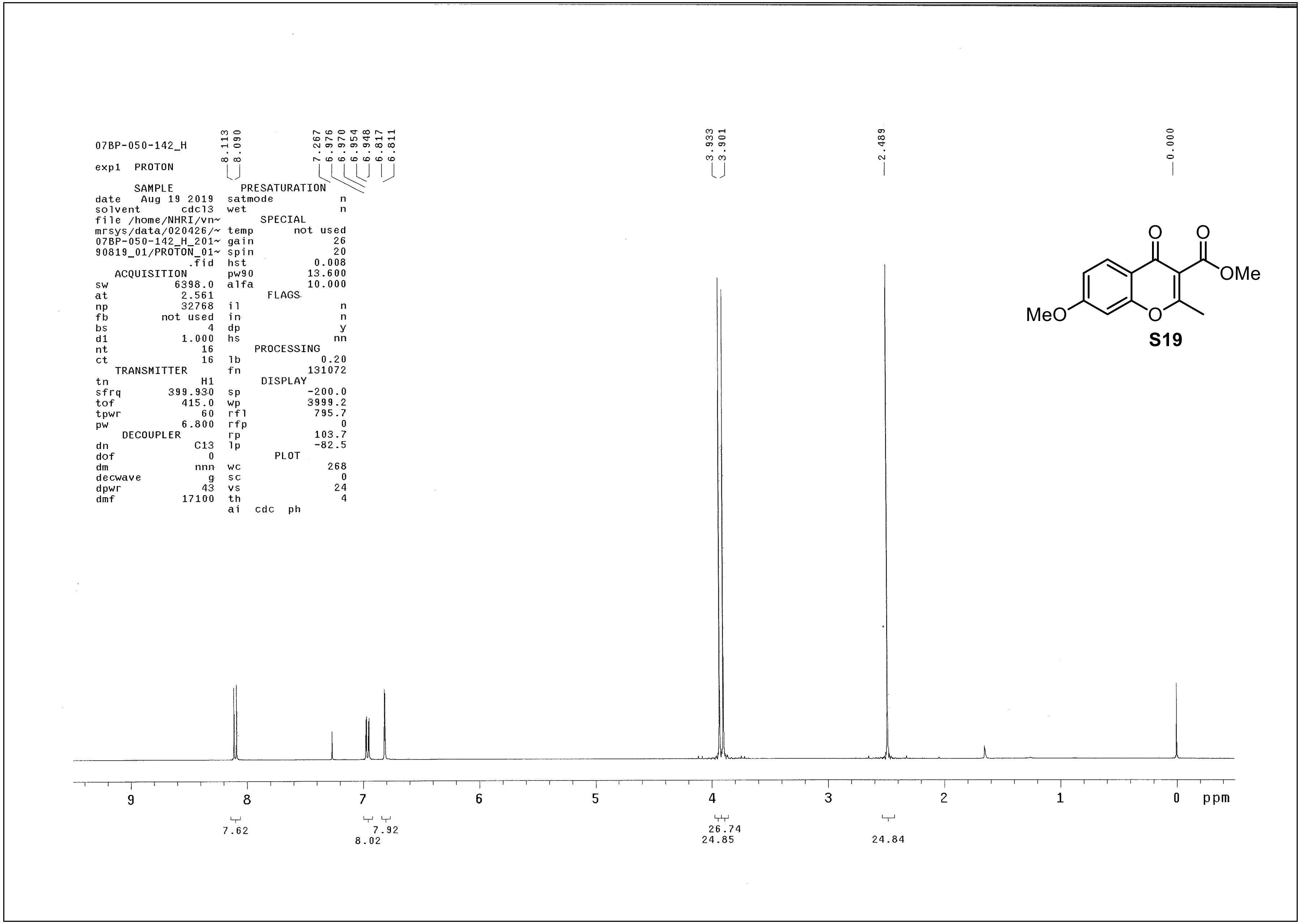

^{1}H NMR spectra for compound **S19**

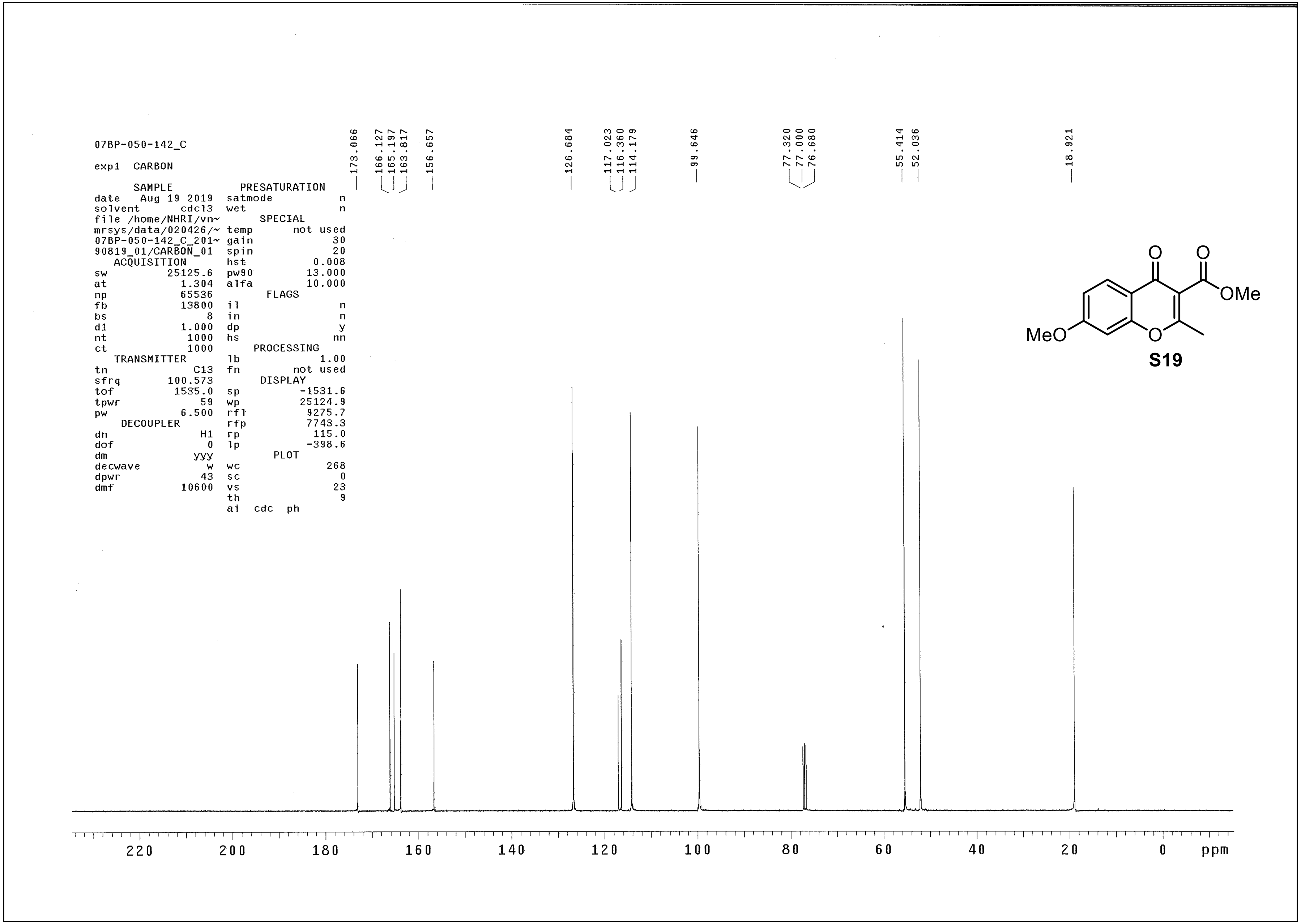

^{13}C NMR spectra for compound **S19**

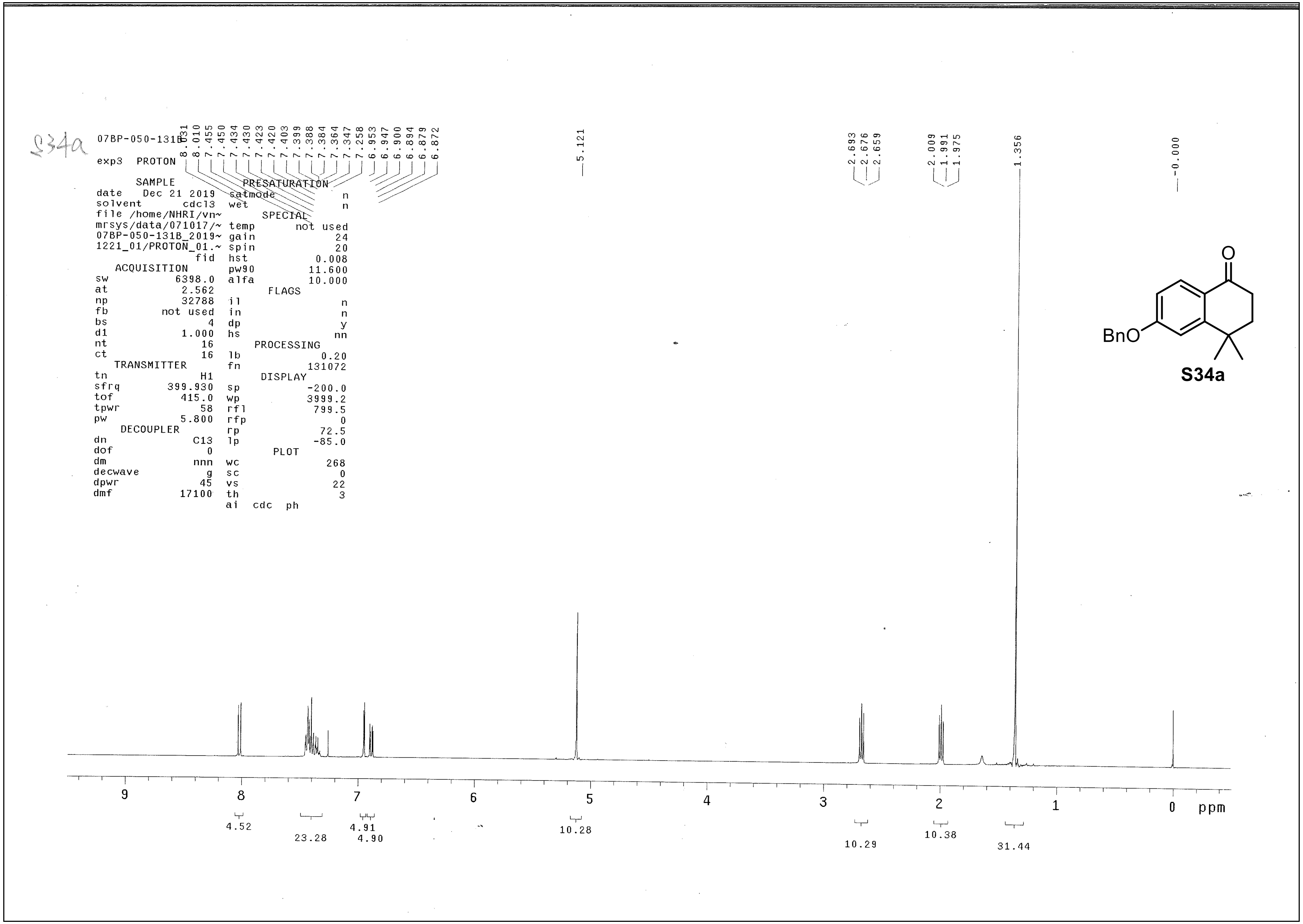

^{1}H NMR spectra for compound **S34a**

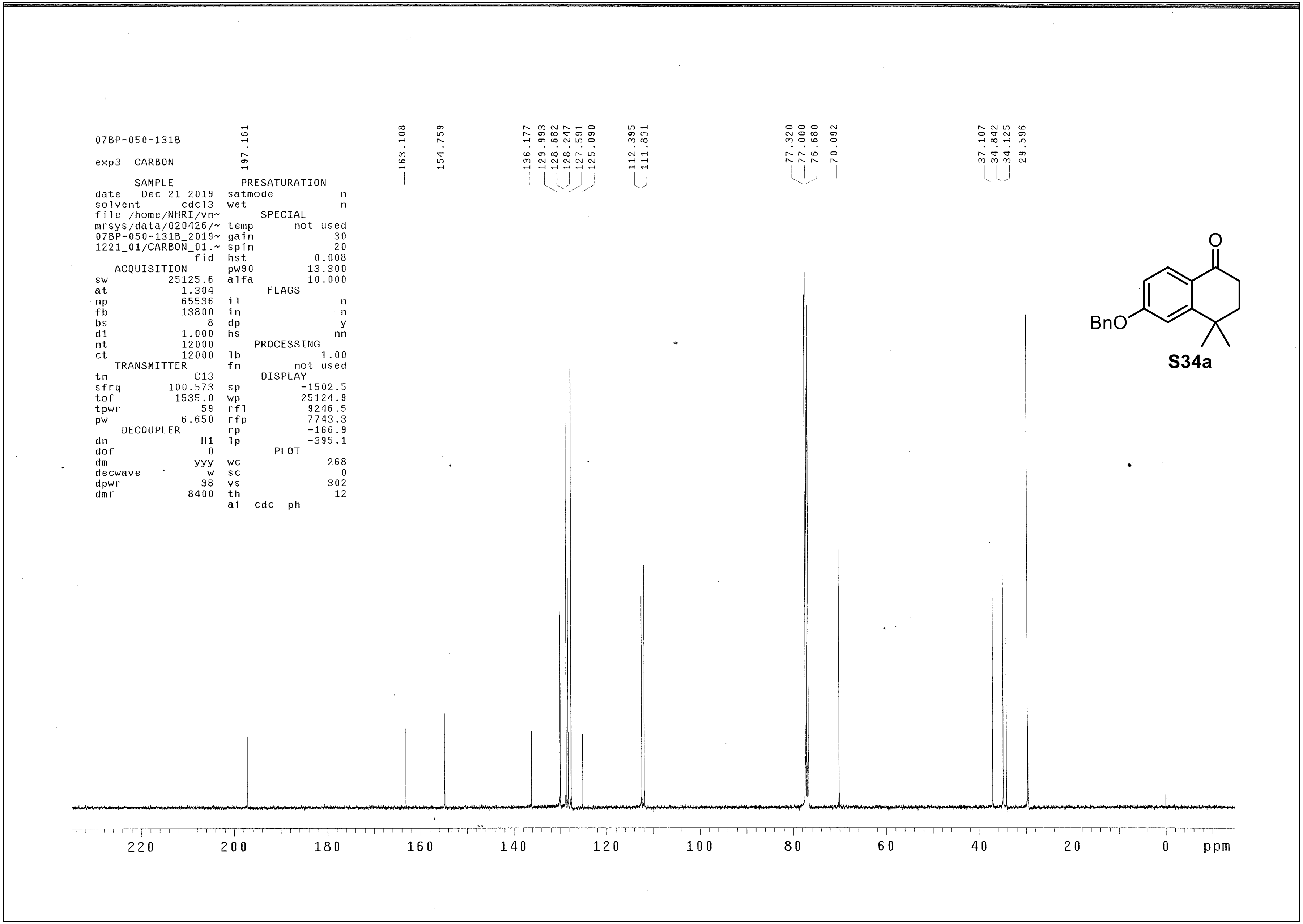

^{13}C NMR spectra for compound **S34a**

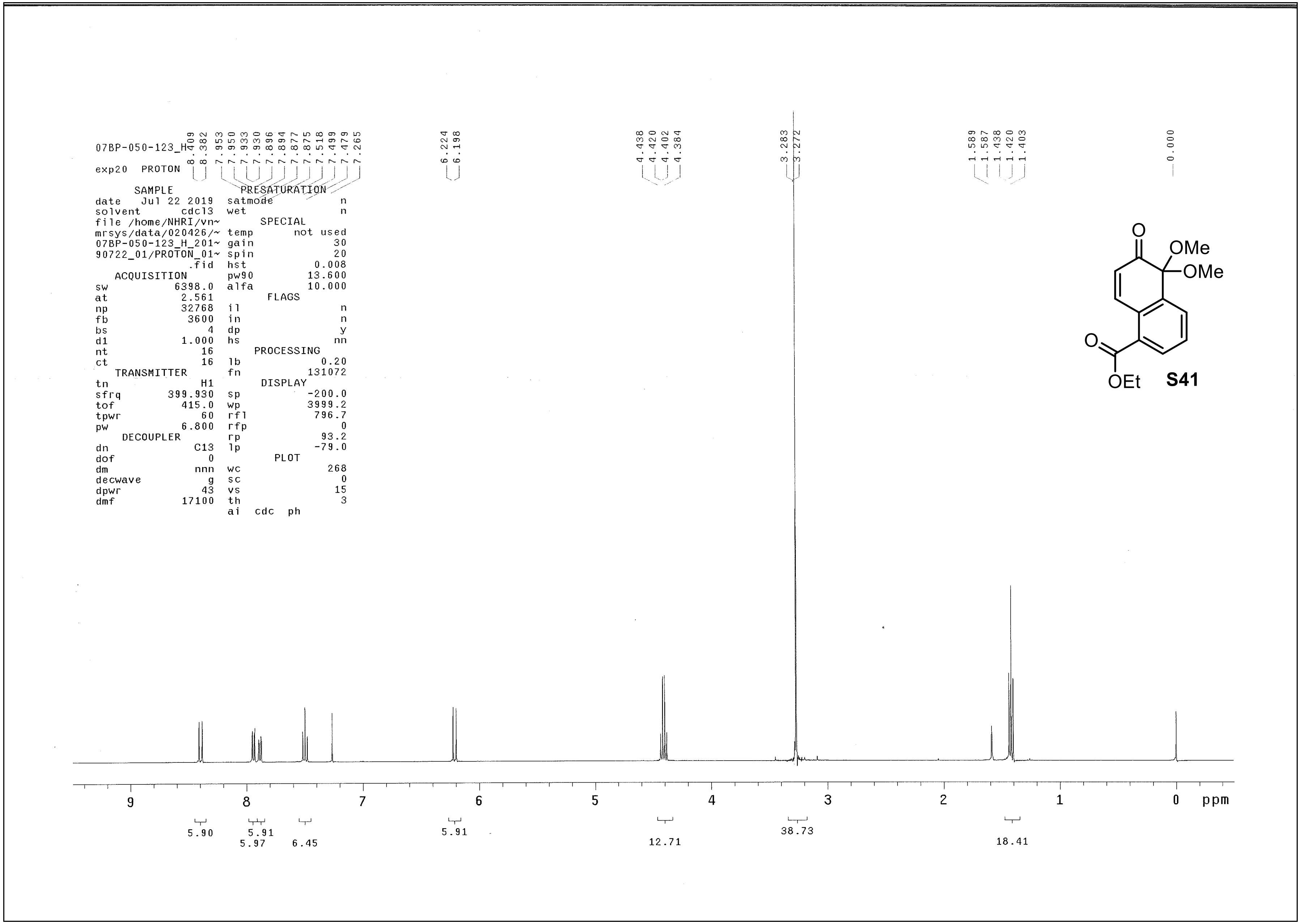

^{1}H NMR spectra for compound **S41**

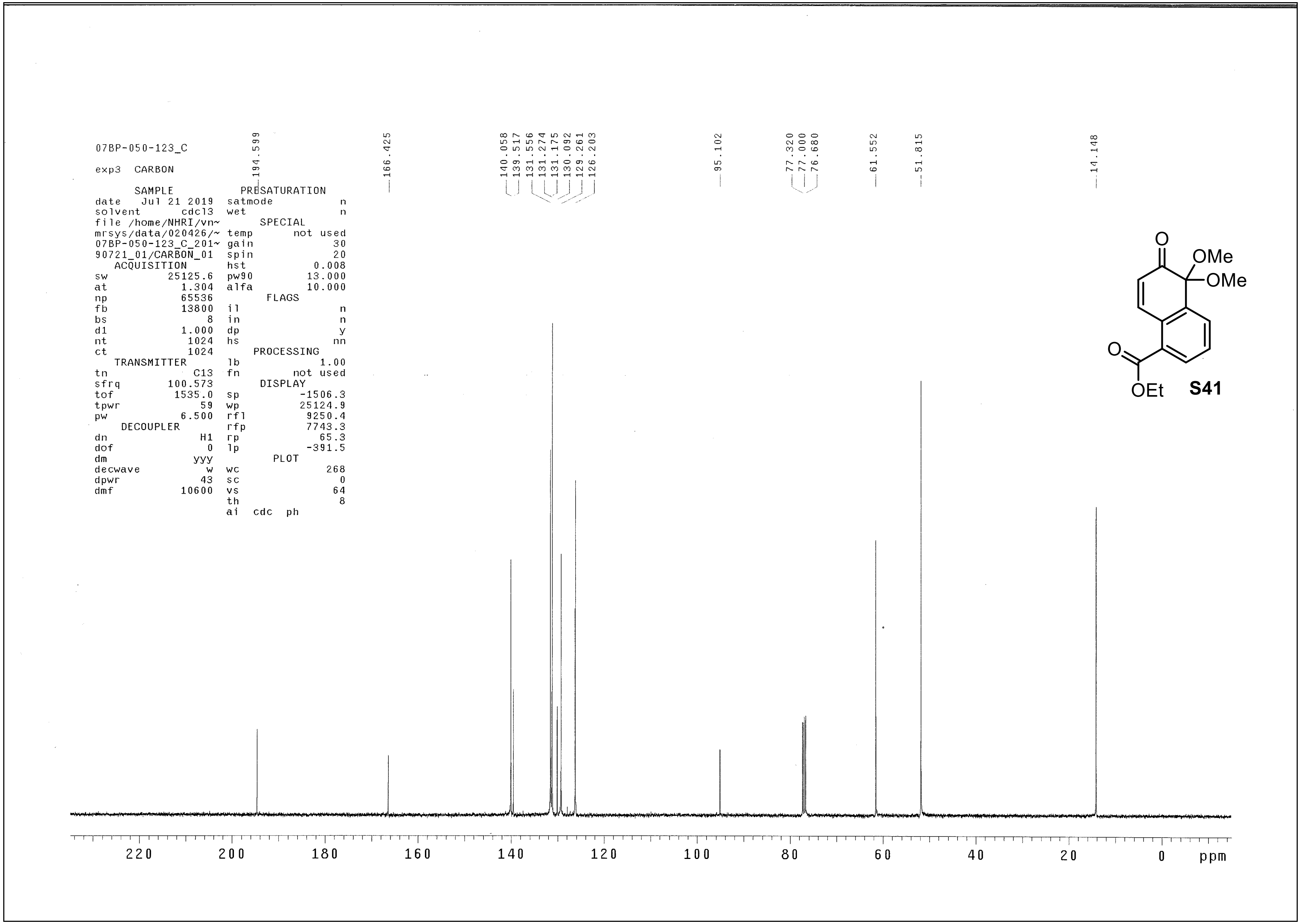

^{13}C NMR spectra for compound **S41**

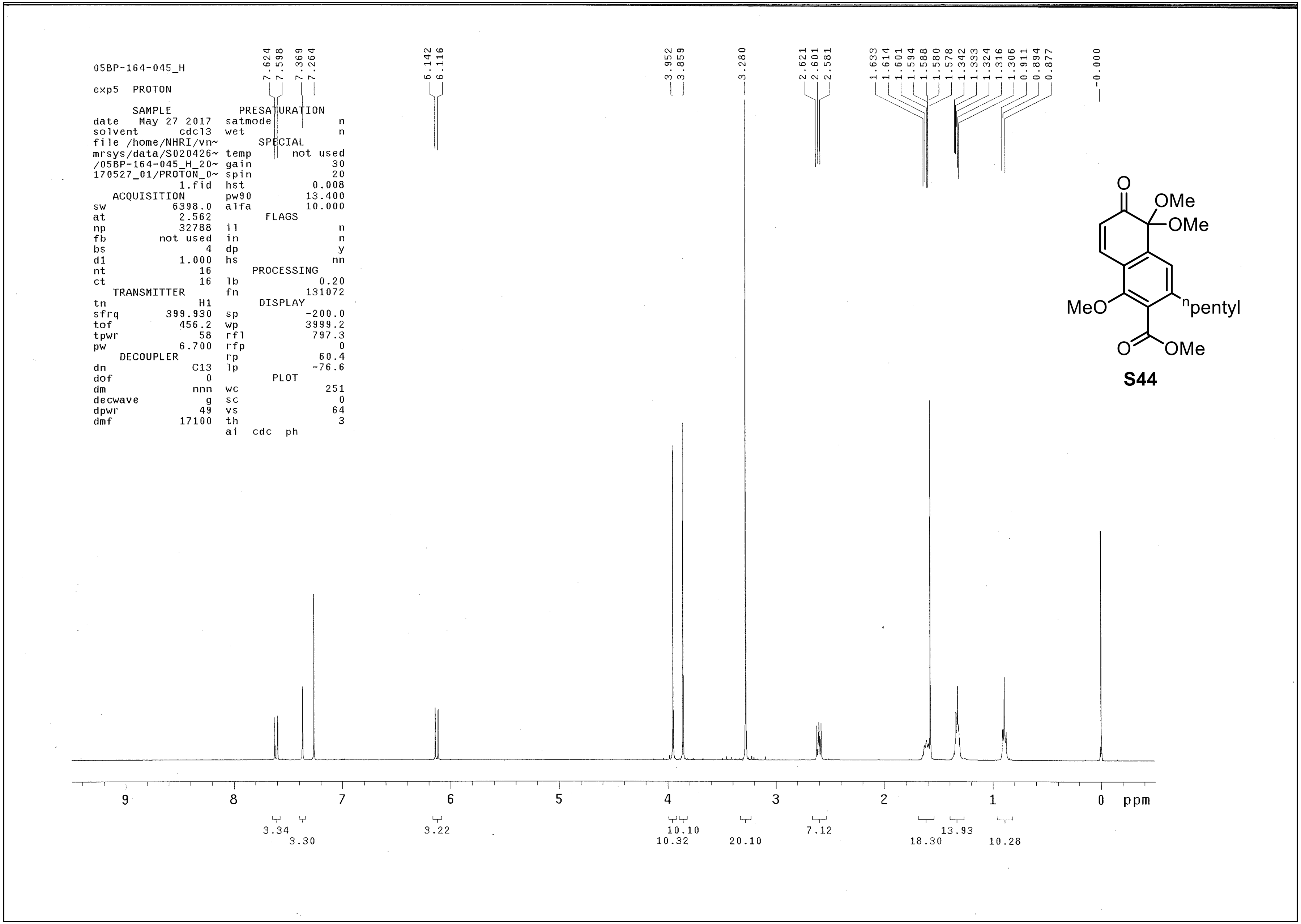

^{1}H NMR spectra for compound **S44**

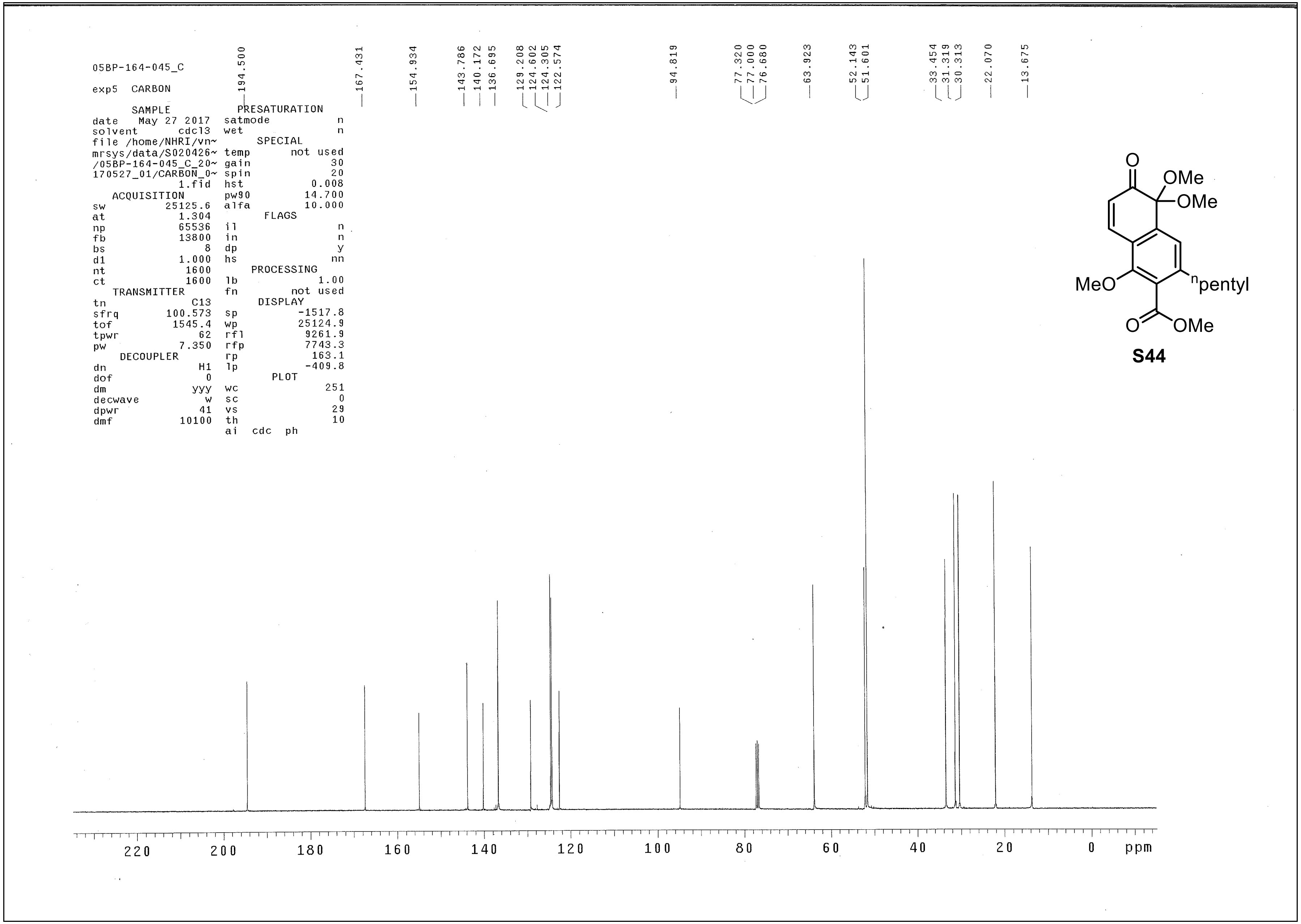

^{13}C NMR spectra for compound **S44**

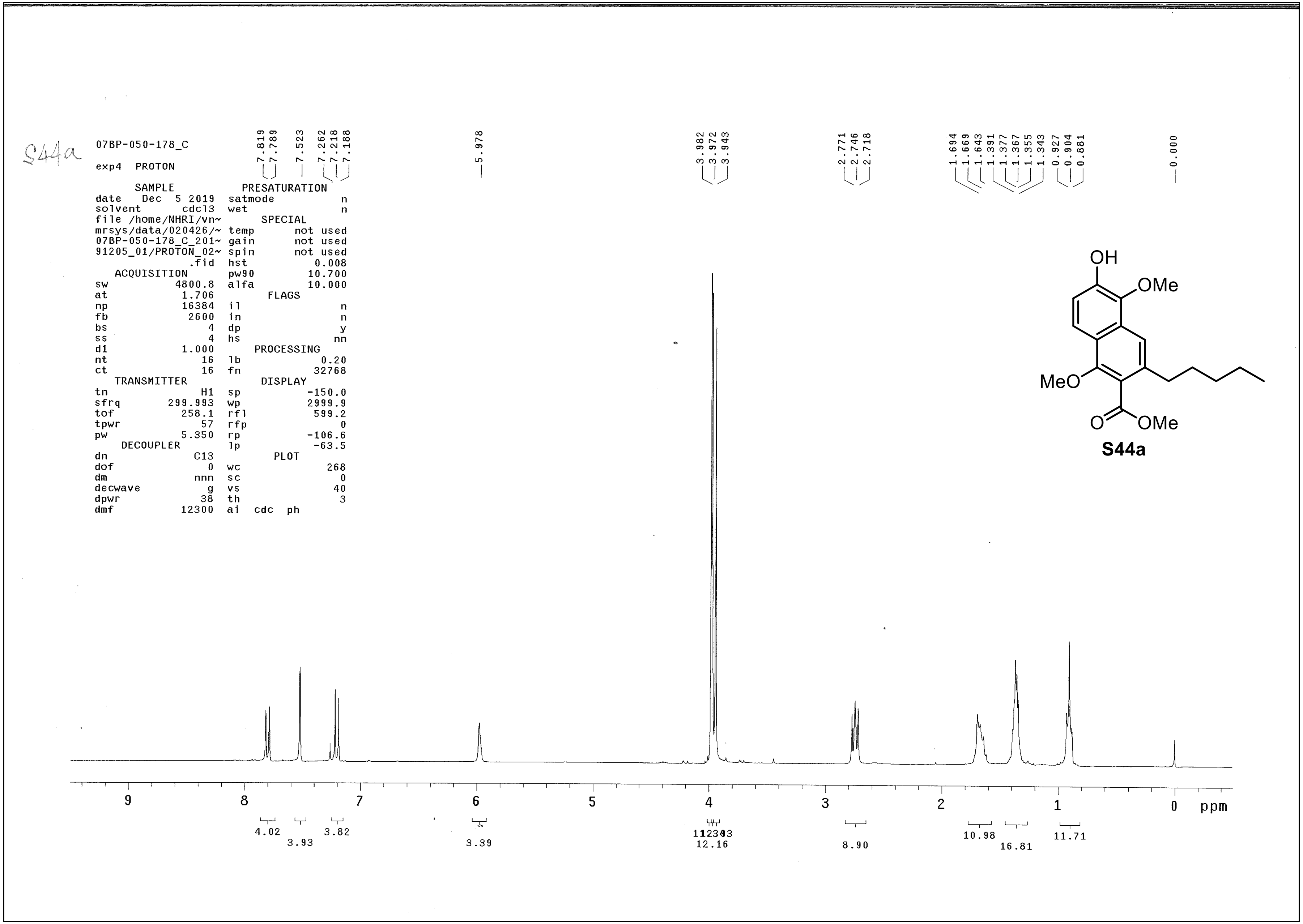

^{1}H NMR spectra for compound **S44a**

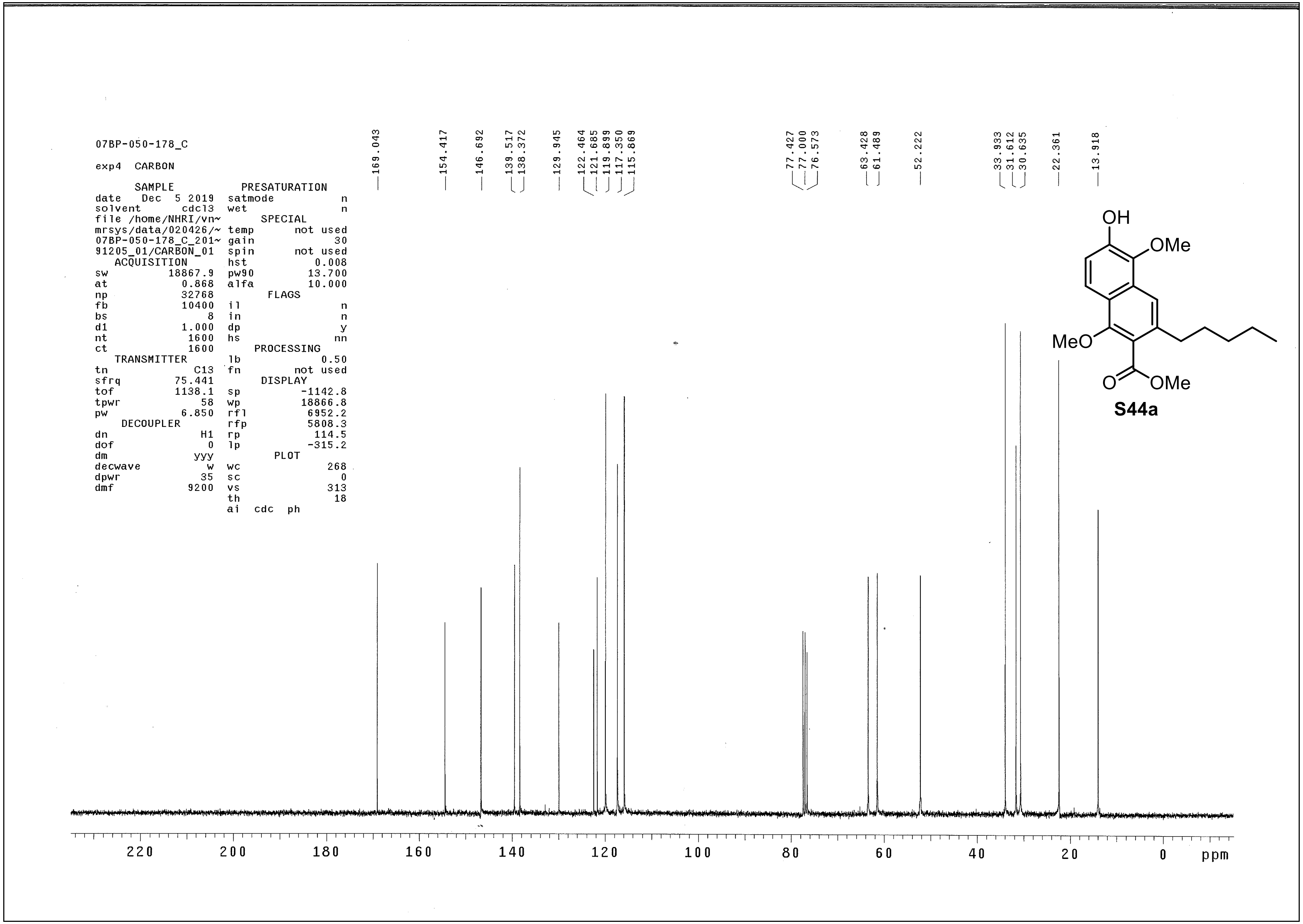

^{13}C NMR spectra for compound **S44a**

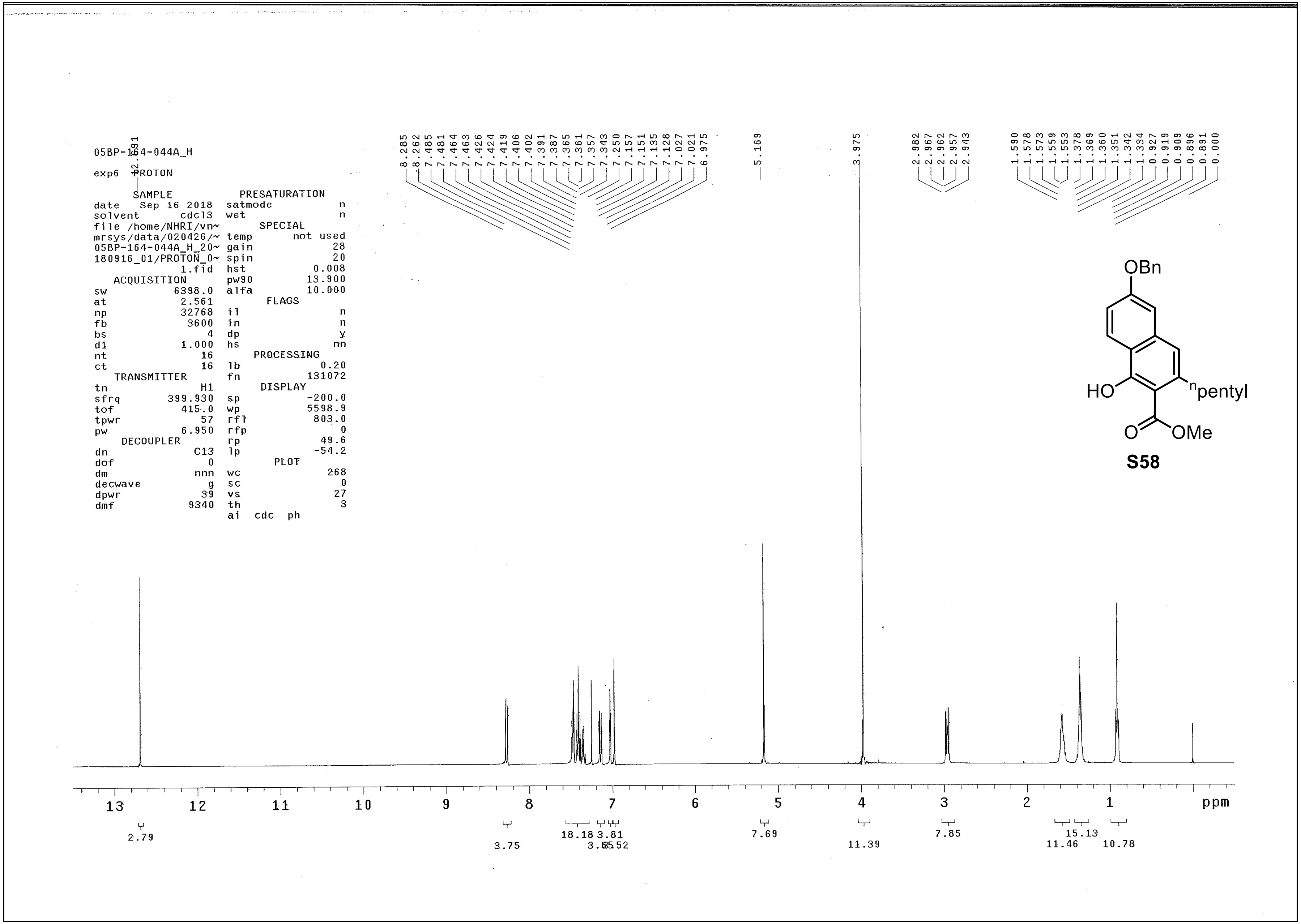

^{1}H NMR spectra for compound **S58**

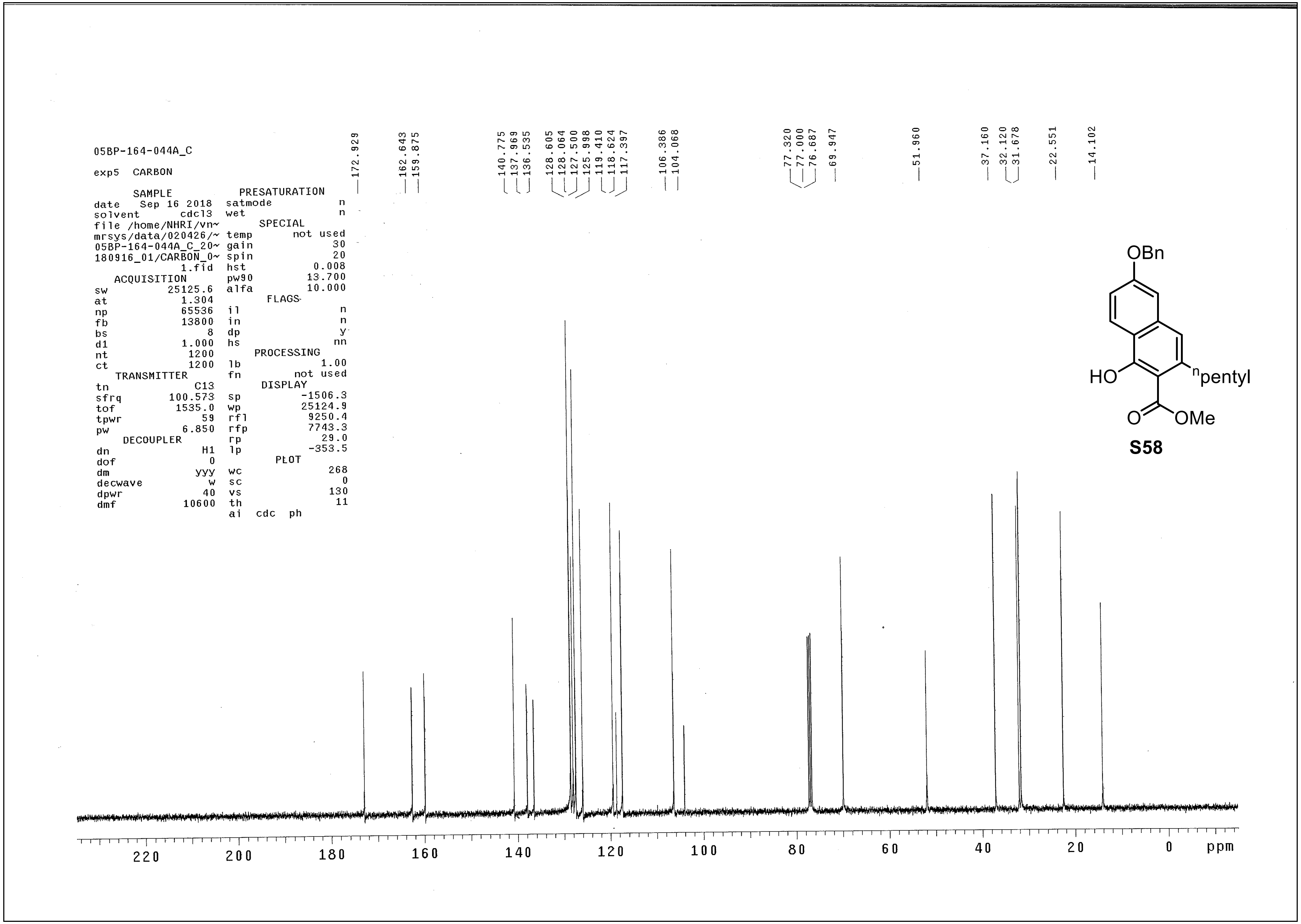

^{13}C NMR spectra for compound **S58**

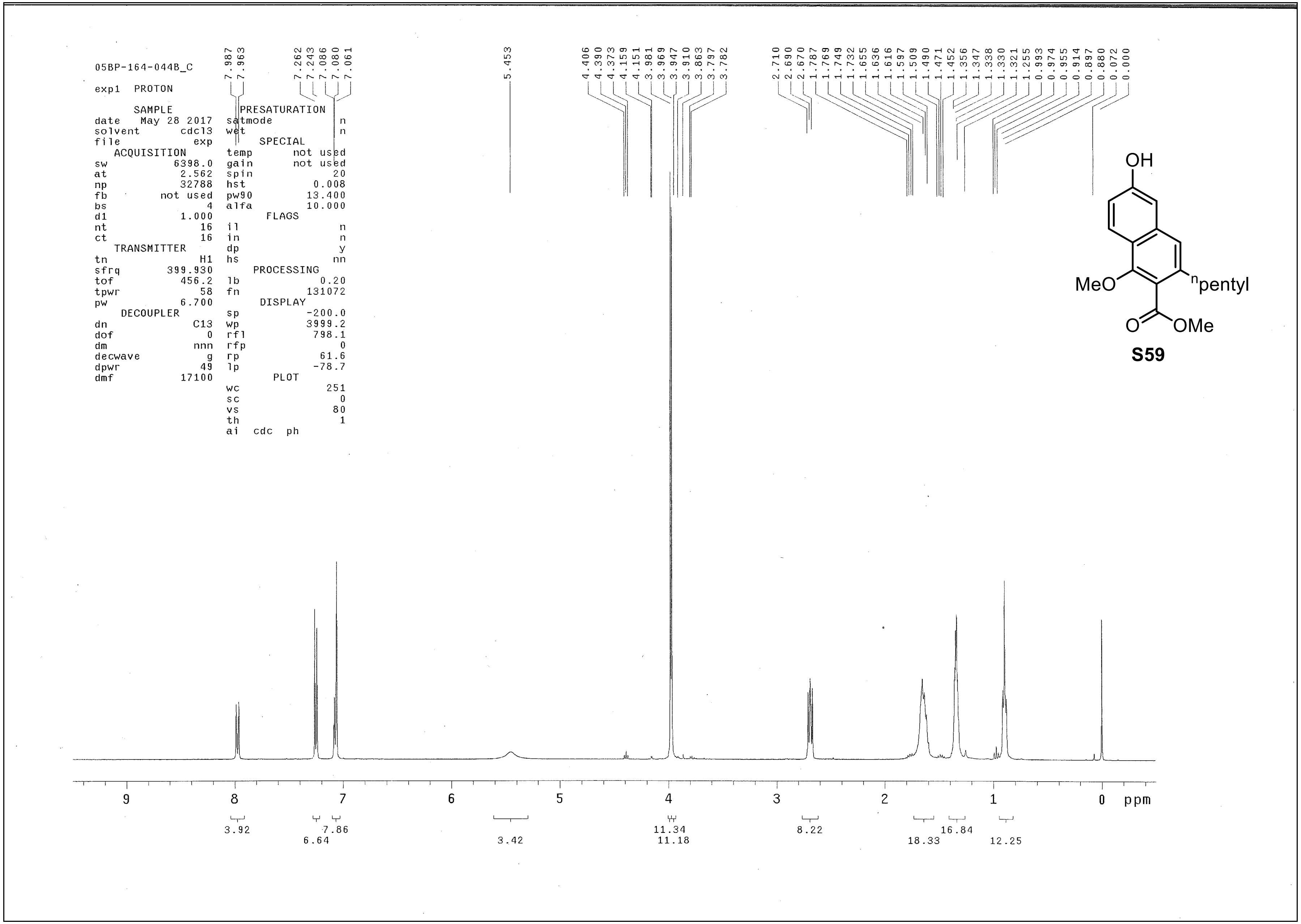

^{1}H NMR spectra for compound **S59**

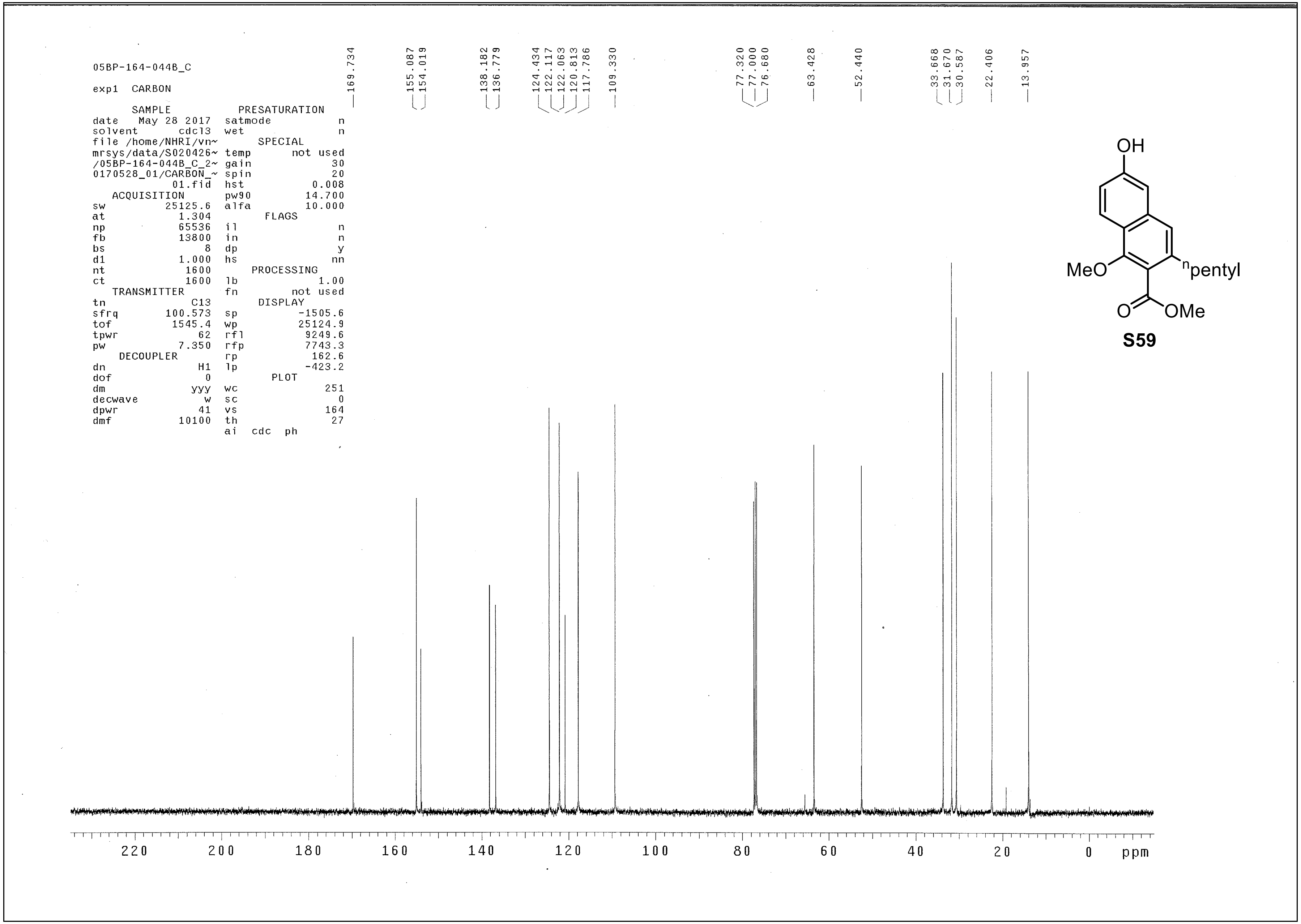

^{13}C NMR spectra for compound **S59**
